# Supplementary material for: The 4.2 ka event is not remarkable in the context of Holocene climate variability
Source: Nat Commun. 2024 Aug 2;15:6555. doi: 10.1038/s41467-024-50886-w (PMC11297131; doi:10.1038/s41467-024-50886-w)
Supplement: Supplementary file 1 — Supplementary Information [file 41467_2024_50886_MOESM1_ESM.pdf]

Supplementary Information

The 4.2 ka event is not remarkable in the context of Holocene climate variability

McKay, Nicholas P.; Kaufman, Darrell S.; Arcusa, Stéphanie; Kolus, Hannah; Edge, David; Erb, Michael P.; Hancock, Chris; Routson, Cody C.; Żarczyński, Maurycy; Marshall, Leah; Roberts, Georgia; Telles, Frank

Corresponding Author: Nicholas P. McKay
Email: Nicholas.McKay@nau.edu

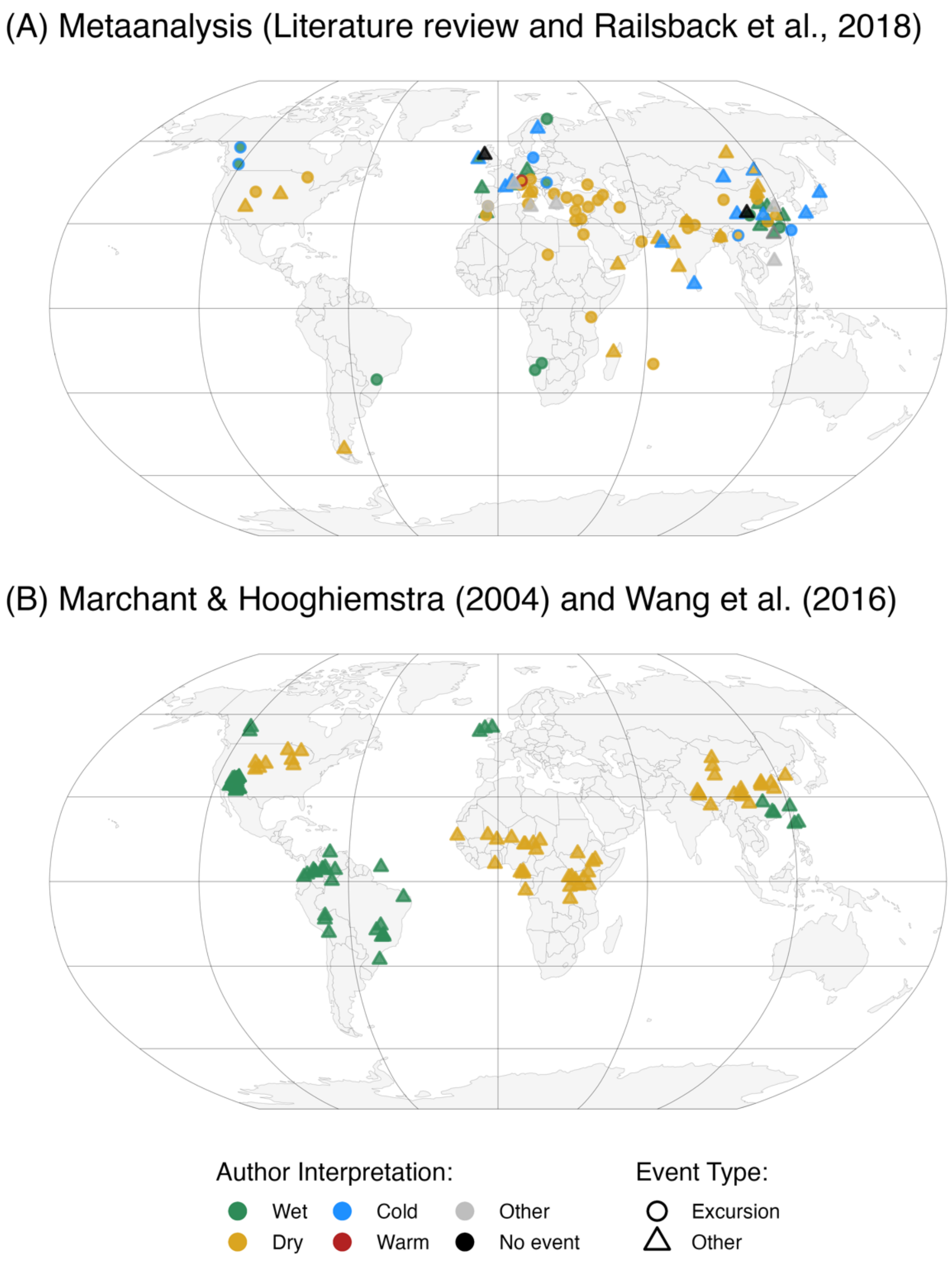

Fig. S1. *Map of proxy records describing the 4.2 event in our meta-analysis. Colors indicate the climate response described by the original author. If both temperature and hydroclimate are described, this multivariate response is shown by differing edge and fill colors. Circles show datasets interpreted as excursions; all other change types shown as triangles. Black indicates where the authors specifically indicate that no 4.2 ka event occurred in the record. Gray circles indicate that the authors did not interpret a definitive wet/dry or warm/cold expression of the 4.2 event. Other indicates where authors described a change in climate other than temperature or hydroclimate.*

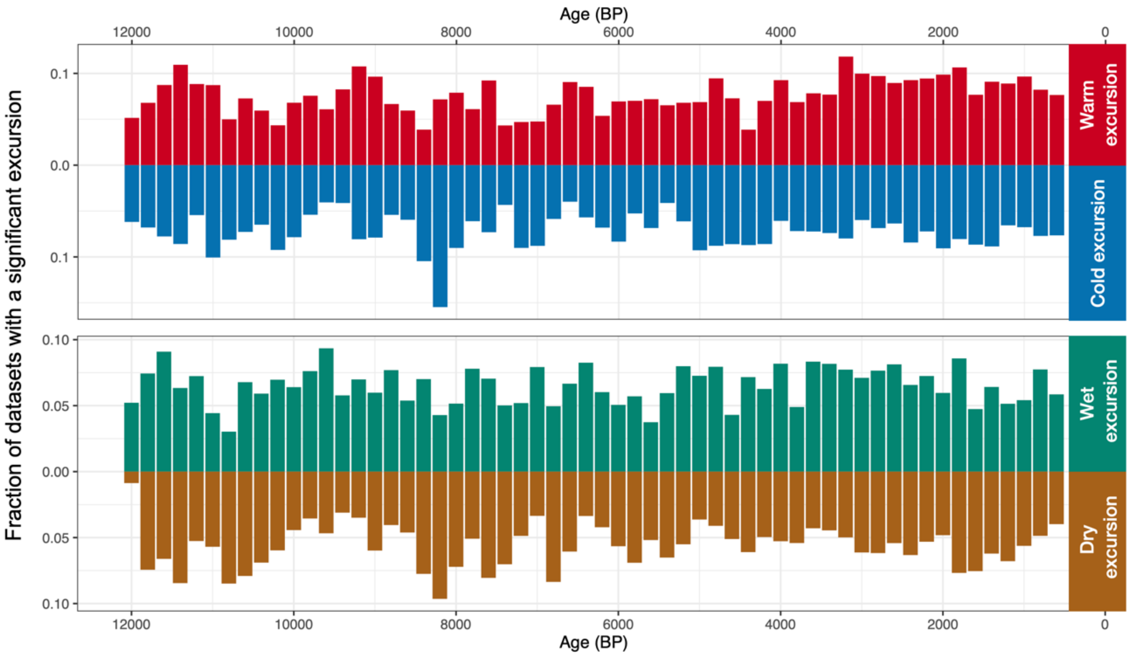

Fig. S2. *Fraction of datasets with at least one significant (p < 0.05) climate excursion through the Holocene. This figure differs from Figure 3 in that it does not consider spatial weighting, show the fraction of significant results in the parametric ensemble or significance beyond the site-level. Each bar simply shows the fraction of significant (p < 0.05) warm, cold, wet and dry excursions in 400 +/- 100 year windows. The axes for cold and dry excursions are inverted. The excursion detection analysis is repeated in 200-year intervals across the Holocene*

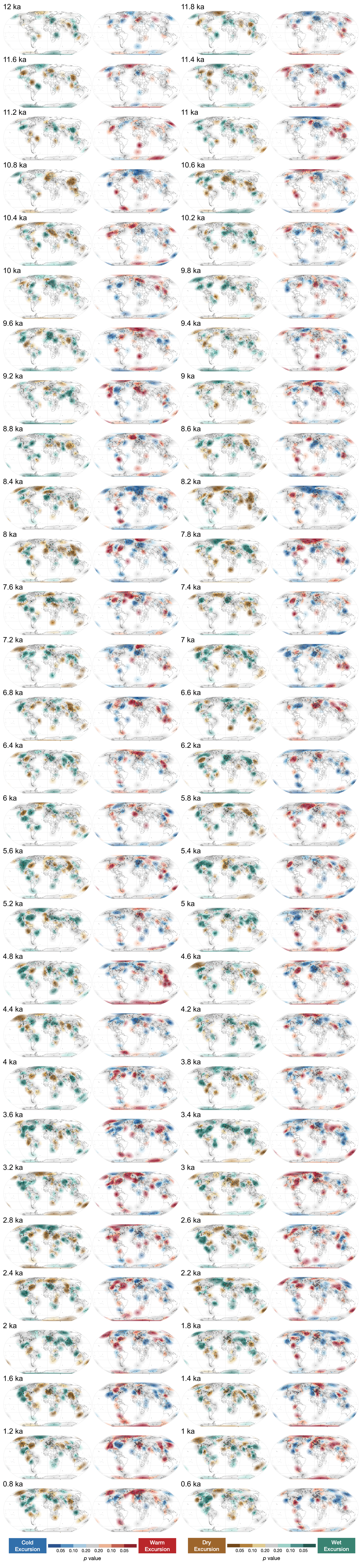

Fig. S3. *Spatial expression of moisture (columns 1 and 3) and temperature (columns 2 and 4) excursions for 200-yr sliding windows from 12,000 to 600 yr BP. Time period labels correspond to hydroclimate map immediately below each label, and the adjacent temperature map to the right. The grid shading indicates the significance of regional net excursions. Darker red indicates more significant net temperature excursions (i.e., the number of warm excursions in a region significantly exceeds the number of cold excursions). Blue colors indicate regions where cold excursions significantly exceed the number of warm excursions. Green (wet) and brown (dry) colors express regional net excursions for hydroclimate. Colored dots show the location of records with significant excursions at the 0.05 level, colored similarly to grid shading. White circles indicate no significant excursions at a site for the specified time period.*

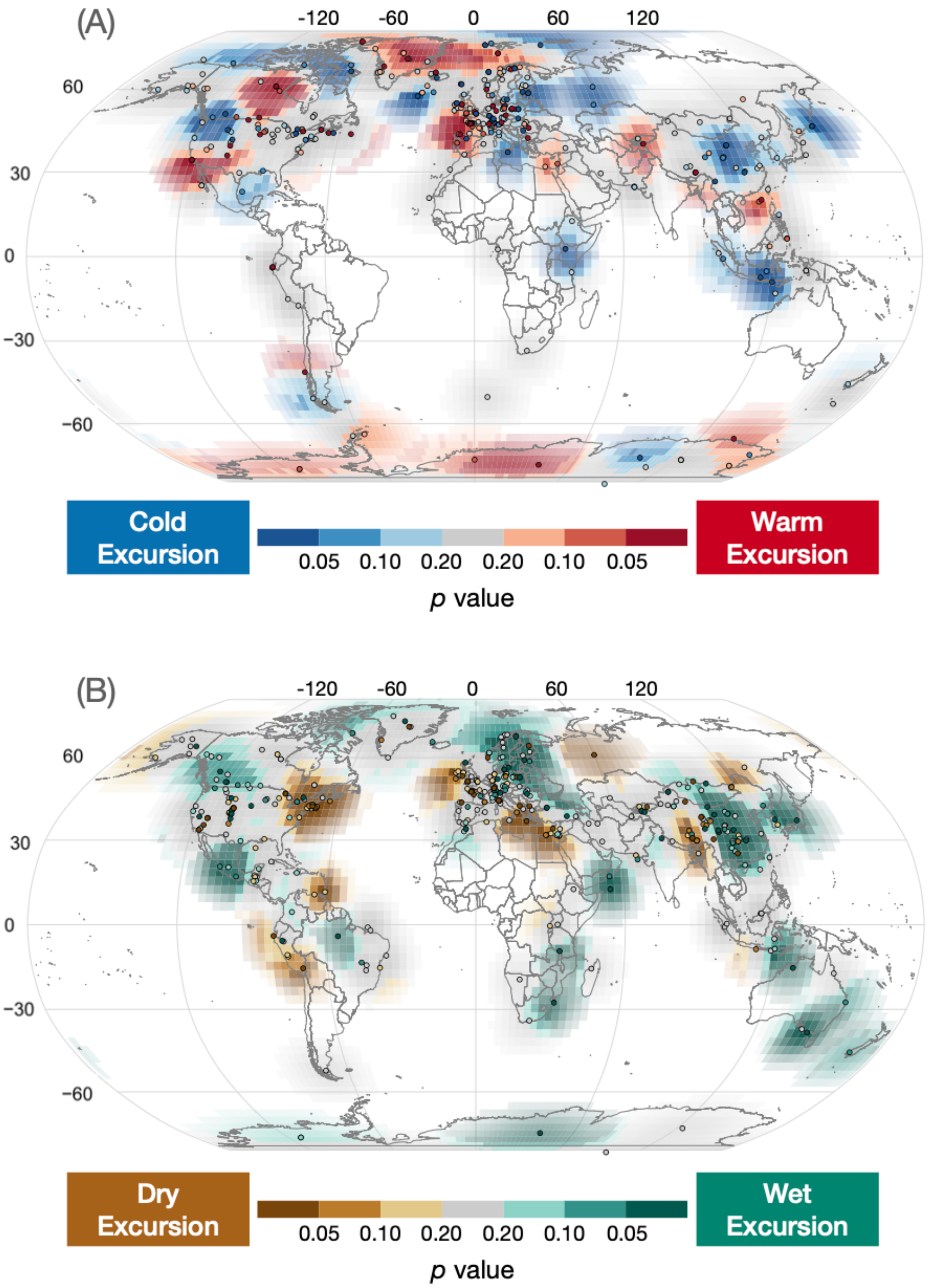

Fig. S4. *Spatial expression of the most significant temperature (A) and moisture (B) excursions found at 4.0 or 4.2 ka. At each site and for each variable, the most significant excursion in either direction was selected before calculating regional net excursions as in Fig 3. The grid shading indicates the significance of regional net excursions. Darker red indicates more significant net temperature excursions (i.e., the number of warm excursions in a region significantly exceeds the number of cold excursions). Blue colors indicate regions where cold excursions significantly exceed the number of warm excursions. Green (wet) and brown (dry) colors express regional net excursions for hydroclimate. Colored dots show the location of records with significant excursions at the 0.05 level, colored similarly to grid shading. White circles indicate no significant excursions at a site for the specified time period.*

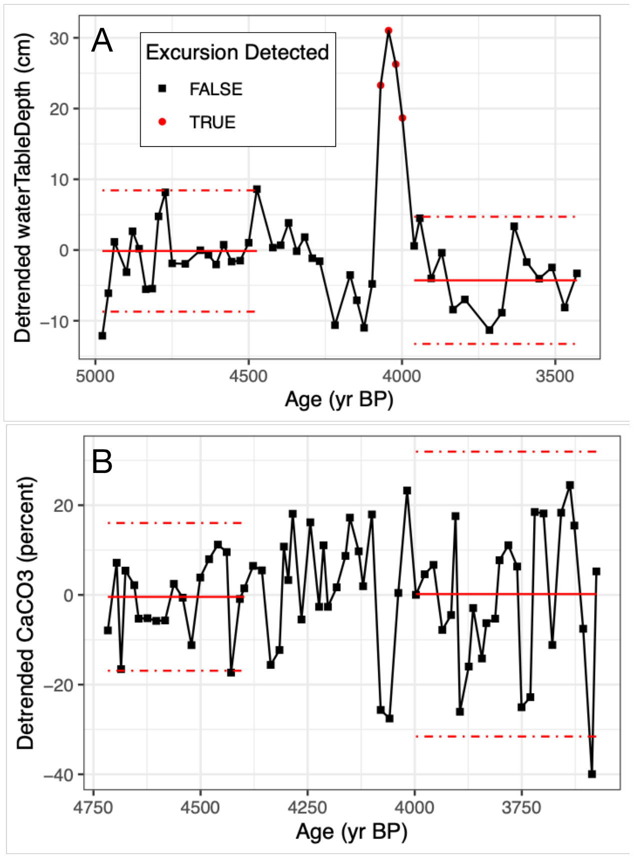

Fig. S5. *Examples of the excursion detection algorithm applied to two segments of proxy climate timeseries: (A) water table depth inferred from testate amoeba (Booth et al., 2005) and (B) effective precipitation inferred from CaCO3 concentration (Hodell et al., 1995). The red solid and dashed lines represent the mean and 2σ ranges of the reference windows. In panel A, an excursion is identified as four consecutive observations in the event window exceed the reference range. In panel B, no excursion is identified.*

Table S1. Results of metanalysis, including reference to paper, site metadata, and interpreted timing, duration and climate interpretation of the 4.2 ka event.

Reference	Source	Site name	Lat (°)	Lon (°)	Archive	Proxy	Change type	Climate signal	Excursion start (ka)	Excursion end (ka)
Amekawa et al. (2016)	Literature search	Bagasra (BSR7)	23.3	67.5	Marine sediment	d18O	Indeterminant: 3 year record	Cold		
An et al. (2021)	Literature search	Multiple Haidai region	36.0	118.0	Terrestrial sediment	Macrofossils (botanical)	Event stratigraphy	Assumed deterioration		
Arenas et al. (2022)	Literature search	Cañada de La Enfermería	24.2	-110.3	Mollusk	d18O	Indeterminant: no overlap with 4.2 ka	No overlap with 4.2 ka		
Arz et al. (2006)	Railsback et al. 2018	GeoB5846-2	26.2	35.4	Marine sediment	d18O	Excursion	Dry	4.20	4.05
Azennoud et al. (2022)	Literature search	Saiss Basin	33.7	-5.0	Terrestrial sediment	Tufa	Hiatus	Wet		
Bar-Matthews et al. (1999)	Railsback et al. 2018	Soreq Cave	31.8	35.0	Speleothem	d18O	Excursion	Dry	4.2	4.0
Berger et al. (2012)	Literature search	Hadramawt	15.5	48.6	Terrestrial sediment	Statigraphy	Event stratigraphy	Dry		
Berkelhammer et al. (2012)	Railsback et al. 2018	Mawmluh Cave (KM-A)	25.3	91.9	Speleothem	d18O	Excursion	Dry	4.1	3.9
Bliedtner et al. (2022)	Literature search	Shireet Naiman Nuur	46.5	101.8	Lake sediment	Multiple	Mean shift	Cold		
Booth et al. (2004)	Railsback et al. 2018	South Rhody Peatland	46.6	-86.1	Peat	Testate amoebae	Excursion	Dry	4.1	4.0
Booth et al. (2005)	Literature search	Multiple mid-west North America	40.5	-95.0	Terrestrial sediment	Dune	Event stratigraphy	Dry		
Brisset et al. (2012)	Literature search	Lac Petit	44.1	7.2	Lake sediment	pollen	Not climate	Not climate		
Carolin et al. (2019)	Literature search	Gol-e-Zard cave	35.8	52.0	Speleothem	Mg/Ca	Excursion	Dry	4.26	3.97
Chase et al. (2009)	Railsback et al. 2018	Spitzkoppe	-21.8	15.2	Midden	d15N	Excursion	Wet	4.15	3.50
Cheng et al. (2021)	Literature search	Multiple China			Speleothem	Multiple	Various	Various		
Cheng et al. (2023)	Literature search	Subei Plain (SPM1)	32.7	120.2	Terrestrial sediment	Multiple	Mean shift	Wet		
Comas-Bru et al. (2022)	Literature search	Multiple global			Speleothem	Multiple	Various	Various		
Constantin et al. (2007)	Railsback et al. 2018	Poleva Cave	44.7	21.8	Speleothem	d18O	Excursion	Wet & cold	4.4	4.2
Cullen et al. (2000)	Railsback et al. 2018	Core M5-422	23.7	59.0	Marine sediment	Dolomite %	Excursion	Dry	4.1	3.9
Dang et al. (2020)	Literature search	Yongxing Island	16.8	112.3	Coral	Growth rate	Variablity shift	ENSO variance		
Dean et al. (2015)	Literature search	Nar Gölü	38.3	34.5	Lake sediment	d18O & mineralogy	Excursion	Dry	4.30	4.15
Di Rita & Magri (2019)	Literature search	Multiple central Mediterranean	37.0	13.0	Lake sediment	Pollen	Excursion	Dry		
Di Rita & Magri (2019)	Literature search	Multiple central Mediterranean	44.0	9.0	Lake sediment	Pollen	No event	NA		
Di Rita et al. (2018)	Literature search	Gulf of Gaeta	41.0	13.8	Marine sediment	Pollen	Mean shift	Dry		
Di Rita et al. (2022)	Literature search	Crovani Pond	42.5	8.7	Lake sediment	Pollen	No event	NA		
Drake et al. (2012)	Literature search	Chaco Canyon	36.0	-108.0	Midden	Pollen	Mean shift	Dry		
Drysdale (2006)	Railsback et al. 2018	Buca della Renella	44.0	10.2	Speleothem	Mg/Ca	Excursion	Dry	4.1	3.8
Dutt et al. (2021)	Literature search	Multiple India			Multiple	Multiple	Various	Various		
Fedotov et al. (2021)	Literature search	Lake Frolikha	55.4	110.0	Lake sediment	Multiple	Inflection	Dry		
Gao et al. (2022)	Literature search	Multiple	37.0	112.0	Terrestrial sediment	Flood frequency	Event stratigraphy	Wet		
Geirsdottir et al. (2019)	Literature search	Multiple Iceland	65.0	21.0	Lake sediment	Multiple	Mean shift	Cold		
Grachev et al. (2021)	Literature search	Lake Khuko	43.9	39.8	Lake sediment	Multiple	Excursion	Dry	4.2	3.5
Groucutt et al. (2022)	Literature search	Multiple Malta	36.0	14.0	Terrestrial sediment	Archaeological	Various	Various		
Hazell et al. (2022)	Literature search	Akrotiri Marsh	34.6	32.9	Terrestrial sediment	Pollen & diatom	Excursion	Dry	4.6	4.2
Hazell et al. (2022)	Literature search	Multiple eastern Mediterranean			Multiple	Multiple	Various	Various		
He et al. (2022)	Literature search	Multiple China			Terrestrial sediment	Macrofossils (botanical)	Event stratigraphy	Assumed deterioration		
Helama and Oinonen (2019)	Literature search	Finnish Lapland	69.0	27.0	Wood	d13C	Excursion	Wet	4.14	4.05
Hu et al. (2020)	Literature search	Xisha Islands	17.1	113.5	Mollusk	d18O	Indeterminant: 40-yr record around 3.7 ka	No overlap with 4.2 ka		
Isola et al. (2019)	Literature search	Corchia Cave (CC27)	44.0	10.3	Speleothem	Multiple	Excursion	Dry	4.5	4.1
Jaffe et al. (2021)	Literature search	Multiple China			Terrestrial sediment	Archaeological	Various	Various		
Jalali and Sicre (2019)	Literature search	KSGC-31	43.0	3.3	Marine sediment	Uk37	Mean shift	Cold		
Jarriel (2021)	Literature search	Cycladic islands	37.0	25.0	Multiple	Archaeological	Event stratigraphy	Assumed deterioration		
Jia et al. (2021)	Literature search	Sanfangwan	30.8	113.1	Terrestrial sediment	Pollen	Event stratigraphy	Wet		
Jia et al. (2021)	Literature search	Nanyang Basin	33.0	112.0	Terrestrial sediment	Macrofossils	Mean shift	Cold		
Jordan et al. (2017)	Search & Railsback et al. 2018	Spiddal (Galway)	53.2	-9.3	Peat	n-Alkane	Indeterminant: single point in low-resolution record	Wet & cold		

Table S1. Results of metanalysis, including reference to paper, site metadata, and interpreted timing, duration and climate interpretation of the 4.2 ka event.

Reference	Source	Site name	Lat (°)	Lon (°)	Archive	Proxy	Change type	Climate signal	Excursion start (ka)	Excursion end (ka)
Kajita et al. (2018)	Literature search	MD06-3040 (Yangtze Delta)	27.7	121.8	Marine sediment	Uk37	Excursion	Cold	4.4	3.8
Kajita et al. (2023)	Literature search	HIUB1–1 (Hakata Bay)	33.7	130.4	Marine sediment	Uk37	Mean shift	Cold		
Kang et al., (2023)	Literature search	HSL (Hunshandake Sandy Land)	43.0	115.0	Terrestrial sediment	Dune	Mean shift	Dry		
Kathayat et al. (2018)	Literature search	Mawmluh Cave (ML.1 & ML.2)	25.3	91.7	Speleothem	d18O & d13C	Mean shift	Dry		
Kawahata (2019)	Literature search	Mutsu Bay	41.0	140.8	Marine sediment	Uk37	Mean shift	Cold		
Kotlia and Joshi (2013)	Literature search	Badanital Lake	30.5	78.9	Lake sediment	Multiple	Mean shift	Dry		
Kropelin et al. (2008)	Railsback et al. 2018	Lake Yoa	19.0	20.3	Lake sediment	MS	Excursion	Dry	4.2	4.0
Laskar and Bohra (2021)	Literature search	Multiple India			Multiple	Multiple	Various	Various		
Le Roy et al. (2017)	Literature search	Ecrins-Pelvoux Massif	44.9	6.3	Moraines	Be10	Mean shift	Cold		
Lemcke and Sturm (1997)	Railsback et al. 2018	Lake Van	38.4	43.2	Lake sediment	Quartz %	Excursion	Dry	4.25	4.05
Li et al. (2018)	Literature search	La Vierge Cave (Rodrigues Island)	-19.7	63.4	Speleothem	d18O & d13C	Excursion	Dry	3.9	3.5
Li et al. (2021)	Literature search	Multiple	36.0	115.0	Terrestrial sediment	Flood frequency	Event stratigraphy	Wet		
Liefert and Shuman (2022)	Literature search	Highway 130 Lake (HL)	41.4	-106.2	Lake sediment	d18O	Excursion	Dry	4.2	4.0
Lin et al. (2022)	Literature search	Lake Tianchi	25.9	99.3	Lake sediment	Pollen	Excursion	Dry & cold	4.5	3.9
Liu and Feng (2012)	Search & Railsback et al. 2018	Multiple China			Multiple	Multiple	Various	Various		
Magny et al. (2009)	Literature search	Lakes Accesa and Maliq	41.5	15.0	Lake sediment	Stratigraphy	Excursion	Dry	4.1	3.95
Menounos et al. (2008)	Railsback et al. 2018	Redbarrel Lake	51.4	-121.2	Lake sediment	LOI	Excursion	Wet & cold	4.3	4.1
Menounos et al. (2008)	Railsback et al. 2018	Green Lake	57.7	-126.7	Lake sediment	LOI	Excursion	Wet & cold	4.4	4.0
Mischke and Zhang (2010)	Literature search	Lake Ximencuo	33.4	101.1	Lake sediment	Multiple	Mean shift	Cold		
Nakamura et al. (2016)	Search & Railsback et al. 2018	Lake Rara	29.5	82.1	Lake sediment	Mn/Ti & Mn/Fe	Excursion	Dry	4.2	3.7
Nichols (2021)	Literature search	Multiple Sibera			Multiple	Archaeological	Event stratigraphy	Assumed deterioration		
O'Donnell et al. (2020)	Literature search	Vung Tha	20.3	105.9	Terrestrial sediment	Pollen	Hiatus	Not stated		
Ocakoglu et al. (2019)	Literature search	Kureysler Valley (KS-6 and KS-4)	39.3	29.8	Lake sediment	Multiple	Excursion	Dry	4.6	4.0
Ohlendorf et al, (2014)	Search & Railsback et al. 2018	Laguna Cháltel (Laguna Azul)	-50.0	-71.1	Lake sediment	Multiple	Mean shift	Dry		
Pleskot et al. (2020)	Literature search	Lake Spore	53.8	16.7	Lake sediment	Multiple	Excursion	Cold	4.25	4.00
Prasad and Enzel (2006)	Railsback et al. 2018	Nal Sarovar	23.0	72.0	Lake sediment	C/N	Indeterminant: single point in low-resolution record	Dry		
Psomiadis et al. (2017)	Railsback et al. 2018	Skala marion Cave	40.6	24.5	Speleothem	d18O	Excursion	Dry	4	3.4
Railsback et al. (2011)	Railsback et al. 2018	Cova da Arcoia	42.6	-7.1	Speleothem	Petrology	Hiatus	Wet		
Railsback et al. (2022)	Literature search	Dante Cave	-19.4	17.9	Speleothem	d18O & d13C	Excursion	Wet	4.15	3.93
Ran and Chen (2019)	Literature search	Multiple NH low-middle latitudes			Multiple	Multiple	Various	Various		
Regattieri et al. (2014)	Railsback et al. 2018	Corchia Cave	44.0	10.2	Speleothem	Moisture index	Excursion	Dry	4.4	4.0
Renssen (2022)	Literature search	Multiple global			Multiple	Multiple	Various	Various		
Robles et al. (2022)	Literature search	Lake Sevan	40.2	45.7	Lake sediment	Multiple	Excursion	Dry	4.2	3.7
Roland et al. (2014)	Literature search	Sluggan Moss; Fallahogy Bog	54.8	-6.4	Peat	Testate amoebae	No event	No event		
Roy et al. (2022)	Literature search	Bhojbasa kame terrace (KMT)	30.8	78.9	Terrestrial sediment	Pollen	Excursion	Dry	4.4	3.8
Saravanan et al. (2019)	Literature search	Core SK291/GC15	14.7	73.2	Marine sediment	Multiple	Mean shift	Dry		
Schirrmacher et al. (2019)	Literature search	GeoB5901-2	36.2	-4.3	Marine sediment	n-Alkane	Excursion	Dry	4.4	4.3
Schirrmacher et al. (2020)	Literature search	ODP-161-976A	36.2	-4.3	Marine sediment	Leafwax dD	Excursion	Moisture source	4.2	3.7
Scroxton et al. (2023)	Literature search	Anjohikely (AK1)	-15.6	46.9	Speleothem	d18O	Hiatus	Dry		
Scroxton et al. (2023)	Literature search	Multiple tropical Indian Ocean			Speleothem	Multiple	Various	Various		
Sharifi et al. (2015)	Railsback et al. 2018	Neor Lake	36.0	38.6	Lake sediment	Ti	Excursion	Dry	4.25	3.95
Shuchun et al. (2022)	Literature search	Yanling (Core YL)	26.4	114.1	Peat	Multiple	Hiatus	Wet & warm		
Singh et al. (2022)	Literature search	Lilaur lake	28.4	79.0	Lake sediment	Grain size	Excursion	Dry	4.25	4.05
Stanley et al. (2003)	Railsback et al. 2018	Nile Delta	31.1	32.5	Marine sediment	Sr	Excursion	Dry	4.2	4.0
Staubwasser (2003)	Search & Railsback et al. 2018	Indus delta (63KA)	24.6	66.0	Marine sediment	d18O	Mean shift	Dry		
Suric et al. (2021)	Literature search	Nova Grgosova	45.8	14.7	Speleothem	Multiple	Excursion	Dry	4.3	4.1
Tan et al. (2018)	Literature search	Xianglong Cve	33.0	106.3	Speleothem	d18O & d13C	Excursion	Wet	4.39	3.8

Table S1. Results of metanalysis, including reference to paper, site metadata, and interpreted timing, duration and climate interpretation of the 4.2 ka event.

Reference	Source	Site name	Lat (°)	Lon (°)	Archive	Proxy	Change type	Climate signal	Excursion start (ka)	Excursion end (ka)
Tan et al. (2020)	Literature search	Wuya Cave	33.8	105.4	Speleothem	d18O	No event	No event		
Thompson et al. (2002)	Railsback et al. 2018	Kilimanjaro	-3.1	37.4	Glacier ice	Dust	Excursion	Dry	3.95	4.05
Tian et al. (2020)	Literature search	Huangqihai Lake	40.8	113.3	Lake sediment	Pollen	Mean shift	Dry		
Ticha et al. (2023)	Literature search	Prášilské jezero	49.1	13.4	Lake sediment	Multiple	Mean shift	Wet		
Toth and Aronson (2019)	Literature search	Contadora Island	8.6	79.0	Coral	Sr/Ca & d18O	Hiatus	Cold		
Wang et al. (2022)	Literature search	Remi Cave	29.2	109.4	Speleothem	d13C & Sr/Ca	Inflection	Wet		
Wang et al. (2022)	Literature search	Yazihai Lake	38.9	112.2	Lake sediment	Pollen	Excursion	Dry	4.34	3.88
Xiao et al. (2019)	Literature search	Daihai Lake	40.6	112.7	Lake sediment	Multiple	Excursion	Dry	4.06	3.69
Yan et al. (2014)	Literature search	Hala Lake	38.5	97.5	Lake sediment	d18O	Excursion	Dry	4.5	4.1
Zanon et al. (2019)	Literature search	Bande di Cavriana	45.4	10.6	Terrestrial sediment	Multiple	Excursion	Dry & warm	4.6	4.3
Zerathe et al. (2014)	Literature search	Southwestern Alps	43.7	7.0	Terrestrial sediment	Landslides	Event stratigraphy	Wet		
Zhang et al. (2010)	Railsback et al. 2018	Yuchuicun	33.0	117.0	Terrestrial sediment	Multiple	Mean shift	Dry		
Zhang et al. (2018)	Literature search	Shennong Cave	28.7	117.3	Speleothem	d18O & d13C	Excursion	Wet	4.2	3.9
Zhang et al. (2020)	Literature search	Hulun Lake	49.0	117.3	Lake sediment	Pollen	Mean shift	Dry & cold		
Zhu et al. (2017)	Railsback et al. 2018	Tanjialing	30.9	113.1	Lake sediment	Phytolith & charcol	Excursion	Dry	4.2	4.0
Zielhofer et al. (2017)	Railsback et al. 2018	Lake Sidi Ali	33.1	-5.0	Lake sediment	Grain size & geochemistry	Excursion	Dry	4.2	4.1
Zolitschka et al. (2021)	Literature search	Lagoa Dourada	-25.2	-50.1	Lake sediment	Multiple	Excursion	Wet	4.4	3.8

SI References

Aagaard-Sørensen, S., K. Husum, K. Werner, R. F. Spielhagen, M. Hald, and T. M. Marchitto. 2014a. “A Late Glacial-Early Holocene Multiproxy Record from the Eastern Fram Strait, Polar North Atlantic.” *Marine Geology* 355 (September): 15–26. https://doi.org/10.1016/j.margeo.2014.05.009.

———. 2014b. “A Late Glacial–Early Holocene Multiproxy Record from the Eastern Fram Strait, Polar North Atlantic.” *Marine Geology* 355 (September): 15–26. https://doi.org/10.1016/j.margeo.2014.05.009.

Abel-Schaad, Daniel, and Jos’e Antonio L’opez-S’aez. 2012. “Vegetation Changes in Relation to Fire History and Human Activities at the Pena Negra Mire (Bejar Range, Iberian Central Mountain System, Spain) During the Past 4,000 Years.” *Vegetation History and Archaeobotany* 22 (3): 199–214. https://doi.org/10.1007/s00334-012-0368-9.

Abraham, V., and P. Pokorny. 2008. “Vegetacni Zmeny v Ceskem Svycarsku Jako Dusledek Lesnickeho Hospodareni-Pokus o Kvantitativni Rekonstrukci (Vegetation Changes in Czech Switzerland as a Result of Forestry Management-an Attempt at Quantitative Reconstruction on the Basis of Pollen Analyses and Historical Sources).” *In: Bioarcheologie v Ceske Republice [Bioarchaeology in the Czech Republic] Ed. By J. Benes and P. Pokorny (Pp.443-470). Jihoceska Univerzita*.

Abrantes, F., S. Lebreiro, T. Rodrigues, I. Gil, H. Bartels-J’onsd’ottir, P. Oliveira, C. Kissel, and J. O. Grimalt. 2005. “Shallow-Marine Sediment Cores Record Climate Variability and Earthquake Activity Off Lisbon (Portugal) for the Last 2000 Years.” *Quaternary Science Reviews* 24 (23-24): 2477–94. https://doi.org/10.1016/j.quascirev.2004.04.009.

Adam, D. P. 1975. “A Late Holocene Pollen Record from Pearson’s Pond, Weeks Creek Landslide, San Francisco Peninsula, California.” *Journal of Research of the US Geological Survey*.

Adams, Jennifer K., and Sarah A. Finkelstein. 2010. “Watershed-Scale Reconstruction of Middle and Late Holocene Paleoenvironmental Changes on Melville Peninsula, Nunavut, Canada.” *Quaternary Science Reviews* 29 (17–18): 2302–14. https://doi.org/10.1016/j.quascirev.2010.05.033.

Addison, Jason A., John Barron, Bruce Finney, Jennifer Kusler, David Bukry, Linda E. Heusser, and Clark R. Alexander. 2018. “A Holocene Record of Ocean Productivity and Upwelling from the Northern California Continental Slope.” *Quaternary International* 469 (March): 96–108. https://doi.org/10.1016/j.quaint.2017.02.021.

Affolter, St’ephane, Anamaria Häuselmann, Dominik Fleitmann, R. Lawrence Edwards, Hai Cheng, and Markus Leuenberger. 2019. “Central Europe Temperature Constrained by Speleothem Fluid Inclusion Water Isotopes over the Past 14,000 Years.” *Science Advances* 5 (6). https://doi.org/10.1126/sciadv.aav3809.

Aharon, Paul, David Aldridge, and John Hellstrom. 2013. “Rainfall Variability and the Rise and Collapse of the Mississippian Chiefdoms: Evidence from a Desoto Caverns Stalagmite.” In *Climates, Landscapes, and Civilizations*, 35–42. American Geophysical Union. https://doi.org/10.1029/2012gm001203.

Aichner, Bernhard, Bernd Wünnemann, Alice Callegaro, Marcel T. J. van der Meer, Dada Yan, Yongzhan Zhang, Carlo Barbante, and Dirk Sachse. 2022. “Asynchronous Responses of Aquatic Ecosystems to Hydroclimatic Forcing on the Tibetan Plateau.” *Communications Earth &Amp; Environment* 3 (1). https://doi.org/10.1038/s43247-021-00325-1.

Ait Brahim, Yassine, Jasper A. Wassenburg, Francisco W. Cruz, Abdelfettah Sifeddine, Denis Scholz, Lhoussaine Bouchaou, Emilie P. Dassi’e, Klaus P. Jochum, R. Lawrence Edwards, and Hai Cheng. 2018. “Multi-Decadal to Centennial Hydro-Climate Variability and Linkage to Solar Forcing in the Western Mediterranean During the Last 1000 Years.” *Scientific Reports* 8 (1). https://doi.org/10.1038/s41598-018-35498-x.

Ait Brahim, Y., J. A. Wassenburg, L. Sha, F. W. Cruz, M. Deininger, A. Sifeddine, L. Bouchaou, Christoph Spötl, R. L. Edwards, and H. Cheng. 2019. “North Atlantic Ice-rafting, Ocean and Atmospheric Circulation During the Holocene: Insights from Western Mediterranean Speleothems.” *Geophysical Research Letters* 46 (13): 7614–23. https://doi.org/10.1029/2019gl082405.

Akers, Pete D., George A. Brook, L. Bruce Railsback, Fuyuan Liang, Gyles Iannone, James W. Webster, Philip P. Reeder, Hai Cheng, and R. Lawrence Edwards. 2016. “An Extended and Higher-Resolution Record of Climate and Land Use from Stalagmite MC01 from Macal Chasm, Belize, Revealing Connections Between Major Dry Events, Overall Climate Variability, and Maya Sociopolitical Changes.” *Palaeogeography, Palaeoclimatology, Palaeoecology* 459 (October): 268–88. https://doi.org/10.1016/j.palaeo.2016.07.007.

Albert, L. E., and D. G. Wyckoff. 1981. “Ferndale Bog and Natural Lake: Five Thousand Years of Environmental Change in Southeastern Oklahoma.” *Studies in Oklahoma’s Past 7. Oklahoma Archaeological Survey*.

Allen, Judy R. M., Antony J. Long, Chris J. Ottley, D. Graham Pearson, and Brian Huntley. 2007. “Holocene Climate Variability in Northernmost Europe.” *Quaternary Science Reviews* 26 (9-10): 1432–53. https://doi.org/10.1016/j.quascirev.2007.02.009.

Alley, Richard B. 2000. “The Younger Dryas Cold Interval as Viewed from Central Greenland.” *Quaternary Science Reviews* 19 (1-5): 213–26. https://doi.org/10.1016/s0277-3791(99)00062-1.

Almquist-Jacobson, Heather, and David Sanger. 1995. “Holocene Climate and Vegetation in the Milford Drainage Basin, Maine, u.s.a., and Their Implications for Human History.” *Vegetation History and Archaeobotany* 4 (4). https://doi.org/10.1007/bf00235752.

Amekawa, Shota, Kaoru Kubota, Yosuke Miyairi, Arisa Seki, Yuta Kawakubo, Saburo Sakai, P. Ajithprasad, Hideaki Maemoku, Toshiki Osada, and Yusuke Yokoyama. 2016. “Fossil Otoliths, from the Gulf of Kutch, Western India, as a Paleo-Archive for the Mid- to Late-Holocene Environment.” *Quaternary International* 397 (March): 281–88. https://doi.org/10.1016/j.quaint.2015.07.006.

Ammann, B. 1985. “Lobsigensee - Late Glacial and Holocene Environments of a Lake on the Central Swiss Plateau.” *Dissertationes Botanicae*.

AMMANN, BRIGITTA, and ANDRE F. LOTTER. 2008. “Late-Glacial Radiocarbon- and Palynostratigraphy on the Swiss Plateau.” *Boreas* 18 (2): 109–26. https://doi.org/10.1111/j.1502-3885.1989.tb00381.x.

An, Jingping, Wiebke Kirleis, and Guiyun Jin. 2021. “Understanding the Collapse of the Longshan Culture (4400-3800 BP) and the 4.2 Ka Event in the Haidai Region of China – from an Agricultural Perspective.” *Environmental Archaeology*, November, 1–15. https://doi.org/10.1080/14614103.2021.2003583.

Anderson, Lesleigh. 2011a. “Holocene Record of Precipitation Seasonality from Lake Calcite d18O in the Central Rocky Mountains, United States.” *Geology* 39 (3): 211–14. https://doi.org/10.1130/g31575.1.

———. 2011b. “Holocene Record of Precipitation Seasonality from Lake Calcite δ18O in the Central Rocky Mountains, United States.” *Geology* 39 (3): 211–14. https://doi.org/10.1130/g31575.1.

———. 2012. “Rocky Mountain Hydroclimate: Holocene Variability and the Role of Insolation, ENSO, and the North American Monsoon.” *Global and Planetary Change* 92-93 (July): 198–208. https://doi.org/10.1016/j.gloplacha.2012.05.012.

Anderson, Lesleigh, Mark B. Abbott, and Bruce P. Finney. 2001. “Holocene Climate Inferred from Oxygen Isotope Ratios in Lake Sediments, Central Brooks Range, Alaska.” *Quaternary Research* 55 (3): 313–21. https://doi.org/10.1006/qres.2001.2219.

Anderson, Lesleigh, Mark B. Abbott, Bruce P. Finney, and Stephen J. Burns. 2005. “Regional Atmospheric Circulation Change in the North Pacific During the Holocene Inferred from Lacustrine Carbonate Oxygen Isotopes, Yukon Territory, Canada.” *Quaternary Research* 64 (1): 21–35. https://doi.org/10.1016/j.yqres.2005.03.005.

———. 2007. “Late Holocene Moisture Balance Variability in the Southwest Yukon Territory, Canada.” *Quaternary Science Reviews* 26 (1-2): 130–41. https://doi.org/10.1016/j.quascirev.2006.04.011.

Anderson, N. J., A. C. Liversidge, S. McGowan, and M. D. Jones. 2012. “Lake and Catchment Response to Holocene Environmental Change: Spatial Variability Along a Climate Gradient in Southwest Greenland.” *Journal of Paleolimnology* 48 (1): 209–22. https://doi.org/10.1007/s10933-012-9616-3.

Anderson, P. M., and A. V. Lozhkin. 2002. “Late Quaternary Vegetation and Climate of Siberia and the Russian Far East (Palynological and Radiocarbon Database).” *North East Science Center*.

Anderson, R. Scott, Renata B. Jass, Jaime L. Toney, Craig D. Allen, Luz M. Cisneros-Dozal, Marcey Hess, Jeff Heikoop, and Julianna Fessenden. 2008. “Development of the Mixed Conifer Forest in Northern New Mexico and Its Relationship to Holocene Environmental Change.” *Quaternary Research* 69 (2): 263–75. https://doi.org/10.1016/j.yqres.2007.12.002.

Anderson, T. W. 1980. “Holocene Vegetation and Climatic History of Prince Edward Island, Canada.” *Canadian Journal of Earth Sciences* 17 (9): 1152–65. https://doi.org/10.1139/e80-122.

Andersson, C., F. S. R. Pausata, E. Jansen, B. Risebrobakken, and R. J. Telford. 2010. “Holocene Trends in the Foraminifer Record from the Norwegian Sea and the North Atlantic Ocean.” *Climate of the Past* 6 (2): 179–93. https://doi.org/10.5194/cp-6-179-2010.

Andersson, Sofia, Gunhild Rosqvist, Melanie j. leng, and Stefan Wastegand Maarten Blaauw. 2010. “Late Holocene Climate Change in Central Sweden Inferred from Lacustrine Stable Isotope Data.” *Journal of Quaternary Science* 25 (8): 1305–16. https://doi.org/10.1002/jqs.1415.

Andreev, A. A, P. E Tarasov, F. A Romanenko, L. D. Sulerzhitskii, and K. I. Terekhov. 1998. “Vegetation of the Western Coast of the Baidaratskaya Bay at the End of the Late Pleistocene.” *Stratigraphy and Geological Correlation*.

Andresen, Camilla S., Svante Björck, Ole Bennike, and Gerard Bond. 2004. “Holocene Climate Changes in Southern Greenland: Evidence from Lake Sediments.” *Journal of Quaternary Science* 19 (8): 783–95. https://doi.org/10.1002/jqs.886.

ANDRESEN, CAMILLA S., SVANTE BJÖRCK, MATS RUNDGREN, DANIEL J. CONLEY, and CATHERINE JESSEN. 2008. “Rapid Holocene Climate Changes in the North Atlantic: Evidence from Lake Sediments from the Faroe Islands.” *Boreas* 35 (1): 23–34. https://doi.org/10.1111/j.1502-3885.2006.tb01110.x.

Andrews, J. E., S. A. Carolin, E. N. Peckover, A. Marca, S. Al-Omari, and P. J. Rowe. 2020. “Holocene Stable Isotope Record of Insolation and Rapid Climate Change in a Stalagmite from the Zagros of Iran.” *Quaternary Science Reviews* 241 (August): 106433. https://doi.org/10.1016/j.quascirev.2020.106433.

Andrews, J. T., L. Keigwin, F. Hall, and Anne E. Jennings. 1999. “Abrupt Deglaciation Events and Holocene Palaeoceanography from High-Resolution Cores, Cartwright Saddle, Labrador Shelf, Canada.” *Journal of Quaternary Science* 14 (5): 383–97. https://doi.org/10.1002/(sici)1099-1417(199908)14:5<383::aid-jqs464>3.0.co;2-j.

Antipina, T. G., and N. K. Panova. 2016. “The Holocene Dynamics of Vegetation and Climatic Conditions on the Eastern Slope of the Subpolar Urals.” *Russian Journal of Ecology* 47 (4): 329–37. https://doi.org/10.1134/s1067413616040056.

Antipina, T. G., N. K. Panova, and O. M. Korona. 2014. “The Holocene Dynamics of Vegetation and Environmental Conditions on the Eastern Slope of the Northern Urals.” *Russian Journal of Ecology* 45 (5): 351–58. https://doi.org/10.1134/s1067413614050026.

Antonsson, Karin, and Heikki Seppä. 2007. “Holocene Temperatures in Bohuslän, Southwest Sweden: A Quantitative Reconstruction from Fossil Pollen Data.” *Boreas* 36 (4): 400–410. https://doi.org/10.1080/03009480701317421.

Arag’on-Moreno, Alejandro Antonio, Gerald A. Islebe, and Nuria Torrescano-Valle. 2012a. “A 3800-Yr, High-Resolution Record of Vegetation and Climate Change on the North Coast of the Yucatan Peninsula.” *Review of Palaeobotany and Palynology* 178 (June): 35–42. https://doi.org/10.1016/j.revpalbo.2012.04.002.

———. 2012b. “A 3800-Yr, High-Resolution Record of Vegetation and Climate Change on the North Coast of the Yucatan Peninsula.” *Review of Palaeobotany and Palynology* 178 (June): 35–42. https://doi.org/10.1016/j.revpalbo.2012.04.002.

Arenas, Fernando, Harumi Fujita, and Alberto S’anchez. 2021. “Paleoenvironmental Reconstruction in La Paz Bay, Gulf of California: Evidence from δ18O inChione Californiensis.” *The Holocene* 32 (4): 254–61. https://doi.org/10.1177/09596836211066594.

Argant, J., and A. Argant. 2000. “Mise En Evidence de l’occupation Ancienne d’un Site d’altitude: Analyse Pollinique Du Lac Du Lauzon (Drome).” *Geologie Alpine*.

Asmerom, Yemane, Victor Polyak, Stephen Burns, and Jessica Rassmussen. 2007. “Solar Forcing of Holocene Climate: New Insights from a Speleothem Record, Southwestern United States.” *Geology* 35 (1): 1. https://doi.org/10.1130/g22865a.1.

Athanasiadis, Nikolaos. 1975. “Zur Postglazialen Vegetationsentwicklung von Litochoro Katerinis Und Pertouli Trikalon (Griechenland).” *Flora* 164 (1): 99–132. https://doi.org/10.1016/s0367-2530(17)31791-7.

Auffret, G’erard, S’ebastien Zaragosi, Bernard Dennielou, Elsa Cortijo, David Van Rooij, Francis Grousset, Claude Pujol, Fr’ed’erique Eynaud, and Martin Siegert. 2002. “Terrigenous Fluxes at the Celtic Margin During the Last Glacial Cycle.” *Marine Geology* 188 (1-2): 79–108. https://doi.org/10.1016/s0025-3227(02)00276-1.

AUTHORS!, NEEDS. 2007. “NEEDS a TITLE!” *Unknown*. https://doi.org/10.1111/j.1365-2699.2006.016.

Axford, Yarrow, Shanna Losee, Jason P. Briner, Donna R. Francis, Peter G. Langdon, and Ian R. Walker. 2013. “Holocene Temperature History at the Western Greenland Ice Sheet Margin Reconstructed from Lake Sediments.” *Quaternary Science Reviews* 59 (January): 87–100. https://doi.org/10.1016/j.quascirev.2012.10.024.

Azennoud, Khalil, Abdennasser Baali, Yassine Ait Brahim, Youssra Ahouach, and Oualid Hakam. 2022. “Climate Controls on Tufa Deposition over the Last 5000 Years: A Case Study from Northwest Africa.” *Palaeogeography, Palaeoclimatology, Palaeoecology* 586 (January): 110767. https://doi.org/10.1016/j.palaeo.2021.110767.

Azevedo, Vitor, Nicol’as M. Strikis, Valdir F. Novello, Camila L. Roland, Francisco W. Cruz, Roberto V. Santos, Mathias Vuille, et al. 2021. “Paleovegetation Seesaw in Brazil Since the Late Pleistocene: A Multiproxy Study of Two Biomes.” *Earth and Planetary Science Letters* 563 (June): 116880. https://doi.org/10.1016/j.epsl.2021.116880.

Bahr, A., S. Kaboth-Bahr, A. Jaeschke, C. Chiessi, F. Cruz, L. Carvalho, J. Rethemeyer, et al. 2021. "Late Holocene Precipitation Fluctuations in South America Triggered by Variability of the North Atlantic Overturning Circulation." *Paleoceanography and Paleoclimatology* 36 (9). https://doi.org/10.1029/2021pa004223.

Baker, Jonathan L., Matthew S. Lachniet, Olga Chervyatsova, Yemane Asmerom, and Victor J. Polyak. 2017. "Holocene Warming in Western Continental Eurasia Driven by Glacial Retreat and Greenhouse Forcing." *Nature Geoscience* 10 (6): 430–35. https://doi.org/10.1038/ngeo2953.

Baker, Paul A., Sherilyn C. Fritz, Stephen J. Burns, Erik Ekdahl, and Catherine A. Rigsby. 2009. "The Nature and Origin of Decadal to Millennial Scale Climate Variability in the Southern Tropics of South America: The Holocene Record of Lago Umayo, Peru." In *Past Climate Variability in South America and Surrounding Regions*, 301–22. Springer Netherlands. https://doi.org/10.1007/978-90-481-2672-9_13.

Baker, Richard G., Louis J. Maher, Craig A. Chumbley, and Kent L. Van Zant. 1992. "Patterns of Holocene Environmental Change in the Midwestern United States." *Quaternary Research* 37 (3): 379–89. https://doi.org/10.1016/0033-5894(92)90074-s.

Bakke, Jostein, Svein Olaf Dahl, Øyvind Paasche, Reidar Løvlie, and Atle Nesje. 2005. "Glacier Fluctuations, Equilibrium-Line Altitudes and Palaeoclimate in Lyngen, Northern Norway, During the Lateglacial and Holocene." *The Holocene* 15 (4): 518–40. https://doi.org/10.1191/0959683605hl815rp.

Bakke, Jostein, Svein Olaf Dahl, Øyvind Paasche, Joachim Riis Simonsen, Bjørn Kvisvik, Kristina Bakke, and Atle Nesje. 2010. "A Complete Record of Holocene Glacier Variability at Austre Okstindbreen, Northern Norway: An Integrated Approach." *Quaternary Science Reviews* 29 (9–10): 1246–62. https://doi.org/10.1016/j.quascirev.2010.02.012.

Bakke, Jostein, øyvind Lie, Atle Nesje, Svein Olaf Dahl, and øyvind Paasche. 2005. "Utilizing Physical Sediment Variability in Glacier-Fed Lakes for Continuous Glacier Reconstructions During the Holocene, Northern Folgefonna, Western Norway." *The Holocene* 15 (2): 161–76. https://doi.org/10.1191/0959683605hl797rp.

Balascio, Nicholas L., and Raymond S. Bradley. 2012. "Evaluating Holocene Climate Change in Northern Norway Using Sediment Records from Two Contrasting Lake Systems." *Journal of Paleolimnology* 48 (1): 259–73. https://doi.org/10.1007/s10933-012-9604-7.

Banakar, Virupaxa K, Sweta Baidya, Alexander M Piotrowski, and D Shankar. 2017. "Indian Summer Monsoon Forcing on the Deglacial Polar Cold Reversals." *Journal of Earth System Science* 126 (6). https://doi.org/10.1007/s12040-017-0864-5.

Band, Shraddha, M. G. Yadava, Mahjoor Ahmad Lone, Chuan-Chou Shen, Kaushik Sree, and R. Ramesh. 2018. "High-Resolution Mid-Holocene Indian Summer Monsoon Recorded in a Stalagmite from the Kotumsar Cave, Central India." *Quaternary International* 479 (June): 19–24. https://doi.org/10.1016/j.quaint.2018.01.026.

Barber, Keith, Alastair Brown, Peter Langdon, and Paul Hughes. 2013. "Comparing and Cross-Validating Lake and Bog Palaeoclimatic Records: A Review and a New 5,000 Year Chironomid-Inferred Temperature Record from Northern England." *Journal of Paleolimnology* 49 (3): 497–512. https://doi.org/10.1007/s10933-012-9656-8.

Barbier, D. 1999. "Histoire de La Vegetation Du Nord-Mayennais de La Fin Du Weichselien a l'aube Du XXIeme Siecle." *Mise En Evidence d'un Tardiglaciaire Armoricain. Interactions Homme-Milieu. Doctoral Dissertation. Universite de Nantes*.

Barbier, D., and L. Visset. 1997. "Logne, a Peat Bog of European Ecological Interest in the Massif Armorican, Western France: Bog Development, Vegetation and Land-Use History." *Vegetation History and Archaeobotany*.

Bar-Matthews, Miryam, Avner Ayalon, Mabs Gilmour, Alan Matthews, and Chris J. Hawkesworth. 2003a. "Sea-Land Oxygen Isotopic Relationships from Planktonic Foraminifera and Speleothems in the Eastern Mediterranean Region and Their Implication for Paleorainfall During Interglacial Intervals." *Geochimica Et Cosmochimica Acta* 67 (17): 3181–99. https://doi.org/10.1016/s0016-7037(02)01031-1.

———. 2003b. "Sea–Land Oxygen Isotopic Relationships from Planktonic Foraminifera and Speleothems in the Eastern Mediterranean Region and Their Implication for Paleorainfall During Interglacial Intervals." *Geochimica Et Cosmochimica Acta* 67 (17): 3181–99. https://doi.org/10.1016/s0016-7037(02)01031-1.

Bar-Matthews, Miryam, Avner Ayalon, Aaron Kaufman, and Gerald J Wasserburg. 1999. "The Eastern Mediterranean Paleoclimate as a Reflection of Regional Events: Soreq Cave, Israel." *Earth and Planetary Science Letters* 166 (1–2): 85–95. https://doi.org/10.1016/s0012-821x(98)00275-1.

Barnosky, Cathy W. 1981. "A Record of Late Quaternary Vegetation from Davis Lake, Southern Puget Lowland, Washington." *Quaternary Research* 16 (2): 221–39. https://doi.org/10.1016/0033-5894(81)90046-6.

Baroni, C., G. Zanchetta, A. E. Fallick, and A. Longinelli. 2006. “Mollusca Stable Isotope Record of a Core from Lake Frassino, Northern Italy: Hydrological and Climatic Changes During the Last 14 Ka.” *The Holocene* 16 (6): 827–37. https://doi.org/10.1191/0959683606hol975rp.

Barr, C., J. Tibby, M. J. Leng, J. J. Tyler, A. C. G. Henderson, J. T. Overpeck, G. L. Simpson, et al. 2019a. “Holocene El Nino-Southern Oscillation Variability Reflected in Subtropical Australian Precipitation.” *Scientific Reports* 9 (1). https://doi.org/10.1038/s41598-019-38626-3.

———, et al. 2019b. “Holocene El Niño–Southern Oscillation Variability Reflected in Subtropical Australian Precipitation.” *Scientific Reports* 9 (1). https://doi.org/10.1038/s41598-019-38626-3.

Barron, John A., David Bukry, Linda E. Heusser, Jason A. Addison, and Clark R. Alexander. 2018a. “High-Resolution Climate of the Past ~7300 Years of Coastal Northernmost California: Results from Diatoms, Silicoflagellates, and Pollen.” *Quaternary International* 469 (March): 109–19. https://doi.org/10.1016/j.quaint.2016.10.039.

———. 2018b. “High-Resolution Climate of the Past ~7300 Years of Coastal Northernmost California: Results from Diatoms, Silicoflagellates, and Pollen.” *Quaternary International* 469 (March): 109–19. https://doi.org/10.1016/j.quaint.2016.10.039.

Barthelmes, Alexandra, Pim de Klerk, Anja Prager, Martin Theuerkauf, Martin Unterseher, and Hans Joosten. 2012. “Expanding NPP Analysis to Eutrophic and Forested Sites: Significance of NPPs in a Holocene Wood Peat Section (NE Germany).” *Review of Palaeobotany and Palynology* 186 (October): 22–37. https://doi.org/10.1016/j.revpalbo.2012.07.007.

BARTLEY, D. D., and A. V. MORGAN. 1990. “The Palynological Record of the Kings Pool, Stafford, England.” *New Phytologist* 116 (1): 177–94. https://doi.org/10.1111/j.1469-8137.1990.tb00522.x.

Batchelor, C. R. 2009. “Middle Holocene Environmental Changes and the History of Yew (Taxus Baccata l.) Woodland in the Lower Thames Valley.” *Doctoral Dissertation. University of London*.

Baucom, P. C, J. F Bratton, S. M Colman, J. Friddell, and A. Rochon. 2000. “Sedimentology and Core Descriptions, Marion-Dufresne Cores MD99-2204 Through - 2209, Chesapeake Bay.” *In: Initial Report on IMAGES V Cruise of the Marion-Dufresne to Chesapeake Bay June 20-22*.

Beer, Ruth, Oliver Heiri, and Willy Tinner. 2007. “Vegetation History, Fire History and Lake Development Recorded for 6300 Years by Pollen, Charcoal, Loss on Ignition and Chironomids at a Small Lake in Southern Kyrgyzstan (Alay Range, Central Asia).” *The Holocene* 17 (7): 977–85. https://doi.org/10.1177/0959683607082413.

Beer, Ruth, Franziska Kaiser, Kaspar Schmidt, Brigitta Ammann, Gabriele Carraro, Ennio Grisa, and Willy Tinner. 2008. “Vegetation History of the Walnut Forests in Kyrgyzstan (Central Asia): Natural or Anthropogenic Origin?” *Quaternary Science Reviews* 27 (5-6): 621–32. https://doi.org/10.1016/j.quascirev.2007.11.012.

Beer, Ruth, and Willy Tinner. 2008. “Four Thousand Years of Vegetation and Fire History in the Spruce Forests of Northern Kyrgyzstan (Kungey Alatau, Central Asia).” *Vegetation History and Archaeobotany* 17 (6): 629–38. https://doi.org/10.1007/s00334-008-0142-1.

Beffa, Giorgia, Tiziana Pedrotta, Daniele Colombaroli, Paul D. Henne, Jacqueline F. N. van Leeuwen, Pascal Süsstrunk, Petra Kaltenrieder, et al. 2015. “Vegetation and Fire History of Coastal North-Eastern Sardinia (Italy) Under Changing Holocene Climates and Land Use.” *Vegetation History and Archaeobotany* 25 (3): 271–89. https://doi.org/10.1007/s00334-015-0548-5.

Begeot, C. 2000. “Histoire de La Vegetation Et Du Climat Au Cours Du Tardiglaciaire Et Du Debut de l’holocene Sur Le Massif Jurassien Central a Partir de l’analyse Pollinique Et l’etude Des Macrorestes Vegetaux.” *Doctoral Dissertation. Universite de Franche-Comte*.

Behre, K. E. 2005. “Das Moor von Sehestedt.” *Landschaftsgeschichte Am Ostlichen Jadebusen. Oldenburger Forschungen NF 21. Isensee*.

Behre, K. E., and D. Kucan. 1986a. “Die Reflektion Archaeologisch Bekannter Siedlungen in Pollendiagrammen Verschiedener Entfernung. - Beispiele Aus Der Siedlungskammer Floegeln, Nordwestdeutschland.” *Anthropogenic Indicators in Pollen Diagrams*, 95 114.

———. 1986b. “Die Reflektion Archaologisch Bekannter Siedlungen in Pollendiagrammen Verschiedener Entfernung - Beispiele Aus Der Siedlungskammer Flogeln, Nordwestdeutschland .in: Anthropogenic Indicators in Pollen Diagrams Ed.” *By K.E. Behre (Pp.95-114). Balkema*.

———. 1986c. “Die Reflektion Archaologisch Bekannter Siedlungen in Pollendiagrammen Verschiedener Entfernung - Beispiele Aus Der Siedlungskammer Flogeln, Nordwestdeutschland.” *In: Anthropogenic Indicators in Pollen Diagrams Ed. By K.-E. Behre (Pp.95-114). Balkema*.

Behre, K. E., and D. Kucan. 1994. “Die Geschichte Der Kulturlandschaft Und Des Ackerbaus in Der Siedlungskammer Flogeln, Niedersachsen, Seit Der Jungsteinzeit.” *Probleme Der Kustenforschung Im Sudlichen Nordseegebiet*.

Bendle, James A. P., and Antoni Rosell-Mel’e. 2007. “High-Resolution Alkenone Sea Surface Temperature Variability on the North Icelandic Shelf: Implications for Nordic Seas Palaeoclimatic Development During the Holocene.” *The Holocene* 17 (1): 9–24. https://doi.org/10.1177/0959683607073269.

Bennett, K. D. 1983. “Devensian Late-Glacial and Flandrian Vegetational History at Hockham Mere, Norfolk, England. II. Pollen Accumulation Rates.” *New Phytologist* 95 (3): 457–87. https://doi.org/10.1111/j.1469-8137.1983.tb03512.x.

———. 1986. “Competitive Interactions Among Forest Tree Populations in Norfolk, England, During the Last 10000 Years.” *New Phytologist* 103 (3): 603–20. https://doi.org/10.1111/j.1469-8137.1986.tb02897.x.

———. 1992. “Holocene History of Forest Trees on the Bruce Peninsula, Southern Ontario.” *Canadian Journal of Botany* 70 (1): 6–18. https://doi.org/10.1139/b92-002.

Benson, Larry, Michaele Kashgarian, Robert Rye, Steve Lund, Fred Paillet, Joseph Smoot, Cynthia Kester, Scott Mensing, Dave Meko, and Susan Lindström. 2002. “Holocene Multidecadal and Multicentennial Droughts Affecting Northern California and Nevada.” *Quaternary Science Reviews* 21 (4-6): 659–82. https://doi.org/10.1016/s0277-3791(01)00048-8.

Berger, Jean-François, Jean-Paul Bravard, Louise Purdue, Anne Benoist, Michel Mouton, and Frank Braemer. 2012. “Rivers of the Hadramawt Watershed (Yemen) During the Holocene: Clues of Late Functioning.” *Quaternary International* 266 (July): 142–61. https://doi.org/10.1016/j.quaint.2011.10.037.

Berglund, B. E. 1966. “Late-Quaternary Vegetation in Eastern Blekinge, South-Eastern Sweden.” *Opera Botanica*, 3 180.

Berke, Melissa A., Thomas C. Johnson, Josef P. Werne, Stefan Schouten, and Jaap S. Sinninghe Damst’e. 2012. “A Mid-Holocene Thermal Maximum at the End of the African Humid Period.” *Earth and Planetary Science Letters* 351-352 (October): 95–104. https://doi.org/10.1016/j.epsl.2012.07.008.

Berkelhammer, M., A. Sinha, L. Stott, H. Cheng, F. S. R. Pausata, and K. Yoshimura. 2013. “An Abrupt Shift in the Indian Monsoon 4000 Years Ago.” In *Geophysical Monograph Series*, 75–88. American Geophysical Union. https://doi.org/10.1029/2012gm001207.

Bernal, Juan Pablo, Matthew Lachniet, Malcolm McCulloch, Graham Mortimer, Pedro Morales, and Edith Cienfuegos. 2011. “A Speleothem Record of Holocene Climate Variability from Southwestern Mexico.” *Quaternary Research* 75 (1): 104–13. https://doi.org/10.1016/j.yqres.2010.09.002.

Bernard, J. 1996. “Paleoenvironnement Du Pays de Retz Et Du Marais Breton-Vendeen.” *Unknown*.

Berner, K. S., N. Koç, D. Divine, F. Godtliebsen, and M. Moros. 2008. “A Decadal-scale Holocene Sea Surface Temperature Record from the Subpolar North Atlantic Constructed Using Diatoms and Statistics and Its Relation to Other Climate Parameters.” *Paleoceanography* 23 (2). https://doi.org/10.1029/2006pa001339.

Bernhardt, Christopher E., and Debra A. Willard. 2009. “Response of the Everglades Ridge and Slough Landscape to Climate Variability and 20th-Century Water Management.” *Ecological Applications* 19 (7): 1723–38. https://doi.org/10.1890/08-0779.1.

Berntsson, Annika, Gunhild C Rosqvist, and Gaute Velle. 2013. “Late-Holocene Temperature and Precipitation Changes in Vindelfjällen, Mid-Western Swedish Lapland, Inferred from Chironomid and Geochemical Data.” *The Holocene* 24 (1): 78–92. https://doi.org/10.1177/0959683613512167.

Bezrukova, Elena V., Alexey V. Belov, and Lyubov A. Orlova. 2011. “Holocene Vegetation and Climate Variability in North Pre-Baikal Region, East Siberia, Russia.” *Quaternary International* 237 (1-2): 74–82. https://doi.org/10.1016/j.quaint.2011.01.012.

Bhattacharya, Tripti, Roger Byrne, Harald Böhnel, Kurt Wogau, Ulrike Kienel, B. Lynn Ingram, and Susan Zimmerman. 2015. “Cultural Implications of Late Holocene Climate Change in the Cuenca Oriental, Mexico.” *Proceedings of the National Academy of Sciences* 112 (6): 1693–98. https://doi.org/10.1073/pnas.1405653112.

Bhushan, R., S. P. Sati, N. Rana, A. D. Shukla, A. S. Mazumdar, and N. Juyal. 2018. “High-Resolution Millennial and Centennial Scale Holocene Monsoon Variability in the Higher Central Himalayas.” *Palaeogeography, Palaeoclimatology, Palaeoecology* 489 (January): 95–104. https://doi.org/10.1016/j.palaeo.2017.09.032.

Bigot, F, G Fosse, J. P Lautridou, A. Truffreau, and G. Verron. 1976. “Prehistoire de La Normandie.” *Neolithique Et Ages Des Metaux Donnees Nouvelles. Direction Des Antiquites Prehistoriques de Basse-Normandie Caen*.

Bilt, Willem G. M., William J. Dand Johannes P. Werner, and Jostein Bakke. 2019. “Early Holocene Temperature Oscillations Exceed Amplitude of Observed and Projected Warming in Svalbard Lakes.” *Geophysical Research Letters* 46 (24): 14732–41. https://doi.org/10.1029/2019gl084384.

Bini, Monica, Giovanni Zanchetta, Aurel Perşoiu, Rosine Cartier, Albert Catal‘a, Isabel Cacho, Jonathan R. Dean, et al. 2019. “The 4.2kaBP Event in the Mediterranean Region: An Overview.” *Climate of the Past* 15 (2): 555–77. https://doi.org/10.5194/cp-15-555-2019.
Binka, K, A Ciesla, B Lacka, T Madeyska, B Marciniak, K. Szeroczynska, and K. Wieckowski. 1991. “The Development of Bledowo Lake (Central Poland) - a Palaeoecological Study.” *Studia Geologica Polonica*.
Binka, K., T. Madeyska, B. Marciniak, K. Seroczynska, and K. Wieckowski. 1988. “Bledowo Lake (Central Poland): History of Vegetation and Lake Development During the Last 12 Kyr.” *Bull. Acad. Polon. Sci.* 36: 147 158.
Bird, Broxton W., Mark B. Abbott, Donald T. Rodbell, and Mathias Vuille. 2011a. “Holocene Tropical South American Hydroclimate Revealed from a Decadally Resolved Lake Sediment d18O Record.” *Earth and Planetary Science Letters* 310 (3-4): 192–202. https://doi.org/10.1016/j.epsl.2011.08.040.
———. 2011b. “Holocene Tropical South American Hydroclimate Revealed from a Decadally Resolved Lake Sediment δ18O Record.” *Earth and Planetary Science Letters* 310 (3–4): 192–202. https://doi.org/10.1016/j.epsl.2011.08.040.
Bird, Broxton W., Pratigya J. Polisar, Yanbin Lei, Lonnie G. Thompson, Tandong Yao, Bruce P. Finney, Daniel J. Bain, David P. Pompeani, and Byron A. Steinman. 2014. “A Tibetan Lake Sediment Record of Holocene Indian Summer Monsoon Variability.” *Earth and Planetary Science Letters* 399 (August): 92–102. https://doi.org/10.1016/j.epsl.2014.05.017.
Bird, Broxton W, Owen Rudloff, Jaime Escobar, William P Gilhooly, Alex Correa-Metrio, Maria V’elez, and Pratigya J Polissar. 2017. “Paleoclimate Support for a Persistent Dry Island Effect in the Colombian Andes During the Last 4700 Years.” *The Holocene* 28 (2): 217–28. https://doi.org/10.1177/0959683617721324.
Birks, H. J. B. 2006. “Estimating the Amount of Compositional Change in Late-Quaternary Pollen-Stratigraphical Data.” *Vegetation History and Archaeobotany* 16 (2-3): 197–202. https://doi.org/10.1007/s00334-006-0079-1.
Birks, Hilary H., Vivienne J. Jones, Stephen J. Brooks, H. John B. Birks, Richard J. Telford, Stephen Juggins, and Sylvia M. Peglar. 2012. “From Cold to Cool in Northernmost Norway: Lateglacial and Early Holocene Multi-Proxy Environmental and Climate Reconstructions from Jansvatnet, Hammerfest.” *Quaternary Science Reviews* 33 (February): 100–120. https://doi.org/10.1016/j.quascirev.2011.11.013.
Bisculm, Martina, Daniele Colombaroli, Elisa Vescovi, Jacqueline F. N. van Leeuwen, Paul D. Henne, Julian Rothen, Giovanni Procacci, Salvatore Pasta, Tommaso La Mantia, and Willy Tinner. 2012. “Holocene Vegetation and Fire Dynamics in the Supra-Mediterranean Belt of the Nebrodi Mountains (Sicily, Italy).” *Journal of Quaternary Science* 27 (7): 687–98. https://doi.org/10.1002/jqs.2551.
Björkman, Leif, Angelica Feurdean, and Barbara Wohlfarth. 2003. “Late-Glacial and Holocene Forest Dynamics at Steregoiu in the Gutaiului Mountains, Northwest Romania.” *Review of Palaeobotany and Palynology* 124 (1-2): 79–111. https://doi.org/10.1016/s0034-6667(02)00249-x.
Blaauw, Maarten, Bas van Geel, Iris Kristen, Birgit Plessen, Anna Lyaruu, Daniel R. Engstrom, Johannes van der Plicht, and Dirk Verschuren. 2011. “High-Resolution 14C Dating of a 25,000-Year Lake-Sediment Record from Equatorial East Africa.” *Quaternary Science Reviews* 30 (21-22): 3043–59. https://doi.org/10.1016/j.quascirev.2011.07.014.
Blanchet, C’ecile L., Martin Frank, and Stefan Schouten. 2014. “Asynchronous Changes in Vegetation, Runoff and Erosion in the Nile River Watershed During the Holocene.” Edited by ChengLi. *PLOS One* 9 (12): e115958. https://doi.org/10.1371/journal.pone.0115958.
Blanchet, C’ecile L., Rik Tjallingii, Martin Frank, Janne Lorenzen, Anja Reitz, Kevin Brown, Tomas Feseker, and Warner Brückmann. 2013. “High- and Low-Latitude Forcing of the Nile River Regime During the Holocene Inferred from Laminated Sediments of the Nile Deep-Sea Fan.” *Earth and Planetary Science Letters* 364 (February): 98–110. https://doi.org/10.1016/j.epsl.2013.01.009.
Blanchet, C’ecile L., Rik Tjallingii, Anja M. Schleicher, Stefan Schouten, Martin Frank, and Achim Brauer. 2021. “Deoxygenation Dynamics on the Western Nile Deep-Sea Fan During Sapropel S1 from Seasonal to Millennial Timescales.” *Climate of the Past* 17 (3): 1025–50. https://doi.org/10.5194/cp-17-1025-2021.
Bliedtner, Marcel, Paul Strobel, Julian Struck, Gary Salazar, Sönke Szidat, Norbert Nowaczyk, Enkhtuya Bazarradnaa, et al. 2022. “Holocene Temperature Variations in Semi-Arid Central Mongolia—a Chronological and Sedimentological Perspective from a 7400-Year Lake Sediment Record from the Khangai Mountains.” *Frontiers in Earth Science* 10 (May). https://doi.org/10.3389/feart.2022.910782.
Blissett, S. D. 2010. “A Late Holocene History of Vegetation, Fire, and Climate from a Desert Wetland in Southwest North America.” *Master’s Thesis. University of Utah.*

Blyakharchuk, T. A. 1980. “Sporovo-Pyl’zevaya Kharakteristika Bolot Verhneketskogo Raiona [Spore and Pollen Characteristics of Peatlands of the Verhneketskii Region During the Holocene].” *In: Molodye Uchoenye Narodnomy Khozyaistvy Ekonomicheskaya Ozenka Landshafta Tomskoi Oblasti [Abstracts of the Conference Scientist for Peoples Economy: Economical Evaluation of the Tomsk Region (Pp.24-25). Tomsk University Press*.

Bohncke, S. J. P. 1991. “Palaeohydrological Changes in the Netherlands During the Last 13000 Years.” *Doctoral Dissertation. Vrije University*.

Bonnefille, R. 2000. “Pollen-Inferred Precipitation Time-Series from Equatorial Mountains, Africa, the Last 40 Kyr BP.” *Global and Planetary Change* 26 (1-3): 25–50. https://doi.org/10.1016/s0921-8181(00)00032-1.

Booth, Robert K., Simon Brewer, Maarten Blaauw, Thomas A. Minckley, and Stephen T. Jackson. 2012a. “Decomposing the Mid-holocene Tsuga Decline in Eastern North America.” *Ecology* 93 (8): 1841–52. https://doi.org/10.1890/11-2062.1.

———. 2012b. “Decomposing the Mid-Holocene<i>tsuga</i>decline in Eastern North America.” *Ecology* 93 (8): 1841–52. https://doi.org/10.1890/11-2062.1.

Booth, Robert K., and Stephen T. Jackson. 2003. “A High-Resolution Record of Late-Holocene Moisture Variability from a Michigan Raised Bog, USA.” *The Holocene* 13 (6): 863–76. https://doi.org/10.1191/0959683603hl669rp.

Booth, Robert K., Stephen T. Jackson, Steven L. Forman, John E. Kutzbach, E. A. Bettis, Joseph Kreigs, and David K. Wright. 2005. “A Severe Centennial-Scale Drought in Midcontinental North America 4200 Years Ago and Apparent Global Linkages.” *The Holocene* 15 (3): 321–28. https://doi.org/10.1191/0959683605hl825ft.

Booth, Robert K., Stephen T. Jackson, and Catherine E. D. Gray. 2004. “Paleoecology and High-Resolution Paleohydrology of a Kettle Peatland in Upper Michigan.” *Quaternary Research* 61 (1): 1–13. https://doi.org/10.1016/j.yqres.2003.07.013.

Booth, Robert K., Stephen T. Jackson, Valerie A. Sousa, Maura E. Sullivan, Thomas A. Minckley, and Michael J. Clifford. 2012. “Multi-Decadal Drought and Amplified Moisture Variability Drove Rapid Forest Community Change in a Humid Region.” *Ecology* 93 (2): 219–26. https://doi.org/10.1890/11-1068.1.

Bordon, Amandine, Odile Peyron, Anne-Marie L’ezine, Simon Brewer, and Eric Fouache. 2009. “Pollen-Inferred Late-Glacial and Holocene Climate in Southern Balkans (Lake Maliq).” *Quaternary International* 200 (1-2): 19–30. https://doi.org/10.1016/j.quaint.2008.05.014.

Borgmark, Anders, and Stefan Wasteg. 2008. “Regional and Local Patterns of Peat Humification in Three Raised Peat Bogs in Värmland, South-Central Sweden.” *GFF* 130 (3): 161–76. https://doi.org/10.1080/11035890809453231.

Börner, Nicole, Klaus Peter Jochum, Marleen Stuhr, Michelle Abstein, Birgit Plessen, Peter Frenzel, Junbo Wang, Liping Zhu, and Antje Schwalb. 2022. “Late Quaternary Changes in Moisture Availability and Weathering Intensity on the Central Tibetan Plateau Indicated by Chemical Signatures of Ostracod Shells.” *Frontiers in Earth Science* 10 (July). https://doi.org/10.3389/feart.2022.826143.

Bortenschlager, S. 1970. “Waldgrenz- Und Klimaschwankungen Im Pollenanalytischen Bild Des Gurgler Rotmooses.” *Mitteilungen Der Ostalpin-Dinarischen Gesellschaft Fur Vegetationskunde*.

———. 1984. “Beitrage Zur Vegetationsgeschichte Tirols i.” *Inneres Otztal Und Unteres Inntal (Contributions to the Vegetational History of Tyrol l. Inner Oetz-Valley and Lower Inn-Valley). Berichte Des Naturwissenschaftlich-Medizinischen Vereins in Innsbruck*.

Bottema, S. 1974. “Late Quaternary Vegetation History of Northwestern Greece.” *Doctoral Dissertation. University of Groningen*.

Bova, Samantha C., Timothy Herbert, Yair Rosenthal, Julie Kalansky, Mark Altabet, Caitlin Chazen, Angel Mojarro, and Jana Zech. 2015. “Links Between Eastern Equatorial Pacific Stratification and Atmospheric CO2 Rise During the Last Deglaciation.” *Paleoceanography* 30 (11): 1407–24. https://doi.org/10.1002/2015pa002816.

Bozilova, Elissaveta, and Hans-JNANArgen Beu. 1992. “On the Holocene History of Vegetation in SE Bulgaria Lake Arkutino, Ropotamo Region.” *Vegetation History and Archaeobotany* 1 (1). https://doi.org/10.1007/bf00190698.

Bozilova, Elissaveta, and Hans-Jorgen Beug. 1994. “Studies on the Vegetation History of Lake Varna Region, Northern Black Sea Coastal Area of Bulgaria.” *Vegetation History and Archaeobotany* 3 (3). https://doi.org/10.1007/bf00202022.

Bozilova, E., and A. G. Smith. 1979. “Palynology of Lake Sucho Ezero from South Rila Mountain (Bulgaria).” *Phytologia*.

Brahim, Y. Ait, J. A. Wassenburg, L. Sha, F. W. Cruz, M. Deininger, A. Sifeddine, L. Bouchaou, Christoph Spötl, R. L. Edwards, and H. Cheng. 2019. "North Atlantic Ice-Rafting, Ocean and Atmospheric Circulation During the Holocene: Insights from Western Mediterranean Speleothems." *Geophysical Research Letters* 46 (13): 7614–23. https://doi.org/10.1029/2019gl082405.

Brande, A. 1996. "Type Region d-s, Berlin." *In: Palaeoecological Events During the Last 15 000 Years: Regional Syntheses of Palaeoecological Studies of Lakes and Mires in Europe Ed. By B.E. Berglund*.

Breitenbach, Sebastian F. M., Franziska A. Lechleitner, Hanno Meyer, Gregory Diengdoh, David Mattey, and Norbert Marwan. 2015a. "Cave Ventilation and Rainfall Signals in Dripwater in a Monsoonal Setting - a Monitoring Study from NE India." *Chemical Geology* 402 (May): 111–24. https://doi.org/10.1016/j.chemgeo.2015.03.011.

———. 2015b. "Cave Ventilation and Rainfall Signals in Dripwater in a Monsoonal Setting – a Monitoring Study from NE India." *Chemical Geology* 402 (May): 111–24. https://doi.org/10.1016/j.chemgeo.2015.03.011.

Bringu'e, Manuel, and Andr'e Rochon. 2012. "Late Holocene Paleoceanography and Climate Variability over the Mackenzie Slope (Beaufort Sea, Canadian Arctic)." *Marine Geology* 291–294 (January): 83–96. https://doi.org/10.1016/j.margeo.2011.11.004.

Brisset, Elodie, Fr'ed'eric Guiter, C'ecile Miramont, Claire Delhon, Fabien Arnaud, Jean-Robert Disnar, J'erôme Poulenard, et al. 2012. "Approche Multidisciplinaire d'une Séquence Lacustre Holocène Dans Les Alpes Du Sud Au Lac Petit (Mercantour, Alt. 2 200 m, France) : Histoire d'un Géosystème Dégradé." *Quaternaire*, no. vol. 23/4 (December). https://doi.org/10.4000/quaternaire.6390.

Brookes, I. A., D. B. Scott, and J. H. McAndrews. 1985. "Postglacial Relative Sea-Level Change, Port Au Port Area, West Newfoundland." *Canadian Journal of Earth Sciences* 22 (7): 1039–47. https://doi.org/10.1139/e85-107.

Brown, A. D. 2006. "Late-Holocene Palaeoclimates: Cross-Validation of Multiple Proxies from Lake and Bog Archives in Northern England." *Doctoral Dissertation. University of Southampton*.

Brown, A. P. 1972. "Late-Weichselian and Flandrian Vegetation of Bodmin Moor, Cornwall." *Doctoral Dissertation. University of Cambridge*.

Brown, K. J., J. S. Clark, E. C. Grimm, J. J. Donovan, P. G. Mueller, B. C. S. Hansen, and I. Stefanova. 2005. "Fire Cycles in North American Interior Grasslands and Their Relation to Prairie Drought." *Proceedings of the National Academy of Sciences* 102 (25): 8865–70. https://doi.org/10.1073/pnas.0503621102.

Brubaker, Linda B., Harriet L. Garfinkel, and Mary E. Edwards. 1983. "A Late Wisconsin and Holocene Vegetation History from the Central Brooks Range: Implications for Alaskan Palaeoecology." *Quaternary Research* 20 (2): 194–214. https://doi.org/10.1016/0033-5894(83)90077-7.

Brugiapaglia, E., and J. L. De Beaulieu. 1995. "Etude de La Dynamique Vegetale Tardiglaciaire Et Holocene En Italie Centrale: Le Marais de Colfiorito (Ombrie)." *Comptes Rendus de l'Academie Des Sciences. Serie 2. Sciences de La Terre Et Des Planetes*.

Budsky, Alexander, Denis Scholz, Jasper A Wassenburg, Regina Mertz-Kraus, Christoph Spötl, Dana FC Riechelmann, Luis Gibert, Klaus Peter Jochum, and Meinrat O Andreae. 2019a. "Speleothem d13C Record Suggests Enhanced Spring/Summer Drought in South-Eastern Spain Between 9.7 and 7.8 Ka - a Circum-Western Mediterranean Anomaly?" *The Holocene* 29 (7): 1113–33. https://doi.org/10.1177/0959683619838021.

———. 2019b. "Speleothem δ13C Record Suggests Enhanced Spring/Summer Drought in South-Eastern Spain Between 9.7 and 7.8 Ka – a Circum-Western Mediterranean Anomaly?" *The Holocene* 29 (7): 1113–33. https://doi.org/10.1177/0959683619838021.

Bunting, M Jane, and Barry G Warner. 1999. "Late Quaternary Vegetation Dynamics and Hydroseral Development in a Shrub Swamp in Southern Ontario, Canada." *Canadian Journal of Earth Sciences* 36 (10): 1603–16. https://doi.org/10.1139/e99-068.

Burdanowitz, Nicole, Lydie Dupont, Matthias Zabel, and Enno Schefuß. 2018. "Holocene Hydrologic and Vegetation Developments in the Orange River Catchment (South Africa) and Their Controls." *The Holocene* 28 (8): 1288–1300. https://doi.org/10.1177/0959683618771484.

Burdanowitz, Nicole, Birgit Gaye, Lea Hilbig, Niko Lahajnar, Andreas Lückge, Tim Rixen, and Kay-Christian Emeis. 2019. "Holocene Monsoon and Sea Level-Related Changes of Sedimentation in the Northeastern Arabian Sea." *Deep Sea Research Part II: Topical Studies in Oceanography* 166 (August): 6–18. https://doi.org/10.1016/j.dsr2.2019.03.003.

Burdanowitz, Nicole, Tim Rixen, Birgit Gaye, and Kay-Christian Emeis. 2021a. "Signals of Holocene Climate Transition Amplified by Anthropogenic Land-Use Changes in the Westerly-Indian Monsoon Realm." *Climate of the Past* 17 (4): 1735–49. https://doi.org/10.5194/cp-17-1735-2021.

———. 2021b. “Signals of Holocene Climate Transition Amplified by Anthropogenic Land-Use Changes in the Westerly–Indian Monsoon Realm.” *Climate of the Past* 17 (4): 1735–49. https://doi.org/10.5194/cp-17-1735-2021.
Burjachs, F, R Perez-Obiol, J. M. Roure, and R. Julia. 1994. “Dinamica de La Vegetacion Durante El Holoceno En La Isla de Mallorca.” *Trabajos de Palinologia Basica y Aplicada*.
Burns, Stephen J., Lisa Kanner Welsh, Nick Scroxton, Hai Cheng, and R. Lawrence Edwards. 2019. “Millennial and Orbital Scale Variability of the South American Monsoon During the Penultimate Glacial Period.” *Scientific Reports* 9 (1). https://doi.org/10.1038/s41598-018-37854-3.
Bustamante, M. G., F. W. Cruz, M. Vuille, J. Apa’estegui, N. Strikis, G. Panizo, F. V. Novello, et al. 2016a. “Holocene Changes in Monsoon Precipitation in the Andes of NE Peru Based on d18O Speleothem Records.” *Quaternary Science Reviews* 146 (August): 274–87. https://doi.org/10.1016/j.quascirev.2016.05.023.
———, et al. 2016b. “Holocene Changes in Monsoon Precipitation in the Andes of NE Peru Based on δ18O Speleothem Records.” *Quaternary Science Reviews* 146 (August): 274–87. https://doi.org/10.1016/j.quascirev.2016.05.023.
Cacho, Isabel, Joan O. Grimalt, Miquel Canals, Laura Sbaffi, Nick J. Shackleton, Joachim Schönfeld, and Rainer Zahn. 2001. “Variability of the Western Mediterranean Sea Surface Temperature During the Last 25,000 Years and Its Connection with the Northern Hemisphere Climatic Changes.” *Paleoceanography* 16 (1): 40–52. https://doi.org/10.1029/2000pa000502.
Cacho, Isabel, Joan O Grimalt, Carles Pelejero, Miquel Canals, Francisco J. Sierro, Jos’e Abel Flores, and Nick Shackleton. 1999. “Dansgaard-Oeschger and Heinrich Event Imprints in Alboran Sea Paleotemperatures.” *Paleoceanography* 14 (6): 698–705. https://doi.org/10.1029/1999pa900044.
Cai, Shanshan, and Zicheng Yu. 2011. “Response of a Warm Temperate Peatland to Holocene Climate Change in Northeastern Pennsylvania.” *Quaternary Research* 75 (3): 531–40. https://doi.org/10.1016/j.yqres.2011.01.003.
Cai, Yanjun, John C. H. Chiang, Sebastian F. M. Breitenbach, Liangcheng Tan, Hai Cheng, R. Lawrence Edwards, and Zhisheng An. 2017. “Holocene Moisture Changes in Western China, Central Asia, Inferred from Stalagmites.” *Quaternary Science Reviews* 158 (February): 15–28. https://doi.org/10.1016/j.quascirev.2016.12.014.
Cai, Yanjun, Inez Y. Fung, R. Lawrence Edwards, Zhisheng An, Hai Cheng, Jung-Eun Lee, Liangcheng Tan, et al. 2015. “Variability of Stalagmite-Inferred Indian Monsoon Precipitation over the Past 252,000 y.” *Proceedings of the National Academy of Sciences* 112 (10): 2954–59. https://doi.org/10.1073/pnas.1424035112.
Cai, Yanjun, Liangcheng Tan, Hai Cheng, Zhisheng An, R. Lawrence Edwards, Megan J. Kelly, Xinggong Kong, and Xianfeng Wang. 2010. “The Variation of Summer Monsoon Precipitation in Central China Since the Last Deglaciation.” *Earth and Planetary Science Letters* 291 (1-4): 21–31. https://doi.org/10.1016/j.epsl.2009.12.039.
Cai, Yanjun, Meiliang Zhang, Zicheng Peng, Yushi Lin, Zhisheng An, Zhaofeng Zhang, and Yunning Cao. 2001a. “The d18O Variation of a Stalagmite from Qixing Cave, Guizhou Province and Indicated Climate Change During the Holocene.” *Chinese Science Bulletin* 46 (22): 1904–8. https://doi.org/10.1007/bf02901169.
———. 2001b. “The δ18O Variation of a Stalagmite from Qixing Cave, Guizhou Province and Indicated Climate Change During the Holocene.” *Chinese Science Bulletin* 46 (22): 1904–8. https://doi.org/10.1007/bf02901169.
Calcote, Randy. 2003. “Mid-Holocene Climate and the Hemlock Decline: The Range Limit of Tsuga Canadensis in the Western Great Lakes Region, USA.” *The Holocene* 13 (2): 215–24. https://doi.org/10.1191/0959683603hl608rp.
Calder, W. J. 2016. “Interactions Among Climate Change, Wildfire, and Vegetation Shaping Landscapes for the Last 2000 Years.” *Doctoral Dissertation. University of Wyoming*.
Calvo, Eva, Joan Grimalt, and Eystein Jansen. 2002. “High Resolution U37K Sea Surface Temperature Reconstruction in the Norwegian Sea During the Holocene.” *Quaternary Science Reviews* 21 (12-13): 1385–94. https://doi.org/10.1016/s0277-3791(01)00096-8.
Came, Rosemarie E., Delia W. Oppo, and Jerry F. McManus. 2007. “Amplitude and Timing of Temperature and Salinity Variability in the Subpolar North Atlantic over the Past 10 k.y.” *Geology* 35 (4): 315. https://doi.org/10.1130/g23455a.1.
Camill, Philip, Charles E. Umbanhowar, Christoph Geiss, William O. Hobbs, Mark B. Edlund, Avery Cook Shinneman, Jeffrey A. Dorale, and Jason Lynch. 2012. “Holocene Climate Change and Landscape

Development from a Low-Arctic Tundra Lake in the Western Hudson Bay Region of Manitoba, Canada." *Journal of Paleolimnology* 48 (1): 175–92. https://doi.org/10.1007/s10933-012-9619-0.
Camill, Philip, Charles E. Umbanhowar, Rebecca Teed, Christoph E. Geiss, Jessica Aldinger, Leah Dvorak, Jon Kenning, Jacob Limmer, and Kristina Walkup. 2003. "Late-Glacial and Holocene Climatic Effects on Fire and Vegetation Dynamics at the Prairie-Forest Ecotone in South-Central Minnesota." *Journal of Ecology* 91 (5): 822–36. https://doi.org/10.1046/j.1365-2745.2003.00812.x.
Campos, Mar., Cristiano M. Chiessi, Matthias Prange, Stefan Mulitza, Henning Kuhnert, Andr'e Paul, Igor M. Venancio, Ana Luiza S. Albuquerque, Francisco W. Cruz, and Andr'e Bahr. 2019. "A New Mechanism for Millennial Scale Positive Precipitation Anomalies over Tropical South America." *Quaternary Science Reviews* 225 (December): 105990. https://doi.org/10.1016/j.quascirev.2019.105990.
Camuera, Jon, Mar. Ramos-Rom'an, Gonzalo Jim'enez-Moreno, Antonio Garc-Alix, Liisa Ilvonen, Leena Ruha, Graciela Gil-Romera, Pen'elope Gonz'alez-Samp'eriz, and Heikki Seppä. 2022. "Past 200 Kyr Hydroclimate Variability in the Western Mediterranean and Its Connection to the African Humid Periods." *Scientific Reports* 12 (1). https://doi.org/10.1038/s41598-022-12047-1.
Caniup'an, Magaly, Frank Lamy, Carina B. Lange, J'erôme Kaiser, Rolf Kilian, Helge W. Arz, Tania Le'on, et al. 2014. "Holocene Sea-Surface Temperature Variability in the Chilean Fjord Region." *Quaternary Research* 82 (2): 342–53. https://doi.org/10.1016/j.yqres.2014.07.009.
Carcaud, N, M Garcin, L Visset, J. Musch, and J. Burnouf. 2002. "Nouvelle Lecture de l'evolution Des Paysages Fluviaux a l'holocene Dans Le Bassin de La Loire Moyenne." *Les Fleuves Ont Une Histoire*.
Carolin, Stacy A. 2019. "Precise Timing of Abrupt Increase in Dust Activity in the Middle East Coincident with 4.2 Ka Social Change." *Proceedings of the National Academy of Sciences of the United States of America* 116: 67–72. https://doi.org/10.1073/pnas.180810311.
Carolin, Stacy A., Kim M. Cobb, Jess F. Adkins, Brian Clark, Jessica L. Conroy, Syria Lejau, Jenny Malang, and Andrew A. Tuen. 2013. "Varied Response of Western Pacific Hydrology to Climate Forcings over the Last Glacial Period." *Science* 340 (6140): 1564–66. https://doi.org/10.1126/science.1233797.
Carolin, Stacy A., Richard T. Walker, Christopher C. Day, Vasile Ersek, R. Alastair Sloan, Michael W. Dee, Morteza Talebian, and Gideon M. Henderson. 2018. "Precise Timing of Abrupt Increase in Dust Activity in the Middle East Coincident with 4.2 Ka Social Change." *Proceedings of the National Academy of Sciences* 116 (1): 67–72. https://doi.org/10.1073/pnas.1808103115.
Carrillo-Bastos, Alicia, Gerald A. Islebe, and Nuria Torrescano-Valle. 2013. "3800 Years of Quantitative Precipitation Reconstruction from the Northwest Yucatan Peninsula." Edited by Gil Bohrer. *PLOS One* 8 (12): e84333. https://doi.org/10.1371/journal.pone.0084333.
Carter, Vachel A., Andrea Brunelle, Thomas A. Minckley, John D. Shaw, R. Justin DeRose, and Simon Brewer. 2017a. "Climate Variability and Fire Effects on Quaking Aspen in the Central Rocky Mountains, <Scp>USA</Scp>." *Journal of Biogeography* 44 (6): 1280–93. https://doi.org/10.1111/jbi.12932.
———. 2017b. "Climate Variability and Fire Effects on Quaking Aspen in the Central Rocky Mountains, USA." *Journal of Biogeography* 44 (6): 1280–93. https://doi.org/10.1111/jbi.12932.
Carter, Vachel A., Alice Moravcov'a, Richard C. Chiverrell, Jennifer L. Clear, Walter Finsinger, Dagmar Dreslerov'a, Karen Halsall, and Petr Kuneš. 2018. "Holocene-Scale Fire Dynamics of Central European Temperate Spruce-Beech Forests." *Quaternary Science Reviews* 191 (July): 15–30. https://doi.org/10.1016/j.quascirev.2018.05.001.
Caseldine, Chris, Peter Langdon, and Naomi Holmes. 2006. "Early Holocene Climate Variability and the Timing and Extent of the Holocene Thermal Maximum (HTM) in Northern Iceland." *Quaternary Science Reviews* 25 (17-18): 2314–31. https://doi.org/10.1016/j.quascirev.2006.02.003.
Castañeda, Isla S., Stefan Schouten, Jürgen Pätzold, Friedrich Lucassen, Simone Kasemann, Holger Kuhlmann, and Enno Schefuß. 2016. "Hydroclimate Variability in the Nile River Basin During the Past 28,000 Years." *Earth and Planetary Science Letters* 438 (March): 47–56. https://doi.org/10.1016/j.epsl.2015.12.014.
Castañeda, Isla S., L. Micaela Smith, Gr'eta Björk Kristj'ansd'ottir, and John T. Andrews. 2004. "Temporal Changes in Holocened18O Records from the Northwest and Central North Iceland Shelf." *Journal of Quaternary Science* 19 (4): 321–34. https://doi.org/10.1002/jqs.841.
Chakraborty, Krish, Sarah A. Finkelstein, Joseph R. Desloges, and Nicole A. Chow. 2010. "Holocene Paleoenvironmental Changes Inferred from Diatom Assemblages in Sediments of Kusawa Lake, Yukon Territory, Canada." *Quaternary Research* 74 (1): 15–22. https://doi.org/10.1016/j.yqres.2010.04.011.
Chang, Fengming, Tiegang Li, Zhifang Xiong, and Zhaokai Xu. 2015. "Evidence for Sea Level and Monsoonally Driven Variations in Terrigenous Input to the Northern East China Sea During the Last 24.3ka." *Paleoceanography* 30 (6): 642–58. https://doi.org/10.1002/2014pa002733.

Chase, B. M., M. E. Meadows, L. Scott, D. S. G. Thomas, E. Marais, J. Sealy, and P. J. Reimer. 2009. "A Record of Rapid Holocene Climate Change Preserved in Hyrax Middens from Southwestern Africa." *Geology* 37 (8): 703–6. https://doi.org/10.1130/g30053a.1.

Chawchai, Sakonvan, Liangcheng Tan, Ludvig Löwemark, Hao-Cheng Wang, Tsai-Luen Yu, Yun-Chuan Chung, Horng-Sheng Mii, et al. 2021. "Hydroclimate Variability of Central Indo-Pacific Region During the Holocene." *Quaternary Science Reviews* 253 (February): 106779. https://doi.org/10.1016/j.quascirev.2020.106779.

Chen, Chen-Tung A, Hsin-Chi Lan, Jiann-Yuh Lou, and Yan-Cheng Chen. 2003. "The Dry Holocene Megathermal in Inner Mongolia." *Palaeogeography, Palaeoclimatology, Palaeoecology* 193 (2): 181–200. https://doi.org/10.1016/s0031-0182(03)00225-6.

Chen, Fa-Hu, Bo Cheng, Yan Zhao, Yan Zhu, and David B. Madsen. 2006. "Holocene Environmental Change Inferred from a High-Resolution Pollen Record, Lake Zhuyeze, Arid China." *The Holocene* 16 (5): 675–84. https://doi.org/10.1191/0959683606hl951rp.

Chen, Fahu, Zicheng Yu, Meilin Yang, Emi Ito, Sumin Wang, David B. Madsen, Xiaozhong Huang, et al. 2008. "Holocene Moisture Evolution in Arid Central Asia and Its Out-of-Phase Relationship with Asian Monsoon History." *Quaternary Science Reviews* 27 (3-4): 351–64. https://doi.org/10.1016/j.quascirev.2007.10.017.

Chen, Fahu, Jifeng Zhang, Jianbao Liu, Xianyong Cao, Juzhi Hou, Liping Zhu, Xiangke Xu, et al. 2020. "Climate Change, Vegetation History, and Landscape Responses on the Tibetan Plateau During the Holocene: A Comprehensive Review." *Quaternary Science Reviews* 243 (September): 106444. https://doi.org/10.1016/j.quascirev.2020.106444.

Chen, Hao, Liping Zhu, Junbo Wang, Jianting Ju, Qingfeng Ma, and Teng Xu. 2021. "Paleoclimate Changes over the Past 13,000 Years Recorded by Chibuzhang Co Sediments in the Source Region of the Yangtze River, China." *Palaeogeography, Palaeoclimatology, Palaeoecology* 573 (July): 110433. https://doi.org/10.1016/j.palaeo.2021.110433.

Chen, S. H. 1988. "Neue Untersuchungen Uber Die Spat-Und Postglaziale Vegetationsgeschichte Im Gebiet Zwischen Harz Und Leine (BRD)." *Flora*.

Chen, Sang, Sharon S. Hoffmann, David C. Lund, Kim M. Cobb, Julien Emile-Geay, and Jess F. Adkins. 2016. "A High-Resolution Speleothem Record of Western Equatorial Pacific Rainfall: Implications for Holocene ENSO Evolution." *Earth and Planetary Science Letters* 442 (May): 61–71. https://doi.org/10.1016/j.epsl.2016.02.050.

Cheng, Hai, R. Lawrence Edwards, Ashish Sinha, Christoph Spötl, Liang Yi, Shitao Chen, Megan Kelly, et al. 2016. "The Asian Monsoon over the Past 640,000 Years and Ice Age Terminations." *Nature* 534 (7609): 640–46. https://doi.org/10.1038/nature18591.

Cheng, Hai, Dominik Fleitmann, R. Lawrence Edwards, Xianfeng Wang, Francisco W. Cruz, Augusto S. Auler, Augusto Mangini, et al. 2009a. "Timing and Structure of the 8.2 Kyr b.p. Event Inferred from d18O Records of Stalagmites from China, Oman, and Brazil." *Geology* 37 (11): 1007–10. https://doi.org/10.1130/g30126a.1.

———, et al. 2009b. "Timing and Structure of the 8.2 Kyr b.p. Event Inferred from δ18O Records of Stalagmites from China, Oman, and Brazil." *Geology* 37 (11): 1007–10. https://doi.org/10.1130/g30126a.1.

Cheng, Hai, Ashish Sinha, Francisco W. Cruz, Xianfeng Wang, R. Lawrence Edwards, Fernando M. d'Horta, Camila C. Ribas, Mathias Vuille, Lowell D. Stott, and Augusto S. Auler. 2013. "Climate Change Patterns in Amazonia and Biodiversity." *Nature Communications* 4 (1). https://doi.org/10.1038/ncomms2415.

Cheng, Hai, Christoph Spötl, Sebastian F. M. Breitenbach, Ashish Sinha, Jasper A. Wassenburg, Klaus Peter Jochum, Denis Scholz, et al. 2016. "Climate Variations of Central Asia on Orbital to Millennial Timescales." *Scientific Reports* 6 (1). https://doi.org/10.1038/srep36975.

Cheng, Hai, Haiwei Zhang, Jingyao Zhao, Hanying Li, Youfeng Ning, and Gayatri Kathayat. 2019. "Chinese Stalagmite Paleoclimate Researches: A Review and Perspective." *Science China Earth Sciences* 62 (10): 1489–1513. https://doi.org/10.1007/s11430-019-9478-3.

Cheng, H., A. Sinha, S. Verheyden, F. H. Nader, X. L. Li, P. Z. Zhang, J. J. Yin, et al. 2015a. "The Climate Variability in Northern Levant over the Past 20,000 Years: CLIMATE VARIABILITY IN NORTHERN LEVANT." *Geophysical Research Letters* 42 (20): 8641–50. https://doi.org/10.1002/2015gl065397.

———, et al. 2015b. "The Climate Variability in Northern Levant over the Past 20,000years." *Geophysical Research Letters* 42 (20): 8641–50. https://doi.org/10.1002/2015gl065397.

Cheng, H., P. Z. Zhang, C. Spötl, R. L. Edwards, Y. J. Cai, D. Z. Zhang, W. C. Sang, M. Tan, and Z. S. An. 2012. "The Climatic Cyclicity in Semiarid-Arid Central Asia over the Past 500,000 Years." *Geophysical Research Letters* 39 (1): n/a–. https://doi.org/10.1029/2011gl050202.

Cheng, Yu, Junwu Shu, Shefeng Hao, Bingfei Gao, Xiangqian Li, Feng Yuan, and Xinqing Zou. 2023. “Mid- to Late Holocene Vegetation Response to Relative Sea-Level Fluctuations Recorded by Multi-Proxy Evidence in the Subei Plain, Eastern China.” *Palaeogeography, Palaeoclimatology, Palaeoecology* 610 (January): 111327. https://doi.org/10.1016/j.palaeo.2022.111327.

Chernavskaya, M. M. 1988. “Climatic Changes in the Historical Past from Stratigraphical Sections of Moors on the Russian Plain.” *In: Data of Meteorological Studies*.

Chiessi, C. M., S. Mulitza, N. K. Taniguchi, M. Prange, M. C. Campos, C. Häggi, E. Schefuß, et al. 2021. “Mid- to Late Holocene Contraction of the Intertropical Convergence Zone over Northeastern South America.” *Paleoceanography and Paleoclimatology* 36 (4). https://doi.org/10.1029/2020pa003936.

Chiessi, Nicol’as M. Strand Cristiano M., Francisco W. Cruz, Mathias Vuille, Hai Cheng, Eline A. de Souza Barreto, Gesine Mollenhauer, Sabine Kasten, et al. 2015. “Timing and Structure of Mega-SACZ Events During Heinrich Stadial 1.” *Geophysical Research Letters* 42 (13): 5477. https://doi.org/10.1002/2015gl064048.

Ciais, P., J. Jouzel, J. R. Petit, V. Lipenkov, and J. W. C. White. 1994. “Holocene Temperature Variations Inferred from Antarctic Ice Cores.” *Annals of Glaciology* 20: 427–36. https://doi.org/10.3189/1994aog20-1-427-436.

Ciesla, A, M. Ralska-Jasiewiczowa, and E. Stupnicka. 1977. “Paleobotanical and Geochemical Investigations of the Lacustrine Deposits at Woryty Near Olsztyn (Northeastern Poland).” *Polskie Archiwum Hydrobiologii*.

Clegg, Benjamin F., Gina H. Clarke, Melissa L. Chipman, Michael Chou, Ian R. Walker, Willy Tinner, and Feng Sheng Hu. 2010. “Six Millennia of Summer Temperature Variation Based on Midge Analysis of Lake Sediments from Alaska.” *Quaternary Science Reviews* 29 (23-24): 3308–16. https://doi.org/10.1016/j.quascirev.2010.08.001.

Clegg, Benjamin F., and Feng Sheng Hu. 2010. “An Oxygen-Isotope Record of Holocene Climate Change in the South-Central Brooks Range, Alaska.” *Quaternary Science Reviews* 29 (7-8): 928–39. https://doi.org/10.1016/j.quascirev.2009.12.009.

Clegg, Benjamin F., Ryan Kelly, Gina H. Clarke, Ian R. Walker, and Feng Sheng Hu. 2011. “Nonlinear Response of Summer Temperature to Holocene Insolation Forcing in Alaska.” *Proceedings of the National Academy of Sciences* 108 (48): 19299–304. https://doi.org/10.1073/pnas.1110913108.

Clerc, J. 1988. “Recherches Pollenanalytiques Sur La Paleo-Ecologie Tardiglaciaire Et Holocene Du Bas-Dauphine.” *Doctoral Dissertation. Universite Aix-Marseille*.

Cobb, Kim M., Jess F. Adkins, Judson W. Partin, and Brian Clark. 2007. “Regional-Scale Climate Influences on Temporal Variations of Rainwater and Cave Dripwater Oxygen Isotopes in Northern Borneo.” *Earth and Planetary Science Letters* 263 (3-4): 207–20. https://doi.org/10.1016/j.epsl.2007.08.024.

Colombaroli, Daniele, Willy Tinner, Jacqueline van Leeuwen, Roland Noti, Elisa Vescovi, Boris Vanni‘ere, Michel Magny, Roland Schmidt, and Harald Bugmann. 2009. “Response of Broadleaved Evergreen Mediterranean Forest Vegetation to Fire Disturbance During the Holocene: Insights from the Peri-Adriatic Region.” *Journal of Biogeography* 36 (2): 314–26. https://doi.org/10.1111/j.1365-2699.2008.01987.x.

Comas-Bru, Laia, Kira Rehfeld, Carla Roesch, Sahar Amirnezhad-Mozhdehi, Sandy P. Harrison, Kamolphat Atsawawaranunt, Syed Masood Ahmad, et al. 2020. “SISALv2: A Comprehensive Speleothem Isotope Database with Multiple Age–Depth Models.” *Earth System Science Data* 12 (4): 2579–2606. https://doi.org/10.5194/essd-12-2579-2020.

Commerford, Julie L, B’erang‘ere Leys, Joshua R Mueller, and Kendra K McLauchlan. 2015. “Great Plains Vegetation Dynamics in Response to Fire and Climatic Fluctuations During the Holocene at Fox Lake, Minnesota (USA).” *The Holocene* 26 (2): 302–13. https://doi.org/10.1177/0959683615608691.

Comtois, Paul. 1982a. “Histoire Holocene Du Climat Et de La Vegetation a Lanoraie (Quebec).” *Canadian Journal of Earth Sciences* 19 (10): 1938–52. https://doi.org/10.1139/e82-172.

———. 1982b. “Histoire Holocene Du Climat Et de La Vegetation aLanoraie (Quebec).” *Canadian Journal of Earth Sciences* 19 (10): 1938–52. https://doi.org/10.1139/e82-172.

———. 1982c. “Histoire Holocène Du Climat Et de La Végétation à Lanoraie (Québec).” *Canadian Journal of Earth Sciences* 19 (10): 1938–52. https://doi.org/10.1139/e82-172.

Connor, Simon E., Daniele Colombaroli, Federico Confortini, Erika Gobet, Boris P. Ilyashuk, Elena A. Ilyashuk, Jacqueline F. N. van Leeuwen, et al. 2017. “Long-Term Population Dynamics: Theory and Reality in a Peatland Ecosystem.” Edited by Jacquelyn Gill. *Journal of Ecology* 106 (1): 333–46. https://doi.org/10.1111/1365-2745.12865.

Conroy, Jessica L., Adam M. Hudson, Jonathan T. Overpeck, Kam-Biu Liu, Luo Wang, and Julia E. Cole. 2017. “The Primacy of Multidecadal to Centennial Variability over Late-Holocene Forced Change of the Asian

Monsoon on the Southern Tibetan Plateau." *Earth and Planetary Science Letters* 458 (January): 337–48. https://doi.org/10.1016/j.epsl.2016.10.044.

Constantin, Silviu, Ana-Voica Bojar, Stein-Erik Lauritzen, and Joyce Lundberg. 2007. "Holocene and Late Pleistocene Climate in the Sub-Mediterranean Continental Environment: A Speleothem Record from Poleva Cave (Southern Carpathians, Romania)." *Palaeogeography, Palaeoclimatology, Palaeoecology* 243 (3–4): 322–38. https://doi.org/10.1016/j.palaeo.2006.08.001.

Cosford, Jason, Hairuo Qing, Bruce Eglington, Dave Mattey, Daoxiang Yuan, Meiliang Zhang, and Hai Cheng. 2008. "East Asian Monsoon Variability Since the Mid-Holocene Recorded in a High-Resolution, Absolute-Dated Aragonite Speleothem from Eastern China." *Earth and Planetary Science Letters* 275 (3-4): 296–307. https://doi.org/10.1016/j.epsl.2008.08.018.

Cosford, Jason, Hairuo Qing, Dave Mattey, Bruce Eglington, and Meiliang Zhang. 2009. "Climatic and Local Effects on Stalagmite δ13C Values at Lianhua Cave, China." *Palaeogeography, Palaeoclimatology, Palaeoecology* 280 (1–2): 235–44. https://doi.org/10.1016/j.palaeo.2009.05.020.

Crausbay, Shelley D., Patrick H. Martin, and Eugene F. Kelly. 2015a. "Tropical Montane Vegetation Dynamics Near the Upper Cloud Belt Strongly Associated with a Shifting ITCZ and Fire." Edited by Matt McGlone. *Journal of Ecology* 103 (4): 891–903. https://doi.org/10.1111/1365-2745.12423.

———. 2015b. "Tropical Montane Vegetation Dynamics Near the Upper Cloud Belt Strongly Associated with a Shifting <Scp>ITCZ</Scp> and Fire." Edited by Matt McGlone. *Journal of Ecology* 103 (4): 891–903. https://doi.org/10.1111/1365-2745.12423.

Crawford, J. N. 2012. "Evidence for Native American Land-Use Impacts on Forest Structure and Fire Regimes in the Lower Klamath River Region of California." *Doctoral Dissertation. University of Nevada*.

Cridlebaugh, P. A. 1984. "American Indian and Euro-American Impact Upon Holocene Vegetation in the Lower Little Tennessee River Valley, East Tennessee." *Doctoral Dissertation. University of Tennessee*.

Crosta, X., M. Debret, D. Denis, M. A. Courty, and O. Ther. 2007. "Holocene Long- and Short-Term Climate Changes Off Adelie Land, East Antarctica." *Geochemistry, Geophysics, Geosystems* 8 (11): n/a–. https://doi.org/10.1029/2007gc001718.

Cruz, Francisco W., Mathias Vuille, Stephen J. Burns, Xianfeng Wang, Hai Cheng, Martin Werner, R. Lawrence Edwards, Ivo Karmann, Augusto S. Auler, and Hanh Nguyen. 2009. "Orbitally Driven East-West Antiphasing of South American Precipitation." *Nature Geoscience* 2 (3): 210–14. https://doi.org/10.1038/ngeo444.

Cruz, Francisco W., Mathias Vuille, Stephen J. Burns, Xianfeng Wang, Hai Cheng, Martin Werner, R. Lawrence Edwards, Ivo Karmann, Augusto S. Auler, and Hanh Nguyen. 2009. "Orbitally Driven East–West Antiphasing of South American Precipitation." *Nature Geoscience* 2 (3): 210–14. https://doi.org/10.1038/ngeo444.

Cuffey, Kurt M., Gary D. Clow, Eric J. Steig, Christo Buizert, T. J. Fudge, Michelle Koutnik, Edwin D. Waddington, Richard B. Alley, and Jeffrey P. Severinghaus. 2016. "Deglacial Temperature History of West Antarctica." *Proceedings of the National Academy of Sciences* 113 (50): 14249–54. https://doi.org/10.1073/pnas.1609132113.

Cullen, H. M., P. B. deMenocal, S. Hemming, G. Hemming, F. H. Brown, T. Guilderson, and F. Sirocko. 2000. "Climate Change and the Collapse of the Akkadian Empire: Evidence from the Deep Sea." *Geology* 28 (4): 379. https://doi.org/10.1130/0091-7613(2000)28<379:ccatco>2.0.co;2.

Cumming, Brian F., Kathleen R. Laird, Joseph R. Bennett, John P. Smol, and Anne K. Salomon. 2002. "Persistent Millennial-Scale Shifts in Moisture Regimes in Western Canada During the Past Six Millennia." *Proceedings of the National Academy of Sciences* 99 (25): 16117–21. https://doi.org/10.1073/pnas.252603099.

Curdy, Philippe, J'erôme Bullinger, Pierre Crotti, Veruchka Valsecchi, and Willy Tinner. 2010. "Archaeological Survey Around the Simplon and Albrun Passes (Valais, Piemont), from Mesolihic to Roman Period." In *Archéologie de La Montagne Européenne*, edited by St'efan Tzortzis and Xavier Delestre, 4:185–95. Errance.

Curtis, Jason H., Mark Brenner, and David A. Hodell. 1999a. "Climate Change in the Lake Valencia Basin, Venezuela, 12600 Yr BP to Present." *The Holocene* 9 (5): 609–19. https://doi.org/10.1191/095968399669724431.

———. 1999b. "Climate Change in the Lake Valencia Basin, Venezuela, 12600 Yr BP to Present." *The Holocene* 9 (5): 609–19. https://doi.org/10.1191/095968399669724431.

Curtis, Jason H, Mark Brenner, David A Hodell, Richard A Balser, Gerald A Islebe, and Henry Hooghiemstra. 1998. "A Multi-Proxy Study of Holocene Environmental Change in the Maya Lowlands of Peten, Guatemala." *Journal of Paleolimnology* 19: 139–59.

Cwynar, Les C. 1990. "A Late Quaternary Vegetation History from Lily Lake, Chilkat Peninsula, Southeast Alaska." *Canadian Journal of Botany* 68 (5): 1106–12. https://doi.org/10.1139/b90-139.

Cyprien, A. L. 2001. "Chronologie de l'interaction de l'homme Et Du Milieu Dans l'espace Central Et Aval de La Loire (Ouest de La France)." *Doctoral Dissertation. Universite de Nantes*.

Cyprien, A. L, L. Visset, and L. Charrieau. 2004. "Etudes Palynologiques Du Site de La Grande Brousse, Vallee de La Choisille, Communes de Cerelles Et Chanceaux-Sur-Choisille (Indre-Et-Loire)." *Rapport Definitif*.

Czymzik, Markus, Achim Brauer, Peter Dulski, Birgit Plessen, Rudolf Naumann, Ulrich von Grafenstein, and Raphael Scheffler. 2013. "Orbital and Solar Forcing of Shifts in Mid- to Late Holocene Flood Intensity from Varved Sediments of Pre-Alpine Lake Ammersee (Southern Germany)." *Quaternary Science Reviews* 61 (February): 96–110. https://doi.org/10.1016/j.quascirev.2012.11.010.

D'Andrea, William J., Yongsong Huang, Sherilyn C. Fritz, and N. John Anderson. 2011. "Abrupt Holocene Climate Change as an Important Factor for Human Migration in West Greenland." *Proceedings of the National Academy of Sciences* 108 (24): 9765–69. https://doi.org/10.1073/pnas.1101708108.

Dalton, Catherine, H. J. B. Birks, Stephen J. Brooks, Nigel G. Cameron, Richard P. Evershed, Sylvia M. Peglar, Julie A. Scott, and Roy Thompson. 2005. "A Multi-Proxy Study of Lake-Development in Response to Catchment Changes During the Holocene at Lochnagar, North-East Scotland." *Palaeogeography, Palaeoclimatology, Palaeoecology* 221 (3-4): 175–201. https://doi.org/10.1016/j.palaeo.2005.02.007.

Dang, Haowen, Zhimin Jian, Franck Bassinot, Peijun Qiao, and Xinrong Cheng. 2012. "Decoupled Holocene Variability in Surface and Thermocline Water Temperatures of the Indo-pacific Warm Pool." *Geophysical Research Letters* 39 (1). https://doi.org/10.1029/2011gl050154.

Dang, Shaohua, Kefu Yu, Shichen Tao, Tao Han, Huiling Zhang, and Wei Jiang. 2020. "El Niño/Southern Oscillation During the 4.2 Ka Event Recorded by Growth Rates of Corals from the North South China Sea." *Acta Oceanologica Sinica* 39 (1): 110–17. https://doi.org/10.1007/s13131-019-1520-5.

Davies, F. M. 1997. "Holocene Palaeoenvironmental Studies in the Oban Region, Western Scotland, Upon Tyne." *Doctoral Dissertation. Department of Geography*.

Davies-Walczak, Maureen, A. C. Mix, J. S. Stoner, J. R. Southon, M. Cheseby, and C. Xuan. 2014. "Late Glacial to Holocene Radiocarbon Constraints on North Pacific Intermediate Water Ventilation and Deglacial Atmospheric CO2 Sources." *Earth and Planetary Science Letters* 397 (July): 57–66. https://doi.org/10.1016/j.epsl.2014.04.004.

Davis, A. M., J. H. McAndrews, and B. L. Wallace. 1988a. "Paleoenvironment and the Archaeological Record at the lanse Aux Meadows Site, Newfoundland." *Geoarchaeology* 3 (1): 53–64. https://doi.org/10.1002/gea.3340030104.

———. 1988b. "Paleoenvironment and the Archaeological Record at the l'anse Aux Meadows Site, Newfoundland." *Geoarchaeology* 3 (1): 53–64. https://doi.org/10.1002/gea.3340030104.

de Beaulieu, J. L. 1974. "Evolution de La Vegetation Sur La Bordure Montagneuse Cevenole Au Postglaciare, d'apres Les Pollens." *Bulletin de La Societe Languedocienne de Geographie*.

———. 1977. "Contribution Pollenanalytique a l'histoire Tardiglaciaire Et Holocene de La Vegetation Des Alpes Meridionales Francaices." *Doctoral Dissertation. Universite d'Aix-Marseille*.

de Beaulieu, J. L, P Leveau, C Miramont, J. M Palet, K Walsh, F Ricou, M Segard, et al. 2003. "Changements Environnementaux Postglaciaires Et Action de l'homme Dans Le Bassin Du Buech Et En Champasaur (Hautes-Alpes, France)." *Premier Bilan d'une Etude Pluridisciplinaire. In: Collection Environnement (Pp.93-101). Des Milieux Et Des Hommes: Fragments d'Histoires Croisees*.

De Beaulieu, Jacques Louis, and Claude Goeury. 1987. "Zonation Automatique Appliquee Alanalyse Pollinique : Exemple de La Narse dampoix (Puy-de-dôme, France)." *Bulletin de lAssociation Française Pour letude Du Quaternaire* 24 (1): 49–61. https://doi.org/10.3406/quate.1987.1831.

de Garidel-Thoron, Thibault, Luc Beaufort, Braddock K. Linsley, and Stefanie Dannenmann. 2001. "Millennial-Scale Dynamics of the East Asian Winter Monsoon During the Last 200,000 Years." *Paleoceanography* 16 (5): 491–502. https://doi.org/10.1029/2000pa000557.

de Klerk, P. 2005. "A Pollen Diagram from the Ahlbecker Seegrund (Ueckermunder Heide, Vorpommern, NE Germany) from the Legacy of Franz Fukarek." *Archiv Fur Naturschutz Und Landschaftsforschung*.

de Klerk, Pim, Andreas Haberl, Andreas Kaffke, Matthias Krebs, Izolda Matchutadze, Merten Minke, Jenny Schulz, and Hans Joosten. 2009. "Vegetation History and Environmental Development Since Ca 6000 Cal Yr BP in

and Around Ispani 2 (Kolkheti Lowlands, Georgia).” *Quaternary Science Reviews* 28 (9-10): 890–910. https://doi.org/10.1016/j.quascirev.2008.12.005.
de Valk, E. J. 1981. “Late Holocene and Present Vegetation of the Kastelberg (Vosges, France).” *Doctoral Dissertation. University of Utrecht.*
de Vernal, Anne, Claude Hillaire-Marcel, and Dennis A. Darby. 2005. “Variability of Sea Ice Cover in the Chukchi Sea (Western Arctic Ocean) During the Holocene.” *Paleoceanography* 20 (4): n/a–. https://doi.org/10.1029/2005pa001157.
de Vernal, Anne, Claude Hillaire-Marcel, Andr’e Rochon, Bianca Fr’echette, Maryse Henry, Sandrine Solignac, and Sophie Bonnet. 2013. “Dinocyst-Based Reconstructions of Sea Ice Cover Concentration During the Holocene in the Arctic Ocean, the Northern North Atlantic Ocean and Its Adjacent Seas.” *Quaternary Science Reviews* 79 (November): 111–21. https://doi.org/10.1016/j.quascirev.2013.07.006.
Dean, Jonathan R., Matthew D. Jones, Melanie J. Leng, Stephen R. Noble, Sarah E. Metcalfe, Hilary J. Sloane, Diana Sahy, Warren J. Eastwood, and C. Neil Roberts. 2015. “Eastern Mediterranean Hydroclimate over the Late Glacial and Holocene, Reconstructed from the Sediments of Nar Lake, Central Turkey, Using Stable Isotopes and Carbonate Mineralogy.” *Quaternary Science Reviews* 124 (September): 162–74. https://doi.org/10.1016/j.quascirev.2015.07.023.
deMenocal, Peter, Joseph Ortiz, Tom Guilderson, Jess Adkins, Michael Sarnthein, Linda Baker, and Martha Yarusinsky. 2000. “Abrupt Onset and Termination of the African Humid Period:” *Quaternary Science Reviews* 19 (1-5): 347–61. https://doi.org/10.1016/s0277-3791(99)00081-5.
Denniston, R. F., Y. Asmerom, V. J. Polyak, A. D. Wanamaker, C. C. Ummenhofer, W. F. Humphreys, J. Cugley, D. Woods, and S. Lucker. 2017. “Decoupling of Monsoon Activity Across the Northern and Southern Indo-Pacific During the Late Glacial.” *Quaternary Science Reviews* 176 (November): 101–5. https://doi.org/10.1016/j.quascirev.2017.09.014.
Denniston, Rhawn F., Amanda N. Houts, Yemane Asmerom, Alan D. Wanamaker Jr., Jonathan A. Haws, Victor J. Polyak, Diana L. Thatcher, et al. 2018. “A Stalagmite Test of North Atlantic SST and Iberian Hydroclimate Linkages over the Last Two Glacial Cycles.” *Climate of the Past* 14 (12): 1893–913. https://doi.org/10.5194/cp-14-1893-2018.
Denniston, Rhawn F., Caroline C. Ummenhofer, Alan D. Wanamaker, Matthew S. Lachniet, Gabriele Villarini, Yemane Asmerom, Victor J. Polyak, et al. 2016. “Expansion and Contraction of the Indo-Pacific Tropical Rain Belt over the Last Three Millennia.” *Scientific Reports* 6 (1). https://doi.org/10.1038/srep34485.
Denniston, Rhawn F., Gabriele Villarini, Angelique N. Gonzales, Karl-Heinz Wyrwoll, Victor J. Polyak, Caroline C. Ummenhofer, Matthew S. Lachniet, et al. 2015a. “Extreme Rainfall Activity in the Australian Tropics Reflects Changes in the El Nino/Southern Oscillation over the Last Two Millennia.” *Proceedings of the National Academy of Sciences* 112 (15): 4576–81. https://doi.org/10.1073/pnas.1422270112.
———, et al. 2015b. “Extreme Rainfall Activity in the Australian Tropics Reflects Changes in the El Niño/Southern Oscillation over the Last Two Millennia.” *Proceedings of the National Academy of Sciences* 112 (15): 4576–81. https://doi.org/10.1073/pnas.1422270112.
Denniston, Rhawn F., Karl-Heinz Wyrwoll, Victor J. Polyak, Josephine R. Brown, Yemane Asmerom, Alan D. Wanamaker, Zachary LaPointe, et al. 2013a. “A Stalagmite Record of Holocene Indonesian-Australian Summer Monsoon Variability from the Australian Tropics.” *Quaternary Science Reviews* 78 (October): 155–68. https://doi.org/10.1016/j.quascirev.2013.08.004.
———, et al. 2013b. “A Stalagmite Record of Holocene Indonesian–Australian Summer Monsoon Variability from the Australian Tropics.” *Quaternary Science Reviews* 78 (October): 155–68. https://doi.org/10.1016/j.quascirev.2013.08.004.
Deplazes, Gaudenz, Andreas Lückge, Larry C. Peterson, Axel Timmermann, Yvonne Hamann, Konrad A. Hughen, Ursula Röhl, et al. 2013. “Links Between Tropical Rainfall and North Atlantic Climate During the Last Glacial Period.” *Nature Geoscience* 6 (3): 213–17. https://doi.org/10.1038/ngeo1712.
Deplazes, Gaudenz, Andreas Lückge, Jan-Berend W. Stuut, Jürgen Pätzold, Holger Kuhlmann, Doroth’ee Husson, Mara Fant, and Gerald H. Haug. 2014a. “Weakening and Strengthening of the Indian Monsoon During Heinrich Events and Dansgaard-Oeschger Oscillations.” *Paleoceanography* 29 (2): 99–114. https://doi.org/10.1002/2013pa002509.
———. 2014b. “Weakening and Strengthening of the Indian Monsoon During Heinrich Events and Dansgaard-Oeschger Oscillations: INDIAN MONSOON DURING HEINRICH EVENTS.” *Paleoceanography* 29 (2): 99–114. https://doi.org/10.1002/2013pa002509.
Di Rita, Federico, Matthieu Ghilardi, Nathalie Fagel, Matteo Vacchi, François Warichet, Doriane Delanghe, Jean Sicurani, Lauriane Martinet, and S’ebastien Robresco. 2022. “Natural and Anthropogenic Dynamics of the

Coastal Environment in Northwestern Corsica (Western Mediterranean) over the Past Six Millennia." *Quaternary Science Reviews* 278 (February): 107372. https://doi.org/10.1016/j.quascirev.2022.107372.
Di Rita, Federico, Fabrizio Lirer, Sergio Bonomo, Antonio Cascella, Luciana Ferraro, Fabio Florindo, Donatella Domenica Insinga, et al. 2018. "Late Holocene Forest Dynamics in the Gulf of Gaeta (Central Mediterranean) in Relation to NAO Variability and Human Impact." *Quaternary Science Reviews* 179 (January): 137–52. https://doi.org/10.1016/j.quascirev.2017.11.012.
Di Rita, Federico, and Donatella Magri. 2019. "The 4.2 Ka Event in the Vegetation Record of the Central Mediterranean." *Climate of the Past* 15 (1): 237–51. https://doi.org/10.5194/cp-15-237-2019.
Diaconu, Andrei-Cosmin, M'onika T'oth, Mariusz Lamentowicz, Oliver Heiri, Eliza Kuske, Ioan Tanţău, Andrei-Marian Panait, Mih'aly Braun, and Angelica Feurdean. 2017. "How Warm? How Wet? Hydroclimate Reconstruction of the Past 7500 Years in Northern Carpathians, Romania." *Palaeogeography, Palaeoclimatology, Palaeoecology* 482 (September): 1–12. https://doi.org/10.1016/j.palaeo.2017.05.007.
Digerfeldt, G. 1977. "The Flandrian Development of Lake Flarken." *Regional Vegetation History and Palaeolimnology: Report 13. University of Lund.*
———. 1982. "The Holocene Development of Lake Sambosjon." *1. The Regional Vegetation History. Lundqua Report 23.*
Dinel, H., P. J. H. Richard, P. E. M. Lev'esque, and A. Larouche. 1986a. "Origine Et Evolution Du Marais Tourbeux de Keswick, Ontario, Par lanalyse Pollinique Et Macrofossile." *Canadian Journal of Earth Sciences* 23 (8): 1145–55. https://doi.org/10.1139/e86-113.
———. 1986b. "Origine Et Évolution Du Marais Tourbeux de Keswick, Ontario, Par l'analyse Pollinique Et Macrofossile." *Canadian Journal of Earth Sciences* 23 (8): 1145–55. https://doi.org/10.1139/e86-113.
Ding, Wei, Qinghai Xu, and Pavel E. Tarasov. 2017. "Examining Bias in Pollen-Based Quantitative Climate Reconstructions Induced by Human Impact on Vegetation in China." *Climate of the Past* 13 (9): 1285–1300. https://doi.org/10.5194/cp-13-1285-2017.
Dixit, Y., D. A. Hodell, and C. A. Petrie. 2014. "Abrupt Weakening of the Summer Monsoon in Northwest India 4100 Yr Ago." *Geology* 42 (4): 339–42. https://doi.org/10.1130/g35236.1.
Djamali, Morteza, Jacques-Louis de Beaulieu, Val'erie Andrieu-Ponel, Manuel Berberian, Naomi F. Miller, Emmanuel Gandouin, Hamid Lahijani, et al. 2009. "A Late Holocene Pollen Record from Lake Almalou in NW Iran: Evidence for Changing Land-Use in Relation to Some Historical Events During the Last 3700 Years." *Journal of Archaeological Science* 36 (7): 1364–75. https://doi.org/10.1016/j.jas.2009.01.022.
Djamali, Morteza, Jacques-Louis De Beaulieu, Naomi F. Miller, Val'erie Andrieu-Ponel, Philippe Ponel, Razieh Lak, Nasser Sadeddin, Hossein Akhani, and Hassan Fazeli. 2008. "Vegetation History of the SE Section of the Zagros Mountains During the Last Five Millennia; a Pollen Record from the Maharlou Lake, Fars Province, Iran." *Vegetation History and Archaeobotany* 18 (2): 123–36. https://doi.org/10.1007/s00334-008-0178-2.
Dodson, J. R., and R. H. W. Bradshaw. 2008. "A History of Vegetation and Fire, 6,600 b.p. To Present, County Sligo, Western Ireland." *Boreas* 16 (2): 113–23. https://doi.org/10.1111/j.1502-3885.1987.tb00762.x.
Doerfler, W. 1989. "Pollenanalytische Untersuchungen Zur Vegetations- Und Siedlungsgeschichte Im Sueden Des Landkreises Cuxhaven, Niedersachsen." *Probleme Der Kuestenforschung Im Suedlichen Nordseegebiet* 17: 1 75.
Dolukhanov, P M, A M Shukurov, Kh A Arslanov, D A Subetto, G I Zaitseva, E N Djinoridze, D D Kuznetsov, A V Ludikova, T V Sapelko, and L A Savelieva. 2007. "Evolution of Waterways and Early Human Settlements in the Eastern Baltic Area: Radiocarbon-Based Chronology." *Radiocarbon* 49 (2): 527–42. https://doi.org/10.1017/s0033822200042442.
Dolven, Jane K., Giuseppe Cortese, and Kjell R. Bjørklund. 2002a. "A High-Resolution Radiolarian-Derived Paleotemperature Record for the Late Pleistocene-Holocene in the Norwegian Sea." *Paleoceanography* 17 (4): 24-1-24-13. https://doi.org/10.1029/2002pa000780.
———. 2002b. "A High-resolution Radiolarian-derived Paleotemperature Record for the Late Pleistocene-holocene in the Norwegian Sea." *Paleoceanography* 17 (4). https://doi.org/10.1029/2002pa000780.
Domack, E., A. Leventer, R. Dunbar, F. Taylor, S. Brachfeld, and C. Sjunneskog. 2001. "Chronology of the Palmer Deep Site, Antarctic Peninsula: A Holocene Palaeoenvironmental Reference for the Circum-Antarctic." *The Holocene* 11 (1): 1–9. https://doi.org/10.1191/095968301673881493.
Donders, Timme H., Friederike Wagner, David L. Dilcher, and Henk Visscher. 2005a. "Mid- to Late-Holocene El Nino-Southern Oscillation Dynamics Reflected in the Subtropical Terrestrial Realm." *Proceedings of the National Academy of Sciences* 102 (31): 10904–8. https://doi.org/10.1073/pnas.0505015102.

———. 2005b. “Mid- to Late-Holocene El Niño-Southern Oscillation Dynamics Reflected in the Subtropical Terrestrial Realm.” *Proceedings of the National Academy of Sciences* 102 (31): 10904–8. https://doi.org/10.1073/pnas.0505015102.

Dong, Haowei, Manman Xie, Wenyu Shang, Siwen Liu, Yuan Ling, Nan Zhan, Shuxian Wang, Qi Li, Guoqiang Chu, and Qing Sun. 2022. “Plant-Wax Carbon Isotopic Evidence of Lateglacial and Holocene Climate Change from Lake Sediments in the Yin Mountains, Inner Mongolia.” *Quaternary International* 622 (June): 10–20. https://doi.org/10.1016/j.quaint.2021.12.017.

Dong, Jinguo, Yongjin Wang, Hai Cheng, Ben Hardt, R. Lawrence Edwards, Xinggong Kong, Jiangying Wu, et al. 2010. “A High-Resolution Stalagmite Record of the Holocene East Asian Monsoon from Mt Shennongjia, Central China.” *The Holocene* 20 (2): 257–64. https://doi.org/10.1177/0959683609350393.

Dorfler, W. 1989. “Pollenanalytische Untersuchungen Zur Vegetations- Und Siedlungsgeschichte Im Suden Des Landkreises Cuxhaven, Niedersachsen.” *Probleme Der Kustenforschung Im Sudlichen Nordseegebiet*.

Drake, Brandon L, WH Wills, and Erik B Erhardt. 2012. “The 5.1 Ka Aridization Event, Expansion of Piñon-Juniper Woodlands, and the Introduction of Maize (Zea Mays) in the American Southwest.” *The Holocene* 22 (12): 1353–60. https://doi.org/10.1177/0959683612449758.

Dreger, Derek. 1999. “Decadal-to-Centenial-Scale Sediment Records of Ice Advance on the Barents Shelf and Meltwater Discharge into the Northeastern Norwegian Sea over the Last 40 Kyr.” *Berichte - Reports*. Institut für Geowissenschaften, Christian-Albrechts-Universität, Kiel. https://doi.org/10.2312/REPORTS-IFG.1999.3.

Drescher-Schneider, Ruth, Jacques-Louis de Beaulieu, Michel Magny, Anne-V’eronique Walter-Simonnet, Gilles Bossuet, Laurent Millet, Elisabetta Brugiapaglia, and Anton Drescher. 2007. “Vegetation History, Climate and Human Impact over the Last 15,000 Years at Lago Dell’accesa (Tuscany, Central Italy).” *Vegetation History and Archaeobotany* 16 (4): 279–99. https://doi.org/10.1007/s00334-006-0089-z.

Dreslerova, D, E Brizova, E. Ruzickova, and A. Zeman. 2004. “Holocene Environmental Processes and Alluvial Archaeology in the Middle Labe (Elbe) Valley.” *In: Ancient Landscape*.

Drysdale, Russell, Giovanni Zanchetta, John Hellstrom, Roland Maas, Anthony Fallick, Matthew Pickett, Ian Cartwright, and Leonardo Piccini. 2006. “Late Holocene Drought Responsible for the Collapse of Old World Civilizations Is Recorded in an Italian Cave Flowstone.” *Geology* 34 (2): 101. https://doi.org/10.1130/g22103.1.

Du, Xiaojing, Ingrid Hendy, and Arndt Schimmelmann. 2018. “A 9000-Year Flood History for Southern California: A Revised Stratigraphy of Varved Sediments in Santa Barbara Basin.” *Marine Geology* 397 (March): 29–42. https://doi.org/10.1016/j.margeo.2017.11.014.

Duarte, Edward, Jonathan Obrist-Farner, Alex Correa-Metrio, and Byron A. Steinman. 2021. “A Progressively Wetter Early Through Middle Holocene Climate in the Eastern Lowlands of Guatemala.” *Earth and Planetary Science Letters* 561 (May): 116807. https://doi.org/10.1016/j.epsl.2021.116807.

Dunnette, Paul V., Philip E. Higuera, Kendra K. McLauchlan, Kelly M. Derr, Christy E. Briles, and Margaret H. Keefe. 2014a. “Biogeochemical Impacts of Wildfires over Four Millennia in a Rocky Mountain Subalpine Watershed.” *New Phytologist* 203 (3): 900–912. https://doi.org/10.1111/nph.12828.

———. 2014b. “Biogeochemical Impacts of Wildfires over Four Millennia in a <Scp>r</Scp>ocky <Scp>m</Scp>ountain Subalpine Watershed.” *New Phytologist* 203 (3): 900–912. https://doi.org/10.1111/nph.12828.

Dunwiddie, P. W. 1990. “Postglacial Vegetation History of Coastal Islands in Southeastern New-England.” *National Geographic Research*.

Dutt, Som, Anil K. Gupta, Steven C. Clemens, Hai Cheng, Raj K. Singh, Gayatri Kathayat, and R. Lawrence Edwards. 2015a. “Abrupt Changes in Indian Summer Monsoon Strength During 33,800 to 5500 Years b.p.” *Geophysical Research Letters* 42 (13): 5526–32. https://doi.org/10.1002/2015gl064015.

———. 2015b. “Abrupt Changes in Indian Summer Monsoon Strength During 33,800 to 5500years b.p.” *Geophysical Research Letters* 42 (13): 5526–32. https://doi.org/10.1002/2015gl064015.

Dutt, Som, Anil K. Gupta, Rahul Devrani, Ram R. Yadav, and Raj K. Singh. 2021. “Regional Disparity in Summer Monsoon Precipitation in the Indian Subcontinent During Northgrippian to Meghalayan Transition.” *Current Science* 120 (9): 1449. https://doi.org/10.18520/cs/v120/i9/1449-1457.

Dwyer, R. B. 1995. “Blanket Bog Initiation and Development in North West Mayo, Ireland.” *Doctoral Dissertation. University of Dublin*.

Dykoski, C., R. Edwards, H. Cheng, D. Yuan, Y. Cai, M. Zhange, Y. Lin, J. Qing, Z. An, and J. Revenaugh. 2005. “A High-Resolution, Absolute-Dated Holocene and Deglacial Asian Monsoon Record from Dongge Cave, China.” *Earth and Planetary Science Letters* 233 (1-2): 71–86. https://doi.org/10.1016/j.epsl.2005.01.036.

Eastwood, Warren J., Melanie J. Leng, Neil Roberts, and Basil Davis. 2006. “Holocene Climate Change in the Eastern Mediterranean Region: A Comparison of Stable Isotope and Pollen Data from Lake Gölhisar, Southwest Turkey.” *Journal of Quaternary Science* 22 (4): 327–41. https://doi.org/10.1002/jqs.1062.
———. 2007. “Holocene Climate Change in the Eastern Mediterranean Region: A Comparison of Stable Isotope and Pollen Data from Lake Gölhisar, Southwest Turkey.” *Journal of Quaternary Science* 22 (4): 327–41. https://doi.org/10.1002/jqs.1062.
Ellison, Christopher R. W., Mark R. Chapman, and Ian R. Hall. 2006. “Surface and Deep Ocean Interactions During the Cold Climate Event 8200 Years Ago.” *Science* 312 (5782): 1929–32. https://doi.org/10.1126/science.1127213.
Emeis, Kay-Christian, and Alastair G. Dawson. 2003. “Holocene Palaeoclimate Records over Europe and the North Atlantic.” *The Holocene* 13 (3): 305–9. https://doi.org/10.1191/0959683603hl622ed.
Emeis, Kay-Christian, Ulrich Struck, Thomas Blanz, Alexander Kohly, and Maren Vo\upbeta. 2003. “Salinity Changes in the Central Baltic Sea (NW Europe) over the Last 10000 Years.” *The Holocene* 13 (3): 411–21. https://doi.org/10.1191/0959683603hl634rp.
Emeis, Kay-Christian, Ulrich Struck, Hans-Martin Schulz, Reinhild Rosenberg, Stefano Bernasconi, Helmut Erlenkeuser, Tatsuhiko Sakamoto, and Francisca Martinez-Ruiz. 2000. “Temperature and Salinity Variations of Mediterranean Sea Surface Waters over the Last 16,000 Years from Records of Planktonic Stable Oxygen Isotopes and Alkenone Unsaturation Ratios.” *Palaeogeography, Palaeoclimatology, Palaeoecology* 158 (3-4): 259–80. https://doi.org/10.1016/s0031-0182(00)00053-5.
Ersek, Vasile, Peter U. Clark, Alan C. Mix, Hai Cheng, and R. Lawrence Edwards. 2012. “Holocene Winter Climate Variability in Mid-Latitude Western North America.” *Nature Communications* 3 (1). https://doi.org/10.1038/ncomms2222.
Eynaud, Fr’ed’erique, L’ucia de Abreu, Antje Voelker, Joachim Schönfeld, Emilia Salgueiro, Jean-Louis Turon, Aur’elie Penaud, et al. 2009. “Position of the Polar Front Along the Western Iberian Margin During Key Cold Episodes of the Last 45 Ka.” *Geochemistry, Geophysics, Geosystems* 10 (7): n/a–. https://doi.org/10.1029/2009gc002398.
Fallu, Marie-Andr’ee, Reinhard Pienitz, Ian R. Walker, and Martin Lavoie. 2005. “Paleolimnology of a Shrub-Tundra Lake and Response of Aquatic and Terrestrial Indicators to Climatic Change in Arctic Québec, Canada.” *Palaeogeography, Palaeoclimatology, Palaeoecology* 215 (3–4): 183–203. https://doi.org/10.1016/j.palaeo.2004.07.006.
Fan, Weijia, Zhimin Jian, Zhihui Chu, Haowen Dang, Yue Wang, Franck Bassinot, Xiqiu Han, and Yeping Bian. 2018. “Variability of the Indonesian Throughflow in the Makassar Strait over the Last 30ka.” *Scientific Reports* 8 (1). https://doi.org/10.1038/s41598-018-24055-1.
Farcas, S, V Lupsa, I. Tantau, and A. Bodnariuc. 2003. “Reflectarea Procesului de Antropizare in Diagramele Sporo-Polinice Din Muntii Apuseni.” *Environment and Progress*.
Farcas, Sorina, Jacques-Louis de Beaulieu, Maurice Reille, George Coldea, Baluta Diaconeasa, Claude Goeury, Thomas Goslar, and Timothy Jull. 1999. “First 14C Datings of Late Glacial and Holocene Pollen Sequences from Romanian Carpathes.” *Comptes Rendus de lAcademie Des Sciences - Series III - Sciences de La Vie* 322 (9): 799–807. https://doi.org/10.1016/s0764-4469(00)80039-6.
Farmer, E. Christa, Peter B. deMenocal, and Thomas M. Marchitto. 2005. “Holocene and Deglacial Ocean Temperature Variability in the Benguela Upwelling Region: Implications for Low-Latitude Atmospheric Circulation.” *Paleoceanography* 20 (2): n/a–. https://doi.org/10.1029/2004pa001049.
Farmer, Elizabeth J., Mark R. Chapman, and Julian E. Andrews. 2008. “Centennial-scale Holocene North Atlantic Surface Temperatures from Mg/Ca Ratios in Globigerina Bulloides.” *Geochemistry, Geophysics, Geosystems* 9 (12). https://doi.org/10.1029/2008gc002199.
Farmer, Jesse R., Thomas M. Cronin, Anne de Vernal, Gary S. Dwyer, Lloyd D. Keigwin, and Robert C. Thunell. 2011. “Western Arctic Ocean Temperature Variability During the Last 8000 Years: ARCTIC OCEAN TEMPERATURE VARIABILITY.” *Geophysical Research Letters* 38 (24): n/a–. https://doi.org/10.1029/2011gl049714.
Fastovich, David, James M. Russell, Stephen T. Jackson, and John W. Williams. 2020. “Deglacial Temperature Controls on No-Analog Community Establishment in the Great Lakes Region.” *Quaternary Science Reviews* 234 (April): 106245. https://doi.org/10.1016/j.quascirev.2020.106245.
Fedotov, Andrey, Svetlana Vorobyeva, Konstantin Vershinin, and Eduard Osipov. 2023. “Climate Changes in the South Part of East Siberia for the Last 5.5 Ka Inferred from Multi-Proxy Sediment Records of Lake Frolikha (Northern Baikal Area, Russia).” *Quaternary International* 644–645 (January): 41–50. https://doi.org/10.1016/j.quaint.2021.11.021.

Fegyveresi, John M., Richard B. Alley, Joan J. Fitzpatrick, Kurt M. Cuffey, Joseph R. McConnell, Donald E. Voigt, Matthew K. Spencer, and Nathan T. Stevens. 2016. "Five Millennia of Surface Temperatures and Ice Core Bubble Characteristics from the WAIS Divide Deep Core, West Antarctica." *Paleoceanography* 31 (3): 416–33. https://doi.org/10.1002/2015pa002851.

Fensterer, Claudia, Denis Scholz, Dirk L. Hoffmann, Christoph Spötl, Andrea Schröder-Ritzrau, Christian Horn, Jesus M. Paj'on, and Augusto Mangini. 2013. "Millennial-Scale Climate Variability During the Last 12.5 Ka Recorded in a Caribbean Speleothem." *Earth and Planetary Science Letters* 361 (January): 143–51. https://doi.org/10.1016/j.epsl.2012.11.019.

Fensterer, Claudia, Denis Scholz, Dirk Hoffmann, Christoph Spötl, Jes'us M Paj'on, and Augusto Mangini. 2012. "Cuban Stalagmite Suggests Relationship Between Caribbean Precipitation and the Atlantic Multidecadal Oscillation During the Past 1.3 Ka." *The Holocene* 22 (12): 1405–12. https://doi.org/10.1177/0959683612449759.

Fensterer, C, D Scholz, D Hoffmann, A Mangini, and J M Paj'on. 2010a. "230Th/u-Dating of a Late Holocene Low Uranium Speleothem from Cuba." *IOP Conference Series: Earth and Environmental Science* 9 (March): 012015. https://doi.org/10.1088/1755-1315/9/1/012015.

———. 2010b. "230Th/u-Dating of a Late Holocene Low Uranium Speleothem from Cuba." *IOP Conference Series: Earth and Environmental Science* 9 (March): 012015. https://doi.org/10.1088/1755-1315/9/1/012015.

Feurdean, Angelica. 2005. "Holocene Forest Dynamics in Northwestern Romnania." *The Holocene* 15 (3): 435–46. https://doi.org/10.1191/0959683605hl803rp.

Finkelstein, Sarah A., Matthew C. Peros, and Anthony M. Davis. 2005. "Late Holocene Paleoenvironmental Change in a Great Lakes Coastal Wetland: Integrating Pollen and Diatom Datasets." *Journal of Paleolimnology* 33 (1): 1–12. https://doi.org/10.1007/s10933-004-0423-3.

Finkenbinder, Matthew S., Mark B. Abbott, Mary E. Edwards, Catherine T. Langdon, Byron A. Steinman, and Bruce P. Finney. 2014. "A 31,000 Year Record of Paleoenvironmental and Lake-Level Change from Harding Lake, Alaska, USA." *Quaternary Science Reviews* 87 (March): 98–113. https://doi.org/10.1016/j.quascirev.2014.01.005.

Finn'e, Martin, Karin Holmgren, Chuan-Chou Shen, Hsun-Ming Hu, Meighan Boyd, and Sharon Stocker. 2017. "Late Bronze Age Climate Change and the Destruction of the Mycenaean Palace of Nestor at Pylos." Edited by John P. Hart. *PLOS ONE* 12 (12): e0189447. https://doi.org/10.1371/journal.pone.0189447.

Finney, Bruce P., Nancy H. Bigelow, Valerie A. Barber, and Mary E. Edwards. 2012. "Holocene Climate Change and Carbon Cycling in a Groundwater-Fed, Boreal Forest Lake: Dune Lake, Alaska." *Journal of Paleolimnology* 48 (1): 43–54. https://doi.org/10.1007/s10933-012-9617-2.

Finsinger, Walter, Daniele Colombaroli, Jacques-Louis De Beaulieu, Verushka Valsecchi, Boris Vanni'ere, Elisa Vescovi, Emmanuel Chapron, Andr'e F. Lotter, Michel Magny, and Willy Tinner. 2010. "Early to Mid-Holocene Climate Change at Lago Dellaccesa (Central Italy): Climate Signal or Anthropogenic Bias?" *Journal of Quaternary Science* 25 (8): 1239–47. https://doi.org/10.1002/jqs.1402.

Finsinger, Walter, Daniele Colombaroli, Jacques Louis De Beaulieu, Verushka Valsecchi, Boris Vanni'ere, Elisa Vescovi, Emmanuel Chapron, Andr'e F. Lotter, Michel Magny, and Willy Tinne. 2010. "Early to Mid-holocene Climate Change at Lago Dell'accesa (Central Italy): Climate Signal or Anthropogenic Bias?" *Journal of Quaternary Science* 25 (8): 1239–47. https://doi.org/10.1002/jqs.1402.

Finsinger, Walter, and Willy Tinner. 2006. "Holocene Vegetation and Land-Use Changes in Response to Climatic Changes in the Forelands of the Southwestern Alps, Italy." *Journal of Quaternary Science* 21 (3): 243–58. https://doi.org/10.1002/jqs.971.

Finsinger, Walter, Quentin Vanel, Adriano Ribolini, and Willy Tinner. 2020. "Early to Late Holocene Vegetation and Fire Dynamics at the Treeline in the Maritime Alps." *Vegetation History and Archaeobotany* 30 (4): 507–24. https://doi.org/10.1007/s00334-020-00795-x.

Fisher, David A., Roy M. Koerner, Jocelyne C. Bourgeois, Greg Zielinski, Cameron Wake, Claus U. Hammer, H. B. Clausen, et al. 1998. "Penny Ice Cap Cores, Baffin Island, Canada, and the Wisconsinan Foxe Dome Connection: Two States of Hudson Bay Ice Cover." *Science* 279 (5351): 692–95. https://doi.org/10.1126/science.279.5351.692.

Fleitmann, Dominik, Stephen J. Burns, Augusto Mangini, Manfred Mudelsee, Jan Kramers, Igor Villa, Ulrich Neff, et al. 2007. "Holocene ITCZ and Indian Monsoon Dynamics Recorded in Stalagmites from Oman and Yemen (Socotra)." *Quaternary Science Reviews* 26 (1-2): 170–88. https://doi.org/10.1016/j.quascirev.2006.04.012.

Fletcher, William J., and Maria Fernanda S'anchez Goñi. 2008. "Orbital- and Sub-Orbital-Scale Climate Impacts on Vegetation of the Western Mediterranean Basin over the Last 48,000 Yr." *Quaternary Research* 70 (3): 451–64. https://doi.org/10.1016/j.yqres.2008.07.002.

Fohlmeister, Jens, Nicole Vollweiler, Christoph Spötl, and Augusto Mangini. 2012. "COMNISPA II: Update of a Mid-European Isotope Climate Record, 11 Ka to Present." *The Holocene* 23 (5): 749–54. https://doi.org/10.1177/0959683612465446.

Fohlmeister, J., A. Schröder-Ritzrau, D. Scholz, C. Spötl, D. F. C. Riechelmann, M. Mudelsee, A. Wackerbarth, et al. 2012. "Bunker Cave Stalagmites: An Archive for Central European Holocene Climate Variability." *Climate of the Past* 8 (5): 1751–64. https://doi.org/10.5194/cp-8-1751-2012.

Fortin, Marie-Claude, and Konrad Gajewski. 2010. "Postglacial Environmental History of Western Victoria Island, Canadian Arctic." *Quaternary Science Reviews* 29 (17-18): 2099–2110. https://doi.org/10.1016/j.quascirev.2010.05.004.

———. 2016. "Multiproxy Paleoecological Evidence of Holocene Climatic Changes on the Boothia Peninsula, Canadian Arctic." *Quaternary Research* 85 (3): 347–57. https://doi.org/10.1016/j.yqres.2016.02.003.

Fortin, Marie-Claude, Andrew S. Medeiros, Konrad Gajewski, Erin M. Barley, Isabelle Larocque-Tobler, David F. Porinchu, and Sue E. Wilson. 2015. "Chironomid-Environment Relations in Northern North America." *Journal of Paleolimnology* 54 (2-3): 223–37. https://doi.org/10.1007/s10933-015-9848-0.

Fossitt, J. A. 1994. "Late-Glacial and Holocene Vegetation History of Western Donegal, Ireland." *Biology and Environment: Proceedings of the Royal Irish Academy*.

FOSSITT, J. A. 1996. "Late Quaternary Vegetation History of the Western Isles of Scotland." *New Phytologist* 132 (1): 171–96. https://doi.org/10.1111/j.1469-8137.1996.tb04522.x.

Foster, David R., Susan Clayden, David A. Orwig, Brian Hall, and Sylvia Barry. 2002. "Oak, Chestnut and Fire: Climatic and Cultural Controls of Long-Term Forest Dynamics in New England, USA." *Journal of Biogeography* 29 (10-11): 1359–79. https://doi.org/10.1046/j.1365-2699.2002.00760.x.

Foster, Louise C., Emma J. Pearson, Steve Juggins, Dominic A. Hodgson, Krystyna M. Saunders, Elie Verleyen, and Stephen J. Roberts. 2016. "Development of a Regional Glycerol Dialkyl Glycerol Tetraether (GDGT)-Temperature Calibration for Antarctic and Sub-Antarctic Lakes." *Earth and Planetary Science Letters* 433 (January): 370–79. https://doi.org/10.1016/j.epsl.2015.11.018.

Fowell, Sarah J., Barbara C. S. Hansen, John A. Peck, P. Khosbayar, and Enebish Ganbold. 2003. "Mid to Late Holocene Climate Evolution of the Lake Telmen Basin, North Central Mongolia, Based on Palynological Data." *Quaternary Research* 59 (3): 353–63. https://doi.org/10.1016/s0033-5894(02)00020-0.

Fraser, Nicholas, Wolfgang Kuhnt, Ann Holbourn, Timoth'e Bolliet, Nils Andersen, Thomas Blanz, and Luc Beaufort. 2014. "Precipitation Variability Within the West Pacific Warm Pool over the Past 120ka: Evidence from the Davao Gulf, Southern Philippines." *Paleoceanography* 29 (11): 1094–1110. https://doi.org/10.1002/2013pa002599.

Friddell, Julie E., Robert C. Thunell, and Linda E. Heusser. 2002. "Direct Comparison of Marine and Terrestrial Climate Variability During Marine Isotope Stages 6 and 5: Results from Santa Barbara Basin ODP Hole 893A." *Paleoceanography* 17 (1). https://doi.org/10.1029/2000pa000594.

Friel, Charlotte E, Sarah A Finkelstein, and Anthony M Davis. 2014. "Relative Importance of Hydrological and Climatic Controls on Holocene Paleoenvironments Inferred Using Diatom and Pollen Records from a Lake in the Central Hudson Bay Lowlands, Canada." *The Holocene* 24 (3): 295–306. https://doi.org/10.1177/0959683613518587.

Fudge, T. J., Bradley R. Markle, Kurt M. Cuffey, Christo Buizert, Kendrick C. Taylor, Eric J. Steig, Edwin D. Waddington, Howard Conway, and Michelle Koutnik. 2016. "Variable Relationship Between Accumulation and Temperature in West Antarctica for the Past 31,000 Years." *Geophysical Research Letters* 43 (8): 3795–3803. https://doi.org/10.1002/2016gl068356.

Fuller, J. L. 1995. "Holocene Forest Dynamics in Southern Ontario, Canada." *Doctoral Dissertation. University of Cambridge*.

Fuller, Janice L. 1997. "Holocene Forest Dynamics in Southern Ontario, Canada: Fine-Resolution Pollen Data." *Canadian Journal of Botany* 75 (10): 1714–27. https://doi.org/10.1139/b97-886.

———. 1998. "Ecological Impact of the Mid-Holocene Hemlock Decline in Southern Ontario, Canada." *Ecology* 79 (7): 2337–51. https://doi.org/10.1890/0012-9658(1998)079[2337:eiotmh]2.0.co;2.

———. 2016. "ECOLOGICAL IMPACT OF THE MID-HOLOCENE HEMLOCK DECLINE IN SOUTHERN ONTARIO, CANADA." *Ecology* 79.

Fuller, Janice L., David R. Foster, Jason S. McLachlan, and Natalie Drake. 1998. “Impact of Human Activity on Regional Forest Composition and Dynamics in Central New England.” *Ecosystems* 1 (1): 76–95. https://doi.org/10.1007/s100219900007.

Fyfe, R. M., A. G. Brown, and B. J. Coles. 2003. “Mesolithic to Bronze Age Vegetation Change and Human Activity in the Exe Valley, Devon, UK.” *Proceedings of the Prehistoric Society* 69: 161–81. https://doi.org/10.1017/s0079497x00001298.

Fyfe, R. M., A. G. Brown, and S. J. Rippon. 2003. “Mid- to Late-Holocene Vegetation History of Greater Exmoor, UK: Estimating the Spatial Extent of Human-Induced Vegetation Change.” *Vegetation History and Archaeobotany* 12 (4): 215–32. https://doi.org/10.1007/s00334-003-0018-3.

Fyfe, R. M., J. Brück, R. Johnston, H. Lewis, T. P. Roland, and H. Wickstead. 2008. “Historical Context and Chronology of Bronze Age Land Enclosure on Dartmoor, UK.” *Journal of Archaeological Science* 35 (8): 2250–61. https://doi.org/10.1016/j.jas.2008.02.007.

Fyfe, R. M., and J. Woodbridge. 2012. “Differences in Time and Space in Vegetation Patterning: Analysis of Pollen Data from Dartmoor, UK.” *Landscape Ecology*.

Fyfe, Ralph. 2007. “The Importance of Local-Scale Openness Within Regions Dominated by Closed Woodland.” *Journal of Quaternary Science* 22 (6): 571–78. https://doi.org/10.1002/jqs.1078.

Fyfe, Ralph M. 2012. “Bronze Age Landscape Dynamics: Spatially Detailed Pollen Analysis from a Ceremonial Complex.” *Journal of Archaeological Science* 39 (8): 2764–73. https://doi.org/10.1016/j.jas.2012.04.015.

Fyfe, Ralph M., Anthony G. Brown, and Stephen J. Rippon. 2004. “Characterising the Late Prehistoric, ‘Romano-British’ and Medieval Landscape, and Dating the Emergence of a Regionally Distinct Agricultural System in South West Britain.” *Journal of Archaeological Science* 31 (12): 1699–1714. https://doi.org/10.1016/j.jas.2004.05.003.

Gandouin, E. 2002. “Enregistrement Paleoclimatique de La Transgression Holocene: Signature Paleo-Environnementale Des Chironomidae (Dipteres) Du Bassin de Saint-Omer (France).” *Doctoral Dissertation. Universite de Lille.*

Gao, Wenhua, Hainan Hu, Weidong Hou, Pengjia Zhang, Panpan Gong, Wenyan Jia, Xiaoli Liu, and Kaifeng Li. 2022. “The Spatiotemporal Patterns of Human Settlement During the Longshan and Erlitou Periods in Relation to Extreme Floods and Subsistence Strategy in the Upper and Middle Qin River Reaches, Central China.” *Land* 11 (7): 1088. https://doi.org/10.3390/land11071088.

Garcin, Yannick, Daniel Melnick, Manfred R. Strecker, Daniel Olago, and Jean-Jacques Tiercelin. 2012a. “East African Mid-Holocene Wet-Dry Transition Recorded in Palaeo-Shorelines of Lake Turkana, Northern Kenya Rift.” *Earth and Planetary Science Letters* 331-332 (May): 322–34. https://doi.org/10.1016/j.epsl.2012.03.016.

———. 2012b. “East African Mid-Holocene Wet–Dry Transition Recorded in Palaeo-Shorelines of Lake Turkana, Northern Kenya Rift.” *Earth and Planetary Science Letters* 331–332 (May): 322–34. https://doi.org/10.1016/j.epsl.2012.03.016.

Garcin, Yannick, Annie Vincens, David Williamson, Joël Guiot, and Guillaume Buchet. 2006. “Wet Phases in Tropical Southern Africa During the Last Glacial Period.” *Geophysical Research Letters* 33 (7). https://doi.org/10.1029/2005gl025531.

Garralla, S. 1991. “L’analyse Pollinique Des Sediments d’un Lac de La Region de Chibougamau, Quebec.” *Master’s Thesis. Universite Laval.*

Garralla, Silvina, and K. Gajewski. 1992. “Holocene Vegetation History of the Boreal Forest Near Chibougamau, Central Quebec.” *Canadian Journal of Botany* 70 (7): 1364–68. https://doi.org/10.1139/b92-171.

Gauthier, E. 2001. “Evolution de l’impact de l’homme Sur La Vegetation Du Massif Jurassien Au Cours Des Quatre Derniers Millenaires: Nouvelles Donnees Palynologiques.” *Doctoral Dissertation. Universite de Franche-Comte.*

Gavin, Daniel G., Linda B. Brubaker, and D. Noah Greenwald. 2013. “Postglacial Climate and Fire-Mediated Vegetation Change on the Western Olympic Peninsula, Washington (USA).” *Ecological Monographs* 83 (4): 471–89. https://doi.org/10.1890/12-1742.1.

Gavin, Daniel G., Andrew C. G. Henderson, Karlyn S. Westover, Sherilyn C. Fritz, Ian R. Walker, Melanie J. Leng, and Feng Sheng Hu. 2011. “Abrupt Holocene Climate Change and Potential Response to Solar Forcing in Western Canada.” *Quaternary Science Reviews* 30 (9-10): 1243–55. https://doi.org/10.1016/j.quascirev.2011.03.003.

Gavin, Daniel G., Feng Sheng Hu, Kenneth Lertzman, and Peter Corbett. 2006. “Weak Climatic Control of Stand-Scale Fire History During the Late Holocene.” *Ecology* 87 (7): 1722–32. https://doi.org/10.1890/0012-9658(2006)87[1722:wccosf]2.0.co;2.

Geirsd'ottir, 'Aslaug, Gifford H. Miller, John T. Andrews, David J. Harning, Leif S. Anderson, Christopher Florian, Darren J. Larsen, and Thor Thordarson. 2019. "The Onset of Neoglaciation in Iceland and the 4.2 Ka Event." *Climate of the Past* 15 (1): 25–40. https://doi.org/10.5194/cp-15-25-2019.

Geirsd'ottir, 'Aslaug, Gifford H. Miller, Darren J. Larsen, and Sædlafsd'ottir. 2013. "Abrupt Holocene Climate Transitions in the Northern North Atlantic Region Recorded by Synchronized Lacustrine Records in Iceland." *Quaternary Science Reviews* 70 (June): 48–62. https://doi.org/10.1016/j.quascirev.2013.03.010.

Geiss, C. E, C. E Umbanhowar, P. Camill, and S. K. Banerjee. 2003. "Sediment Magnetic Properties Reveal Holocene Climate Change Along the Minnesota Prairie-Forest Ecotone." *Journal of Paleolimnology*.

Gennett, J. A. 1977. "Palynology and Paleoecology of Sediments from Blacktail Pond, Northern Yellowstone Park, Wyoming." *Master's Thesis. University of Iowa*.

Gibb, Olivia T, Sarah Steinhauer, Bianca Fr'echette, Anne de Vernal, and Claude Hillaire-Marcel. 2015. "Diachronous Evolution of Sea Surface Conditions in the Labrador Sea and Baffin Bay Since the Last Deglaciation." *The Holocene* 25 (12): 1882–97. https://doi.org/10.1177/0959683615591352.

Gibbons, Fern T., Delia W. Oppo, Mahyar Mohtadi, Yair Rosenthal, Jun Cheng, Zhengyu Liu, and Braddock K. Linsley. 2014. "Deglacial d18O and Hydrologic Variability in the Tropical Pacific and Indian Oceans." *Earth and Planetary Science Letters* 387 (February): 240–51. https://doi.org/10.1016/j.epsl.2013.11.032.

Giesecke, T. 2001. "Pollenanalytische Und Sedimentchemische Untersuchungen Zur Naturlichen Und Anthropogenen Geschichte Im Schlaubetal." *Sitzungsberichte Der Gesellschaft Naturforschender Freunde Zu Berlin*.

Giesecke, T., A. E. Bjune, R. C. Chiverrell, H. Seppä, A. E. K. Ojala, and H. J. B. Birks. 2008. "Exploring Holocene Continentality Changes in Fennoscandia Using Present and Past Tree Distributions." *Quaternary Science Reviews* 27 (13-14): 1296–1308. https://doi.org/10.1016/j.quascirev.2008.03.008.

Giesecke, Thomas. 2005a. "Holocene Forest Development in the Central Scandes Mountains, Sweden." *Vegetation History and Archaeobotany* 14 (2): 133–47. https://doi.org/10.1007/s00334-005-0070-2.

———. 2005b. "Holocene Dynamics of the Southern Boreal Forest in Sweden." *The Holocene* 15 (6): 858–72. https://doi.org/10.1191/0959683605hl859ra.

Giraudeau, Jacques, Michel Cremer, Sandrine Manth'e, Laurent Labeyrie, and Gerard Bond. 2000. "Coccolith Evidence for Instabilities in Surface Circulation South of Iceland During Holocene Times." *Earth and Planetary Science Letters* 179 (2): 257–68. https://doi.org/10.1016/s0012-821x(00)00113-8.

Gkinis, V., S. B. Simonsen, S. L. Buchardt, J. W. C. White, and B. M. Vinther. 2014a. "Water Isotope Diffusion Rates from the NorthGRIP Ice Core for the Last 16,000 Years - Glaciological and Paleoclimatic Implications." *Earth and Planetary Science Letters* 405 (November): 132–41. https://doi.org/10.1016/j.epsl.2014.08.022.

———. 2014b. "Water Isotope Diffusion Rates from the NorthGRIP Ice Core for the Last 16,000 Years – Glaciological and Paleoclimatic Implications." *Earth and Planetary Science Letters* 405 (November): 132–41. https://doi.org/10.1016/j.epsl.2014.08.022.

Gobet, Erika, Willy Tinner, Christian Bigler, Peter A. Hochuli, and Brigitta Ammann. 2005. "Early-Holocene Afforestation Processes in the Lower Subalpine Belt of the Central Swiss Alps as Inferred from Macrofossil and Pollen Records." *The Holocene* 15 (5): 672–86. https://doi.org/10.1191/0959683605hl843rp.

Gobet, E., W. Tinner, P. Hubschmid, I. Jansen, M. Wehrli, B. Ammann, and L. Wick. 2000. "Influence of Human Impact and Bedrock Differences on the Vegetational History of the Insubrian Southern Alps." *Vegetation History and Archaeobotany* 9 (3): 175–87. https://doi.org/10.1007/bf01299802.

Goldsmith, Yonaton, Wallace S. Broecker, Hai Xu, Pratigya J. Polissar, Peter B. deMenocal, Naomi Porat, Jianghu Lan, Peng Cheng, Weijian Zhou, and Zhisheng An. 2017. "Northward Extent of East Asian Monsoon Covaries with Intensity on Orbital and Millennial Timescales." *Proceedings of the National Academy of Sciences* 114 (8): 1817–21. https://doi.org/10.1073/pnas.1616708114.

Goman, Michelle, Arthur Joyce, Steve Lund, Charlotte Pearson, William Guerra, Darren Dale, Douglas E. Hammond, and Aaron J. Celestian. 2018a. "Preliminary Results from Laguna Minucúa: A Potentially Annually Resolved Record of Climate and Environmental Change for the Past ~5000 Years in the Mixteca Alta of Oaxaca, Mexico." *Quaternary International* 469 (March): 85–95. https://doi.org/10.1016/j.quaint.2017.01.027.

———. 2018b. "Preliminary Results from Laguna Minucúa: A Potentially Annually Resolved Record of Climate and Environmental Change for the Past ~5000 Years in the Mixteca Alta of Oaxaca, Mexico." *Quaternary International* 469 (March): 85–95. https://doi.org/10.1016/j.quaint.2017.01.027.

Gonzales, Leila M., and Eric C. Grimm. 2009. "Synchronization of Late-Glacial Vegetation Changes at Crystal Lake, Illinois, USA with the North Atlantic Event Stratigraphy." *Quaternary Research* 72 (2): 234–45. https://doi.org/10.1016/j.yqres.2009.05.001.

Goransson, H. 1977. "The Flandrian Vegetational History of Southern Ostergotland." *Lundqua Thesis*.

———. 1989. "Dags Mosse: Ostergotlands Forhistoriska Kalender." *Svensk Botanisk Tidskrift*.

———. 1991. "Vegetation and Man Around Lake Bjarsjoholmssjon During Prehistoric Time." *Unknown*.

Goudeau, Marie-Louise S., Anna-Lena Grauel, Chiara Tessarolo, Arne Leider, Liang Chen, Stefano M. Bernasconi, Gerard J. M. Versteegh, et al. 2014a. "The Glacial-Interglacial Transition and Holocene Environmental Changes in Sediments from the Gulf of Taranto, Central Mediterranean." *Marine Geology* 348 (February): 88–102. https://doi.org/10.1016/j.margeo.2013.12.003.

———, et al. 2014b. "The Glacial–Interglacial Transition and Holocene Environmental Changes in Sediments from the Gulf of Taranto, Central Mediterranean." *Marine Geology* 348 (February): 88–102. https://doi.org/10.1016/j.margeo.2013.12.003.

Gouramanis, C., J. Dodson, D. Wilkins, P. De Deckker, and B. M. Chase. 2012. "Holocene Palaeoclimate and Sea Level Fluctuation Recorded from the Coastal Barker Swamp, Rottnest Island, South-Western Western Australia." *Quaternary Science Reviews* 54 (October): 40–57. https://doi.org/10.1016/j.quascirev.2012.05.007.

Gouramanis, Chris, Daniel Wilkins, and Patrick De Deckker. 2010. "6000years of Environmental Changes Recorded in Blue Lake, South Australia, Based on Ostracod Ecology and Valve Chemistry." *Palaeogeography, Palaeoclimatology, Palaeoecology* 297 (1): 223–37. https://doi.org/10.1016/j.palaeo.2010.08.005.

Grachev, Alexei M, Elena Y Novenko, Evgeniy A Grabenko, Mikhail Y Alexandrin, Elya P Zazovskaya, Evgeniy A Konstantinov, Vasiliy A Shishkov, et al. 2020. "The Holocene Paleoenvironmental History of Western Caucasus (Russia) Reconstructed by Multi-Proxy Analysis of the Continuous Sediment Sequence from Lake Khuko." *The Holocene* 31 (3): 368–79. https://doi.org/10.1177/0959683620972782.

Grant, K. M., E. J. Rohling, M. Bar-Matthews, A. Ayalon, M. Medina-Elizalde, C. Bronk Ramsey, C. Satow, and A. P. Roberts. 2012a. "Rapid Coupling Between Ice Volume and Polar Temperature over the Past 150,000 Years." *Nature* 491 (7426): 744–47. https://doi.org/10.1038/nature11593.

———. 2012b. "Rapid Coupling Between Ice Volume and Polar Temperature over the Past 150,000years." *Nature* 491 (7426): 744–47. https://doi.org/10.1038/nature11593.

Grant, M. J, K. E. Barber, and P. D. M. Hughes. 2009. "Early to Mid-Holocene Vegetation-Fire Interactions and Responses to Climatic Change at Cranes Moor, New Forest." *In: The Quaternary of the Solent Basin and West Sussex Raised Beaches Ed by M.R.B.R.T.H.a.F.F.W.-S. R.M. Briant (Pp.198-214). Quaternary Research Association*.

Griffiths, M. L., R. N. Drysdale, M. K. Gagan, J.-x. Zhao, L. K. Ayliffe, J. C. Hellstrom, W. S. Hantoro, et al. 2009. "Increasing Australian–Indonesian Monsoon Rainfall Linked to Early Holocene Sea-Level Rise." *Nature Geoscience* 2 (9): 636–39. https://doi.org/10.1038/ngeo605.

Grindean, Roxana, and Ioan Tanţău, Sorina Fărcaş, Andrei Panait, and and and. 2014. "Middle to Late Holocene Vegetation Shifts in the NW Transylvanian Lowlands (Romania)." *Studia Universitatis Babes-Bolyai, Geologia* 59 (1-2): 29–37. https://doi.org/10.5038/1937-8602.59.1.2.

Groucutt, Huw S., W. Christopher Carleton, Katrin Fenech, Ritienne Gauci, Reuben Grima, Eleanor M. L. Scerri, Mathew Stewart, and Nicholas C. Vella. 2022. "The 4.2 Ka Event and the End of the Maltese 'Temple Period'." *Frontiers in Earth Science* 9 (January). https://doi.org/10.3389/feart.2021.771683.

Grousset, Francis E., Claude Pujol, Laurent Labeyrie, G'erard Auffret, and An Boelaert. 2000. "Were the North Atlantic Heinrich Events Triggered by the Behavior of the European Ice Sheets?" *Geology* 28 (2): 123. https://doi.org/10.1130/0091-7613(2000)28<123:wtnahe>2.0.co;2.

Grudd, Håkan, Keith R. Briffa, Wibjorn Karl'en, Thomas S. Bartholin, Philip D. Jones, and Bernd Kromer. 2002. "A 7400-Year Tree-Ring Chronology in Northern Swedish Lapland: Natural Climatic Variability Expressed on Annual to Millennial Timescales." *The Holocene* 12 (6): 657–65. https://doi.org/10.1191/0959683602hl578rp.

Gu'edron, St'ephane, Christophe Delaere, Sherilyn. C. Fritz, Julie Tolu, Pierre Sabatier, Anne-Lise Devel, Carlos Heredia, Claire V'erin, Eduardo Q. Alves, and Paul A. Baker. 2023. "Holocene Variations in Lake Titicaca Water Level and Their Implications for Sociopolitical Developments in the Central Andes." *Proceedings of the National Academy of Sciences* 120 (2). https://doi.org/10.1073/pnas.2215882120.

Guenet, P. 1986. "Analyse Pollinique de La Tourbiere de Chambedaze Et Recherches Pollenanalytiques Dans Les Monts Dore Et Le Cezallier, Massif Central, France." *Doctoral Dissertation. Aix-Marseille Universite*.

———. 1993. “Analyses Pollenanalytique En Artense Et Sur Le Plateau de Millevaches (Massif Central, France).” *Palynosciences*.
Guo, S. Q, Y Shi, L. J Yang, J. D Cao, W. Q Zhang, J Zhang, F. Lin, and JX. He. 1999. “A Study of Paleoclimatic Fluctuations in the Last 2600 Years in the Ningjin Lake Area, Hebei Province.” *Journal of Geomechanics*.
Hadorn, P. 1992. “Vegetationsgeschichtliche Studie Am Nordufer Des Lac de Neuchatel - Pollenanalytische Untersuchungen Im Loclat, in Der Bucht von Hauterive/Saint- Blaise Und in Den Neolithischen Ufersiedlungen von Saint- Blaise/Bains Des Dames.” *Unknown*.
Hajdas, Irka, and Adam Michczy’nski. 2010. “Age-Depth Model of Lake Soppensee (Switzerland) Based on the High-Resolution ¹⁴c Chronology Compared with Varve Chronology.” *Radiocarbon* 52 (3): 1027–40. https://doi.org/10.1017/s0033822200046117.
Hallett, Douglas J., and Leonard V. Hills. 2006. “Holocene Vegetation Dynamics, Fire History, Lake Level and Climate Change in the Kootenay Valley, Southeastern British Columbia, Canada.” *Journal of Paleolimnology* 35 (2): 351–71. https://doi.org/10.1007/s10933-005-1335-6.
Hammarlund, D. 2003. “Rapid Hydrological Changes During the Holocene Revealed by Stable Isotope Records of Lacustrine Carbonates from Lake Igelsjön, Southern Sweden.” *Quaternary Science Reviews* 22 (2-4): 353–70. https://doi.org/10.1016/s0277-3791(02)00091-4.
Han, H. Y, S. F Li, L. X Zhang, S Zhou, J. X Zhang, S. G. Wu, and F. B. Wang. 2000. “Study on Pollen Analysis and Palaeoenvironments in Baohuashan Mt.” *Of Jurong County*.
Harada, Naomi, Naokazu Ahagon, Tatsuhiko Sakamoto, Masao Uchida, Minoru Ikehara, and Yasuyuki Shibata. 2006. “Rapid Fluctuation of Alkenone Temperature in the Southwestern Okhotsk Sea During the Past 120 Ky.” *Global and Planetary Change* 53 (1–2): 29–46. https://doi.org/10.1016/j.gloplacha.2006.01.010.
Harada, Naomi, Naokazu Ahagon, Masao Uchida, and Masafumi Murayama. 2004. “Northward and Southward Migrations of Frontal Zones During the Past 40 Kyr in the Kuroshio-Oyashio Transition Area.” *Geochemistry, Geophysics, Geosystems* 5 (9): n/a–. https://doi.org/10.1029/2004gc000740.
Harbert, Robert S., and Kevin C. Nixon. 2018. “Quantitative Late Quaternary Climate Reconstruction from Plant Macrofossil Communities in Western North America.” *Open Quaternary* 4 (1): 8. https://doi.org/10.5334/oq.46.
Harning, David J., ’Aslaug Geirsd’ottir, and Gifford H. Miller. 2018. “Punctuated Holocene Climate of Vestfirir, Iceland, Linked to Internal/External Variables and Oceanographic Conditions.” *Quaternary Science Reviews* 189 (June): 31–42. https://doi.org/10.1016/j.quascirev.2018.04.009.
Haslett, J., M. Whiley, S. Bhattacharya, M. Salter-Townshend, Simon P. Wilson, J. R. M. Allen, B. Huntley, and F. J. G. Mitchell. 2006. “Bayesian Palaeoclimate Reconstruction.” *Journal of the Royal Statistical Society Series A: Statistics in Society* 169 (3): 395–438. https://doi.org/10.1111/j.1467-985x.2006.00429.x.
Hathorn, J. H. 2008. “Paleoecology and Paleotempestology of the Pascagoula Marsh, Mississippi.” *Master’s Thesis. Louisiana State University*.
Haug, Gerald H., Konrad A. Hughen, Daniel M. Sigman, Larry C. Peterson, and Ursula RoNANAh. 2001. “Southward Migration of the Intertropical Convergence Zone Through the Holocene.” *Science* 293 (5533): 1304–8. https://doi.org/10.1126/science.1059725.
Hausmann, Sonja, Isabelle Larocque-Tobler, Pierre J. H. Richard, Reinhard Pienitz, Guillaume St-Onge, and Falko Fye. 2011. “Diatom-Inferred Wind Activity at Lac Du Sommet, Southern Quebec, Canada: A Multiproxy Paleoclimate Reconstruction Based on Diatoms, Chironomids and Pollen for the Past 9500 Years.” *The Holocene* 21 (6): 925–38. https://doi.org/10.1177/0959683611400199.
Hazell, Calian J., Matthew J. Pound, and Emma P. Hocking. 2022a. “Response of the Akrotiri Marsh, Island of Cyprus, to Bronze Age Climate Change.” *Palaeogeography, Palaeoclimatology, Palaeoecology* 587 (February): 110788. https://doi.org/10.1016/j.palaeo.2021.110788.
———. 2022b. “High-Resolution Bronze Age Palaeoenvironmental Change in the Eastern Mediterranean: Exploring the Links Between Climate and Societies.” *Palynology* 46 (4): 1–20. https://doi.org/10.1080/01916122.2022.2067259.
He, Keyang, Houyuan Lu, Guiyun Jin, Can Wang, Hai Zhang, Jianping Zhang, Deke Xu, Caiming Shen, Naiqin Wu, and Zhengtang Guo. 2022. “Antipodal Pattern of Millet and Rice Demography in Response to 4.2 Ka Climate Event in China.” *Quaternary Science Reviews* 295 (November): 107786. https://doi.org/10.1016/j.quascirev.2022.107786.
Head, Martin J. 2019. “Formal Subdivision of the Quaternary System/Period: Present Status and Future Directions.” *Quaternary International* 500 (January): 32–51. https://doi.org/10.1016/j.quaint.2019.05.018.
Heery, A. 1998. “The Vegetation History of the Irish Midlands: Palaeoecological Reconstructions of Two Lake Sites Adjacent to Eskers.” *Doctoral Dissertation. University of Dublin*.

Heikkilä, Maija, Thomas W. D. Edwards, Heikki Seppä, and Eloni Sonninen. 2010. “Sediment Isotope Tracers from Lake Saarikko, Finland, and Implications for Holocene Hydroclimatology.” *Quaternary Science Reviews* 29 (17-18): 2146–60. https://doi.org/10.1016/j.quascirev.2010.05.010.

Heikkilä, Maija, and Heikki Seppä. 2003. “A 11,000yr Palaeotemperature Reconstruction from the Southern Boreal Zone in Finland.” *Quaternary Science Reviews* 22 (5–7): 541–54. https://doi.org/10.1016/s0277-3791(02)00189-0.

Heinrichs, M. L. 1999. “A Late-Quaternary Paleoecological Analysis in the Engelmann Spruce-Subalpine Fir Biogeoclimatic Zone of the Okanagan/Ashnola Region, British Columbia, Canada.” *Doctoral Dissertation. University of Victoria*.

Heinsalu, A, T Martma, R Rajamäe, L Saarse, and S Veski. 1995. “Litho- and Biostratigraphy of Lake Paidre, South Estonia.” *Proceedings of the Estonian Academy of Sciences. Geology* 44 (1): 45. https://doi.org/10.3176/geol.1995.1.05.

Helama, Samuli, and Markku Oinonen. 2019. “Exact Dating of the Meghalayan Lower Boundary Based on High-Latitude Tree-Ring Isotope Chronology.” *Quaternary Science Reviews* 214 (June): 178–84. https://doi.org/10.1016/j.quascirev.2019.04.013.

Helama, S., M. M. Fauria, K. Mielikainen, M. Timonen, and M. Eronen. 2010. “Sub-Milankovitch Solar Forcing of Past Climates: Mid and Late Holocene Perspectives.” *Geological Society of America Bulletin* 122 (11–12): 1981–88. https://doi.org/10.1130/b30088.1.

Hennekam, Rick, Bregje Bolt, Egbert H. Nes, Gert J. Lange, Marten Scheffer, and Gert-Jan Reichart. 2020. “Early-Warning Signals for Marine Anoxic Events.” *Geophysical Research Letters* 47 (20). https://doi.org/10.1029/2020gl089183.

Hennekam, Rick, Timme H. Donders, Karin Zwiep, and Gert J. de Lange. 2015. “Integral View of Holocene Precipitation and Vegetation Changes in the Nile Catchment Area as Inferred from Its Delta Sediments.” *Quaternary Science Reviews* 130 (December): 189–99. https://doi.org/10.1016/j.quascirev.2015.05.031.

Hennekam, Rick, Tom Jilbert, Bernhard Schnetger, and Gert J. de Lange. 2014. “Solar Forcing of Nile Discharge and Sapropel S1 Formation in the Early to Middle Holocene Eastern Mediterranean.” *Paleoceanography* 29 (5): 343–56. https://doi.org/10.1002/2013pa002553.

Herzschuh, Ulrike, Thomas Böhmer, Chenzhi Li, Manuel Chevalier, Anne Dallmeyer, Xianyong Cao, Nancy H. Bigelow, et al. 2022. “LegacyClimate 1.0: A Dataset of Pollen-Based Climate Reconstructions from 2594 Northern Hemisphere Sites Covering the Late Quaternary.” *Earth System Science Data*. Copernicus GmbH. https://doi.org/10.5194/essd-2022-38.

Herzschuh, Ulrike, Thomas Böhmer, Chenzhi Li, Manuel Chevalier, Raphaël H’ebert, Anne Dallmeyer, Xianyong Cao, et al. 2023. “LegacyClimate 1.0: A Dataset of Pollen-Based Climate Reconstructions from 2594 Northern Hemisphere Sites Covering the Last 30 Kyr and Beyond.” *Earth System Science Data* 15 (6): 2235–58. https://doi.org/10.5194/essd-15-2235-2023.

Herzschuh, Ulrike, Annette Kramer, Steffen Mischke, and Chengjun Zhang. 2009. “Quantitative Climate and Vegetation Trends Since the Late Glacial on the Northeastern Tibetan Plateau Deduced from Koucha Lake Pollen Spectra.” *Quaternary Research* 71 (2): 162–71. https://doi.org/10.1016/j.yqres.2008.09.003.

Herzschuh, Ulrike, Katja Winter, Bernd Wünnemann, and Shijie Li. 2006. “A General Cooling Trend on the Central Tibetan Plateau Throughout the Holocene Recorded by the Lake Zigetang Pollen Spectra.” *Quaternary International* 154-155 (October): 113–21. https://doi.org/10.1016/j.quaint.2006.02.005.

Hildebrandt, H, B Heuser-Hildebrandt, S. Wolters, and M. Dormos. 2007. “Kulturlandschaftsgenetische Und Bestandsgeschichtliche Untersuchungen Anhand von Kohlholzspektren Aus Historischen Meilerplatzen, Pollendiagrammen Und Archivalischen Quellen Im Naturpark Pfalzerwald, Forstamt Johanniskreuz.” *Unknown*.

Hildebrandt, W. R., and J. F. Hayes. 1983. “Archaeological Investigations on Pilot Ridge Six Rivers National Forest.” *U.S. Forest Service Contract 53-9JHA-2-140*.

Hodell, David A., Jason H. Curtis, and Mark Brenner. 1995. “Possible Role of Climate in the Collapse of Classic Maya Civilization.” *Nature* 375 (6530): 391–94. https://doi.org/10.1038/375391a0.

Hodell, David A., Jason H. Curtis, Glenn A. Jones, Antonia Higuera-Gundy, Mark Brenner, Michael W. Binford, and Kathleen T. Dorsey. 1991. “Reconstruction of Caribbean Climate Change over the Past 10,500 Years.” *Nature* 352 (6338): 790–93. https://doi.org/10.1038/352790a0.

Holland, S. M. 1975. “A Pollen-Analytical Study Concerning Settlement and Early Agriculture in County down, Northern Ireland.” *Doctoral Dissertation. Queen’s University*.

Hong, Y. T., H. B. Jiang, T. S. Liu, L. P. Zhou, J. Beer, H. D. Li, X. T. Leng, B. Hong, and X. G. Qin. 2000a. "Response of Climate to Solar Forcing Recorded in a 6000-yeard18O Time-Series of Chinese Peat Cellulose." *The Holocene* 10 (1): 1–7. https://doi.org/10.1191/095968300669856361.

———. 2000b. "Response of Climate to Solar Forcing Recorded in a 6000-yearδ18O Time-Series of Chinese Peat Cellulose." *The Holocene* 10 (1): 1–7. https://doi.org/10.1191/095968300669856361.

Hong, Y. T., Z. G. Wang, H. B. Jiang, Q. H. Lin, B. Hong, Y. X. Zhu, Y. Wang, L. S. Xu, X. T. Leng, and H. D. Li. 2001a. "A 6000-Year Record of Changes in Drought and Precipitation in Northeastern China Based on a d13C Time Series from Peat Cellulose." *Earth and Planetary Science Letters* 185 (1-2): 111–19. https://doi.org/10.1016/s0012-821x(00)00367-8.

———. 2001b. "A 6000-Year Record of Changes in Drought and Precipitation in Northeastern China Based on a δ13C Time Series from Peat Cellulose." *Earth and Planetary Science Letters* 185 (1–2): 111–19. https://doi.org/10.1016/s0012-821x(00)00367-8.

Hotchkiss, Sara C., Randy Calcote, and Elizabeth A. Lynch. 2007. "Response of Vegetation and Fire to Little Ice Age Climate Change: Regional Continuity and Landscape Heterogeneity." *Landscape Ecology* 22 (S1): 25–41. https://doi.org/10.1007/s10980-007-9133-3.

Hu, Chaoyong, Gideon M. Henderson, Junhua Huang, Shucheng Xie, Ying Sun, and Kathleen R. Johnson. 2008. "Quantification of Holocene Asian Monsoon Rainfall from Spatially Separated Cave Records." *Earth and Planetary Science Letters* 266 (3-4): 221–32. https://doi.org/10.1016/j.epsl.2007.10.015.

Hu, Feng Sheng, Darrell Kaufman, Sumiko Yoneji, David Nelson, Aldo Shemesh, Yongsong Huang, Jian Tian, Gerard Bond, Benjamin Clegg, and Thomas Brown. 2003. "Cyclic Variation and Solar Forcing of Holocene Climate in the Alaskan Subarctic." *Science* 301 (5641): 1890–93. https://doi.org/10.1126/science.1088568.

Hu, Y, X Cao, Z Zhao, Y Li, Y. Sun, and H. Wang. 2016. "The Palaeoenvironmental and Palaeoclimatic Reconstruction and the Relation with the Human Activities During the Early and Middle Holocene in the Upper Western Liao River Region." *Quaternary Sci*.

Hu, Yue, Xiaoming Sun, Hai Cheng, and Hong Yan. 2020. "Evidence from Giant-Clam &Amp;lt;i>δ</i>¹⁸o of Intense El Ninõ–Southern Oscillation-Related Variability but Reduced Frequency 3700 Years Ago." *Climate of the Past* 16 (2): 597–610. https://doi.org/10.5194/cp-16-597-2020.

Huang, C. Q, Z. D Feng, Y. Z Ma, L. L. Guo, and W. Wang. 2009. "Holocene Palaeoenvironment Changes Recorded by Pollen of Baahar Nuur Lake." *Journal of Lanzhou University (Natural Sciences)*.

Huang, Wei, Yongjin Wang, Hai Cheng, Richard Lawrence Edwards, Chuan-Chou Shen, Dianbing Liu, Qingfeng Shao, Chao Deng, Zhenqiu Zhang, and Quan Wang. 2016. "Multi-Scale Holocene Asian Monsoon Variability Deduced from a Twin-Stalagmite Record in Southwestern China." *Quaternary Research* 86 (1): 34–44. https://doi.org/10.1016/j.yqres.2016.05.001.

Huang, Xianyu, Philip A Meyers, Chengling Jia, Min Zheng, Jiantao Xue, Xinxin Wang, and Shucheng Xie. 2013. "Paleotemperature Variability in Central China During the Last 13 Ka Recorded by a Novel Microbial Lipid Proxy in the Dajiuhu Peat Deposit." *The Holocene* 23 (8): 1123–29. https://doi.org/10.1177/0959683613483617.

Huang, Xiaozhong, Wei Peng, Natalia Rudaya, Eric C. Grimm, Xuemei Chen, Xianyong Cao, Jun Zhang, et al. 2018. "Holocene Vegetation and Climate Dynamics in the Altai Mountains and Surrounding Areas." *Geophysical Research Letters* 45 (13): 6628–36. https://doi.org/10.1029/2018gl078028.

Hughes, Malcolm K, and Lisa J Graumlich. 1996. "Multimillennial Dendroclimatic Studies from the Western United States." In *Climatic Variations and Forcing Mechanisms of the Last 2000 Years*, 109–24. Springer Berlin Heidelberg. https://doi.org/10.1007/978-3-642-61113-1_6.

Huguet, Carme, Joyanto Routh, Susanne Fietz, Mahjoor Ahmad Lone, M. S. Kalpana, Prosenjit Ghosh, Augusto Mangini, Vikash Kumar, and Ravi Rangarajan. 2018. "Temperature and Monsoon Tango in a Tropical Stalagmite: Last Glacial-Interglacial Climate Dynamics." *Scientific Reports* 8 (1). https://doi.org/10.1038/s41598-018-23606-w.

Humphries, M. S., K. L. Kirsten, and T. S. McCarthy. 2019. "Rapid Changes in the Hydroclimate of Southeast Africa During the Mid- to Late-Holocene." *Quaternary Science Reviews* 212 (May): 178–86. https://doi.org/10.1016/j.quascirev.2019.04.006.

Ilyashuk, Elena A., Karin A. Koinig, Oliver Heiri, Boris P. Ilyashuk, and Roland Psenner. 2011. "Holocene Temperature Variations at a High-Altitude Site in the Eastern Alps: A Chironomid Record from Schwarzsee Ob Sölden, Austria." *Quaternary Science Reviews* 30 (1-2): 176–91. https://doi.org/10.1016/j.quascirev.2010.10.008.

Innes, James B., Yongqiang Zong, Zhanghua Wang, and Zhongyuan Chen. 2014. "Climatic and Palaeoecological Changes During the Mid- to Late Holocene Transition in Eastern China: High-Resolution Pollen and Non-Pollen Palynomorph Analysis at Pingwang, Yangtze Coastal Lowlands." *Quaternary Science Reviews* 99 (September): 164–75. https://doi.org/10.1016/j.quascirev.2014.06.013.

Isola, Ilaria, Giovanni Zanchetta, Russell N. Drysdale, Eleonora Regattieri, Monica Bini, Petra Bajo, John C. Hellstrom, et al. 2019a. "The 4.2 Ka Event in the Central Mediterranean: New Data from a Corchia Speleothem (Apuan Alps, Central Italy)." *Climate of the Past* 15 (1): 135–51. https://doi.org/10.5194/cp-15-135-2019.

———, et al. 2019b. "The 4.2ka Event in the Central Mediterranean: New Data from a Corchia Speleothem (Apuan Alps, Central Italy)." *Climate of the Past* 15 (1): 135–51. https://doi.org/10.5194/cp-15-135-2019.

Isono, Dai, Masanobu Yamamoto, Tomohisa Irino, Tadamichi Oba, Masafumi Murayama, Toshio Nakamura, and Hodaka Kawahata. 2009. "The 1500-Year Climate Oscillation in the Midlatitude North Pacific During the Holocene." *Geology* 37 (7): 591–94. https://doi.org/10.1130/g25667a.1.

Jackson, Stephen T., Robert K. Booth, Kelly Reeves, Jennifer Jewell Andersen, Thomas A. Minckley, and Rachel A. Jones. 2014. "Inferring Local to Regional Changes in Forest Composition from Holocene Macrofossils and Pollen of a Small Lake in Central Upper Michigan." *Quaternary Science Reviews* 98 (August): 60–73. https://doi.org/10.1016/j.quascirev.2014.05.030.

Jacobson Jr, G. L. 1975. "A Palynological Study of the History and Ecology of White Pine in Minnesota." *Doctoral Dissertation. University of Minnesota*.

Jacobson, Matthew J., Pascal Flohr, Alison Gascoigne, Melanie J. Leng, Aleksey Sadekov, Hai Cheng, R. Lawrence Edwards, Okan Tüysüz, and Dominik Fleitmann. 2021. "Heterogenous Late Holocene Climate in the Eastern Mediterranean—the Kocain Cave Record from SW Turkey." *Geophysical Research Letters* 48 (20). https://doi.org/10.1029/2021gl094733.

Jaeschke, Andrea, Carsten Rühlemann, Helge Arz, Gerrit Heil, and Gerrit Lohmann. 2007. "Coupling of Millennial-Scale Changes in Sea Surface Temperature and Precipitation Off Northeastern Brazil with High-Latitude Climate Shifts During the Last Glacial Period." *Paleoceanography* 22 (4): n/a–. https://doi.org/10.1029/2006pa001391.

Jaffe, Yitzchak Y., Lorenzo Castellano, Gideon Shelach-Lavi, and Roderick B. Campbell. 2020. "Mismatches of Scale in the Application of Paleoclimatic Research to Chinese Archaeology." *Quaternary Research* 99 (August): 14–33. https://doi.org/10.1017/qua.2020.60.

Jahns, S. 1999. "Pollen Analysis Studies at the Grosser Krebssee, East Brandenburg." *Germania*.

———. 2004. "Ein Fruhholozanes Pollendiagramm Aus Dem Tagebau Cottbus-Nord." *Verhandlungen Des Botanischen Vereins von Berlin Und Brandenburg*.

Jahns, Susanne. 2000. "Late-Glacial and Holocene Woodland Dynamics and Land-Use History of the Lower Oder Valley, North-Eastern Germany, Based on Two, AMS14C-Dated, Pollen Profiles." *Vegetation History and Archaeobotany* 9 (2): 111–23. https://doi.org/10.1007/bf01300061.

———. 2001. "On the Late Pleistocene and Holocene History of Vegetation and Human Impact in the Ücker Valley, North-Eastern Germany." *Vegetation History and Archaeobotany* 10 (2): 97–104. https://doi.org/10.1007/pl00006924.

———. 2004. "The Holocene History of Vegetation and Settlement at the Coastal Site of Lake Voulkaria in Acarnania, Western Greece." *Vegetation History and Archaeobotany* 14 (1): 55–66. https://doi.org/10.1007/s00334-004-0053-8.

———. 2006. "Palynological Investigations into the Late Pleistocene and Holocene History of Vegetation and Settlement at the Löddigsee, Mecklenburg, Germany." *Vegetation History and Archaeobotany* 16 (2-3): 157–69. https://doi.org/10.1007/s00334-006-0074-6.

Jalali, Bassem, and Marie-Alexandrine Sicre. 2019. "The 4.2 Ka Event in the Euro-Mediterranean Region—a Study from the MISTRALS/PALEOMEX Program." In *Advances in Science, Technology &Amp; Innovation*, 13–15. Springer International Publishing. https://doi.org/10.1007/978-3-030-01599-2_3.

Jalali, Bassem, Marie-Alexandrine Sicre, Nejib Kallel, Julien Azuara, Nathalie Combourieu-Nebout, Maria-Angela Bassetti, and Vincent Klein. 2017. "High-Resolution Holocene Climate and Hydrological Variability from Two Major Mediterranean Deltas (Nile and Rhone)." *The Holocene* 27 (8): 1158–68. https://doi.org/10.1177/0959683616683258.

Janbu, Aina Dahlø, Øyvind Paasche, and Michael R. Talbot. 2011. "Paleoclimate Changes Inferred from Stable Isotopes and Magnetic Properties of Organic-Rich Lake Sediments in Arctic Norway." *Journal of Paleolimnology* 46 (1): 29–44. https://doi.org/10.1007/s10933-011-9512-2.

Jarriel, Katherine. 2021. “Climate Disaster and the Resilience of Local Maritime Networks: Two Examples from the Aegean Bronze Age.” *Quaternary International* 597 (September): 118–30. https://doi.org/10.1016/j.quaint.2020.08.010.

Jennings, A., J. Andrews, and L. Wilson. 2011. “Holocene Environmental Evolution of the SE Greenland Shelf North and South of the Denmark Strait: Irminger and East Greenland Current Interactions.” *Quaternary Science Reviews* 30 (7-8): 980–98. https://doi.org/10.1016/j.quascirev.2011.01.016.

Jennings, Anne, John Andrews, Christof Pearce, Lindsay Wilson, and Sæd’ıs ’Olfasd’otttir. 2015. “Detrital Carbonate Peaks on the Labrador Shelf, a 13–7ka Template for Freshwater Forcing from the Hudson Strait Outlet of the Laurentide Ice Sheet into the Subpolar Gyre.” *Quaternary Science Reviews* 107 (January): 62–80. https://doi.org/10.1016/j.quascirev.2014.10.022.

Jensen, Allison M., David Fastovich, Ben I. Watson, Jacquelyn L. Gill, Stephen T. Jackson, James M. Russell, Joseph Bevington, et al. 2020. “More Than One Way to Kill a Spruce Forest: The Role of Fire and Climate in the Late-Glacial Termination of Spruce Woodlands Across the Southern Great Lakes.” Edited by Peter Bellingham. *Journal of Ecology* 109 (1): 459–77. https://doi.org/10.1111/1365-2745.13517.

JI, J, J SHEN, W BALSAM, J CHEN, L LIU, and X LIU. 2005a. “Asian Monsoon Oscillations in the Northeastern Qinghai-Tibet Plateau Since the Late Glacial as Interpreted from Visible Reflectance of Qinghai Lake Sediments.” *Earth and Planetary Science Letters* 233 (1-2): 61–70. https://doi.org/10.1016/j.epsl.2005.02.025.

———. 2005b. “Asian Monsoon Oscillations in the Northeastern Qinghai–Tibet Plateau Since the Late Glacial as Interpreted from Visible Reflectance of Qinghai Lake Sediments.” *Earth and Planetary Science Letters* 233 (1–2): 61–70. https://doi.org/10.1016/j.epsl.2005.02.025.

Jia, L, L Yu, and W Zhang. 2007. “Pollen and Paloenvironment Analysis of Holocene in Shanghai Region.” *JOURNAL-EAST CHINA NORMAL UNIVERSITY NATURAL SCIENCE* 2 (132): 23.

Jia, Mingming, Changan Li, Xin Mao, Dai Zhang, Chuanyi Wei, and Hui Liu. 2021. “Climate–Human–Environment Interactions in the Middle Yangtze Basin (Central China) During the Middle Holocene, Based on Pollen and Geochemical Records from the Sanfangwan Site.” *CATENA* 204 (September): 105357. https://doi.org/10.1016/j.catena.2021.105357.

Jia, Xin, Haiming Li, Harry F Lee, Zhen Liu, Yong Lu, Zhujun Hu, Xueqiang Sun, and Zhijun Zhao. 2021. “Agricultural Adaptations to Topography and Climate Changes in Central China During the Mid- to Late-Holocene.” *The Holocene* 31 (11–12): 1705–15. https://doi.org/10.1177/09596836211033201.

Jiang, W. Y., J. Guiot, G. Q. Chu, H. Wu Hand B. Yuan, C. Hatta, and Z. T. Guo. 2010. “An Improved Methodology of the Modern Analogues Technique for Palaeoclimate Reconstruction in Arid and Semi-Arid Regions.” *Boreas* 39 (1): 145–53. https://doi.org/10.1111/j.1502-3885.2009.00115.x.

JIANG, WENYING, JO‘IL GUIOT, GUOQIANG CHU, HAIBIN WU, BAOYIN YUAN, CHRISTINE HATT’E, and ZHENGTANG GUO. 2009. “An Improved Methodology of the Modern Analogues Technique for Palaeoclimate Reconstruction in Arid and Semi-Arid Regions.” *Boreas* 39 (1): 145–53. https://doi.org/10.1111/j.1502-3885.2009.00115.x.

Jiang, XiuYang, YaoQi He, ChuanChou Shen, XingGong Kong, ZhiZhong Li, and YuWei Chang. 2011. “Stalagmite-Inferred Holocene Precipitation in Northern Guizhou Province, China, and Asynchronous Termination of the Climatic Optimum in the Asian Monsoon Territory.” *Chinese Science Bulletin* 57 (7): 795–801. https://doi.org/10.1007/s11434-011-4848-6.

Jiang, Xiuyang, Yaoqi He, Chuan-Chou Shen, Zhizhong Li, and Ke Lin. 2013. “Replicated Stalagmite-Inferred Centennial-to Decadal-Scale Monsoon Precipitation Variability in Southwest China Since the Mid Holocene.” *The Holocene* 23 (6): 841–49. https://doi.org/10.1177/0959683612471986.

Jim’enez-Amat, Patricia, and Rainer Zahn. 2015. “Offset Timing of Climate Oscillations During the Last Two Glacial-Interglacial Transitions Connected with Large-Scale Freshwater Perturbation.” *Paleoceanography* 30 (6): 768–88. https://doi.org/10.1002/2014pa002710.

Jim’enez-Moreno, Gonzalo, and R Scott Anderson. 2012. “Pollen and Macrofossil Evidence of Late Pleistocene and Holocene Treeline Fluctuations from an Alpine Lake in Colorado, USA.” *The Holocene* 23 (1): 68–77. https://doi.org/10.1177/0959683612450199.

Jim’enez-Moreno, Gonzalo, Peter J. Fawcett, and R. Scott Anderson. 2008. “Millennial- and Centennial-Scale Vegetation and Climate Changes During the Late Pleistocene and Holocene from Northern New Mexico (USA).” *Quaternary Science Reviews* 27 (13-14): 1442–52. https://doi.org/10.1016/j.quascirev.2008.04.004.

Jimenez-Moreno, G., R. S. Anderson, V. Atudorei, and J. L. Toney. 2010. “A High-Resolution Record of Climate, Vegetation, and Fire in the Mixed Conifer Forest of Northern Colorado, USA.” *Geological Society of America Bulletin* 123 (1-2): 240–54. https://doi.org/10.1130/b30240.1.

Jo, Kyoung-nam, Sangheon Yi, Jin-Yong Lee, Kyung Sik Woo, Hai Cheng, Lawrence R. Edwards, and Sang-Tae Kim. 2017. “1000-Year Quasi-Periodicity of Weak Monsoon Events in Temperate Northeast Asia Since the Mid-Holocene.” *Scientific Reports* 7 (1). https://doi.org/10.1038/s41598-017-15566-4.

Johnsen, S. J., H. B. Clausen, W. Dansgaard, K. Fuhrer, N. Gundestrup, C. U. Hammer, P. Iversen, J. Jouzel, B. Stauffer, and J. P. steffensen. 1992. “Irregular Glacial Interstadials Recorded in a New Greenland Ice Core.” *Nature* 359 (6393): 311–13. https://doi.org/10.1038/359311a0.

Johnsen, S. J., W. Dansgaard, H. B. Clausen, and C. C. Langway. 1972. “Oxygen Isotope Profiles Through the Antarctic and Greenland Ice Sheets.” *Nature*, 429–34.

Johnsen, Sigfus J., Dorthe Dahl-Jensen, Willi Dansgaard, and Niels Gundestrup. 1995. “Greenland Palaeotemperatures Derived from GRIP Bore Hole Temperature and Ice Core Isotope Profiles.” *Tellus B: Chemical and Physical Meteorology* 47 (5): 624. https://doi.org/10.3402/tellusb.v47i5.16077.

Johnsen, Sigfus J., Dorthe Dahl-Jensen, Niels Gundestrup, Jørgen P. Steffensen, Henrik B. Clausen, Heinz Miller, Valerie Masson-Delmotte, Arny E. Sveinbjörnsdottir, and James White. 2001. “Oxygen Isotope and Palaeotemperature Records from Six Greenland Ice-Core Stations: Camp Century, Dye-3, GRIP, GISP2, Renland and NorthGRIP.” *Journal of Quaternary Science* 16 (4): 299–307. https://doi.org/10.1002/jqs.622.

Joly, C. 2006. “Histoire de La Vegetation Dans l’espace Centre-Ouest Atlantique (France) : Relations Societes/Vegetation Et Evolution Du Trait de Cote Depuis Le Mesolithique Recent-Final.” *Doctoral Dissertation. Universite de Nantes*.

Joly, Camille, Lionel Visset, Camille Scaon, Corinne Pont-Tricoire, and H’el‘ene Froquet-Uzel. 2008. “Archeologie Preventive Et Evolution Du Paysage Vegetal de l’age Du Fer Au Moyen Age Dans Le Gatinais : Mise En Evidence de Culture de Chanvre Et d’activites de Rouissage (Courcelles Et Sceaux-En-Gatinais, Loiret, France).” *ArcheoSciences*, no. 32 (December): 15–30. https://doi.org/10.4000/archeosciences.921.

Joly, C., and L. Visset. 2011. “Etude Paleoenvironnementale de La Grande Pree de Varades (La Boire Torse) Dans Le Bassin Aval de La Loire (Loire-Atlantique, France).” *Revue Archeologique Du Loiret*.

Jones, Matthew D., Sarah E. Metcalfe, Sarah J. Davies, and Anders Noren. 2015. “Late Holocene Climate Reorganisation and the North American Monsoon.” *Quaternary Science Reviews* 124 (September): 290–95. https://doi.org/10.1016/j.quascirev.2015.07.004.

Jones, Matthew D., C. Neil Roberts, Melanie J. Leng, and Murat Türkeş. 2006. “A High-Resolution Late Holocene Lake Isotope Record from Turkey and Links to North Atlantic and Monsoon Climate.” *Geology* 34 (5): 361. https://doi.org/10.1130/g22407.1.

Jones, Miriam C., Christopher E. Bernhardt, Ken W. Krauss, and Gregory B. Noe. 2017. “The Impact of Late Holocene Land Use Change, Climate Variability, and Sea Level Rise on Carbon Storage in Tidal Freshwater Wetlands on the Southeastern United States Coastal Plain.” *Journal of Geophysical Research: Biogeosciences* 122 (12): 3126–41. https://doi.org/10.1002/2017jg004015.

Jordan, S. F., B. T. Murphy, S. S. Oand K. P. Doyle, M. D. Williams, A. Grey, S. Lee, M. V. McCaul, and B. P. Kellehe. 2017. “Mid-Holocene Climate Change and Landscape Formation in Ireland: Evidence from a Geochemical Investigation of a Coastal Peat Bog.” *Organic Geochemistry* 109 (July): 67–76. https://doi.org/10.1016/j.orggeochem.2017.02.004.

Jouffroy-Bapicot, I. 2010. “Evolution de La Vegetation Du Massif Du Morvan (Bourgogne - France) Depuis La Derniere Glaciation a Partir de l’analyse Pollinique.” *Variations Climatiques Et Impact Des Activites Anthropiques. Doctoral Dissertation. Universite de Franche-Comte*.

Jouzel, J., V. Masson-Delmotte, O. Cattani, G. Dreyfus, S. Falourd, G. Hoffmann, B. Minster, et al. 2007. “Orbital and Millennial Antarctic Climate Variability over the Past 800,000 Years.” *Science* 317 (5839): 793–96. https://doi.org/10.1126/science.1141038.

Jung, Simon J. A. 1996. “Wassermassenaustausch Zwischen NE-Atlantik Und Nordmeer w _Hrend Der Letzten 300.000/80.000 Jahre Im Abbild Stabiler 0- Und c-Isotope.” *Berichte Aus Dem Sonderforschungsbereich 313, Ver _Nderungen Der Umwelt - Der Ņrdliche Nordatlantik*, 154.

Justwan, Aur’elie, Nalan Koç, and Anne E. Jennings. 2008. “Evolution of the Irminger and East Icelandic Current Systems Through the Holocene, Revealed by Diatom-Based Sea Surface Temperature Reconstructions.” *Quaternary Science Reviews* 27 (15–16): 1571–82. https://doi.org/10.1016/j.quascirev.2008.05.006.

Kaboth-Bahr, Stefanie, Andr’e Bahr, Kweku A. Yamoah, Chih-Kai Chuang, Hong-Chun Li, Chih-Chieh Su, and Kuo-Yen Wei. 2021a. “Rapid Humidity Changes Across the Northern South China Sea During the Last 40 Kyrs.” *Marine Geology* 440 (October): 106579. https://doi.org/10.1016/j.margeo.2021.106579.

———. 2021b. “Rapid Humidity Changes Across the Northern South China Sea During the Last 40 Kyrs.” *Marine Geology* 440 (October): 106579. https://doi.org/10.1016/j.margeo.2021.106579.
Kahle, Emma C., Eric J. Steig, Tyler R. Jones, T. J. Fudge, Michelle R. Koutnik, Valerie A. Morris, Bruce H. Vaughn, et al. 2021. “Reconstruction of Temperature, Accumulation Rate, and Layer Thinning from an Ice Core at South Pole, Using a Statistical Inverse Method.” *Journal of Geophysical Research: Atmospheres* 126 (13). https://doi.org/10.1029/2020jd033300.
Kajita, Hiroto, Yuta Isaji, Rintaro Kato, Yoko Nishikura, Masafumi Murayama, Naohiko Ohkouchi, Shouye Yang, et al. 2023. “Climatic Change Around the 4.2 Ka Event in Coastal Areas of the East China Sea and Its Potential Influence on Prehistoric Japanese People.” *Palaeogeography, Palaeoclimatology, Palaeoecology* 609 (January): 111310. https://doi.org/10.1016/j.palaeo.2022.111310.
Kajita, Hiroto, Hodaka Kawahata, Ke Wang, Hongbo Zheng, Shouye Yang, Naohiko Ohkouchi, Masayuki Utsunomiya, Bin Zhou, and Bang Zheng. 2018. “Extraordinary Cold Episodes During the Mid-Holocene in the Yangtze Delta: Interruption of the Earliest Rice Cultivating Civilization.” *Quaternary Science Reviews* 201 (December): 418–28. https://doi.org/10.1016/j.quascirev.2018.10.035.
Kalansky, Julie, Yair Rosenthal, Timothy Herbert, Samantha Bova, and Mark Altabet. 2015. “Southern Ocean Contributions to the Eastern Equatorial Pacific Heat Content During the Holocene.” *Earth and Planetary Science Letters* 424 (August): 158–67. https://doi.org/10.1016/j.epsl.2015.05.013.
Kalis, Arie J., W. O. van der Knaap, Astrid Schweizer, and Ralf Urz. 2006. “A Three Thousand Year Succession of Plant Communities on a Valley Bottom in the Vosges Mountains, NE France, Reconstructed from Fossil Pollen, Plant Macrofossils, and Modern Phytosociological Communities.” *Vegetation History and Archaeobotany* 15 (4): 377–90. https://doi.org/10.1007/s00334-006-0065-7.
Kaniewski, David, Elise Van Campo, Christophe Morhange, Joël Guiot, Dov Zviely, Idan Shaked, Thierry Otto, and Michal Artzy. 2013. “Early Urban Impact on Mediterranean Coastal Environments.” *Scientific Reports* 3 (1). https://doi.org/10.1038/srep03540.
Kanner, Lisa C., Stephen J. Burns, Hai Cheng, R. Lawrence Edwards, and Mathias Vuille. 2013. “High-Resolution Variability of the South American Summer Monsoon over the Last Seven Millennia: Insights from a Speleothem Record from the Central Peruvian Andes.” *Quaternary Science Reviews* 75 (September): 1–10. https://doi.org/10.1016/j.quascirev.2013.05.008.
Kasper, Thomas, Torsten Haberzettl, Stefan Doberschütz, Gerhard Daut, Junbo Wang, Liping Zhu, Norbert Nowaczyk, and Roland Mäusbacher. 2012. “Indian Ocean Summer Monsoon (IOSM)-Dynamics Within the Past 4 Ka Recorded in the Sediments of Lake Nam Co, Central Tibetan Plateau (China).” *Quaternary Science Reviews* 39 (April): 73–85. https://doi.org/10.1016/j.quascirev.2012.02.011.
Kathayat, Gayatri, Hai Cheng, Ashish Sinha, Max Berkelhammer, Haiwei Zhang, Pengzhen Duan, Hanying Li, Xianglei Li, Youfeng Ning, and R. Lawrence Edwards. 2018a. “Evaluating the Timing and Structure of the 4.2 Ka Event in the Indian Summer Monsoon Domain from an Annually Resolved Speleothem Record from Northeast India.” *Climate of the Past* 14 (12): 1869–79. https://doi.org/10.5194/cp-14-1869-2018.
———. 2018b. “Evaluating the Timing and Structure of the 4.2ka Event in the Indian Summer Monsoon Domain from an Annually Resolved Speleothem Record from Northeast India.” *Climate of the Past* 14 (12): 1869–79. https://doi.org/10.5194/cp-14-1869-2018.
Kathayat, Gayatri, Hai Cheng, Ashish Sinha, Liang Yi, Xianglei Li, Haiwei Zhang, Hangying Li, Youfeng Ning, and R. Lawrence Edwards. 2017. “The Indian Monsoon Variability and Civilization Changes in the Indian Subcontinent.” *Science Advances* 3 (12). https://doi.org/10.1126/sciadv.1701296.
Kaufman, Darrell S., Yarrow Axford, R. Scott Anderson, Scott F. Lamoureux, Daniel E. Schindler, Ian R. Walker, and Al Werner. 2012. “A Multi-Proxy Record of the Last Glacial Maximum and Last 14,500 Years of Paleoenvironmental Change at Lone Spruce Pond, Southwestern Alaska.” *Journal of Paleolimnology* 48 (1): 9–26. https://doi.org/10.1007/s10933-012-9607-4.
Kaufman, Darrell, Nicholas McKay, Cody Routson, Michael Erb, Basil Davis, Oliver Heiri, Samuel Jaccard, et al. 2020. “A Global Database of Holocene Paleotemperature Records.” *Scientific Data* 7 (1). https://doi.org/10.1038/s41597-020-0445-3.
Kaushal, Nikita, Sebastian F. M. Breitenbach, Franziska A. Lechleitner, Ashish Sinha, Vinod C. Tewari, Syed Masood Ahmad, Max Berkelhammer, et al. 2018. “The Indian Summer Monsoon from a Speleothem δ18O Perspective—a Review.” *Quaternary* 1 (3): 29. https://doi.org/10.3390/quat1030029.
Kawahata, Hodaka. 2019a. “Climatic Reconstruction at the Sannai-Maruyama Site Between Bond Events 4 and 3-Implication for the Collapse of the Society at 4.2 Ka Event.” *Progress in Earth and Planetary Science* 6 (1). https://doi.org/10.1186/s40645-019-0308-8.

———. 2019b. “Climatic Reconstruction at the Sannai-Maruyama Site Between Bond Events 4 and 3—Implication for the Collapse of the Society at 4.2 Ka Event.” *Progress in Earth and Planetary Science* 6 (1). https://doi.org/10.1186/s40645-019-0308-8.

Kawahata, Hodaka, Hisashi Yamamoto, KenOhkushi, Yusuke Yokoyama, Katsunori Kimoto, Hideki Ohshima, and Hiroyuki Matsuzaki. 2009. “Changes of Environments and Human Activity at the Sannai-Maruyama Ruins in Japan During the Mid-Holocene Hypsithermal Climatic Interval.” *Quaternary Science Reviews* 28 (9-10): 964–74. https://doi.org/10.1016/j.quascirev.2008.12.009.

Keigwin, L. D., J. P. Sachs, Y. Rosenthal, and E. A. Boyle. 2005. “The 8200 Year b.p. Event in the Slope Water System, Western Subpolar North Atlantic.” *Paleoceanography* 20 (2). https://doi.org/10.1029/2004pa001074.

Kelly, Robert L., Todd A. Surovell, Bryan N. Shuman, and Geoffrey M. Smith. 2012. “A Continuous Climatic Impact on Holocene Human Population in the Rocky Mountains.” *Proceedings of the National Academy of Sciences* 110 (2): 443–47. https://doi.org/10.1073/pnas.1201341110.

Kennett, Douglas J., James P. Kennett, Jon M. Erlandson, and Kevin G. Cannariato. 2007a. “Human Responses to Middle Holocene Climate Change on California’s Channel Islands.” *Quaternary Science Reviews* 26 (3–4): 351–67. https://doi.org/10.1016/j.quascirev.2006.07.019.

———. 2007b. “Human Responses to Middle Holocene Climate Change on Californias Channel Islands.” *Quaternary Science Reviews* 26 (3-4): 351–67. https://doi.org/10.1016/j.quascirev.2006.07.019.

Kim, Jung-Hyun, Helge Meggers, Norel Rimbu, Gerrit Lohmann, Tim Freudenthal, Peter J. Müller, and Ralph R. Schneider. 2007. “Impacts of the North Atlantic Gyre Circulation on Holocene Climate Off Northwest Africa.” *Geology* 35 (5): 387. https://doi.org/10.1130/g23251a.1.

Kim, Jung-Hyun, Norel Rimbu, Stephan J. Lorenz, Gerrit Lohmann, Seung-Il Nam, Stefan Schouten, Carsten Rühlemann, and Ralph R. Schneider. 2004. “North Pacific and North Atlantic Sea-Surface Temperature Variability During the Holocene.” *Quaternary Science Reviews* 23 (20-22): 2141–54. https://doi.org/10.1016/j.quascirev.2004.08.010.

Kimmel, K, R. Pirrus, and A. Raukas. 1999. “Holocene Deposits.” *In: Lake Peipsi: Geology Ed. By A. Miidel and A. Raukas (Pp.42-52). Sulemees Publishers*.

Kimmel, K, R. Rajamae, and M. Sakson. 1996. “The Holocene Development of Tondi Mire, Northern Estonia: Pollen, Diatom and Chronological Studies.” *In: Coastal Estonia. Recent Advances in Environmental and Cultural History Ed. By T. Hackens*.

King, James E. 1981. “Late Quaternary Vegetational History of Illinois.” *Ecological Monographs* 51 (1): 43–62. https://doi.org/10.2307/2937306.

Kirby, M. E., S. P. Lund, W. P. Patterson, M. A. Anderson, B. W. Bird, L. Ivanovici, P. Monarrez, and S. Nielsen. 2010. “A Holocene Record of Pacific Decadal Oscillation (PDO)-Related Hydrologic Variability in Southern California (Lake Elsinore, CA).” *Journal of Paleolimnology* 44 (3): 819–39. https://doi.org/10.1007/s10933-010-9454-0.

Kirby, Matthew Edward Christopher, William Paul Patterson, Matthew Lachniet, James A. Noblet, Michael A. Anderson, Kevin Nichols, and Judith Avila. 2019a. “Pacific Southwest United States Holocene Droughts and Pluvials Inferred from Sediment d18O(calcite) and Grain Size Data (Lake Elsinore, California).” *Frontiers in Earth Science* 7 (April). https://doi.org/10.3389/feart.2019.00074.

———. 2019b. “Pacific Southwest United States Holocene Droughts and Pluvials Inferred from Sediment δ18O(calcite) and Grain Size Data (Lake Elsinore, California).” *Frontiers in Earth Science* 7 (April). https://doi.org/10.3389/feart.2019.00074.

Kirby, Matthew E., Edward J. Knell, William T. Anderson, Matthew S. Lachniet, Jennifer Palermo, Holly Eeg, Ricardo Lucero, et al. 2015. “Evidence for Insolation and Pacific Forcing of Late Glacial Through Holocene Climate in the Central Mojave Desert (Silver Lake, CA).” *Quaternary Research* 84 (2): 174–86. https://doi.org/10.1016/j.yqres.2015.07.003.

Kirby, Matthew E., Susan R. H. Zimmerman, William P. Patterson, and Jose J. Rivera. 2012. “A 9170-Year Record of Decadal-to-Multi-Centennial Scale Pluvial Episodes from the Coastal Southwest United States: A Role for Atmospheric Rivers?” *Quaternary Science Reviews* 46 (July): 57–65. https://doi.org/10.1016/j.quascirev.2012.05.008.

Knipping, M. 1989. “Zur Spat-Und Postglazialen Vegetationsgeschichte Des-Oberpfalzer Waldes.” *Dissertationes Botanicae*.

Konigsson, L.-K. 1984. “Vegetationsgeschichte Und Kultureinflusse in Der Landschaftsentwicklung Der Sudschwedischen Gebirge in Harjedalen.” *Dissertationes Botanicae 72 (Festschrift Max Welten)*.

Korhola, A., K. Vaskov, H. Toivonen, and H. Olander. 2002. "Holocene Temperature Changes in Northern Fennoscandia Reconstructed from Chironomids Using Bayesian Modelling." *Quaternary Science Reviews* 21 (16-17): 1841–60. https://doi.org/10.1016/s0277-3791(02)00003-3.

Kouli, Katerina, and Michael D. Dermitzakis. 2010. "11. Lake Orestiás (Kastoria, Northern Greece)." *Grana* 49 (2): 154–56. https://doi.org/10.1080/00173131003780016.

Kramer, Annette, Ulrike Herzschuh, Steffen Mischke, and Chengjun Zhang. 2010. "Holocene Treeline Shifts and Monsoon Variability in the Hengduan Mountains (Southeastern Tibetan Plateau), Implications from Palynological Investigations." *Palaeogeography, Palaeoclimatology, Palaeoecology* 286 (1-2): 23–41. https://doi.org/10.1016/j.palaeo.2009.12.001.

Krause, Claire E., Michael K. Gagan, Gavin B. Dunbar, Wahyoe S. Hantoro, John C. Hellstrom, Hai Cheng, R. Lawrence Edwards, Bambang W. Suwargadi, Nerilie J. Abram, and Hamdi Rifai. 2019. "Spatio-Temporal Evolution of Australasian Monsoon Hydroclimate over the Last 40,000 Years." *Earth and Planetary Science Letters* 513 (May): 103–12. https://doi.org/10.1016/j.epsl.2019.01.045.

Krause, Teresa R., Yanbin Lu, Cathy Whitlock, Sherilyn C. Fritz, and Kenneth L. Pierce. 2015. "Patterns of Terrestrial and Limnologic Development in the Northern Greater Yellowstone Ecosystem (USA) During the Late-Glacial/Early-Holocene Transition." *Palaeogeography, Palaeoclimatology, Palaeoecology* 422 (March): 46–56. https://doi.org/10.1016/j.palaeo.2014.12.018.

Krause, Teresa R., and Cathy Whitlock. 2013. "Climate and Vegetation Change During the Late-Glacial/Early-Holocene Transition Inferred from Multiple Proxy Records from Blacktail Pond, Yellowstone National Park, USA." *Quaternary Research* 79 (3): 391–402. https://doi.org/10.1016/j.yqres.2013.01.005.

Krisai, R, W Mayer, C. SCHROCK, and R. Turk. 2006. "Das Gradenmoos in Der Schobergruppe (NP Hohe Tauern, Karnten) Vegetation Und Entstehung." *Carinthia II*.

Kristj'ansd'ottir, Greta B, Matthias Moros, John T Andrews, and Anne E Jennings. 2016. "Holocene Mg/Ca, Alkenones, and Light Stable Isotope Measurements on the Outer North Iceland Shelf (MD99-2269): A Comparison with Other Multi-Proxy Data and Sub-Division of the Holocene." *The Holocene* 27 (1): 52–62. https://doi.org/10.1177/0959683616652703.

KroNANApelin, S., D. Verschuren, A.-M. LNAeNAzine, H. Eggermont, C. Cocquyt, P. Francus, J.-P. Cazet, et al. 2008. "Climate-Driven Ecosystem Succession in the Sahara: The Past 6000 Years." *Science* 320 (5877): 765–68. https://doi.org/10.1126/science.1154913.

Kubota, Yoshimi, Katsunori Kimoto, Ryuji Tada, Hirokuni Oda, Yusuke Yokoyama, and Hiroyuki Matsuzaki. 2010. "Variations of East Asian Summer Monsoon Since the Last Deglaciation Based on Mg/Ca and Oxygen Isotope of Planktic Foraminifera in the Northern East China Sea." *Paleoceanography* 25 (4): n/a–. https://doi.org/10.1029/2009pa001891.

Kuhnert, Henning, Holger Kuhlmann, Mahyar Mohtadi, Helge Meggers, Karl-Heinz Baumann, and Jürgen Pätzold. 2014. "Holocene Tropical Western Indian Ocean Sea Surface Temperatures in Covariation with Climatic Changes in the Indonesian Region." *Paleoceanography* 29 (5): 423–37. https://doi.org/10.1002/2013pa002555.

Kvavadze, E. V., and L. P. Rukhadze. 1989. "Vegetation and Climate of the Holocene of Abkhazia." *In: Metsniereba*.

L'opez-Merino, L., J. A. L'opez-S'aez, F. Alba-S'anchez, and S. P'erez-Dand J. S. Carri'on. 2009. "2000 Years of Pastoralism and Fire Shaping High-Altitude Vegetation of Sierra de Gredos in Central Spain." *Review of Palaeobotany and Palynology* 158 (1-2): 42–51. https://doi.org/10.1016/j.revpalbo.2009.07.003.

L'opez-Merino, Lourdes, and Antonio Martand Jos'e Antonio L'opez-S'aez. 2011. "Human-Induced Changes on Wetlands: A Study Case from NW Iberia." *Quaternary Science Reviews* 30 (19-20): 2745–54. https://doi.org/10.1016/j.quascirev.2011.06.004.

L'opez-S'aez, Jos'e Antonio, Lourdes L'opez-Merino, Francisca Alba-S'anchez, Sebasti'an P'erez-Dand Daniel Abel-Schaad, and Jos'e S. Carri'on. 2009. "Late Holocene Ecological History of Pinus Pinaster Forests in the Sierra de Gredos of Central Spain." *Plant Ecology* 206 (2): 195–209. https://doi.org/10.1007/s11258-009-9634-z.

Lacey, Jack H., Alexander Francke, Melanie J. Leng, Christopher H. Vane, and Bernd Wagner. 2014. "A High-Resolution Late Glacial to Holocene Record of Environmental Change in the Mediterranean from Lake Ohrid (Macedonia/Albania)." *International Journal of Earth Sciences* 104 (6): 1623–38. https://doi.org/10.1007/s00531-014-1033-6.

Lachniet, Matthew S., Rhawn F. Denniston, Yemane Asmerom, and Victor J. Polyak. 2014. "Orbital Control of Western North America Atmospheric Circulation and Climate over Two Glacial Cycles." *Nature Communications* 5 (1). https://doi.org/10.1038/ncomms4805.

Laird, K. R. 1996. “A High-Resolution Paleoclimatic Record of a Closed-Basin Lake in the Northern Great Plains.” *Doctoral Dissertation. University of Minnesota*.

Laird, Kathleen R., Sherilyn C. Fritz, Eric C. Grimm, and Pietra G. Mueller. 1996. “Century Scale Paleoclimatic Reconstruction from Moon Lake, a Closed-Basin Lake in the Northern Great Plains.” *Limnology and Oceanography* 41 (5): 890–902. https://doi.org/10.4319/lo.1996.41.5.0890.

Lamy, Frank, Helge W. Arz, G’erard C. Bond, Andr’e Bahr, and Jürgen Pätzold. 2006. “Multicentennial-scale Hydrological Changes in the Black Sea and Northern Red Sea During the Holocene and the Arctic/North Atlantic Oscillation.” *Paleoceanography* 21 (1). https://doi.org/10.1029/2005pa001184.

Lamy, Frank, Carsten Rühlemann, Dierk Hebbeln, and Gerold Wefer. 2002. “High- and Low-Latitude Climate Control on the Position of the Southern Peru-Chile Current During the Holocene.” *Paleoceanography* 17 (2): 16-1-16-10. https://doi.org/10.1029/2001pa000727.

Larmon, J. T. 2013. “The Pacific Coast of Guatemala: A Palynological Investigation of Climate Change and Human Occupation.” *Master’s Thesis. Washington State University*.

LAROCQUE, I. 2004. “Holocene Temperature Estimates and Chironomid Community Composition in the Abisko Valley, Northern Sweden.” *Quaternary Science Reviews* 23 (23-24): 2453–65. https://doi.org/10.1016/j.quascirev.2004.04.006.

Larrasoaña, Juan C., Andrew P. Roberts, and Eelco J. Rohling. 2008. “Magnetic Susceptibility of Eastern Mediterranean Marine Sediments as a Proxy for Saharan Dust Supply?” *Marine Geology* 254 (3–4): 224–29. https://doi.org/10.1016/j.margeo.2008.06.003.

Larsen, Darren J., Gifford H. Miller, ’Aslaug Geirsd’ottir, and Thorvaldur Thordarson. 2011a. “A 3000-Year Varved Record of Glacier Activity and Climate Change from the Proglacial Lake Hvítárvatn, Iceland.” *Quaternary Science Reviews* 30 (19–20): 2715–31. https://doi.org/10.1016/j.quascirev.2011.05.026.

———. 2011b. “A 3000-Year Varved Record of Glacier Activity and Climate Change from the Proglacial Lake Hvítárvatn, Iceland.” *Quaternary Science Reviews* 30 (19-20): 2715–31. https://doi.org/10.1016/j.quascirev.2011.05.026.

Lasher, G. Everett, Mark B. Abbott, Lesleigh Anderson, Lindsey Yasarer, Michael Rosenmeier, and Bruce P. Finney. 2021. “Holocene Hydroclimatic Reorganizations in Northwest Canada Inferred from Lacustrine Carbonate Oxygen Isotopes.” *Geophysical Research Letters* 48 (16). https://doi.org/10.1029/2021gl092948.

Laskar, Amzad Hussain, and Archna Bohra. 2021. “Impact of Indian Summer Monsoon Change on Ancient Indian Civilizations During the Holocene.” *Frontiers in Earth Science* 9 (September). https://doi.org/10.3389/feart.2021.709455.

Latalowa, M. 1976. “Diagram Pylkowy Osadow Poznoglacjalnych i Holocenskich z Torfowiska w Wolbromiu.” *Acta Palaeobotanica*.

———. 1992. “Forest Changes at the Mesolithic/Early Neolithic Transition and in the Migration Period on Wolin Island (NW Poland).” *Palaeoclimate Res*.

Lauritzen, Stein-Erik, and Joyce Lundberg. 1999. “Calibration of the Speleothem Delta Function: An Absolute Temperature Record for the Holocene in Northern Norway.” *The Holocene* 9 (6): 659–69. https://doi.org/10.1191/095968399667823929.

LAUTERBACH, STEFAN, ACHIM BRAUER, NILS ANDERSEN, DAN L. DANIELOPOL, PETER DULSKI, MATTHIAS HÜLS, KRYSTYNA MILECKA, et al. 2010. “Multi-Proxy Evidence for Early to Mid-Holocene Environmental and Climatic Changes in Northeastern Poland.” *Boreas* 40 (1): 57–72. https://doi.org/10.1111/j.1502-3885.2010.00159.x.

Lauterbach, Stefan, Roman Witt, Birgit Plessen, Peter Dulski, Sushma Prasad, Jens Mingram, Gerd Gleixner, et al. 2014. “Climatic Imprint of the Mid-Latitude Westerlies in the Central Tian Shan of Kyrgyzstan and Teleconnections to North Atlantic Climate Variability During the Last 6000 Years.” *The Holocene* 24 (8): 970–84. https://doi.org/10.1177/0959683614534741.

Le Roy, Melaine, Philip Deline, Julien Carcaillet, Irene Schimmelpfennig, and Magali Ermini. 2017. “10Be Exposure Dating of the Timing of Neoglacial Glacier Advances in the Ecrins-Pelvoux Massif, Southern French Alps.” *Quaternary Science Reviews* 178 (December): 118–38. https://doi.org/10.1016/j.quascirev.2017.10.010.

Lecavalier, Benoit S., David A. Fisher, Glenn A. Milne, Bo M. Vinther, Lev Tarasov, Philippe Huybrechts, Denis Lacelle, et al. 2017. “High Arctic Holocene Temperature Record from the Agassiz Ice Cap and Greenland Ice Sheet Evolution.” *Proceedings of the National Academy of Sciences* 114 (23): 5952–57. https://doi.org/10.1073/pnas.1616287114.

Lechleitner, Franziska A., Sebastian F. M. Breitenbach, Hai Cheng, Birgit Plessen, Kira Rehfeld, Bedartha Goswami, Norbert Marwan, Deniz Eroglu, Jess Adkins, and Gerald Haug. 2017a. “Climatic and in-Cave

Influences on d18O and d13C in a Stalagmite from Northeastern India Through the Last Deglaciation." *Quaternary Research* 88 (3): 458–71. https://doi.org/10.1017/qua.2017.72.
———. 2017b. "Climatic and in-Cave Influences on δ18O and δ13C in a Stalagmite from Northeastern India Through the Last Deglaciation." *Quaternary Research* 88 (3): 458–71. https://doi.org/10.1017/qua.2017.72.
Leipe, Christian, Norio Kito, Yumi Sakaguchi, and Pavel E. Tarasov. 2013. "Vegetation and Climate History of Northern Japan Inferred from the 5500-Year Pollen Record from the Oshima Peninsula, SW Hokkaido." *Quaternary International* 290-291 (March): 151–63. https://doi.org/10.1016/j.quaint.2012.07.014.
Leira, M, E. E. Cole, and F. J. G. Mitchell. 2007. "Long Term Impacts of Atmospheric Deposition and Peat Erosion on an Oligotrophic Lake in Eastern Ireland." *Journal of Paleolimnology*.
Lemcke, Gerry, and Michael Sturm. 1997. "Δ18o and Trace Element Measurements as Proxy for the Reconstruction of Climate Changes at Lake van (Turkey): Preliminary Results." In *Third Millennium BC Climate Change and Old World Collapse*, 653–78. Springer Berlin Heidelberg. https://doi.org/10.1007/978-3-642-60616-8_29.
Leroy, S. A. G. 2010. "Pollen Analysis of Core DS7-1SC (Dead Sea) Showing Intertwined Effects of Climatic Change and Human Activities in the Late Holocene." *Journal of Archaeological Science* 37 (2): 306–16. https://doi.org/10.1016/j.jas.2009.09.042.
Leroy, Suzanne A. G., Lourdes L'opez-Merino, Alina Tudryn, Françoise Chali'e, and Françoise Gasse. 2014. "Late Pleistocene and Holocene Palaeoenvironments in and Around the Middle Caspian Basin as Reconstructed from a Deep-Sea Core." *Quaternary Science Reviews* 101 (October): 91–110. https://doi.org/10.1016/j.quascirev.2014.07.011.
Lespez, L, J. M Cador, V Carpentier, M Clet-Pellerin, M. A Germaine, E. Garnier, and C. Marcigny. 2008. "Trajectoire Des Paysages Des Vallees Normandes Et Gestion de l'eau, Du Neolithique Aux Enjeux de La Gestion Contemporaine." *In: Paysage Et Environnement: De La Reconstitution Du Passe Aux Modeles Prospectifs Ed. By Galop*.
Lespez, L, B Hardel, M Clet-Pellerin, R. Davidson, and C. Marcigny. 2005. "Evolution Des Paysages Du Neolithique a Nos Jours Dans La Peninsule de La Hague (Normandie, France), l'exemple de l'anse Saint-Martin." *Archeologie*.
Levac, Elisabeth, Anne De Vernal, and Weston Blake Jr. 2001. "Sea-surface Conditions in Northernmost Baffin Bay During the Holocene: Palynological Evidence." *Journal of Quaternary Science* 16 (4): 353–63. https://doi.org/10.1002/jqs.614.
Levy, Laura B., Darrell S. Kaufman, and Al Werner. 2004. "Holocene Glacier Fluctuations, Waskey Lake, Northeastern Ahklun Mountains, Southwestern Alaska." *The Holocene* 14 (2): 185–93. https://doi.org/10.1191/0959683604hl675rp.
Li, Chunhai, Yanhong Wu, and Xinhua Hou. 2011. "Holocene Vegetation and Climate in Northeast China Revealed from Jingbo Lake Sediment." *Quaternary International* 229 (1-2): 67–73. https://doi.org/10.1016/j.quaint.2009.12.015.
Li, Hanying, Hai Cheng, Ashish Sinha, Gayatri Kathayat, Christoph Spötl, Aur'ele Anquetil Andr'e, Arnaud Meunier, et al. 2018. "Hydro-Climatic Variability in the Southwestern Indian Ocean Between 6000 and 3000 Years Ago." *Climate of the Past* 14 (12): 1881–91. https://doi.org/10.5194/cp-14-1881-2018.
Li, Hong-Chun, Zhong-Hong Lee, Nai-Jung Wan, Chuan-Chou Shen, Ting-Yong Li, Dao-Xian Yuan, and Yong-Heng Chen. 2011a. "The d18O and d13C Records in an Aragonite Stalagmite from Furong Cave, Chongqing, China: A-2000-Year Record of Monsoonal Climate." *Journal of Asian Earth Sciences* 40 (6): 1121–30. https://doi.org/10.1016/j.jseaes.2010.06.011.
———. 2011b. "The δ18O and δ13C Records in an Aragonite Stalagmite from Furong Cave, Chongqing, China: A-2000-Year Record of Monsoonal Climate." *Journal of Asian Earth Sciences* 40 (6): 1121–30. https://doi.org/10.1016/j.jseaes.2010.06.011.
Li, Jianyong, John Dodson, Hong Yan, Weiming Wang, James B. Innes, Yongqiang Zong, Xiaojian Zhang, Qinghai Xu, Jian Ni, and Fengyan Lu. 2017. "Quantitative Holocene Climatic Reconstructions for the Lower Yangtze Region of China." *Climate Dynamics* 50 (3-4): 1101–13. https://doi.org/10.1007/s00382-017-3664-3.
Li, Jun-Yun, Hong-Chun Li, Ting-Yong Li, Horng-Sheng Mii, Tsai-Lune Yu, Chuan-Chou Shen, and Xiaomei Xu. 2017a. "High-Resolution d18O and d13C Records of an AMS 14C and 230Th/u Dated Stalagmite from Xinya Cave in Chongqing: Climate and Vegetation Change During the Late Holocene." *Quaternary International* 447 (August): 75–88. https://doi.org/10.1016/j.quaint.2017.06.075.

———. 2017b. “High-Resolution δ18O and δ13C Records of an AMS 14C and 230Th/u Dated Stalagmite from Xinya Cave in Chongqing: Climate and Vegetation Change During the Late Holocene.” *Quaternary International* 447 (August): 75–88. https://doi.org/10.1016/j.quaint.2017.06.075.

Li, Kaifeng, and Wenhua Gao. 2021. “Human Settlement Distribution Patterns During the Longshan and Xinzhai-Erlitou Periods and Their Hydrogeomorphic Contexts in the Central Plains, China.” *CATENA* 204 (September): 105433. https://doi.org/10.1016/j.catena.2021.105433.

Li, Ting, Christopher M. Wurster, Jordahna Haig, Youping Zhou, Costijn Zwart, Junli Ren, Rainy Comley, Niels C. Munksgaard, Patricia S. Gadd, and Michael I. Bird. 2022. “Environmental Change Inferred from Multiple Proxies from an 18 Cal Ka BP Sediment Record, Lake Barrine, NE Australia.” *Quaternary Science Reviews* 294 (October): 107751. https://doi.org/10.1016/j.quascirev.2022.107751.

Li, Ting-Yong, Chuan-Chou Shen, Hong-Chun Li, Jun-Yun Li, Hong-Wei Chiang, Sheng-Rong Song, Dao-Xian Yuan, et al. 2011. “Oxygen and Carbon Isotopic Systematics of Aragonite Speleothems and Water in Furong Cave, Chongqing, China.” *Geochimica Et Cosmochimica Acta* 75 (15): 4140–56. https://doi.org/10.1016/j.gca.2011.04.003.

Li, TingYong, DaoXian Yuan, HongChun Li, Yan Yang, JianLi Wang, XinYa Wang, JunYun Li, JiaMing Qin, MeiLiang Zhang, and YuShi Lin. 2007a. “High-Resolution Climate Variability of Southwest China During 57-70 Ka Reflected in a Stalagmite d18O Record from Xinya Cave.” *Science in China Series D: Earth Sciences* 50 (8): 1202–8. https://doi.org/10.1007/s11430-007-0059-z.

———. 2007b. “High-Resolution Climate Variability of Southwest China During 57–70 Ka Reflected in a Stalagmite δ 18O Record from Xinya Cave.” *Science in China Series D: Earth Sciences* 50 (8): 1202–8. https://doi.org/10.1007/s11430-007-0059-z.

Li, Yun, Li Han, Xingqi Liu, Yougui Song, and Yixuan Wang. 2021. “Correlation and Anti-Correlation of the Asian Summer Monsoon and Westerlies During the Holocene.” *Gondwana Research* 91 (March): 112–20. https://doi.org/10.1016/j.gr.2020.12.013.

Liefert, David T., and Bryan N. Shuman. 2020. “Pervasive Desiccation of North American Lakes During the Late Quaternary.” *Geophysical Research Letters* 47 (3). https://doi.org/10.1029/2019gl086412.

———. 2022a. “Expression of the ‘4.2 Ka Event’ in the Southern Rocky Mountains, USA.” *Climate of the Past* 18 (5): 1109–24. https://doi.org/10.5194/cp-18-1109-2022.

———. 2022b. “Expression of the"4.2ka Event" in the Southern Rocky Mountains, USA.” *Climate of the Past* 18 (5): 1109–24. https://doi.org/10.5194/cp-18-1109-2022.

Liew, Ping-Mei, and Shu-Yue Huang. 1994. “A 5000-Year Pollen Record from Chitsai Lake, Central Taiwan.” *Terrestrial, Atmospheric and Oceanic Sciences* 5 (3): 411. https://doi.org/10.3319/tao.1994.5.3.411(pages).

Lin, Jie, Wenying Jiang, Luo Wang, Enlou Zhang, Lingyu Tang, Xiaoxiao Yang, Guoqiang Chu, Shiling Yang, and Jule Xiao. 2022. “Spatially Diverse Hydroclimatic Response to the 4.2 Ka Event in the Asian Monsoon Region.” *Quaternary Science Reviews* 296 (November): 107809. https://doi.org/10.1016/j.quascirev.2022.107809.

Lin, Yu-Shih, Kuo-Yen Wei, In-Tian Lin, Pai-Sen Yu, Hong-Wei Chiang, Chen-Yin Chen, Chuan-Chou Shen, Horng-Sheng Mii, and Yue-Gau Chen. 2006. “The Holocene Pulleniatina Minimum Event Revisited: Geochemical and Faunal Evidence from the Okinawa Trough and Upper Reaches of the Kuroshio Current.” *Marine Micropaleontology* 59 (3-4): 153–70. https://doi.org/10.1016/j.marmicro.2006.02.003.

Linge, H., S.-E. Lauritzen, C. Andersson, J. K. Hansen, R. Ø. Skoglund, and H. S. Sundqvist. 2009. “Stable Isotope Records for the Last 10 000 Years from Okshola Cave (Fauske, Northern Norway) and Regional Comparisons.” *Climate of the Past* 5 (4): 667–82. https://doi.org/10.5194/cp-5-667-2009.

Linsley, Braddock K., Yair Rosenthal, and Delia W. Oppo. 2010. “Holocene Evolution of the Indonesian Throughflow and the Western Pacific Warm Pool.” *Nature Geoscience* 3 (8): 578–83. https://doi.org/10.1038/ngeo920.

LITT, THOMAS, CHRISTIAN SCHÖLZEL, NORBERT KÜHL, and ACHIM BRAUER. 2009. “Vegetation and Climate History in the Westeifel Volcanic Field (Germany) During the Past 11 000 Years Based on Annually Laminated Lacustrine Maar Sediments.” *Boreas* 38 (4): 679–90. https://doi.org/10.1111/j.1502-3885.2009.00096.x.

Litt, T., and K. Tobolski. 1991. “Beitraege Zur Postglazialen Vegetaionsgeschichte Im Lednica-Gebiet.” *Wstep Do Paleoekologii Lednickiego Parku Krajobrazowego*, 57 61.

Liu, Dianbing, Yongjin Wang, Hai Cheng, R. L. Edwards, and Xinggong Kong. 2015. “Cyclic Changes of Asian Monsoon Intensity During the Early Mid-Holocene from Annually-Laminated Stalagmites, Central China.” *Quaternary Science Reviews* 121 (August): 1–10. https://doi.org/10.1016/j.quascirev.2015.05.003.

Liu, Fenggui, and Zhaodong Feng. 2012. "A Dramatic Climatic Transition at 4000 Cal. Yr BP and Its Cultural Responses in Chinese Cultural Domains." *The Holocene* 22 (10): 1181–97. https://doi.org/10.1177/0959683612441839.
Liu, Xiaokang, Zhiguo Rao, Chuan-Chou Shen, Jianbao Liu, Jianhui Chen, Shengqian Chen, Xianfeng Wang, and Fahu Chen. 2019. "Holocene Solar Activity Imprint on Centennial- to Multidecadal-Scale Hydroclimatic Oscillations in Arid Central Asia." *Journal of Geophysical Research: Atmospheres* 124 (5): 2562–73. https://doi.org/10.1029/2018jd029699.
LIU, Xingqi. 2002. "A 16000-Year Pollen Record of Qinghai Lake and Its Paleocli-Mate and Paleoenvironment." *Chinese Science Bulletin* 47: 1931.
Liu, Xingqi, Ulrike Herzschuh, Ji Shen, Qingfen Jiang, and Xiayun Xiao. 2008. "Holocene Environmental and Climatic Changes Inferred from Wulungu Lake in Northern Xinjiang, China." *Quaternary Research* 70 (3): 412–25. https://doi.org/10.1016/j.yqres.2008.06.005.
Liu, Xiting, Rebecca Rendle-Bühring, and Rüdiger Henrich. 2018. "High-and Low-Latitude Forcing of the East African Climate Since the LGM: Inferred from the Elemental Composition of Marine Sediments Off Tanzania." *Quaternary Science Reviews* 196 (September): 124–36. https://doi.org/10.1016/j.quascirev.2018.08.004.
Liu, Y-H., G. M. Henderson, C-Y. Hu, A. J. Mason, N. Charnley, K. R. Johnson, and S-C. Xie. 2013. "Links Between the East Asian Monsoon and North Atlantic Climate During the 8,200 Year Event." *Nature Geoscience* 6 (2): 117–20. https://doi.org/10.1038/ngeo1708.
Long, Colin J., Jaqueline J. Shinker, Thomas A. Minckley, Mitchell J. Power, and Patrick J. Bartlein. 2019. "A 7600 Yr Vegetation and Fire History from Anthony Lake, Northeastern Oregon, USA, with Linkages to Modern Synoptic Climate Patterns." *Quaternary Research* 91 (2): 705–13. https://doi.org/10.1017/qua.2018.124.
Lorrey, Andrew, Paul Williams, Jim Salinger, Tim Martin, Jonathan Palmer, Anthony Fowler, Jian-xin Zhao, and Helen Neil. 2008. "Speleothem Stable Isotope Records Interpreted Within a Multi-Proxy Framework and Implications for New Zealand Palaeoclimate Reconstruction." *Quaternary International* 187 (1): 52–75. https://doi.org/10.1016/j.quaint.2007.09.039.
Lotter, A. F. 1988. "Palaookologische Und Palaolimnologische Studie Des Rotsees Bei Luzern." *Pollen-*.
Lotter, Andr'e F., Oliver Heiri, Wolfgang Hofmann, Willem O. van der Knaap, Jacqueline F. N. van Leeuwen, Ian R. Walker, and Lucia Wick. 2006. "Holocene Timber-Line Dynamics at Bachalpsee, a Lake at 2265 m a.s.l. In the Northern Swiss Alps." *Vegetation History and Archaeobotany* 15 (4): 295–307. https://doi.org/10.1007/s00334-006-0060-z.
Lucas, Jennifer D., and Terri Lacourse. 2013a. "Holocene Vegetation History and Fire Regimes of <i>pseudotsuga Menziesii</i> Forests in the Gulf Islands National Park Reserve, Southwestern British Columbia, Canada." *Quaternary Research* 79 (3): 366–76. https://doi.org/10.1016/j.yqres.2013.03.001.
———. 2013b. "Holocene Vegetation History and Fire Regimes of Pseudotsuga Menziesii Forests in the Gulf Islands National Park Reserve, Southwestern British Columbia, Canada." *Quaternary Research* 79 (3): 366–76. https://doi.org/10.1016/j.yqres.2013.03.001.
Lückge, Andreas, Mahyar Mohtadi, Carsten Rühlemann, Georg Scheeder, Annemiek Vink, Lutz Reinhardt, and Michael Wiedicke. 2009. "Monsoon Versus Ocean Circulation Controls on Paleoenvironmental Conditions Off Southern Sumatra During the Past 300,000 Years." *Paleoceanography* 24 (1). https://doi.org/10.1029/2008pa001627.
Lundeen, Zachary, Andrea Brunelle, Stephen J. Burns, Victor Polyak, and Yemane Asmerom. 2013. "A Speleothem Record of Holocene Paleoclimate from the Northern Wasatch Mountains, Southeast Idaho, USA." *Quaternary International* 310 (October): 83–95. https://doi.org/10.1016/j.quaint.2013.03.018.
Lynch, Elizabeth A., Sara C. Hotchkiss, and Randy Calcote. 2011. "Charcoal Signatures Defined by Multivariate Analysis of Charcoal Records from 10 Lakes in Northwest Wisconsin (USA)." *Quaternary Research* 75 (1): 125–37. https://doi.org/10.1016/j.yqres.2010.08.007.
MacDonald, Glen M., Katrina A. Moser, Amy M. Bloom, Aaron P. Potito, David F. Porinchu, James R. Holmquist, Julia Hughes, and Konstantine V. Kremenetski. 2016. "Prolonged California Aridity Linked to Climate Warming and Pacific Sea Surface Temperature." *Scientific Reports* 6 (1). https://doi.org/10.1038/srep33325.
Maenza-Gmelch, Terryanne E. 1997. "Holocene Vegetation, Climate, and Fire History of the Hudson Highlands, Southeastern New York, USA." *The Holocene* 7 (1): 25–37. https://doi.org/10.1177/095968369700700103.
Magny, Michel, Boris Vanni'ere, Gianni Zanchetta, Eric Fouache, Gilles Touchais, Lera Petrika, C'eline Coussot, Anne-V'eronique Walter-Simonnet, and Fabien Arnaud. 2009. "Possible Complexity of the Climatic Event

Around 4300—3800 Cal. BP in the Central and Western Mediterranean." *The Holocene* 19 (6): 823–33. https://doi.org/10.1177/0959683609337360.
MAHER, LOUIS J. 1963. "Pollen Analyses of Surface Materials from the Southern San Juan Mountains, Colorado." *Geological Society of America Bulletin* 74 (12): 1485. https://doi.org/10.1130/0016-7606(1963)74[1485:paosmf]2.0.co;2.
Makohonienko, M. 1991. "Untitled." *Wstep Do Paleoecologi Lednideiego Parku Krajobvazowego*, 63 70.
Malamud-Roam, Frances, and B. Lynn Ingram. 2004. "Late Holocene d¹³c and Pollen Records of Paleosalinity from Tidal Marshes in the San Francisco Bay Estuary, California." *Quaternary Research* 62 (2): 134–45. https://doi.org/10.1016/j.yqres.2004.02.011.
Malamud-Roam, Frances, and B. Lynn Ingram. 2004. "Late Holocene δ13C and Pollen Records of Paleosalinity from Tidal Marshes in the San Francisco Bay Estuary, California." *Quaternary Research* 62 (2): 134–45. https://doi.org/10.1016/j.yqres.2004.02.011.
Mann, D. H, P. A Heiser, and B. P Finney. 2002. "Holocene History of the Great Kobuk Sand Dunes, Northwestern Alaska." *Quaternary Science Reviews* 21 (4-6): 709–31. https://doi.org/10.1016/s0277-3791(01)00120-2.
Marchal, Olivier, Isabel Cacho, Thomas F. Stocker, Joan O. Grimalt, Eva Calvo, Belen Martrat, Nicholas Shackleton, et al. 2002. "Apparent Long-Term Cooling of the Sea Surface in the Northeast Atlantic and Mediterranean During the Holocene." *Quaternary Science Reviews* 21 (4-6): 455–83. https://doi.org/10.1016/s0277-3791(01)00105-6.
Marchand, Gr'egor, Michel Tessier, and Jacques Bernard. 1999. "Les Occupations Mesolithiques Et Neolithiques de La Fillauderie (Saint-Pere-En-Retz, Loire-Atlantique) Et La Prehistoire Recente de La Basse Vallee Du Boivre." *Revue Archeologique de louest* 16 (1): 39–65. https://doi.org/10.3406/rao.1999.1087.
Marchant, R. and H. Hooghiemstra 2004. "Rapid environmental change in African and South American tropics around 4000 years before present: a review". *Earth-Sci. Rev.* **66**, 217–260.
Marchitto, Thomas M., Raimund Muscheler, Joseph D. Ortiz, Jose D. Carriquiry, and Alexander van Geen. 2010. "Dynamical Response of the Tropical Pacific Ocean to Solar Forcing During the Early Holocene." *Science* 330 (6009): 1378–81. https://doi.org/10.1126/science.1194887.
Marguerie, D., and L. Visset. 1995. "Etude Palynologique Des Tourbieres de Yeun Elez, Reservoir Saint-Michel, Monts d'arree (Finistere) : Contribution a La Connaissance Du Developpement Et de La Sauvegarde Des Marais." *Conseil General Du Finistere and CNRS-UMR 6566*.
Marshall, Michael H., Henry F. Lamb, Sarah J. Davies, Melanie J. Leng, Zelalem Kubsa, Mohammed Umer, and Charlotte Bryant. 2009. "Climatic Change in Northern Ethiopia During the Past 17,000 Years: A Diatom and Stable Isotope Record from Lake Ashenge." *Palaeogeography, Palaeoclimatology, Palaeoecology* 279 (1-2): 114–27. https://doi.org/10.1016/j.palaeo.2009.05.003.
Marsicek, Jeremiah P., Bryan Shuman, Simon Brewer, David R. Foster, and W. Wyatt Oswald. 2013. "Moisture and Temperature Changes Associated with the Mid-Holocene Tsuga Decline in the Northeastern United States." *Quaternary Science Reviews* 80 (November): 129–42. https://doi.org/10.1016/j.quascirev.2013.09.001.
Marsicek, Jeremiah, Bryan N. Shuman, Patrick J. Bartlein, Sarah L. Shafer, and Simon Brewer. 2018. "Reconciling Divergent Trends and Millennial Variations in Holocene Temperatures." *Nature* 554 (7690): 92–96. https://doi.org/10.1038/nature25464.
Mart-Chivelet, Javier, M. Bel'en Muñoz-Garcand R. Lawrence Edwards, Mar. Turrero, and Ana I. Ortega. 2011. "Land Surface Temperature Changes in Northern Iberia Since 4000yrBP, Based on d13C of Speleothems." *Global and Planetary Change* 77 (1-2): 1–12. https://doi.org/10.1016/j.gloplacha.2011.02.002.
Martrat, Belen, Joan O. Grimalt, Joan Villanueva, Shirley van Kreveld, and Michael Sarnthein. 2003. "Climatic Dependence of the Organic Matter Contributions in the North Eastern Norwegian Sea over the Last 15,000 Years." *Organic Geochemistry* 34 (8): 1057–70. https://doi.org/10.1016/s0146-6380(03)00084-6.
Martrat, Belen, Patricia Jimenez-Amat, Rainer Zahn, and Joan O. Grimalt. 2014. "Similarities and Dissimilarities Between the Last Two Deglaciations and Interglaciations in the North Atlantic Region." *Quaternary Science Reviews* 99 (September): 122–34. https://doi.org/10.1016/j.quascirev.2014.06.016.
Mary, Yannick, Fr'ed'erique Eynaud, Christophe Colin, Linda Rossignol, Sandra Brocheray, Meryem Mojtahid, Jennifer Garcia, et al. 2017. "Changes in Holocene Meridional Circulation and Poleward Atlantic Flow: The Bay of Biscay as a Nodal Point." *Climate of the Past* 13 (3): 201–16. https://doi.org/10.5194/cp-13-201-2017.
Massa, Charly, Bianca B. Perren, 'Emilie Gauthier, Vincent Bichet, Christophe Petit, and Herv'e Richard. 2012. "A Multiproxy Evaluation of Holocene Environmental Change from Lake Igaliku, South Greenland." *Journal of Paleolimnology* 48 (1): 241–58. https://doi.org/10.1007/s10933-012-9594-5.

Masson, Val'erie, Françoise Vimeux, Jean Jouzel, Vin Morgan, Marc Delmotte, Philippe Ciais, Claus Hammer, et al. 2000. "Holocene Climate Variability in Antarctica Based on 11 Ice-Core Isotopic Records." *Quaternary Research* 54 (3): 348–58. https://doi.org/10.1006/qres.2000.2172.

Masson-Delmotte, V., D. Buiron, A. Ekaykin, M. Frezzotti, H. Gall'ee, J. Jouzel, G. Krinner, et al. 2011. "A Comparison of the Present and Last Interglacial Periods in Six Antarctic Ice Cores." *Climate of the Past* 7 (2): 397–423. https://doi.org/10.5194/cp-7-397-2011.

Mateus, J. E. 1992. "Holocene and Present-Day Ecosystems of the Carvalhal Region, South-West Portugal." *Doctoral Dissertation. University of Utrecht*.

Mathewes, Rolf W. 1973. "A Palynological Study of Postglacial Vegetation Changes in the University Research Forest, Southwestern British Columbia." *Canadian Journal of Botany* 51 (11): 2085–2103. https://doi.org/10.1139/b73-271.

Mathewes, Rolf W., and Linda E. Heusser. 1981a. "A 12 000 Year Palynological Record of Temperature and Precipitation Trends in Southwestern British Columbia." *Canadian Journal of Botany* 59 (5): 707–10. https://doi.org/10.1139/b81-100.

———. 1981b. "A 12000 Year Palynological Record of Temperature and Precipitation Trends in Southwestern British Columbia." *Canadian Journal of Botany* 59 (5): 707–10. https://doi.org/10.1139/b81-100.

Max, L., L. Lembke-Jene, J.-R. Riethdorf, R. Tiedemann, D. Nürnberg, H. Kühn, and A. Mackensen. 2014. "Pulses of Enhanced North Pacific Intermediate Water Ventilation from the Okhotsk Sea and Bering Sea During the Last Deglaciation." *Climate of the Past* 10 (2): 591–605. https://doi.org/10.5194/cp-10-591-2014.

McAndrews, John H. 1984. "Pollen Analysis of the 1973 Ice Core from Devon Island Glacier, Canada." *Quaternary Research* 22 (1): 68–76. https://doi.org/10.1016/0033-5894(84)90007-3.

McCarthy, F. M. G. 1986. "Late Holocene Water Levels in Lake Ontario: Evidence from Grenadier Pond." *Master's Thesis. University of Toronto*.

McCarthy, FrancineM.G., and JohnH. McAndrews. 1988. "Water Levels in Lake Ontario 4230?2000 Years b.p.: Evidence from Grenadier Pond, Toronto, Canada." *Journal of Paleolimnology* 1 (2). https://doi.org/10.1007/bf00196067.

McClymont, Erin L., Raja S. Ganeshram, Laetitia E. Pichevin, Helen M. Talbot, Bart E. van Dongen, Robert C. Thunell, Alan M. Haywood, Joy S. Singarayer, and Paul J. Valdes. 2012. "Sea-Surface Temperature Records of Termination 1 in the Gulf of California: Challenges for Seasonal and Interannual Analogues of Tropical Pacific Climate Change." *Paleoceanography* 27 (2): n/a–. https://doi.org/10.1029/2011pa002226.

McGann, Mary. 2008. "High-Resolution Foraminiferal, Isotopic, and Trace Element Records from Holocene Estuarine Deposits of San Francisco Bay, California." *Journal of Coastal Research* 245 (September): 1092–1109. https://doi.org/10.2112/08a-0003.1.

McGHEE, ROBERT, and JAMES A. TUCK. 1975. *Archaic Sequence from the Strait of Belle Isle, Labrador*. Canadian Museum of History. https://doi.org/10.2307/j.ctt22zmcws.

McGlone, Matt S., Chris S. M. Turney, Janet M. Wilmshurst, James Renwick, and Katharina Pahnke. 2010. "Divergent Trends in Land and Ocean Temperature in the Southern Ocean over the Past 18,000 Years." *Nature Geoscience* 3 (9): 622–26. https://doi.org/10.1038/ngeo931.

McGregor, Helen V., Michael N. Evans, Hugues Goosse, Guillaume Leduc, Belen Martrat, Jason A. Addison, P. Graham Mortyn, et al. 2015. "Robust Global Ocean Cooling Trend for the Pre-Industrial Common Era." *Nature Geoscience* 8 (9): 671–77. https://doi.org/10.1038/ngeo2510.

McKay, Nicholas P., and Darrell S. Kaufman. 2008. "Holocene Climate and Glacier Variability at Hallet and Greyling Lakes, Chugach Mountains, South-Central Alaska." *Journal of Paleolimnology* 41 (1): 143–59. https://doi.org/10.1007/s10933-008-9260-0.

Menounos, Brian, John J. Clague, Gerald Osborn, Brian H. Luckman, Thomas R. Lakeman, and Ryan Minkus. 2008. "Western Canadian Glaciers Advance in Concert with Climate Change Circa 4.2 Ka." *Geophysical Research Letters* 35 (7). https://doi.org/10.1029/2008gl033172.

Metcalfe, S. E., M. D. Jones, S. J. Davies, A. Noren, and A. MacKenzie. 2010a. "Climate Variability over the Last Two Millennia in the North American Monsoon Region, Recorded in Laminated Lake Sediments from Laguna de Juanacatlán, Mexico." *The Holocene* 20 (8): 1195–1206. https://doi.org/10.1177/0959683610371994.

———. 2010b. "Climate Variability over the Last Two Millennia in the North American Monsoon Region, Recorded in Laminated Lake Sediments from Laguna de Juanacatlán, Mexico." *The Holocene* 20 (8): 1195–1206. https://doi.org/10.1177/0959683610371994.

Meyer, Vera D., Lars Max, Jens Hefter, Ralf Tiedemann, and Gesine Mollenhauer. 2016. "Glacial-to-Holocene Evolution of Sea Surface Temperature and Surface Circulation in the Subarctic Northwest Pacific and the

Western Bering Sea: NW PACIFIC SST OVER THE PAST 20 KA." *Paleoceanography* 31 (7): 916–27. https://doi.org/10.1002/2015pa002877.
Mezgec, K., B. Stenni, X. Crosta, V. Masson-Delmotte, C. Baroni, M. Braida, V. Ciardini, et al. 2017. "Holocene Sea Ice Variability Driven by Wind and Polynya Efficiency in the Ross Sea." *Nature Communications* 8 (1). https://doi.org/10.1038/s41467-017-01455-x.
Michels, A., K. R. Laird, S. E. Wilson, D. Thomson, P. R. Leavitt, R. J. Oglesby, and B. F. Cumming. 2007. "Multidecadal to Millennial-Scale Shifts in Drought Conditions on the Canadian Prairies over the Past Six Millennia: Implications for Future Drought Assessment." *Global Change Biology* 13 (7): 1295–1307. https://doi.org/10.1111/j.1365-2486.2007.01367.x.
Milecka, K., and K. Szeroczynska. 2002. "Tymczasowa Informacja o Paleoekologii i Paleolimnologii Jeziora Ostrowite Na Podstawie Glebokowodnego Rdzenia (z SW Czesci Zbionika)." *Park Narodowy "Bory Tucholskie". (National Park "Bory Tucholskie")*, 61 74.
Miller, Charlotte, Annette Hahn, Diederik Liebrand, Matthias Zabel, and Enno Schefuß. 2020. "Mid- and Low Latitude Effects on Eastern South African Rainfall over the Holocene." *Quaternary Science Reviews* 229 (February): 106088. https://doi.org/10.1016/j.quascirev.2019.106088.
Minckley, T. A. 2014. "Postglacial Vegetation History of Southeastern Wyoming, u.s.a." *Rocky Mountain Geology* 49 (1): 61–74. https://doi.org/10.2113/gsrocky.49.1.61.
Minoshima, Kayo, Hodaka Kawahata, and Ken Ikehara. 2007. "Changes in Biological Production in the Mixed Water Region (MWR) of the Northwestern North Pacific During the Last 27 Kyr." *Palaeogeography, Palaeoclimatology, Palaeoecology* 254 (3-4): 430–47. https://doi.org/10.1016/j.palaeo.2007.06.022.
Miotk, G. 1986. "Badania Palinologiczne Osadow z Polnocnego Obrzeza Jeziora Godziszewskiego Kolo Tczewa/Woj." *Gdanskie Fizjograficzne Na Polska Zach* T.XXXVI: 123 135.
Miotk-Szpiganowicz, G. 1992. "The History of Vegetation of Bory Tucholskie and the Role of Man in the Light of Palynological Investigations." *Acta Paleobotanica*.
Miras, Y. 2004. "L'analyse Pollinique Du Plateau de Millevaches (Massif Central, France) Et de Sites Periferiques Limousins Et Auvergnats: Approche Des Paleoenvironnements, Des Systemes Agro-Pastoraux Et Evolution Des Territoires Ruraux." *[The Pollen Analysis of the Plateau de Millevaches (Massif Central*.
Miras, Y., and P. Guenet. 2013. "Une Histoire Plurimillenaire Des Paysages Du Cezallier Et Ses Liens Avec Les Activites Agrosylvopastorales Depuis Le Neolithique a Partir de l'analyse Pollinique de La Tourbiere de La Borie (1170 m, Saint-Saturnin, Cantal, France)." *Les Arvernes Et Leurs Vosins Du Massif Central a l'epoque Romaine*.
Mischke, Steffen, and Chengjun Zhang. 2010. "Holocene Cold Events on the Tibetan Plateau." *Global and Planetary Change* 72 (3): 155–63. https://doi.org/10.1016/j.gloplacha.2010.02.001.
Mischke, S., and B. Wünnemann. 2006. "The Holocene Salinity History of Bosten Lake (Xinjiang, China) Inferred from Ostracod Species Assemblages and Shell Chemistry: Possible Palaeoclimatic Implications." *Quaternary International* 154-155 (October): 100–112. https://doi.org/10.1016/j.quaint.2006.02.014.
Mitchell, F. J. G., and T. Cooney. 2004. "Vegetation History in the Killarney Valley." *In: Ross Island-Mining*.
Mitchell, Fraser J. G., and Edwina Cole. 1998. "Reconstruction of Long-Term Successional Dynamics of Temperate Woodland in Bialowieza Forest, Poland." *Journal of Ecology* 86 (6): 1042–59. https://doi.org/10.1046/j.1365-2745.1998.00323.x.
Mitchell, Michael Oand Frazer J. G., Peter W. Readman, Terence J. Doherty, and Declan A. Murray. 1987. "Palaeoecological Investigations Towards the Reconstruction of the Post-Glacial Environment at Lough Doo, County Mayo, Ireland." *Journal of Quaternary Science* 2 (2): 149–64. https://doi.org/10.1002/jqs.3390020208.
Moerman, Jessica W., Kim M. Cobb, Jess F. Adkins, Harald Sodemann, Brian Clark, and Andrew A. Tuen. 2013a. "Diurnal to Interannual Rainfall d18O Variations in Northern Borneo Driven by Regional Hydrology." *Earth and Planetary Science Letters* 369-370 (May): 108–19. https://doi.org/10.1016/j.epsl.2013.03.014.
———. 2013b. "Diurnal to Interannual Rainfall δ18O Variations in Northern Borneo Driven by Regional Hydrology." *Earth and Planetary Science Letters* 369–370 (May): 108–19. https://doi.org/10.1016/j.epsl.2013.03.014.
Moerman, Jessica W., Kim M. Cobb, Judson W. Partin, A. Nele Meckler, Stacy A. Carolin, Jess F. Adkins, Syria Lejau, Jenny Malang, Brian Clark, and Andrew A. Tuen. 2014a. "Transformation of ENSO-Related Rainwater to Dripwater d18O Variability by Vadose Water Mixing." *Geophysical Research Letters* 41 (22): 7907–15. https://doi.org/10.1002/2014gl061696.
———. 2014b. "Transformation of ENSO-related Rainwater to Dripwater δ18O Variability by Vadose Water Mixing." *Geophysical Research Letters* 41 (22): 7907–15. https://doi.org/10.1002/2014gl061696.

Mohtadi, Mahyar, Matthias Prange, Delia W. Oppo, Ricardo De Pol-Holz, Ute Merkel, Xiao Zhang, Stephan Steinke, and Andreas Lückge. 2014. “North Atlantic Forcing of Tropical Indian Ocean Climate.” *Nature* 509 (7498): 76–80. https://doi.org/10.1038/nature13196.

Mohtadi, Mahyar, Pamela Rossel, Carina B. Lange, Silvio Pantoja, Philipp Böning, Daniel J. Repeta, Maik Grunwald, Frank Lamy, Dierk Hebbeln, and Hans-Jürgen Brumsack. 2008. “Deglacial Pattern of Circulation and Marine Productivity in the Upwelling Region Off Central-South Chile.” *Earth and Planetary Science Letters* 272 (1-2): 221–30. https://doi.org/10.1016/j.epsl.2008.04.043.

Mohtadi, Mahyar, Stephan Steinke, Andreas Lückge, Jeroen Groeneveld, and Ed C. Hathorne. 2010. “Glacial to Holocene Surface Hydrography of the Tropical Eastern Indian Ocean.” *Earth and Planetary Science Letters* 292 (1-2): 89–97. https://doi.org/10.1016/j.epsl.2010.01.024.

Mollier-Vogel, Elfi, Guillaume Leduc, Tebke Böschen, Philippe Martinez, and Ralph R. Schneider. 2013. “Rainfall Response to Orbital and Millennial Forcing in Northern Peru over the Last 18 Ka.” *Quaternary Science Reviews* 76 (September): 29–38. https://doi.org/10.1016/j.quascirev.2013.06.021.

Moossen, Heiko, James Bendle, Osamu Seki, Ursula Quillmann, and Kimitaka Kawamura. 2015. “North Atlantic Holocene Climate Evolution Recorded by High-Resolution Terrestrial and Marine Biomarker Records.” *Quaternary Science Reviews* 129 (December): 111–27. https://doi.org/10.1016/j.quascirev.2015.10.013.

Morales-Molino, C’esar, Daniele Colombaroli, Mar-Carabaña, Willy Tinner, Roberto L. Salom’on, Jos’e S. Carri’on, and Luis Gil. 2017. “Land-Use History as a Major Driver for Long-Term Forest Dynamics in the Sierra de Guadarrama National Park (Central Spain) During the Last Millennia: Implications for Forest Conservation and Management.” *Global and Planetary Change* 152 (May): 64–75. https://doi.org/10.1016/j.gloplacha.2017.02.012.

Morales-Molino, C’esar, Mercedes Garc-Ant’on, Jos’e M. Postigo-Mijarra, and Carlos Morla. 2013. “Holocene Vegetation, Fire and Climate Interactions on the Westernmost Fringe of the Mediterranean Basin.” *Quaternary Science Reviews* 59 (January): 5–17. https://doi.org/10.1016/j.quascirev.2012.10.027.

Morales-Molino, C’esar, Willy Tinner, Mercedes Garc-Ant’on, and Daniele Colombaroli. 2016. “The Historical Demise of <i>pinus Nigra</i> Forests in the Northern Iberian Plateau (South-Western Europe).” Edited by Matt McGlone. *Journal of Ecology* 105 (3): 634–46. https://doi.org/10.1111/1365-2745.12702.

Moreno, Ana, Carlos P’erez-Mejand Miguel Bartolom’e, Carlos Sancho, Isabel Cacho, Heather Stoll, Antonio Delgado-Huertas, John Hellstrom, R. Lawrence Edwards, and Hai Cheng. 2017. “New Speleothem Data from Molinos and Ejulve Caves Reveal Holocene Hydrological Variability in Northeast Iberia.” *Quaternary Research* 88 (2): 223–33. https://doi.org/10.1017/qua.2017.39.

Morrill, Carrie, Jonathan T. Overpeck, Julia E. Cole, Kam-biu Liu, Caiming Shen, and Lingyu Tang. 2006. “Holocene Variations in the Asian Monsoon Inferred from the Geochemistry of Lake Sediments in Central Tibet.” *Quaternary Research* 65 (02): 232–43. https://doi.org/10.1016/j.yqres.2005.02.014.

Morris, Jesse L., Andrea Brunelle, R. Justin DeRose, Heikki Seppä, Mitchell J. Power, Vachel Carter, and Ryan Bares. 2013. “Using Fire Regimes to Delineate Zones in a High-Resolution Lake Sediment Record from the Western United States.” *Quaternary Research* 79 (1): 24–36. https://doi.org/10.1016/j.yqres.2012.10.002.

Mosley-Thompson, Ellen. 1996. “Holocene Climate Changes Recorded in an East Antarctica Ice Core.” In *Climatic Variations and Forcing Mechanisms of the Last 2000 Years*, 263–79. Springer Berlin Heidelberg. https://doi.org/10.1007/978-3-642-61113-1_13.

Mulitza, Stefan, Cristiano M. Chiessi, Enno Schefuß, Jörg Lippold, David Wichmann, Benny Antz, Andreas Mackensen, et al. 2017. “Synchronous and Proportional Deglacial Changes in Atlantic Meridional Overturning and Northeast Brazilian Precipitation.” *Paleoceanography* 32 (6): 622–33. https://doi.org/10.1002/2017pa003084.

Mulvaney, Robert, Nerilie J. Abram, Richard C. A. Hindmarsh, Carol Arrowsmith, Louise Fleet, Jack Triest, Louise C. Sime, Olivier Alemany, and Susan Foord. 2012. “Recent Antarctic Peninsula Warming Relative to Holocene Climate and Ice-Shelf History.” *Nature* 489 (7414): 141–44. https://doi.org/10.1038/nature11391.

Muñoz, Arsenio, Miguel Bartolom’e, Alicia Muñoz, Carlos Sancho, Ana Moreno, John C. Hellstrom, M Cinta Os’acar, and Isabel Cacho. 2015a. “Solar Influence and Hydrological Variability During the Holocene from a Speleothem Annual Record (Molinos Cave, NE Spain).” *Terra Nova* 27 (4): 300–311. https://doi.org/10.1111/ter.12160.

———. 2015b. “Solar Influence and Hydrological Variability During the Holocene from a Speleothem Annual Record (Molinos Cave, <Scp>NE</Scp> Spain).” *Terra Nova* 27 (4): 300–311. https://doi.org/10.1111/ter.12160.

Myers, Christopher G., Jessica L. Oster, Warren D. Sharp, Ralf Bennartz, Neil P. Kelley, Aaron K. Covey, and Sebastian F. M. Breitenbach. 2015. "Northeast Indian Stalagmite Records Pacific Decadal Climate Change: Implications for Moisture Transport and Drought in India." *Geophysical Research Letters* 42 (10): 4124–32. https://doi.org/10.1002/2015gl063826.

Nakagawa, T. 1998. "Etudes Palynologiques Dans Les Alpes Francaises Centrales Et Meridionales: Histoire de La Vegetation Tardiglaciaire Et Holocene [Pollen Studies in the Central Meridional French Alps: Lateglacial and Holocene Vegetation History]." *Doctoral Dissertation. Universite d'Aix-Marseille*.

Nakamura, Atsunori, Yusuke Yokoyama, Hideaki Maemoku, Hiroshi Yagi, Makoto Okamura, Hiromi Matsuoka, Nao Miyake, et al. 2016a. "Weak Monsoon Event at 4.2 Ka Recorded in Sediment from Lake Rara, Himalayas." *Quaternary International* 397 (March): 349–59. https://doi.org/10.1016/j.quaint.2015.05.053.

———, et al. 2016b. "Weak Monsoon Event at 4.2 Ka Recorded in Sediment from Lake Rara, Himalayas." *Quaternary International* 397 (March): 349–59. https://doi.org/10.1016/j.quaint.2015.05.053.

?NANANA. 2010. "南海北部晚第四纪高分辨率浮游氧同位素地层学及其古气候信息." *Earth Science-Journal of China University of Geosciences* 35 (4): 515. https://doi.org/10.3799/dqkx.2010.067.

Nazarova, Larisa, Annette Bleibtreu, Ulrike Hoff, Veronika Dirksen, and Bernhard Diekmann. 2017. "Changes in Temperature and Water Depth of a Small Mountain Lake During the Past 3000 Years in Central Kamchatka Reflected by a Chironomid Record." *Quaternary International* 447 (August): 46–58. https://doi.org/10.1016/j.quaint.2016.10.008.

Neil, Karen, Konrad Gajewski, and Matthew Betts. 2014. "Human-Ecosystem Interactions in Relation to Holocene Environmental Change in Port Joli Harbour, Southwestern Nova Scotia, Canada." *Quaternary Research* 81 (2): 203–12. https://doi.org/10.1016/j.yqres.2014.01.001.

Nelson, Daniel B., Mark B. Abbott, Byron Steinman, Pratigya J. Polissar, Nathan D. Stansell, Joseph D. Ortiz, Michael F. Rosenmeier, Bruce P. Finney, and Jon Riedel. 2011. "Drought Variability in the Pacific Northwest from a 6,000-Yr Lake Sediment Record." *Proceedings of the National Academy of Sciences* 108 (10): 3870–75. https://doi.org/10.1073/pnas.1009194108.

Nelson, David M., and Feng Sheng Hu. 2008a. "Patterns and Drivers of Holocene Vegetational Change Near the Prairie-Forest Ecotone in Minnesota: Revisiting McAndrews' Transect." *New Phytologist* 179 (2): 449–59. https://doi.org/10.1111/j.1469-8137.2008.02482.x.

———. 2008b. "Patterns and Drivers of Holocene Vegetational Change Near the Prairie–Forest Ecotone in Minnesota: Revisiting McAndrews' Transect." *New Phytologist* 179 (2): 449–59. https://doi.org/10.1111/j.1469-8137.2008.02482.x.

Nesje, Atle, John A. Matthews, Svein Olaf Dahl, Mark S. Berrisford, and Carin Andersson. 2001. "Holocene Glacier Fluctuations of Flatebreen and Winter-Precipitation Changes in the Jostedalsbreen Region, Western Norvay, Based on Glaciolacustrine Sediment Records." *The Holocene* 11 (3): 267–80. https://doi.org/10.1191/095968301669980885.

Neugebauer, Ina, Mich'ele Dinies, Birgit Plessen, Nadine Dräger, Achim Brauer, Helmut Brückner, Peter Frenzel, et al. 2022. "The Unexpectedly Short Holocene Humid Period in Northern Arabia." *Communications Earth &Amp; Environment* 3 (1). https://doi.org/10.1038/s43247-022-00368-y.

Newby, Paige E., Bryan N. Shuman, Jeffrey P. Donnelly, Kristopher B. Karnauskas, and Jeremiah Marsicek. 2014. "Centennial-to-Millennial Hydrologic Trends and Variability Along the North Atlantic Coast, USA, During the Holocene." *Geophysical Research Letters* 41 (12): 4300–4307. https://doi.org/10.1002/2014gl060183.

Newby, Paige E., Bryan N. Shuman, Jeffrey P. Donnelly, and Dana MacDonald. 2011. "Repeated Century-Scale Droughts over the Past 13,000 Yr Near the Hudson River Watershed, USA." *Quaternary Research* 75 (3): 523–30. https://doi.org/10.1016/j.yqres.2011.01.006.

Nguyen, Shawn Oand Thi Mai Huong, Christopher Stimpson, Rachael Holmes, Thorsten Kahlert, Evan Hill, Thuy Vo, and Ryan Rabet. 2020. "Holocene Development and Human Use of Mangroves and Limestone Forest at an Ancient Hong Lagoon in the Tràng an Karst, Ninh Binh, Vietnam." *Quaternary Science Reviews* 242 (August): 106416. https://doi.org/10.1016/j.quascirev.2020.106416.

Nichols, H. 1975. "Palynological and Paleoclimatic Study of the Late Quaternary Displacements of the Boreal Forest-Tundra Ecotone in Keewatin and Mackenzie, n.w.t., Canada." *Occasional Paper 15. Institute of Arctic and Alpine Research*.

Nichols, Johanna. 2021. "The Origin and Dispersal of Uralic: Distributional Typological View." *Annual Review of Linguistics* 7 (1): 351–69. https://doi.org/10.1146/annurev-linguistics-011619-030405.

Nicol-Pichard, Sylvie, and Michel Dubar. 1998. "Reconstruction of Late-Glacial and Holocene Environments in Southeast France Based on the Study of a 66-m Long Core from Biot, Alpes Maritimes." *Vegetation History and Archaeobotany* 7 (1): 11–15. https://doi.org/10.1007/bf01393413.
Niedermeyer, Eva M., Alex L. Sessions, Sarah J. Feakins, and Mahyar Mohtadi. 2014. "Hydroclimate of the Western Indo-Pacific Warm Pool During the Past 24,000 Years." *Proceedings of the National Academy of Sciences* 111 (26): 9402–6. https://doi.org/10.1073/pnas.1323585111.
Nielsen, Simon H. H., Nalân Koç, and Xavier Crosta. 2004. "Holocene Climate in the Atlantic Sector of the Southern Ocean: Controlled by Insolation or Oceanic Circulation?" *Geology* 32 (4): 317. https://doi.org/10.1130/g20334.1.
Niggemann, Stefan, Augusto Mangini, Manfred Mudelsee, Detlev K Richter, and Georg Wurth. 2003. "Sub-Milankovitch Climatic Cycles in Holocene Stalagmites from Sauerland, Germany." *Earth and Planetary Science Letters* 216 (4): 539–47. https://doi.org/10.1016/s0012-821x(03)00513-2.
Niinemets, Eve, and Leili Saarse. 2006. "Holocene Forest Dynamics and Human Impact in Southeastern Estonia." *Vegetation History and Archaeobotany* 16 (1): 1–13. https://doi.org/10.1007/s00334-005-0030-x.
Nilsson, T. 1964. "Standard Pollen Diagramme Und C14 Datiengen Aus Dem Agerods Mosse in Mittleren Schonen." *Lunds Universitets Arsskrift N. F.*
Noryskiewicz, B. 1995. "Zmiany Szaty Roslinnej Okolic Jeziora Biskupinskiego Pod Wplywem Czynnikow Naturalnych i Antropogenicznych w Poznym Glacjale i Holocenie [Changes in Vegetation of the Biskupin (Biskupinskie) Lake Area During the Late-Glacial and the Holocene, Caused by Natural and Anthropogenic Factors]." *Unknown*, 147 180.
Nov'ak, Jan, Lenka Lis'a, Petr Pokor ý, and Martin Kuna. 2012. "Charcoal Analyses as an Environmental Tool for the Study of Early Medieval Sunken Houses Infills in Roztoky Near Prague, Czech Republic." *Journal of Archaeological Science* 39 (4): 808–17. https://doi.org/10.1016/j.jas.2011.06.026.
Novello, V. F., F. W. Cruz, J. S. Moquet, M. Vuille, M. S. de Paula, D. Nunes, R. L. Edwards, et al. 2018. "Two Millennia of South Atlantic Convergence Zone Variability Reconstructed from Isotopic Proxies." *Geophysical Research Letters* 45 (10): 5045–51. https://doi.org/10.1029/2017gl076838.
Novello, Valdir F., Francisco W. Cruz, Mathias Vuille, Nicol'as M. Strand R. Lawrence Edwards, Hai Cheng, Suellyn Emerick, Marcos S. de Paula, et al. 2017. "A High-Resolution History of the South American Monsoon from Last Glacial Maximum to the Holocene." *Scientific Reports* 7 (1). https://doi.org/10.1038/srep44267.
Nürnberg, Dirk, Tebke Böschen, Kristin Doering, Elfi MollierNANAVogel, Jacek Raddatz, and Ralph Schneide. 2015. "Sea Surface and Subsurface Circulation Dynamics Off Equatorial Peru During the Last 17 Kyr." *Paleoceanography* 30 (7): 984–99. https://doi.org/10.1002/2014pa002706.
Nürnberg, Dirk, Tebke Böschen, Kristin Doering, Elfi Mollier-Vogel, Jacek Raddatz, and Ralph Schneider. 2015. "Sea Surface and Subsurface Circulation Dynamics Off Equatorial Peru During the Last 17kyr." *Paleoceanography* 30 (7): 984–99. https://doi.org/10.1002/2014pa002706.
Nürnberg, Dirk, Tabitha Riff, Andr'e Bahr, Cyrus Karas, Karl Meier, and Jörg Lippold. 2021. "Western Boundary Current in Relation to Atlantic Subtropical Gyre Dynamics During Abrupt Glacial Climate Fluctuations." *Global and Planetary Change* 201 (June): 103497. https://doi.org/10.1016/j.gloplacha.2021.103497.
Ocakoğlu, Faruk, Çiler Çilingiroğlu, İsmühan Potoğlu Erkara, Serdar Ünan, Berkay Dinçer, and Mehmet Serkan Akkiraz. 2019. "Human-Climate Interactions Since the Neolithic Period in Central Anatolia: Novel Multi-Proxy Data from the Kureyşler Area, Kütahya, Turkey." *Quaternary Science Reviews* 213 (June): 1–17. https://doi.org/10.1016/j.quascirev.2019.04.016.
Oeggl, K. 1988. "Beitrage Zur Vegetationsgeschichte Tirols VII: Das Hochmoor Schwemm Bei Walchsee." *Berichte Des Naturwissenschaftlich-Medizinischen Vereins in Innsbruck.*
Oehlerich, M., C. Mayr, N. Gussone, A. Hahn, S. Hölzl, A. Lücke, C. Ohlendorf, S. Rummel, B. M. A. Teichert, and B. Zolitschka. 2015. "Lateglacial and Holocene Climatic Changes in South-Eastern Patagonia Inferred from Carbonate Isotope Records of Laguna Potrok Aike (Argentina)." *Quaternary Science Reviews* 114 (April): 189–202. https://doi.org/10.1016/j.quascirev.2015.02.006.
Ohlendorf, C., M. Fey, J. Massaferro, T. Haberzettl, C. Laprida, A. Lücke, N. Maidana, et al. 2014. "Late Holocene Hydrology Inferred from Lacustrine Sediments of Laguna Cháltel (Southeastern Argentina)." *Palaeogeography, Palaeoclimatology, Palaeoecology* 411 (October): 229–48. https://doi.org/10.1016/j.palaeo.2014.06.030.
'Olafsd'ottir, Sæd'ıs, Anne E. Jennings, 'Aslaug Geirsd'ottir, John Andrews, and Gifford H. Miller. 2010. "Holocene Variability of the North Atlantic Irminger Current on the South- and Northwest Shelf of Iceland." *Marine Micropaleontology* 77 (3–4): 101–18. https://doi.org/10.1016/j.marmicro.2010.08.002.

Olsen, Jesper, Svante Björck, Melanie J. Leng, Esther Ruth Gudmundsd'ottir, Bent V. Odgaard, Christina M. Lutz, Chris P. Kendrick, Thorbjørn J. Andersen, and Marit-Solveig Seidenkrantz. 2010. "Lacustrine Evidence of Holocene Environmental Change from Three Faroese Lakes: A Multiproxy XRF and Stable Isotope Study." *Quaternary Science Reviews* 29 (19-20): 2764–80. https://doi.org/10.1016/j.quascirev.2010.06.029.

Ön, Z. Bora, Sena Akçer-Ön, M. Sinan Özeren, K. Kadir Eriş, Alan M. Greaves, and M. Namık Çağatay. 2018a. "Climate Proxies for the Last 17.3 Ka from Lake Hazar (Eastern Anatolia), Extracted by Independent Component Analysis of μ -XRF Data." *Quaternary International* 486 (August): 17–28. https://doi.org/10.1016/j.quaint.2017.08.066.

———. 2018b. "Climate Proxies for the Last 17.3 Ka from Lake Hazar (Eastern Anatolia), Extracted by Independent Component Analysis of μ-XRF Data." *Quaternary International* 486 (August): 17–28. https://doi.org/10.1016/j.quaint.2017.08.066.

Orland, Ian J., Miryam Bar-Matthews, Avner Ayalon, Alan Matthews, Reinhard Kozdon, Takayuki Ushikubo, and John W. Valley. 2012. "Seasonal Resolution of Eastern Mediterranean Climate Change Since 34ka from a Soreq Cave Speleothem." *Geochimica Et Cosmochimica Acta* 89 (July): 240–55. https://doi.org/10.1016/j.gca.2012.04.035.

Orland, Ian J., Miryam Bar-Matthews, Noriko T. Kita, Avner Ayalon, Alan Matthews, and John W. Valley. 2009. "Climate Deterioration in the Eastern Mediterranean as Revealed by Ion Microprobe Analysis of a Speleothem That Grew from 2.2 to 0.9 Ka in Soreq Cave, Israel." *Quaternary Research* 71 (1): 27–35. https://doi.org/10.1016/j.yqres.2008.08.005.

Oswald, W. Wyatt, David R. Foster, Bryan N. Shuman, Elaine D. Doughty, Edward K. Faison, Brian R. Hall, Barbara C. S. Hansen, Matts Lindbladh, Adriana Marroquin, and Sarah A. Truebe. 2018. "Subregional Variability in the Response of New England Vegetation to Postglacial Climate Change." *Journal of Biogeography* 45 (10): 2375–88. https://doi.org/10.1111/jbi.13407.

Ouellet-Bernier, Marie-Mich'ele, Anne de Vernal, Claude Hillaire-Marcel, and Matthias Moros. 2014. "Paleoceanographic Changes in the Disko Bugt Area, West Greenland, During the Holocene." *The Holocene* 24 (11): 1573–83. https://doi.org/10.1177/0959683614544060.

Ouguerram, A. 2002. "Histoire de La Vallee de l'erdre (Affluent de La Loire, Massif Armoricain, France) de La Fin Du Tardiglaciaire Aux Epoques Actuelles." *Doctoral Dissertation. Universite Moulay Ismail (Maroc) Et Universite de Nantes (France)*.

Pahnke, Katharina, and Julian P. Sachs. 2006. "Sea Surface Temperatures of Southern Midlatitudes 0-160 Kyr b.p." *Paleoceanography* 21 (2): n/a–. https://doi.org/10.1029/2005pa001191.

Panova, N. K. 1990. "Novye Dannye Po Paleoekologii i Istorii Rastitelnosti Yuzhnogo Yamala v Golotsene [New Data for Paleoecology and Vegetation History of Southern Yamal During the Holocene]." *In: Chetvertichnyi Period: Metody Issledovania*.

Paquette, Nathalie, and Konrad Gajewski. 2013. "Climatic Change Causes Abrupt Changes in Forest Composition, Inferred from a High-Resolution Pollen Record, Southwestern Quebec, Canada." *Quaternary Science Reviews* 75 (September): 169–80. https://doi.org/10.1016/j.quascirev.2013.06.007.

Park, Jinheum, Jungjae Park, Sangheon Yi, Jaesoo Lim, Jin Cheul Kim, Qiuhong Jin, and Jieun Choi. 2021. "Holocene Hydroclimate Reconstruction Based on Pollen, XRF, and Grain-Size Analyses and Its Implications for Past Societies of the Korean Peninsula." *The Holocene* 31 (9): 1489–1500. https://doi.org/10.1177/09596836211019115.

Parrenin, F., J.-M. Barnola, J. Beer, T. Blunier, E. Castellano, J. Chappellaz, G. Dreyfus, et al. 2007. "The EDC3 Chronology for the EPICA Dome c Ice Core." *Climate of the Past* 3 (3): 485–97. https://doi.org/10.5194/cp-3-485-2007.

Parrenin, F., G. Dreyfus, G. Durand, S. Fujita, O. Gagliardini, F. Gillet, J. Jouzel, et al. 2007. "1-d-Ice Flow Modelling at EPICA Dome c and Dome Fuji, East Antarctica." *Climate of the Past* 3 (2): 243–59. https://doi.org/10.5194/cp-3-243-2007.

Parshall, T., and D. R. Foster. 2002. "Fire on the New England Landscape: Regional and Temporal Variation, Cultural and Environmental Controls." *Journal of Biogeography* 29 (10-11): 1305–17. https://doi.org/10.1046/j.1365-2699.2002.00758.x.

Partin, Judson W., Kim M. Cobb, Jess F. Adkins, Brian Clark, and Diego P. Fernandez. 2007. "Millennial-Scale Trends in West Pacific Warm Pool Hydrology Since the Last Glacial Maximum." *Nature* 449 (7161): 452–55. https://doi.org/10.1038/nature06164.

Partin, Judson W., Kim M. Cobb, Jess F. Adkins, Andrew A. Tuen, and Brian Clark. 2013. "Trace Metal and Carbon Isotopic Variations in Cave Dripwater and Stalagmite Geochemistry from Northern Borneo." *Geochemistry, Geophysics, Geosystems* 14 (9): 3567–85. https://doi.org/10.1002/ggge.20215.

Paus, Aage. 2009. "Vegetation and Environment of the rødalen Alpine Area, Central Norway, with Emphasis on the Early Holocene." *Vegetation History and Archaeobotany* 19 (1): 29–51. https://doi.org/10.1007/s00334-009-0228-4.

Paus, Aage, Gaute Velle, and Jan Berge. 2011. "The Lateglacial and Early Holocene Vegetation and Environment in the Dovre Mountains, Central Norway, as Signalled in Two Lateglacial Nunatak Lakes." *Quaternary Science Reviews* 30 (13-14): 1780–96. https://doi.org/10.1016/j.quascirev.2011.04.010.

Peckover, E. N., J. E. Andrews, M. R. Leeder, P. J. Rowe, A. Marca, D. Sahy, S. Noble, and R. Gawthorpe. 2019a. "Coupled Stalagmite - Alluvial Fan Response to the 8.2 Ka Event and Early Holocene Palaeoclimate Change in Greece." *Palaeogeography, Palaeoclimatology, Palaeoecology* 532 (October): 109252. https://doi.org/10.1016/j.palaeo.2019.109252.

———. 2019b. "Coupled Stalagmite – Alluvial Fan Response to the 8.2 Ka Event and Early Holocene Palaeoclimate Change in Greece." *Palaeogeography, Palaeoclimatology, Palaeoecology* 532 (October): 109252. https://doi.org/10.1016/j.palaeo.2019.109252.

Pelejero, Carles, Joan O. Grimalt, Stephanie Heilig, Markus Kienast, and Luejiang Wang. 1999. "High-Resolution u^k₃₇temperature Reconstructions in the South China Sea over the Past 220 Kyr." *Paleoceanography* 14 (2): 224–31. https://doi.org/10.1029/1998pa900015.

Penalba, M. C. 1989. "Dynamique de Vegetation Tardiglaciaire Et Holocene Du Centre-Nord de l'espagne d'apres l'analyse Pollinique." *Doctoral Dissertation. Universite d'Aix-Marseille*.

Pennington, W. 1977. "The Late Devensian Flora and Vegetation of Britain." *Philosophical Transactions of the Royal Society of London. B*.

Pennington, W, E. Y Haworth, A. P. Bonny, and J. P. Lishman. 1972. "Lake Sediments in Northern Scotland." *Philosophical Transactions of the Royal Society of London. B*.

Peros, Matthew C., and Konrad Gajewski. 2008. "Pollen-Based Reconstructions of Late Holocene Climate from the Central and Western Canadian Arctic." *Journal of Paleolimnology* 41 (1): 161–75. https://doi.org/10.1007/s10933-008-9256-9.

Perren, Bianca B., N. John Anderson, Marianne S. V. Douglas, and Sherilyn C. Fritz. 2012. "The Influence of Temperature, Moisture, and Eolian Activity on Holocene Lake Development in West Greenland." *Journal of Paleolimnology* 48 (1): 223–39. https://doi.org/10.1007/s10933-012-9613-6.

Peteet, D., A. Andreev, W. Bardeen, and F. Mistretta. 2008. "Long-Term Arctic Peatland Dynamics, Vegetation and Climate History of the Pur-Taz Region, Western Siberia." *Boreas* 27 (2): 115–26. https://doi.org/10.1111/j.1502-3885.1998.tb00872.x.

Petersen, K. L. 1985. "Palynology in Montezuma County, Southwestern Colorado: The Local History of Pinyon Pine (Pinus Edulis)." *Unknown*, 47 62.

———. 1988. "Climate and the Dolores River Anasazi: A Paleoenvironmental Reconstruction from a 10,000-Year Pollen Record, La Plata Mountains, Southwestern Colorado." *University of Utah*.

Pirrus, R, A. M. Rouk, and A. Liiva. 1987. "Geology and Stratigraphy of the Reference Site of Lake Raigastvere in Saadjarv Drumlin Field." *In: Palaeohydrology of the Temperate Zone II. Lakes Ed. By A. Raukas and L. Saarse (Pp.101-122). Valgus*.

Pleskot, Krzysztof, Karina Apolinarska, Piotr Kołaczek, Magdalena Suchora, Michał Fojutowski, Tomasz Joniak, Bartosz Kotrys, et al. 2020. "Searching for the 4.2 Ka Climate Event at Lake Spore, Poland." *CATENA* 191 (August): 104565. https://doi.org/10.1016/j.catena.2020.104565.

Plunkett, Gill, Faye Carroll, Barrie Hartwell, Nicki J. Whitehouse, and Paula J. Reimer. 2008. "Vegetation History at the Multi-Period Prehistoric Complex at Ballynahatty, Co. Down, Northern Ireland." *Journal of Archaeological Science* 35 (1): 181–90. https://doi.org/10.1016/j.jas.2007.03.006.

Plunkett, Gill, Sarah E. Coulter, Vera V. Ponomareva, Maarten Blaauw, Andrea Klimaschewski, and Dan Hammarlund. 2015. "Distal Tephrochronology in Volcanic Regions: Challenges and Insights from Kamchatkan Lake Sediments." *Global and Planetary Change* 134 (November): 26–40. https://doi.org/10.1016/j.gloplacha.2015.04.006.

Pokorny, P. 2005. "Role of Man in the Development of Holocene Vegetation in Central Bohemia." *Preslia*.

Pompeani, D. P., B. A. Steinman, and M. B. Abbott. 2012. "A Sedimentary and Geochemical Record of Water-Level Changes from Rantin Lake, Yukon, Canada." *Journal of Paleolimnology* 48 (1): 147–58. https://doi.org/10.1007/s10933-012-9602-9.

Poore, R. Z., H. J. Dowsett, S. Verardo, and T. M. Quinn. 2003. "Millennial- to Century-Scale Variability in Gulf of Mexico Holocene Climate Records." *Paleoceanography* 18 (2): n/a–. https://doi.org/10.1029/2002pa000868.

Poore, R. Z., M. J. Pavich, and H. D. Grissino-Mayer. 2005. "Record of the North American Southwest Monsoon from Gulf of Mexico Sediment Cores." *Geology* 33 (3): 209. https://doi.org/10.1130/g21040.1.
Poore, R. Z., S. Verardo, J. Caplan, K. Pavich, and T. Quinn. 2009. "Planktic Foraminiferal Relative Abundance and Trends in Gulf of Mexico Holocene Sediments Records of Climate Variability." *Gulf of Mexico Origin, Waters, and Biota: Volume III, Geology*, 367–79.
Praetorius, S. K., A. C. Mix, M. H. Walczak, M. D. Wolhowe, J. A. Addison, and F. G. Prahl. 2015. "North Pacific Deglacial Hypoxic Events Linked to Abrupt Ocean Warming." *Nature* 527 (7578): 362–66. https://doi.org/10.1038/nature15753.
Praetorius, Summer K., Jerry F. McManus, Delia W. Oppo, and William B. Curry. 2008. "Episodic Reductions in Bottom-Water Currents Since the Last Ice Age." *Nature Geoscience* 1 (7): 449–52. https://doi.org/10.1038/ngeo227.
Pribyl, Paul, and Bryan N. Shuman. 2014. "A Computational Approach to Quaternary Lake-Level Reconstruction Applied in the Central Rocky Mountains, Wyoming, USA." *Quaternary Research* 82 (1): 249–59. https://doi.org/10.1016/j.yqres.2014.01.012.
Priore, T. M. Del. 2007. "4,000 Years of Environmental Change in Central Colorado: A Paleoecological Perspective." *Master's Thesis. University of Colorado*.
Prokopenko, Alexander A., Mikhail I. Kuzmin, Douglas F. Williams, Vladimir F. Gelety, Gennady V. Kalmychkov, Alexander N. Gvozdkov, and Pavel A. Solotchin. 2005. "Basin-Wide Sedimentation Changes and Deglacial Lake-Level Rise in the Hovsgol Basin, NW Mongolia." *Quaternary International* 136 (1): 59–69. https://doi.org/10.1016/j.quaint.2004.11.008.
Psomiadis, D., E. Dotsika, K. Albanakis, B. Ghaleb, and C. Hillaire-Marcel. 2018. "Speleothem Record of Climatic Changes in the Northern Aegean Region (Greece) from the Bronze Age to the Collapse of the Roman Empire." *Palaeogeography, Palaeoclimatology, Palaeoecology* 489 (January): 272–83. https://doi.org/10.1016/j.palaeo.2017.10.021.
Puertas, O. 1997. "Evolution de La Vegetation Depuis Le Dryas Recent Dans La Plaine Littorale de Montpellier (Herault, France) a Partir de l'analyse Pollinique." *Dynamique Naturelle Et Anthropisation Du Milieu. Doctoral Dissertation. Universite de Franche-Comte*.
Pulido, M. 2006. "Consequenses de l'anthropisation Sur La Dynamique Postglaciaire de La Vegetation Dans Le Sud Du Massif Central, France." *[Anthropogenic Impact on the Postglacial Vegetation Dynamics in South Massif Central*.
Punning, Jaan-Mati, Mati Ilomets, and Tiiu Koff. 1993. "Possibilities for Detailed Dating of Peat Bog Deposits." *Radiocarbon* 35 (3): 379–85. https://doi.org/10.1017/s0033822200060380.
PUNNING, JAAN-MATI, THU KOFF, MATI ILOMETS, and JAAN JOTGI. 2008. "The Relative Influence of Local, Extra-Local, and Regional Factors on Organic Sedimentation in the Vällamäe Kettle Hole, Estonia." *Boreas* 24 (1): 65–80. https://doi.org/10.1111/j.1502-3885.1995.tb00628.x.
Railsback, L. Bruce, Fuyuan Liang, G. A. Brook, Ny Riavo G. Voarintsoa, Hillary R. Sletten, Eugene Marais, Ben Hardt, Hai Cheng, and R. Lawrence Edwards. 2018. "The Timing, Two-Pulsed Nature, and Variable Climatic Expression of the 4.2 Ka Event: A Review and New High-Resolution Stalagmite Data from Namibia." *Quaternary Science Reviews* 186 (April): 78–90. https://doi.org/10.1016/j.quascirev.2018.02.015.
Railsback, L. Bruce, Fuyuan Liang, George A. Brook, Hai Cheng, and R. Lawrence Edwards. 2022. "Additional Multi-Proxy Stalagmite Evidence from Northeast Namibia Supports Recent Models of Wetter Conditions During the 4.2 Ka Event in the Southern Hemisphere." *Palaeogeography, Palaeoclimatology, Palaeoecology* 586 (January): 110756. https://doi.org/10.1016/j.palaeo.2021.110756.
Railsback, L. Bruce, Fuyuan Liang, Juan Ram'on Vidal Roma ı, Aurora Grandal-dand Marcos Vaqueiro Rodrand Luisa Santos Fidalgo, Daniel Fern'andez Mosquera, Hai Cheng, and R. Lawrence Edwards. 2011. "Petrographic and Isotopic Evidence for Holocene Long-Term Climate Change and Shorter-Term Environmental Shifts from a Stalagmite from the Serra Do Courel of Northwestern Spain, and Implications for Climatic History Across Europe and the Mediterranean." *Palaeogeography, Palaeoclimatology, Palaeoecology* 305 (1-4): 172–84. https://doi.org/10.1016/j.palaeo.2011.02.030.
Rainville, Rebecca A., and Konrad Gajewski. 2013. "Holocene Environmental History of the Aishihik Region, Yukon, Canada." Edited by Timothy G. Fisher. *Canadian Journal of Earth Sciences* 50 (4): 397–405. https://doi.org/10.1139/cjes-2012-0103.
Ralska-Jasiewiczowa, M. 1989. "Type Region p-e: The Bieszczady Mountains." *Acta Palaeobotanica*.
Ran, Min, and Liang Chen. 2019. "The 4.2 Ka BP Climatic Event and Its Cultural Responses." *Quaternary International* 521 (June): 158–67. https://doi.org/10.1016/j.quaint.2019.05.030.

Rankama, T., and I. Vuorela. 1988. “Between Inland and Coast in Metal Age Finland-Human Impact on the Primeval Forests of Southern Hame During the Iron Age.” *Memoranda Societatis Pro Fauna Et Flora Fennica*.
Rao, Zhiguo, Chao Huang, Luhua Xie, Fuxi Shi, Yan Zhao, Jiantao Cao, Xiaohua Gou, Jianhui Chen, and Fahu Chen. 2019. “Long-Term Summer Warming Trend During the Holocene in Central Asia Indicated by Alpine Peat α-Cellulose d13C Record.” *Quaternary Science Reviews* 203 (January): 56–67. https://doi.org/10.1016/j.quascirev.2018.11.010.
Rasmussen, K. A., R. D. Ricketts, T. C. Johnson, V. V. Romanovsky, and O. M. Grigina. 2001. “An 8,000 Year Multi-Proxy Record from Lake Issyk-Kul, Kyrgyzstan.” *PAGES News* 9 (2): 5–6. https://doi.org/10.22498/pages.9.2.5.
Rasmussen, KA, RD Ricketts, TC Johnson, VV Romanovsky, and OM Grigina. 2001. “An 8,000 Year Multi–Proxy Record from Lake Issyk–Kul, Kyrgyzstan.” *PAGES News* 9 (2): 5–6. https://doi.org/10.22498/pages.9.2.5.
Rasmussen, S. O., P. M. Abbott, T. Blunier, A. J. Bourne, E. Brook, S. L. Buchardt, C. Buizert, et al. 2013. “A First Chronology for the North Greenland Eemian Ice Drilling (NEEM) Ice Core.” *Climate of the Past* 9 (6): 2713–30. https://doi.org/10.5194/cp-9-2713-2013.
Ravindra, R. 2009. “A High-Resolution Vegetation, Fire, and Climate History from the Aishihik Region, Yukon Territory, Canada.” *Master's Thesis. University of Ottawa*.
Rawat, Varsha, Suman Rawat, Priyeshu Srivastava, P. S. Negi, Muthusamy Prakasam, and Bahadur Singh Kotlia. 2021. “Multiproxy Paleoclimate Dataset from the Bednikund Alpine Lake in the Central Himalaya.” *Data in Brief* 35 (April): 106930. https://doi.org/10.1016/j.dib.2021.106930.
Regattieri, E., G. Zanchetta, R. N. Drysdale, I. Isola, J. C. Hellstrom, and L. Dallai. 2014. “Lateglacial to Holocene Trace Element Record (Ba, Mg, Sr) from Corchia Cave (Apuan Alps, Central Italy): Paleoenvironmental Implications.” *Journal of Quaternary Science* 29 (4): 381–92. https://doi.org/10.1002/jqs.2712.
REGATTIERI, E., G. ZANCHETTA, R. N. DRYSDALE, I. ISOLA, J. C. HELLSTROM, and L. DALLAI. 2014. “Lateglacial to Holocene Trace Element Record (Ba, Mg, Sr) from Corchia Cave (Apuan Alps, Central Italy): Paleoenvironmental Implications: Trace Element Record from Corchia Cave, Central Italy.” *Journal of Quaternary Science* 29 (4): 381–92. https://doi.org/10.1002/jqs.2712.
Regnell, J. 1989. “Vegetation and Land Use During 6000 Years.” *Palaeoecology of the Cultural Landscape at Two Lake Sites in Southern Skane*.
Reich, K. 1989. “Pollenanalytische Untersuchengen Des Moores Chavants Bei Les Houches (Mont Blanc-Massiv, Franzosische Alpen).” *Doctoral Dissertation. Universitat Zu Kiel*.
Reinemann, Scott A., David F. Porinchu, Amy M. Bloom, Bryan G. Mark, and Jason E. Box. 2009. “A Multi-Proxy Paleolimnological Reconstruction of Holocene Climate Conditions in the Great Basin, United States.” *Quaternary Research* 72 (3): 347–58. https://doi.org/10.1016/j.yqres.2009.06.003.
Renssen, Hans. 2022. “Climate Model Experiments on the 4.2 Ka Event: The Impact of Tropical Sea-Surface Temperature Anomalies and Desertification.” *The Holocene* 32 (5): 378–89. https://doi.org/10.1177/09596836221074031.
Repschläger, Janne, Dieter Garbe-Schönberg, Mara Weinelt, and Ralph Schneider. 2017. “Holocene Evolution of the North Atlantic Subsurface Transport.” *Climate of the Past* 13 (4): 333–44. https://doi.org/10.5194/cp-13-333-2017.
Revel, Marie, E. Ducassou, C. Skonieczny, C. Colin, L. Bastian, D. Bosch, S. Migeon, and J. Mascle. 2015. “20,000 Years of Nile River Dynamics and Environmental Changes in the Nile Catchment Area as Inferred from Nile Upper Continental Slope Sediments.” *Quaternary Science Reviews* 130 (December): 200–221. https://doi.org/10.1016/j.quascirev.2015.10.030.
Rey, Fabian, Erika Gobet, Christoph Schwörer, Othmar Wey, Albert Hafner, and Willy Tinner. 2019. “Causes and Mechanisms of Synchronous Succession Trajectories in Primeval Central European Mixed<i>fagus Sylvatica</i>forests.” Edited by Crystal McMichael. *Journal of Ecology* 107 (3): 1392–1408. https://doi.org/10.1111/1365-2745.13121.
Rey, Fabian, Christoph Schwörer, Erika Gobet, Daniele Colombaroli, Jacqueline FN van Leeuwen, Silke Schleiss, and Willy Tinner. 2013. “Climatic and Human Impacts on Mountain Vegetation at Lauenensee (Bernese Alps, Switzerland) During the Last 14,000 Years.” *The Holocene* 23 (10): 1415–27. https://doi.org/10.1177/0959683613489585.
Richard, Herv'e, and Ludwig Eschenlohr. 1998. “Essai de Correlation Entre Les Donnees Polliniques Et Les Donnees Archeologiques [Le Cas Des forêts de Lajoux Dans Les Franches-Montagnes (Lajoux, Ju, Suisse)].” *Revue dArcheometrie* 22 (1): 29–37. https://doi.org/10.3406/arsci.1998.959.

Rippke, Molly Beland, Matthew T. Distler, and John M. Farrell. 2010. “Holocene Vegetation Dynamics of an Upper St. Lawrence River Wetland: Paleoecological Evidence for a Recent Increase in Cattail (Typha).” *Wetlands* 30 (4): 805–16. https://doi.org/10.1007/s13157-010-0068-0.
Risebrobakken, Bjørg, Trond Dokken, and Eystein Jansen. 2005. “Extent and Variability of the Meridional Atlantic Circulation in the Eastern Nordic Seas During Marine Isotope Stage 5 and Its Influence on the Inception of the Last Glacial.” In *The Nordic Seas: An Integrated Perspective Oceanography, Climatology, Biogeochemistry, and Modeling*, 323–39. American Geophysical Union. https://doi.org/10.1029/158gm20.
Risebrobakken, Bjørg, Trond Dokken, Lars Henrik Smedsrud, Carin Andersson, Eystein Jansen, Matthias Moros, and Elena V. Ivanova. 2011. “Early Holocene Temperature Variability in the Nordic Seas: The Role of Oceanic Heat Advection Versus Changes in Orbital Forcing.” *Paleoceanography* 26 (4). https://doi.org/10.1029/2011pa002117.
Risebrobakken, Bjørg, Eystein Jansen, Carin Andersson, Eirik Mjelde, and Kjersti Hevrøy. 2003. “A High-Resolution Study of Holocene Paleoclimatic and Paleoceanographic Changes in the Nordic Seas.” *Paleoceanography* 18 (1): n/a–. https://doi.org/10.1029/2002pa000764.
Ritchie, J. C. 1976. “The Late-Quaternary Vegetational History of the Western Interior of Canada.” *Canadian Journal of Botany* 54 (15): 1793–1818. https://doi.org/10.1139/b76-194.
Rivers, William Petty. 1999. *Landscape-Level Control of Population Dynamics: Late-Quaternary Paleoecology of Beech in Upper Michigan*. The University of Tennessee.
Roberts, Neil, Samantha L. Allcock, Fabien Arnaud, Jonathan R. Dean, Warren J. Eastwood, Matthew D. Jones, Melanie J. Leng, et al. 2016. “A Tale of Two Lakes: A Multi-Proxy Comparison of Lateglacial and Holocene Environmental Change in Cappadocia, Turkey.” *Journal of Quaternary Science* 31 (4): 348–62. https://doi.org/10.1002/jqs.2852.
Roberts, N., M. D. Jones, A. Benkaddour, W. J. Eastwood, M. L. Filippi, M. R. Frogley, H. F. Lamb, et al. 2008. “Stable Isotope Records of Late Quaternary Climate and Hydrology from Mediterranean Lakes: The ISOMED Synthesis.” *Quaternary Science Reviews* 27 (25-26): 2426–41. https://doi.org/10.1016/j.quascirev.2008.09.005.
Robles, Mary, Odile Peyron, Elisabetta Brugiapaglia, Guillemette M’enot, Lucas Dugerdil, Vincent Ollivier, Salom’e Ansanay-Alex, et al. 2022. “Impact of Climate Changes on Vegetation and Human Societies During the Holocene in the South Caucasus (Vanevan, Armenia): A Multiproxy Approach Including Pollen, NPPs and brGDGTs.” *Quaternary Science Reviews* 277 (February): 107297. https://doi.org/10.1016/j.quascirev.2021.107297.
Rodrigues, Teresa, Joan O. Grimalt, F’atima Abrantes, Filipa Naughton, and Jos’e-Abel Flores. 2010. “The Last Glacial-Interglacial Transition (LGIT) in the Western Mid-Latitudes of the North Atlantic: Abrupt Sea Surface Temperature Change and Sea Level Implications.” *Quaternary Science Reviews* 29 (15-16): 1853–62. https://doi.org/10.1016/j.quascirev.2010.04.004.
Roesch, M. 2009. “Zur Vorgeschichtlichen Besiedlung Und Landnutzung Im Noerdlichen Schwarzwald Aufgrund Vegetationsgeschichtlicher Untersuchungen in Zwei Karseen. [Prehistoric Settlement and Land Use History of the Northern Black Forest as Indicated by Pollenanalytical Investigations in Two Cirque Lakes].” *Mitt. Ver. Forstl. Standortskunde u. Forstpflanzenzuechtung* 46: 15 24.
Roland, T. P., C. J. Caseldine, D. J. Charman, C. S. M. Turney, and M. J. Amesbury. 2014. “Was There a ‘4.2 Ka Event’ in Great Britain and Ireland? Evidence from the Peatland Record.” *Quaternary Science Reviews* 83 (January): 11–27. https://doi.org/10.1016/j.quascirev.2013.10.024.
Romahn, S., A. Mackensen, J. Groeneveld, and J. Pätzold. 2014. “Deglacial Intermediate Water Reorganization: New Evidence from the Indian Ocean.” *Climate of the Past* 10 (1): 293–303. https://doi.org/10.5194/cp-10-293-2014.
Roos-Barraclough, F., A. Martinez-Cortizas, Eduardo GarcıNANAa-Rodeja, and W. Shoty. 2002. “A 14 500 Year Record of the Accumulation of Atmospheric Mercury in Peat: Volcanic Signals, Anthropogenic Influences and a Correlation to Bromine Accumulation.” *Earth and Planetary Science Letters* 202 (2): 435–51. https://doi.org/10.1016/s0012-821x(02)00805-1.
Rösch, M. 1985. “Ein Pollenprofil Aus Dem Feuenried Bei Uberlingen Am Ried: Stratigraphische Und Landschaftsgeschichtliche Bedeutung Fur Das Holozan Im Bodenseegebiet in Berichte Zu Ufer-Und Moorsiedlungen Sudwestdeutschlands 2.” *Materialhefte Zur Vor-Und Fruhgeschichte in Baden-Wurttemberg*.
———. 1990. “Vegetationsgeschichtliche Untersuchungen Im Durchenbergried in Siedlungsarchaologie Im Alpenvorland II.” *Forschungen Und Berichte Zur Vor-Und Fruhgeschichte in Baden-Wurttemberg*.

———. 1992. “Human Impact as Registered in the Pollen Record: Some Results from the Western Lake Constance Region, Southern Germany.” *Vegetation History and Archaeobotany* 1 (2). https://doi.org/10.1007/bf00206090.
———. 2000. “Long-Term Human Impact as Registered in an Upland Pollen Profile from the Southern Black Forest, South-Western Germany.” *Vegetation History and Archaeobotany* 9 (4): 205–18. https://doi.org/10.1007/bf01294635.
———. 2020. “Zur Vegetationsgeschichte Des Südlichen Kraichgaus - Botanische Untersuchungen Bei Großvillars, Gemeinde Oberderdingen, Landkreis Karlsruhe.” *Fundberichte Aus Baden-Württemberg*, Bd. 28 Nr. 1 (2005): Fundberichte aus Baden–Württemberg. https://doi.org/10.11588/FBBW.2005.1.74511.
Rösch, Manfred. 2009. “Botanical Evidence for Prehistoric and Medieval Land Use in the Black Forest.” In *Ruralia*, 335–43. Brepols Publishers. https://doi.org/10.1484/m.ruralia-eb.3.1181.
Rösch, M., and E. Fischer. 2000. “A Radiocarbon Dated Holocene Pollen Profile from the Banat Mountains (Southwestern Carpathians, Romania).” *Flora* 195 (3): 277–86. https://doi.org/10.1016/s0367-2530(17)30981-7.
Rösch, M., and J. Lechterbeck. 2016. “Seven Millennia of Human Impact as Reflected in a High Resolution Pollen Profile from the Profundal Sediments of Litzelsee, Lake Constance Region, Germany.” *Vegetation History and Archaeobotany* 25 (4): 339–58. https://doi.org/10.1007/s00334-015-0552-9.
Rösch, M., and W. Ostendorf. 1988. “Pollenanalytische, Torf- Und Sedimentpetrographische Untersuchungen an Einen Telmatischen Profil Vom Bodensee-Ufer Bei Gaienhofen.” *Telma*.
Rösch, M., and G. Tserendorj. 2011. “The Northern Black Forest - Settled Earlier Than Thought? Pollen Profiles Show Extensive Prehistoric Settlement and Land Use.” *Preservation of Monuments in Baden-Wuerttemberg -Newsblatt Der Landesdenkmalpflege*.
Rosenthal, Yair, Delia W. Oppo, and Braddock K. Linsley. 2003. “The Amplitude and Phasing of Climate Change During the Last Deglaciation in the Sulu Sea, Western Equatorial Pacific.” *Geophysical Research Letters* 30 (8). https://doi.org/10.1029/2002gl016612.
Rossi, Carlos, Petra Bajo, Rafael P. Lozano, and John Hellstrom. 2018. “Younger Dryas to Early Holocene Paleoclimate in Cantabria (n Spain): Constraints from Speleothem Mg, Annual Fluorescence Banding and Stable Isotope Records.” *Quaternary Science Reviews* 192 (July): 71–85. https://doi.org/10.1016/j.quascirev.2018.05.025.
Routson, Cody C., Michael P. Erb, and Nicholas P. McKay. 2022. “High Latitude Modulation of the Holocene North American Monsoon.” *Geophysical Research Letters* 49 (16). https://doi.org/10.1029/2022gl099772.
Roy, Ipsita, Parminder Singh Ranhotra, Nidhi Tomar, Mayank Shekhar, Shailesh Agrawal, Amalava Bhattacharyya, Pankaj Kumar, Shiva Kumar Patil, and Rajveer Sharma. 2022. “Reconstruction of the Late Holocene Climate Variability from the Summer Monsoon Dominated Bhagirathi Valley, Western Himalaya.” *Journal of Asian Earth Sciences* 227 (April): 105080. https://doi.org/10.1016/j.jseaes.2022.105080.
Ruan, J., F. Kherbouche, D. Genty, D. Blamart, H. Cheng, F. Dewilde, S. Hachi, R. L. Edwards, E. R’egnier, and J.-L. Michelot. 2016. “Evidence of a Prolonged Drought Ca. 4200 Yr BP Correlated with Prehistoric Settlement Abandonment from the Gueldaman GLD1 Cave, Northern Algeria.” *Climate of the Past* 12 (1): 1–14. https://doi.org/10.5194/cp-12-1-2016.
Rudaya, Natalia, Pavel Tarasov, Nadezhda Dorofeyuk, Nadia Solovieva, Ivan Kalugin, Andrei Andreev, Andrei Daryin, Bernhard Diekmann, Frank Riedel, and Narantsetseg Tserendash. 2009. “Holocene Environments and Climate in the Mongolian Altai Reconstructed from the Hoton-Nur Pollen and Diatom Records: A Step Towards Better Understanding Climate Dynamics in Central Asia.” *Quaternary Science Reviews* 28 (5-6): 540–54. https://doi.org/10.1016/j.quascirev.2008.10.013.
Ruffaldi, P. 1993. “Histoire de La Vegetation Du Jura Meridional Depuis Le Retrait Du Glacier Wurmien a Partir Des Analyses Palynologiques Du Lac de Cerin (Ain, France).” *Doctoral Dissertation. Universite de Franche-Comte*.
Russell, James M., and Thomas C. Johnson. 2005. “A High-Resolution Geochemical Record from Lake Edward, Uganda Congo and the Timing and Causes of Tropical African Drought During the Late Holocene.” *Quaternary Science Reviews* 24 (12-13): 1375–89. https://doi.org/10.1016/j.quascirev.2004.10.003.
Rybn’ıček, Kamil, and Eliška Rybn’ıčkov’a. 1985. “A Palaeoecological Reconstruction of Precultural Vegetation in the Intermontane Basins of the Western Carpathians.” *Ecologia Mediterranea* 11 (1): 27–31. https://doi.org/10.3406/ecmed.1985.1067.
Rybn’ıčkov’a, Eliška, and Kamil Rybn’ıček. 2006. “Pollen and Macroscopic Analyses of Sediments from Two Lakes in the High Tatra Mountains, Slovakia.” *Vegetation History and Archaeobotany* 15 (4): 345–56. https://doi.org/10.1007/s00334-006-0050-1.

Rybnicek, K., and E. Rybnickova. 1977. “Moor Investigations in the Upper Gurgltal, Otztal Alps.” *Folia Geobotanica Et Phytotaxonomica*.

Saarse, L., and L. K. Konigsson. 1992. “Holocene Environmental Changes on the Island of Saaremaa, Estonia.” *PACT*.

Saarse, L, S Veski, A Heinsalu, R. Rajamae, and T. Martma. 1995. “Litho- and Biostratigraphy of Lake Paidre, South Estonia.” *Proceedings of the Estonian Academy of Sciences. Geology*.

Sabatier, Pierre, Laurent Dezileau, Christophe Colin, Louis Briqueu, Fr’ed’eric Bouchette, Philippe Martinez, Giuseppe Siani, Olivier Raynal, and Ulrich Von Grafenstein. 2012. “7000 Years of Paleostorm Activity in the NW Mediterranean Sea in Response to Holocene Climate Events.” *Quaternary Research* 77 (1): 1–11. https://doi.org/10.1016/j.yqres.2011.09.002.

Sachs, Julian P. 2007. “Cooling of Northwest Atlantic Slope Waters During the Holocene.” *Geophysical Research Letters* 34 (3). https://doi.org/10.1029/2006gl028495.

Sakamoto, Tatsuhiko, Minoru Ikehara, Masao Uchida, Kaori Aoki, Yasuyuki Shibata, Toshiya Kanamatsu, Naomi Harada, Koichi Iijima, Kota Katsuki, and Hiroshi Asahi. 2006. “Millennial-Scale Variations of Sea-Ice Expansion in the Southwestern Part of the Okhotsk Sea During the Past 120 Kyr: Age Model and Ice-Rafted Debris in IMAGES Core MD01-2412.” *Global and Planetary Change* 53 (1-2): 58–77. https://doi.org/10.1016/j.gloplacha.2006.01.012.

Salonen, J. Sakari, Heikki Seppä, Minna Väliranta, Vivienne J. Jones, Angela Self, Maija Heikkilä, Seija Kultti, and Handong Yang. 2011. “The Holocene Thermal Maximum and Late-Holocene Cooling in the Tundra of NE European Russia.” *Quaternary Research* 75 (3): 501–11. https://doi.org/10.1016/j.yqres.2011.01.007.

Salvatteci, Renato, Ralph R. Schneider, Thomas Blanz, and Elfi Mollier-Vogel. 2019. “Deglacial to Holocene Ocean Temperatures in the Humboldt Current System as Indicated by Alkenone Paleothermometry.” *Geophysical Research Letters* 46 (1): 281–92. https://doi.org/10.1029/2018gl080634.

Saraswat, Rajeev, David W. Lea, Rajiv Nigam, Andreas Mackensen, and Dinesh K. Naik. 2013. “Deglaciation in the Tropical Indian Ocean Driven by Interplay Between the Regional Monsoon and Global Teleconnections.” *Earth and Planetary Science Letters* 375 (August): 166–75. https://doi.org/10.1016/j.epsl.2013.05.022.

Saravanan, Ponnusamy, Anil K. Gupta, Hongbo Zheng, Mruganka K. Panigrahi, and Muthusamy Prakasam. 2019. “Late Holocene Long Arid Phase in the Indian Subcontinent as Seen in Shallow Sediments of the Eastern Arabian Sea.” *Journal of Asian Earth Sciences* 181 (September): 103915. https://doi.org/10.1016/j.jseaes.2019.103915.

Sarmaja-Korjonen, Kaarina, and Heikki Seppä. 2007. “Abrupt and Consistent Responses of Aquatic and Terrestrial Ecosystems to the 8200 Cal. Yr Cold Event: A Lacustrine Record from Lake Arapisto, Finland.” *The Holocene* 17 (4): 457–67. https://doi.org/10.1177/0959683607077020.

Sarmaja-Korjonen, K, Y. Vasari, and C. A. Haeggstrom. 1991a. “January.” *Taxus Baccata and Influence of Iron Age Man on the Vegetation in Aland*.

———. 1991b. “Taxus Baccata and Influence of Iron Age Man on the Vegetation in Aland, SW Finland.” *Annales Botanici Fennici*.

Sarnthein, Michael, Sven Balmer, Pieter M Grootes, and Manfred Mudelsee. 2015a. “Planktic and Benthic ¹⁴c Reservoir Ages for Three Ocean Basins, Calibrated by a Suite of ¹⁴c Plateaus in the Glacial-to-Deglacial Suigetsu Atmospheric ¹⁴c Record.” *Radiocarbon* 57 (1): 129–51. https://doi.org/10.2458/azu_rc.57.17916.

———. 2015b. “Planktic and Benthic14C Reservoir Ages for Three Ocean Basins, Calibrated by a Suite of14C Plateaus in the Glacial-to-Deglacial Suigetsu Atmospheric14C Record.” *Radiocarbon* 57 (1): 129–51. https://doi.org/10.2458/azu_rc.57.17916.

SARNTHEIN, M., S. VAN KREVELD, H. ERLENKEUSER, P. M. GROOTES, M. KUCERA, U. PFLAUMANN, and M. SCHULZ. 2003. “Centennial-to-millennial-scale Periodicities of Holocene Climate and Sediment Injections Off the Western Barents Shelf, 75°n.” *Boreas* 32 (3): 447–61. https://doi.org/10.1111/j.1502-3885.2003.tb01227.x.

Sauchyn, Mary A., and David J. Sauchyn. 1991. “A Continuous Record of Holocene Pollen from Harris Lake, Southwestern Saskatchewan, Canada.” *Palaeogeography, Palaeoclimatology, Palaeoecology* 88 (1-2): 13–23. https://doi.org/10.1016/0031-0182(91)90012-g.

Savoie, Louise, and Pierre Richard. 2010a. “Paleophytogeographie de l’episode de Saint-Narcisse Dans La Region de Sainte-Agathe, Quebec.” *Geographie Physique Et Quaternaire* 33 (2): 175–88. https://doi.org/10.7202/1000067ar.

———. 2010b. “Paléophytogéographie de l’épisode de Saint-Narcisse Dans La Région de Sainte-Agathe, Québec.” *Géographie Physique Et Quaternaire* 33 (2): 175–88. https://doi.org/10.7202/1000067ar.

Schauffler, M. 1998. “Paleoecology of Coastal and Interior Picea (Spruce) Stands in Maine.” *Doctoral Dissertation. University of Maine*.
Schefuß, Enno, Stefan Schouten, and Ralph R. Schneider. 2005. “Climatic Controls on Central African Hydrology During the Past 20,000years.” *Nature* 437 (7061): 1003–6. https://doi.org/10.1038/nature03945.
Schirrmacher, Julien, Nils Andersen, Ralph R. Schneider, and Mara Weinelt. 2020. “Fossil Leaf Wax Hydrogen Isotopes Reveal Variability of Atlantic and Mediterranean Climate Forcing on the Southeast Iberian Peninsula Between 6000 to 3000 Cal. BP.” Edited by Peter F. Biehl. *PLOS ONE* 15 (12): e0243662. https://doi.org/10.1371/journal.pone.0243662.
Schirrmacher, Julien, Mara Weinelt, Thomas Blanz, Nils Andersen, Em’ılia Salgueiro, and Ralph R. Schneider. 2019. “Multi-Decadal Atmospheric and Marine Climate Variability in Southern Iberia During the Mid- to Late-Holocene.” *Climate of the Past* 15 (2): 617–34. https://doi.org/10.5194/cp-15-617-2019.
Schmidt, Matthew W., William A. Weinlein, Franco Marcantonio, and Jean Lynch-Stieglitz. 2012. “Solar Forcing of Florida Straits Surface Salinity During the Early Holocene.” *Paleoceanography* 27 (3): n/a–. https://doi.org/10.1029/2012pa002284.
Schmieder, J., S. C. Fritz, J. B. Swinehart, A. L. C. Shinneman, A. P. Wolfe, G. Miller, N. Daniels, K. C. Jacobs, and E. C. Grimm. 2011. “A Regional-Scale Climate Reconstruction of the Last 4000 Years from Lakes in the Nebraska Sand Hills, USA.” *Quaternary Science Reviews* 30 (13-14): 1797–1812. https://doi.org/10.1016/j.quascirev.2011.04.011.
Schröder, Jan F., Ann Holbourn, Wolfgang Kuhnt, and Kevin Küssner. 2016. “Variations in Sea Surface Hydrology in the Southern Makassar Strait over the Past 26 Kyr.” *Quaternary Science Reviews* 154 (December): 143–56. https://doi.org/10.1016/j.quascirev.2016.10.018.
Schwörer, Christoph, Daniele Colombaroli, Petra Kaltenrieder, Fabian Rey, and Willy Tinner. 2015a. “Early Human Impact (5000-3000 BC) Affects Mountain Forest Dynamics in the Alps.” Edited by Amy Austin. *Journal of Ecology* 103 (2): 281–95. https://doi.org/10.1111/1365-2745.12354.
———. 2015b. “Early Human Impact (5000–3000 <Scp>BC</Scp>) Affects Mountain Forest Dynamics in the <Scp>a</Scp>lps.” Edited by Amy Austin. *Journal of Ecology* 103 (2): 281–95. https://doi.org/10.1111/1365-2745.12354.
Scroxton, Nick, Stephen J. Burns, David McGee, Laurie R. Godfrey, Lovasoa Ranivoharimanana, Peterson Faina, and Benjamin H. Tiger. 2023a. “Hydroclimate Variability in the Madagascar and Southeast African Summer Monsoons at the Mid- to Late-Holocene Transition.” *Quaternary Science Reviews* 300 (January): 107874. https://doi.org/10.1016/j.quascirev.2022.107874.
———. 2023b. “Tropical Indian Ocean Basin Hydroclimate at the Mid- to Late-Holocene Transition and the Double Drying Hypothesis.” *Quaternary Science Reviews* 300 (January): 107837. https://doi.org/10.1016/j.quascirev.2022.107837.
Sejrup, H. P., H. Haflidason, and J. T. Andrews. 2011. “A Holocene North Atlantic SST Record and Regional Climate Variability.” *Quaternary Science Reviews* 30 (21-22): 3181–95. https://doi.org/10.1016/j.quascirev.2011.07.025.
Self, A. E., A. Klimaschewski, N. Solovieva, V. J. Jones, E. Andr’en, A. A. Andreev, D. Hammarlund, and S. J. Brooks. 2015. “The Relative Influences of Climate and Volcanic Activity on Holocene Lake Development Inferred from a Mountain Lake in Central Kamchatka.” *Global and Planetary Change* 134 (November): 67–81. https://doi.org/10.1016/j.gloplacha.2015.06.012.
Seppa, H. 1996. “Post-Glacial Dynamics of Vegetation and Tree-Lines in the Far North of Fennoscandia.” *Fennia-International Journal of Geography*.
Seppä, H., A. E. Bjune, R. J. Telford, H. J. B. Birks, and S. Veski. 2009. “Last Nine-Thousand Years of Temperature Variability in Northern Europe.” *Climate of the Past* 5 (3): 523–35. https://doi.org/10.5194/cp-5-523-2009.
Seppä, Heikki, and H. J. B. Birks. 2001. “July Mean Temperature and Annual Precipitation Trends During the Holocene in the Fennoscandian Tree-Line Area: Pollen-Based Climate Reconstructions.” *The Holocene* 11 (5): 527–39. https://doi.org/10.1191/095968301680223486.
Seppä, Heikki, Marjut Nyman, Atte Korhola, and Jan Weckström. 2002. “Changes of Treelines and Alpine Vegetation in Relation to Post-Glacial Climate Dynamics in Northern Fennoscandia Based on Pollen and Chironomid Records.” *Journal of Quaternary Science* 17 (4): 287–301. https://doi.org/10.1002/jqs.678.
Seppä, Heikki, and Anneli Poska. 2004. “Holocene Annual Mean Temperature Changes in Estonia and Their Relationship to Solar Insolation and Atmospheric Circulation Patterns.” *Quaternary Research* 61 (1): 22–31. https://doi.org/10.1016/j.yqres.2003.08.005.

Seppä, H., D. Hammarlund, and K. Antonsson. 2005. “Low-Frequency and High-Frequency Changes in Temperature and Effective Humidity During the Holocene in South-Central Sweden: Implications for Atmospheric and Oceanic Forcings of Climate.” *Climate Dynamics* 25 (2–3): 285–97. https://doi.org/10.1007/s00382-005-0024-5.

Sha, Lijuan, Yassine Ait Brahim, Jasper A. Wassenburg, Jianjun Yin, Matthew Peros, Francisco W. Cruz, Yanjun Cai, et al. 2019. “How Far North Did the African Monsoon Fringe Expand During the African Humid Period? Insights from Southwest Moroccan Speleothems.” *Geophysical Research Letters* 46 (23): 14093–102. https://doi.org/10.1029/2019gl084879.

Shakun, Jeremy D., Stephen J. Burns, Peter U. Clark, Hai Cheng, and R. Lawrence Edwards. 2011a. “Milankovitch-Paced Termination II in a Nevada Speleothem?” *Geophysical Research Letters* 38 (18): n/a–. https://doi.org/10.1029/2011gl048560.

———. 2011b. “Milankovitch-Paced Termination II in a Nevada Speleothem?: NEVADA SPELEOTHEM TERMINATION II?” *Geophysical Research Letters* 38 (18): n/a–. https://doi.org/10.1029/2011gl048560.

Shane, L. C. 1976. “Late-Glacial and Postglacial Palynology and Chronology of Darke County, West-Central Ohio.” *Doctoral Dissertation. Kent State University*.

Shapley, M. D., E. Ito, and J. J. Donovan. 2009. “Lateglacial and Holocene Hydroclimate Inferred from a Groundwater Flow-Through Lake, Northern Rocky Mountains, USA.” *The Holocene* 19 (4): 523–35. https://doi.org/10.1177/0959683609104029.

Sharifi, Arash, Ali Pourmand, Elizabeth A. Canuel, Erin Ferer-Tyler, Larry C. Peterson, Bernhard Aichner, Sarah J. Feakins, et al. 2015. “Abrupt Climate Variability Since the Last Deglaciation Based on a High-Resolution, Multi-Proxy Peat Record from NW Iran: The Hand That Rocked the Cradle of Civilization?” *Quaternary Science Reviews* 123 (September): 215–30. https://doi.org/10.1016/j.quascirev.2015.07.006.

Shemesh, Aldo, Gunhild Rosqvist, Miri Rietti-Shati, Lena Rubensdotter, Christian Bigler, Ruth Yam, and Wibjörn Karl’en. 2001. “Holocene Climatic Change in Swedish Lapland Inferred from an Oxygen-Isotope Record of Lacustrine Biogenic Silica.” *The Holocene* 11 (4): 447–54. https://doi.org/10.1191/095968301678302887.

Shen, Caiming, Kam-Biu Liu, Carrie Morrill, Jonathan. T. Overpeck, Jinlan Peng, and Lingyu Tang. 2008. “Ecotone Shift and Major Droughts During the Mid-Late Holocene in the Central Tibetan Plateau.” *Ecology* 89 (4): 1079–88. https://doi.org/10.1890/06-2016.1.

Shen, Caiming, Kam-biu Liu, Lingyu Tang, and Jonathan T. Overpeck. 2006. “Quantitative Relationships Between Modern Pollen Rain and Climate in the Tibetan Plateau.” *Review of Palaeobotany and Palynology* 140 (1-2): 61–77. https://doi.org/10.1016/j.revpalbo.2006.03.001.

Shu, Peixian, Shugang Kang, Zhengguo Shi, David A. Grimley, Zeke Zhang, Jiaju Zhao, Hong Wang, Weijian Zhou, and Zhisheng An. 2023. “Southward Migration of the Monsoonal Rainbelt Hinders Paleosol Development and Preservation in North-Central China Dunefield After the Middle-Late Holocene Transition.” *Quaternary Science Reviews* 301 (February): 107919. https://doi.org/10.1016/j.quascirev.2022.107919.

Shuchun, Yao, Li Chunhai, Chen Yangsheng, Li Yongfei, Tang Lingyu, Shen Ji, and Xue Bin. 2022. “Moisture Conditions During the Younger Dryas and 4.2 Ka Event as Revealed from a Subalpine Peat Record in the Luoxiao Mountains, Southern China.” *Review of Palaeobotany and Palynology* 305 (October): 104747. https://doi.org/10.1016/j.revpalbo.2022.104747.

Shuman, B. N., G. E. Carter, D. D. Hougardy, K. Powers, and J. J. Shinker. 2014. “A North-South Moisture Dipole at Multi-Century Scales in the Central and Southern Rocky Mountains, u.s.a., During the Late Holocene.” *Rocky Mountain Geology* 49 (1): 33–49. https://doi.org/10.2113/gsrocky.49.1.33.

Shuman, Bryan N., and Jeremiah Marsicek. 2016. “The Structure of Holocene Climate Change in Mid-Latitude North America.” *Quaternary Science Reviews* 141 (June): 38–51. https://doi.org/10.1016/j.quascirev.2016.03.009.

Shuman, Bryan N., Paul Pribyl, and Jacob Buettner. 2015. “Hydrologic Changes in Colorado During the Mid-Holocene and Younger Dryas.” *Quaternary Research* 84 (2): 187–99. https://doi.org/10.1016/j.yqres.2015.07.004.

Shuman, Bryan N., and Marc Serravezza. 2017. “Patterns of Hydroclimatic Change in the Rocky Mountains and Surrounding Regions Since the Last Glacial Maximum.” *Quaternary Science Reviews* 173 (October): 58–77. https://doi.org/10.1016/j.quascirev.2017.08.012.

Shuman, Bryan, Anna K. Henderson, Steven M. Colman, Jeffery R. Stone, Sherilyn C. Fritz, Lora R. Stevens, Mitchell J. Power, and Cathy Whitlock. 2009. “Holocene Lake-Level Trends in the Rocky Mountains, u.s.a.” *Quaternary Science Reviews* 28 (19-20): 1861–79. https://doi.org/10.1016/j.quascirev.2009.03.003.

Singh, Shweta, Anil K. Gupta, Suman Rawat, Ajoy K. Bhaumik, Pankaj Kumar, and Santosh K. Rai. 2022. "Paleomonsoonal Shifts During ~13700 to 3100 Yr BP in the Central Ganga Basin, India with a Severe Arid Phase at ~4.2 Ka." *Quaternary International* 629 (August): 65–73. https://doi.org/10.1016/j.quaint.2021.01.015.

Sinha, Ashish, Gayatri Kathayat, Hai Cheng, Sebastian F. M. Breitenbach, Max Berkelhammer, Manfred Mudelsee, Jayant Biswas, and R. L. Edwards. 2015. "Trends and Oscillations in the Indian Summer Monsoon Rainfall over the Last Two Millennia." *Nature Communications* 6 (1). https://doi.org/10.1038/ncomms7309.

Sinninghe Damst'e, Jaap S., Jort Ossebaar, Stefan Schouten, and Dirk Verschuren. 2012. "Distribution of Tetraether Lipids in the 25-Ka Sedimentary Record of Lake Challa: Extracting Reliable TEX86 and MBT/CBT Palaeotemperatures from an Equatorial African Lake." *Quaternary Science Reviews* 50 (September): 43–54. https://doi.org/10.1016/j.quascirev.2012.07.001.

Sletten, Hillary R., L. Bruce Railsback, Fuyuan Liang, George A. Brook, Eugene Marais, Benjamin F. Hardt, Hai Cheng, and R. Lawrence Edwards. 2013a. "A Petrographic and Geochemical Record of Climate Change over the Last 4600years from a Northern Namibia Stalagmite, with Evidence of Abruptly Wetter Climate at the Beginning of Southern Africa's Iron Age." *Palaeogeography, Palaeoclimatology, Palaeoecology* 376 (April): 149–62. https://doi.org/10.1016/j.palaeo.2013.02.030.

———. 2013b. "A Petrographic and Geochemical Record of Climate Change over the Last 4600years from a Northern Namibia Stalagmite, with Evidence of Abruptly Wetter Climate at the Beginning of Southern Africas Iron Age." *Palaeogeography, Palaeoclimatology, Palaeoecology* 376 (April): 149–62. https://doi.org/10.1016/j.palaeo.2013.02.030.

Smith, Andrew C., Peter M. Wynn, Philip A. Barker, Melanie J. Leng, Stephen R. Noble, and Wlodek Tych. 2016. "North Atlantic Forcing of Moisture Delivery to Europe Throughout the Holocene." *Scientific Reports* 6 (1). https://doi.org/10.1038/srep24745.

Snyder, G. G, L. C. K. Shane, and R. O. Kapp. 1991. "Palynological Studies Associated with the Mound City Group National Monument, Chillicothe, Ohio." *Unknown*.

Solignac, Sandrine, Jacques Giraudeau, and Anne de Vernal. 2006. "Holocene Sea Surface Conditions in the Western North Atlantic: Spatial and Temporal Heterogeneities." *Paleoceanography* 21 (2). https://doi.org/10.1029/2005pa001175.

Solignac, Sandrine, Michael Grelaud, Anne de Vernal, Jacques Giraudeau, Matthias Moros, I. Nicholas McCave, and Babette Hoogakker. 2008. "Reorganization of the Upper Ocean Circulation in the Mid-Holocene in the Northeastern." Edited by Pete Hollings. *Canadian Journal of Earth Sciences* 45 (11): 1417–33. https://doi.org/10.1139/e08-061.

Solovieva, N., A. Klimaschewski, A. E. Self, V. J. Jones, E. Andr'en, A. A. Andreev, D. Hammarlund., E. V. Lepskaya, and L. Nazarova. 2015. "The Holocene Environmental History of a Small Coastal Lake on the North-Eastern Kamchatka Peninsula." *Global and Planetary Change* 134 (November): 55–66. https://doi.org/10.1016/j.gloplacha.2015.06.010.

Sone, Tomomi, Akihiro Kano, Tomoyo Okumura, Kenji Kashiwagi, Masako Hori, Xiuyang Jiang, and Chuan-Chou Shen. 2013. "Holocene Stalagmite Oxygen Isotopic Record from the Japan Sea Side of the Japanese Islands, as a New Proxy of the East Asian Winter Monsoon." *Quaternary Science Reviews* 75 (September): 150–60. https://doi.org/10.1016/j.quascirev.2013.06.019.

Speranza, A. 2000. "Improving the Time Control of the Subboreal/Subatlantic Transition in a Czech Peat Sequence by 14C Wiggle-Matching." *Quaternary Science Reviews* 19 (16): 1589–1604. https://doi.org/10.1016/s0277-3791(99)00108-0.

Spigel, K. M. 2006. "Erosion and Sedimentation History of Emrick Lake, South-Central Wisconsin, in Response to Holocene Environmental Change." *Doctoral Dissertation. University of Wisconsin*.

Springer, Gregory S., Harold D. Rowe, Ben Hardt, R. Lawrence Edwards, and Hai Cheng. 2008. "Solar Forcing of Holocene Droughts in a Stalagmite Record from West Virginia in East-Central North America." *Geophysical Research Letters* 35 (17). https://doi.org/10.1029/2008gl034971.

Stager, J. Curt, Brian F. Cumming, and L. David Meeker. 2003. "A 10,000-Year High-Resolution Diatom Record from Pilkington Bay, Lake Victoria, East Africa." *Quaternary Research* 59 (2): 172–81. https://doi.org/10.1016/s0033-5894(03)00008-5.

Stager, J.Curt, Paul A Mayewski, and L.David Meeker. 2002. "Cooling Cycles, Heinrich Event 1, and the Desiccation of Lake Victoria." *Palaeogeography, Palaeoclimatology, Palaeoecology* 183 (1–2): 169–78. https://doi.org/10.1016/s0031-0182(01)00468-0.

Stanley, JeanNANADaniel, Michael D. Krom, Robert A. Cliff, and Jamie C. Woodwar. 2003. "Short Contribution: Nile Flow Failure at the End of the Old Kingdom, Egypt: Strontium Isotopic and Petrologic Evidence." *Geoarchaeology* 18 (3): 395–402. https://doi.org/10.1002/gea.10065.

Staubwasser, M., F. Sirocko, P. M. Grootes, and M. Segl. 2003. "Climate Change at the 4.2 Ka BP Termination of the Indus Valley Civilization and Holocene South Asian Monsoon Variability." *Geophysical Research Letters* 30 (8). https://doi.org/10.1029/2002gl016822.

Stebich, Martina, Kira Rehfeld, Frank Schlütz, Pavel E. Tarasov, Jiaqi Liu, and Jens Mingram. 2015. "Holocene Vegetation and Climate Dynamics of NE China Based on the Pollen Record from Sihailongwan Maar Lake." *Quaternary Science Reviews* 124 (September): 275–89. https://doi.org/10.1016/j.quascirev.2015.07.021.

Stebich, M., and T. Litt. 1997. "Das Georgenfelder Hochmoor-Ein Archiv Fur Vegetations-, Siedlungs-Und Bergbaugeschichte." *Leipziger Geowissenschaften*.

Stefanini, B. S. 2008. "A Comparison of Climate and Vegetation Dynamics in Central Ireland and NW Spain Since the Mid-Holocene." *Doctoral Dissertation. University of Dublin*.

Steig, Eric J., David L. Morse, Edwin D. Waddington, Minze Stuiver, Pieter M. Grootes, Paul A. Mayewski, Mark S. Twickler, and Sallie I. Whitlow. 2000. "Wisconsinan and Holocene Climate History from an Ice Core at Taylor Dome, Western Ross Embayment, Antarctica." *Geografiska Annaler: Series A, Physical Geography* 82 (2-3): 213–35. https://doi.org/10.1111/j.0435-3676.2000.00122.x.

Steinke, Stephan, Cornelia Glatz, Mahyar Mohtadi, Jeroen Groeneveld, Qianyu Li, and Zhimin Jian. 2011. "Past Dynamics of the East Asian Monsoon: No Inverse Behaviour Between the Summer and Winter Monsoon During the Holocene." *Global and Planetary Change* 78 (3–4): 170–77. https://doi.org/10.1016/j.gloplacha.2011.06.006.

Steinke, Stephan, Mahyar Mohtadi, Matthias Prange, Vidya Varma, Daniela Pittauerova, and Helmut W. Fischer. 2014a. "Mid- to Late-Holocene Australian-Indonesian Summer Monsoon Variability." *Quaternary Science Reviews* 93 (June): 142–54. https://doi.org/10.1016/j.quascirev.2014.04.006.

———. 2014b. "Mid- to Late-Holocene Australian–Indonesian Summer Monsoon Variability." *Quaternary Science Reviews* 93 (June): 142–54. https://doi.org/10.1016/j.quascirev.2014.04.006.

Steinman, Byron A., David P. Pompeani, Mark B. Abbott, Joseph D. Ortiz, Nathan D. Stansell, Matthew S. Finkenbinder, Lorita N. Mihindukulasooriya, and Aubrey L. Hillman. 2016. "Oxygen Isotope Records of Holocene Climate Variability in the Pacific Northwest." *Quaternary Science Reviews* 142 (June): 40–60. https://doi.org/10.1016/j.quascirev.2016.04.012.

Stenni, Barbara, Valerie Masson-Delmotte, Sigfus Johnsen, Jean Jouzel, Antonio Longinelli, Eric Monnin, Regine RoNANAthlisberger, and Enrico Selm. 2001. "An Oceanic Cold Reversal During the Last Deglaciation." *Science* 293 (5537): 2074–77. https://doi.org/10.1126/science.1059702.

Stenni, B., J. Jouzel, V. Masson-Delmotte, R. Röthlisberger, E. Castellano, O. Cattani, S. Falourd, S. J. Johnsen, A. Longinelli, and J. P. Sachs. 2004. "A Late-Glacial High-Resolution Site and Source Temperature Record Derived from the EPICA Dome c Isotope Records (East Antarctica)." *Earth and Planetary Science Letters* 217 (1-2): 183–95. https://doi.org/10.1016/s0012-821x(03)00574-0.

Stenni, B., V. Masson-Delmotte, E. Selmo, H. Oerter, H. Meyer, R. Röthlisberger, J. Jouzel, et al. 2010. "The Deuterium Excess Records of EPICA Dome c and Dronning Maud Land Ice Cores (East Antarctica)." *Quaternary Science Reviews* 29 (1-2): 146–59. https://doi.org/10.1016/j.quascirev.2009.10.009.

Steponaitis, Elena, Alexandra Andrews, David McGee, Jay Quade, Yu-Te Hsieh, Wallace S. Broecker, Bryan N. Shuman, Stephen J. Burns, and Hai Cheng. 2015. "Mid-Holocene Drying of the u.s. Great Basin Recorded in Nevada Speleothems." *Quaternary Science Reviews* 127 (November): 174–85. https://doi.org/10.1016/j.quascirev.2015.04.011.

Stewart, RJ. 1996. "The Vegetation and Land Use History of Moynagh Lough, County Meath with Correlation to the Archaeological Evidence from the Site." *Master's Thesis. Trinity College Dublin*.

STINE, SCOTT. 1990. "Past Climate at Mono Lake." *Nature* 345 (6274): 391–91. https://doi.org/10.1038/345391b0.

Stone, Jeffery R., and Sherilyn C. Fritz. 2006. "Multidecadal Drought and Holocene Climate Instability in the Rocky Mountains." *Geology* 34 (5): 409. https://doi.org/10.1130/g22225.1.

Stoner, Joseph S., Anne Jennings, Gr'eta B. Kristj'ansd'ottir, Gita Dunhill, John T. Andrews, and Jorunn Hardard'ottir. 2007. "A Paleomagnetic Approach Toward Refining Holocene Radiocarbon-Based Chronologies: Paleoceanographic Records from the North Iceland (MD99-2269) and East Greenland (MD99-2322) Margins." *Paleoceanography* 22 (1): n/a–. https://doi.org/10.1029/2006pa001285.

Stott, Lowell D. 2007a. “Comment on "Anomalous Radiocarbon Ages for Foraminifera Shells" by w. Broecker Et Al.: A Correction to the Western Tropical Pacific MD9821-81 Record.” *Paleoceanography* 22 (1): n/a–. https://doi.org/10.1029/2006pa001379.
———. 2007b. “Comment on ‘Anomalous Radiocarbon Ages for Foraminifera Shells’ by w. Broecker Et Al.: A Correction to the Western Tropical Pacific MD9821-81 Record.” *Paleoceanography* 22 (1). https://doi.org/10.1029/2006pa001379.
Stott, Lowell, Axel Timmermann, and Robert Thunell. 2007. “Southern Hemisphere and Deep-Sea Warming Led Deglacial Atmospheric CO2 Rise and Tropical Warming.” *Science* 318 (5849): 435–38. https://doi.org/10.1126/science.1143791.
Str’ıkis, Nicol’as M., Cristiano M. Chiessi, Francisco W. Cruz, Mathias Vuille, Hai Cheng, Eline A. de Souza Barreto, Gesine Mollenhauer, et al. 2015. “Timing and Structure of Mega-SACZ Events During Heinrich Stadial 1.” *Geophysical Research Letters* 42 (13): 5477. https://doi.org/10.1002/2015gl064048.
Str’ıkis, Nicol’as M., Francisco W. Cruz, Eline A. S. Barreto, Filipa Naughton, Mathias Vuille, Hai Cheng, Antje H. L. Voelker, et al. 2018. “South American Monsoon Response to Iceberg Discharge in the North Atlantic.” *Proceedings of the National Academy of Sciences* 115 (15): 3788–93. https://doi.org/10.1073/pnas.1717784115.
Strikis, N. M., F. W. Cruz, H. Cheng, I. Karmann, R. L. Edwards, M. Vuille, X. Wang, M. S. de Paula, V. F. Novello, and A. S. Auler. 2011. “Abrupt Variations in South American Monsoon Rainfall During the Holocene Based on a Speleothem Record from Central-Eastern Brazil.” *Geology* 39 (11): 1075–78. https://doi.org/10.1130/g32098.1.
Strobel, Paul, Marcel Bliedtner, Andrew S. Carr, Peter Frenzel, Björn Klaes, Gary Salazar, Julian Struck, Sönke Szidat, Roland Zech, and Torsten Haberzettl. 2021a. “Holocene Sea Level and Environmental Change at the Southern Cape - an 8.5kyr Multi-Proxy Paleoclimate Record from Lake Voëlvlei, South Africa.” *Climate of the Past* 17 (4): 1567–86. https://doi.org/10.5194/cp-17-1567-2021.
———. 2021b. “Holocene Sea Level and Environmental Change at the Southern Cape – an 8.5 Kyr Multi-Proxy Paleoclimate Record from Lake Voëlvlei, South Africa.” *Climate of the Past* 17 (4): 1567–86. https://doi.org/10.5194/cp-17-1567-2021.
Strother, Stephanie L, Ulrich Salzmann, Stephen J Roberts, Dominic A Hodgson, John Woodward, Wim Van Nieuwenhuyze, Elie Verleyen, Wim Vyverman, and Steven G Moreton. 2014. “Changes in Holocene Climate and the Intensity of Southern Hemisphere Westerly Winds Based on a High-Resolution Palynological Record from Sub-Antarctic South Georgia.” *The Holocene* 25 (2): 263–79. https://doi.org/10.1177/0959683614557576.
Sun, A. Z, Z. D Feng, L. Y. Tang, and Y. Z. Ma. 2008. “Vegetation and Climate Changes in the Western Part of the Loess Plateau Since 13 Ka BP.” *Acta Geographica SInica*.
Sun, Aizhi, and Zhaodong Feng. 2013. “Holocene Climatic Reconstructions from the Fossil Pollen Record at Qigai Nuur in the Southern Mongolian Plateau.” *The Holocene* 23 (10): 1391–1402. https://doi.org/10.1177/0959683613489581.
Sun, Huiling, Aifeng Zhou, Xiaowei Zhang, and Fahu Chen. 2011. “Fir Trees Disappeared 500years Ago in the Liupan Mountains on the Southwestern Loess Plateau, China.” *Review of Palaeobotany and Palynology* 166 (1-2): 69–75. https://doi.org/10.1016/j.revpalbo.2011.05.003.
Sundqvist, H. S., D. S. Kaufman, N. P. McKay, N. L. Balascio, J. P. Briner, L. C. Cwynar, H. P. Sejrup, et al. 2014. “Arctic Holocene Proxy Climate Database - New Approaches to Assessing Geochronological Accuracy and Encoding Climate Variables.” *Climate of the Past* 10 (4): 1605–31. https://doi.org/10.5194/cp-10-1605-2014.
SUNDQVIST, HANNA S., KARIN HOLMGREN, ANDERS MOBERG, CHRISTOPH SPÖTL, and AUGUSTO MANGINI. 2009. “Stable Isotopes in a Stalagmite from NW Sweden Document Environmental Changes over the Past 4000 Years.” *Boreas* 39 (1): 77–86. https://doi.org/10.1111/j.1502-3885.2009.00099.x.
Sun, and Xiangjun. 1993. “Holocene Palynological Records in Lake Selincuo, Northern Xizang.” *Acta Botanica Sinica* 35.
Suri’c, Maša, Andrea Columbu, Robert Lončari’c, Petra Bajo, Neven Boči’c, Nina Lončar, Russell N Drysdale, and John C Hellstrom. 2021. “Holocene Hydroclimate Changes in Continental Croatia Recorded in Speleothem δ13C and δ18O from Nova Grgosova Cave.” *The Holocene* 31 (9): 1401–16. https://doi.org/10.1177/09596836211019120.
Surmely, Fr’ed’eric, Yannick Miras, Pascal Guenet, Violaine Nicolas, Aur’elie Savignat, Boris Vanni‘ere, Anne-V’eronique Walter-Simonnet, Gabriel Servera, and St’efan Tzortzis. 2009. “Occupation and Land-Use History of a Medium Mountain from the Mid-Holocene: A Multidisciplinary Study Performed in the South

Cantal (French Massif Central).” *Comptes Rendus Palevol* 8 (8): 737–48. https://doi.org/10.1016/j.crpv.2009.07.002.
Svensson, N. O. 1989. “Late Weichseian and Early Holocene Shore Displacement in the Central Baltic, Based on Stratigraphical and Morphological Records from Eastern Smaland and Gotland, Sweden.” *Doctoral Dissertation. Lund University*.
Svobodov’a, Helena, Maurice Reille, and Claude Goeury. 2001a. “Past Vegetation Dynamics of Vltavský Luh, Upper Vltava River Valley in the Šumava Mountains. Czech Republic.” *Vegetation History and Archaeobotany* 10 (4): 185–99. https://doi.org/10.1007/pl00006930.
———. 2001b. “Past Vegetation Dynamics of Vltavskyluh, Upper Vltava River Valley in the Sumava Mountains. Czech Republic.” *Vegetation History and Archaeobotany* 10 (4): 185–99. https://doi.org/10.1007/pl00006930.
Svobodov’a, Helena, Lenka Soukupov’a, and Maurice Reille. 2002. “Diversified Development of Mountain Mires, Bohemian Forest, Central Europe, in the Last 13,000 Years.” *Quaternary International* 91 (1): 123–35. https://doi.org/10.1016/s1040-6182(01)00106-9.
Svobodova, H. 2002. “Preliminary Results of the Vegetation History in the Giant Mountains (Upska Raselina Mire and Cernohorska Raselina Bog).” *Opera Corcontica*.
Swierczynski, Tina, Stefan Lauterbach, Peter Dulski, Jos’e Delgado, Bruno Merz, and Achim Brauer. 2013. “Mid- to Late Holocene Flood Frequency Changes in the Northeastern Alps as Recorded in Varved Sediments of Lake Mondsee (Upper Austria).” *Quaternary Science Reviews* 80 (November): 78–90. https://doi.org/10.1016/j.quascirev.2013.08.018.
Szeicz, J. M., and G. M. MacDonald. 1991. “Postglacial Vegetation History of Oak Savanna in Southern Ontario.” *Canadian Journal of Botany* 69 (7): 1507–19. https://doi.org/10.1139/b91-195.
T’oth, M’onika, Enikő K Magyari, Krisztina Buczk’o, Mih’aly Braun, Konstantinos Panagiotopoulos, and Oliver Heiri. 2015. “Chironomid-Inferred Holocene Temperature Changes in the South Carpathians (Romania).” *The Holocene* 25 (4): 569–82. https://doi.org/10.1177/0959683614565953.
Talma, A. S., and John C. Vogel. 1992. “Late Quaternary Paleotemperatures Derived from a Speleothem from Cango Caves, Cape Province, South Africa.” *Quaternary Research* 37 (2): 203–13. https://doi.org/10.1016/0033-5894(92)90082-t.
Tamalavage, Anne E., Peter J. van Hengstum, Patrick Louchouarn, Sergey Molodtsov, Karl Kaiser, Jeffrey P. Donnelly, Nancy A. Albury, and Patricia L. Fall. 2018. “Organic Matter Sources and Lateral Sedimentation in a Bahamian Karst Basin (Sinkhole) over the Late Holocene: Influence of Local Vegetation and Climate.” *Palaeogeography, Palaeoclimatology, Palaeoecology* 506 (October): 70–83. https://doi.org/10.1016/j.palaeo.2018.06.014.
Tan, Liangcheng, Yanjun Cai, Hai Cheng, Lawrence R. Edwards, Yongli Gao, Hai Xu, Haiwei Zhang, and Zhisheng An. 2018. “Centennial- to Decadal-Scale Monsoon Precipitation Variations in the Upper Hanjiang River Region, China over the Past 6650 Years.” *Earth and Planetary Science Letters* 482 (January): 580–90. https://doi.org/10.1016/j.epsl.2017.11.044.
Tan, Liangcheng, Guanghui Dong, Zhisheng An, R. Lawrence Edwards, Haiming Li, Dong Li, Robert Spengler, et al. 2021. “Megadrought and Cultural Exchange Along the Proto-Silk Road.” *Science Bulletin* 66 (6): 603–11. https://doi.org/10.1016/j.scib.2020.10.011.
Tan, Liangcheng, Yanzhen Li, Xiqian Wang, Yanjun Cai, Fangyuan Lin, Hai Cheng, Le Ma, Ashish Sinha, and R. Lawrence Edwards. 2020. “Holocene Monsoon Change and Abrupt Events on the Western Chinese Loess Plateau as Revealed by Accurately Dated Stalagmites.” *Geophysical Research Letters* 47 (21). https://doi.org/10.1029/2020gl090273.
Tang, L. Y, C. H Li, C. B. An, and W. G. Wang. 2007. “Vegetation History of the Western Loess Plateau of China During the Last 40ka Based on Pollen Record.” *Acta Palaeontologica Sinica*.
Tang, and Lin-Yu. 1999. “New High Resolution Pollen Records from Two Lakes in Xizang (Tibet).” *Journal of Integrative Plant Biology*.
Tantau, I. 2003. “Pollen Analytic Researches in the Eastern Romanian Carpathians.” *History of Vegetation and Human Impact. Doctoral Dissertation. Aix-Marseille III University and Babes-Bolyai of Cluj-Napoca University*.
Tao, ShiChen, ChenBang An, FaHu Chen, LingYu Tang, ZongLi Wang, YanBin Lü, ZhiFei Li, TongMing Zheng, and JiaJu Zhao. 2010. “Pollen-Inferred Vegetation and Environmental Changes Since 16.7 Ka BP at Balikun Lake, Xinjiang.” *Chinese Science Bulletin* 55 (22): 2449–57. https://doi.org/10.1007/s11434-010-3174-8.

Taylor, Karen J., Seamus McGinley, Aaron P. Potito, Karen Molloy, and David W. Beilman. 2018. “A Mid to Late Holocene Chironomid-Inferred Temperature Record from Northwest Ireland.” *Palaeogeography, Palaeoclimatology, Palaeoecology* 505 (September): 274–86. https://doi.org/10.1016/j.palaeo.2018.06.006.
Teed, Rebecca, Charles Umbanhower, and Philip Camill. 2009. “Multiproxy Lake Sediment Records at the Northern and Southern Boundaries of the Aspen Parkland Region of Manitoba, Canada.” *The Holocene* 19 (6): 937–48. https://doi.org/10.1177/0959683609336569.
Them, T. R., M. W. Schmidt, and J. Lynch-Stieglitz. 2015. “Millennial-Scale Tropical Atmospheric and Atlantic Ocean Circulation Change from the Last Glacial Maximum and Marine Isotope Stage 3.” *Earth and Planetary Science Letters* 427 (October): 47–56. https://doi.org/10.1016/j.epsl.2015.06.062.
Thöle, Lena, Christoph Schwörer, Daniele Colombaroli, Erika Gobet, Petra Kaltenrieder, Jacqueline van Leeuwen, and Willy Tinner. 2015. “Reconstruction of Holocene Vegetation Dynamics at Lac de Bretaye, a High-Mountain Lake in the Swiss Alps.” *The Holocene* 26 (3): 380–96. https://doi.org/10.1177/0959683615609746.
Thomas, E. K., I. S. Castañeda, N. P. McKay, J. P. Briner, J. M. Salacup, K. Q. Nguyen, and A. D. Schweinsberg. 2018. “A Wetter Arctic Coincident with Hemispheric Warming 8,000 Years Ago.” *Geophysical Research Letters* 45 (19). https://doi.org/10.1029/2018gl079517.
Thomas, Elizabeth K., Jason Szymanski, and Jason P. Briner. 2010. “Holocene Alpine Glaciation Inferred from Lacustrine Sediments on Northeastern Baffin Island, Arctic Canada.” *Journal of Quaternary Science* 25 (2): 146–61. https://doi.org/10.1002/jqs.1286.
Thompson, Lonnie G., Ellen Mosley-Thompson, Mary E. Davis, Keith A. Henderson, Henry H. Brecher, Victor S. Zagorodnov, Tracy A. Mashiotta, et al. 2002. “Kilimanjaro Ice Core Records: Evidence of Holocene Climate Change in Tropical Africa.” *Science* 298 (5593): 589–93. https://doi.org/10.1126/science.1073198.
Thornalley, David J. R., Harry Elderfield, and I. Nick McCave. 2009. “Holocene Oscillations in Temperature and Salinity of the Surface Subpolar North Atlantic.” *Nature* 457 (7230): 711–14. https://doi.org/10.1038/nature07717.
TIAN, Fei, Yong WANG, Zhili ZHAO, Yang LI, Jin DONG, Jin LIU, Yuan LING, Lupeng YUAN, and Mengni YE. 2020. “Holocene Vegetation and Climate Changes in the Huangqihai Lake Region, Inner Mongolia.” *Acta Geologica Sinica - English Edition* 94 (4): 1178–86. https://doi.org/10.1111/1755-6724.14565.
Tich’a, Anna, Daniel Vondr’ak, Alice Moravcov’a, Richard Chiverrell, and Petr Kuneš. 2023. “Climate-Related Soil Saturation and Peatland Development May Have Conditioned Surface Water Brownification at a Central European Lake for Millennia.” *Science of The Total Environment* 858 (February): 159982. https://doi.org/10.1016/j.scitotenv.2022.159982.
Tinner, Willy, Marco Conedera, Brigitta Ammann, Heinz W. Gaggeler, Sharon Gedye, Richard Jones, and Beat Sagesser. 1998. “Pollen and Charcoal in Lake Sediments Compared with Historically Documented Forest Fires in Southern Switzerland Since AD 1920.” *The Holocene* 8 (1): 31–42. https://doi.org/10.1191/095968398667205430.
Tinner, Willy, Jacqueline F. N. van Leeuwen, Daniele Colombaroli, Elisa Vescovi, W. O. van der Knaap, Paul D. Henne, Salvatore Pasta, and Stefania Dand Tommaso La Mantia. 2009. “Holocene Environmental and Climatic Changes at Gorgo Basso, a Coastal Lake in Southern Sicily, Italy.” *Quaternary Science Reviews* 28 (15-16): 1498–1510. https://doi.org/10.1016/j.quascirev.2009.02.001.
Tinner, Willy, Elisa Vescovi, Jacqueline F. N. van Leeuwen, Daniele Colombaroli, Paul D. Henne, Petra Kaltenrieder, C’esar Morales-Molino, et al. 2016. “Holocene Vegetation and Fire History of the Mountains of Northern Sicily (Italy).” *Vegetation History and Archaeobotany* 25 (5): 499–519. https://doi.org/10.1007/s00334-016-0569-8.
Tong, G. B, Y Shi, R. J Wu, X. D. Yang, and W. C. Qu. 1997. “Vegetation and Climatic Quantitative Reconstruction of Longgan Lake Since the Past 3000 Years.” *Marine Geology and Quaternary Geology*.
Toth, Lauren T., and Richard B. Aronson. 2019. “The 4.2 Ka Event, ENSO, and Coral Reef Development.” *Climate of the Past* 15 (1): 105–19. https://doi.org/10.5194/cp-15-105-2019.
Uemura, R., N. Yoshida, N. Kurita, M. Nakawo, and O. Watanabe. 2004. “An Observation-Based Method for Reconstructing Ocean Surface Changes Using a 340,000-Year Deuterium Excess Record from the Dome Fuji Ice Core, Antarctica.” *Geophysical Research Letters* 31 (13): n/a–. https://doi.org/10.1029/2004gl019954.
Uemura, Ryu, Hideaki Motoyama, Val’erie Masson-Delmotte, Jean Jouzel, Kenji Kawamura, Kumiko Goto-Azuma, Shuji Fujita, et al. 2018. “Asynchrony Between Antarctic Temperature and CO2 Associated with Obliquity over the Past 720,000 Years.” *Nature Communications* 9 (1). https://doi.org/10.1038/s41467-018-03328-3.

Umbanhowar, Charles E. 2004. "Interaction of Fire, Climate and Vegetation Change at a Large Landscape Scale in the Big Woods of Minnesota, USA." *The Holocene* 14 (5): 661–76. https://doi.org/10.1191/0959683604hl745rp.

Väliranta, M., J. S. Salonen, M. Heikkilä, L. Amon, K. Helmens, A. Klimaschewski, P. Kuhry, et al. 2015. "Plant Macrofossil Evidence for an Early Onset of the Holocene Summer Thermal Maximum in Northernmost Europe." *Nature Communications* 6 (1). https://doi.org/10.1038/ncomms7809.

van Breukelen, M. R., H. B. Vonhof, J. C. Hellstrom, W. C. G. Wester, and D. Kroon. 2008. "Fossil Dripwater in Stalagmites Reveals Holocene Temperature and Rainfall Variation in Amazonia." *Earth and Planetary Science Letters* 275 (1-2): 54–60. https://doi.org/10.1016/j.epsl.2008.07.060.

Van Campo, E., and F. Gasse. 1993. "Pollen-and Diatom-Inferred Climatic and Hydrological Changes in Sumxi Co Basin (Western Tibet) Since 13,000 Yr BP." *Quaternary Research*.

van der Bilt, Willem G. M., William J. Dand Jostein Bakke, Nicholas L. Balascio, Johannes P. Werner, Marthe Gjerde, and Raymond S. Bradley. 2018. "Alkenone-Based Reconstructions Reveal Four-Phase Holocene Temperature Evolution for High Arctic Svalbard." *Quaternary Science Reviews* 183 (March): 204–13. https://doi.org/10.1016/j.quascirev.2016.10.006.

van der Knaap, W. O., and B. Ammann. 1997. "Depth-Age Relationships of 25 Well-Dated Swiss Holocene Pollen Sequences Archived in the Alpine Palynological Data-Base." *Revue de Paleobiologie*.

Van der Knaap, W. O., and J. van Leeuwen. 1994. "Holocene Vegetation, Human Impact, and Climatic Change in the Serra Da Estrela, Portugal." *Dissertationes Botanicae*.

van der Knaap, W. O., and J. F. N. Van Leeuwen. 2001. "Vegetationsgeschichte Und Menschlicher Einfluss in Der Umgebung Des Bibersees Zwischen 2600 Und 50 v." *Chr. Cham-Oberwil*.

van der Knaap, W. O, Jacqueline F. N van Leeuwen, Andreas Fankhauser, and Brigitta Ammann. 2000. "Palynostratigraphy of the Last Centuries in Switzerland Based on 23 Lake and Mire Deposits: Chronostratigraphic Pollen Markers, Regional Patterns, and Local Histories." *Review of Palaeobotany and Palynology* 108 (1-2): 85–142. https://doi.org/10.1016/s0034-6667(99)00035-4.

Van Rampelbergh, Maït'e, Dominik Fleitmann, Sophie Verheyden, Hai Cheng, Lawrence Edwards, Peter De Geest, David De Vleeschouwer, et al. 2013. "Mid- to Late Holocene Indian Ocean Monsoon Variability Recorded in Four Speleothems from Socotra Island, Yemen." *Quaternary Science Reviews* 65 (April): 129–42. https://doi.org/10.1016/j.quascirev.2013.01.016.

Van Zant, Kent. 1979. "Late Glacial and Postglacial Pollen and Plant Macrofossils from Lake West Okoboji, Northwestern Iowa." *Quaternary Research* 12 (3): 358–80. https://doi.org/10.1016/0033-5894(79)90034-6.

van Zeist, W., and M. R. van der Spoel-Walvius. 1980. "A Palynological Study of the Late-Glacial and the Postglacial in the Paris Basin." *Palaeohistoria*.

Vanniere, B., and F. Laggoun-Defarge. 2002. "Premiere Contribution a l'etude Des Evolutions Paleohydrologiques Et a l'histoire Des Feux En Champagne Berrichonne Durant l'holocene." *In: Les Fleuves Ont Une Histoire : Paleo- Environnement Des Rivieres Et Des Lacs Francais Depuis 15 000 Ans.*

Vasskog, Kristian, Øyvind Paasche, Atle Nesje, John F. Boyle, and H. J. B. Birks. 2012. "A New Approach for Reconstructing Glacier Variability Based on Lake Sediments Recording Input from More Than One Glacier." *Quaternary Research* 77 (1): 192–204. https://doi.org/10.1016/j.yqres.2011.10.001.

Venancio, I. M., S. Mulitza, A. Govin, T. P. Santos, D. O. Lessa, A. L. S. Albuquerque, C. M. Chiessi, et al. 2018. "Millennial- to Orbital-Scale Responses of Western Equatorial Atlantic Thermocline Depth to Changes in the Trade Wind System Since the Last Interglacial." *Paleoceanography and Paleoclimatology* 33 (12): 1490–1507. https://doi.org/10.1029/2018pa003437.

Verheyden, Sophie, Eddy Keppens, Ian J. Fairchild, Frank McDermott, and Dominique Weis. 2000. "Mg, Sr and Sr Isotope Geochemistry of a Belgian Holocene Speleothem: Implications for Paleoclimate Reconstructions." *Chemical Geology* 169 (1-2): 131–44. https://doi.org/10.1016/s0009-2541(00)00299-0.

Vescovi, Elisa, Brigitta Ammann, Cesare Ravazzi, and Willy Tinner. 2010. "A New Late-Glacial and Holocene Record of Vegetation and Fire History from Lago Del Greppo, Northern Apennines, Italy." *Vegetation History and Archaeobotany* 19 (3): 219–33. https://doi.org/10.1007/s00334-010-0243-5.

Vescovi, E, C Ravazzi, E Arpenti, W Finsinger, R Pini, V Valsecchi, L Wick, B. Ammann, and W. Tinner. 2007. "Interactions Between Climate and Vegetation During the Lateglacial Period as Recorded by Lake and Mire Sediment Archives in Northern Italy and Southern Switzerland." *Quaternary Science Reviews*.

Veski, S. 1998. "Vegetation History, Human Impact and Palaeogeography of West Estonia: Pollen Analytical Studies of Lake and Bog Sediments." *Doctoral Dissertation*.

Vimeux, Françoise, Kurt M. Cuffey, and Jean Jouzel. 2002. “New Insights into Southern Hemisphere Temperature Changes from Vostok Ice Cores Using Deuterium Excess Correction.” *Earth and Planetary Science Letters* 203 (3-4): 829–43. https://doi.org/10.1016/s0012-821x(02)00950-0.
Vinther, B. M., K. K. Andersen, P. D. Jones, K. R. Briffa, and J. Cappelen. 2006. “Extending Greenland Temperature Records into the Late Eighteenth Century.” *Journal of Geophysical Research* 111 (D11). https://doi.org/10.1029/2005jd006810.
Vinther, B. M., S. L. Buchardt, H. B. Clausen, D. Dahl-Jensen, S. J. Johnsen, D. A. Fisher, R. M. Koerner, et al. 2009. “Holocene Thinning of the Greenland Ice Sheet.” *Nature* 461 (7262): 385–88. https://doi.org/10.1038/nature08355.
Visset, L, A. L. Cyprien, and N. Carcaud. 2010. “Paleoenvironnements Tardiglaciaires Et Holocenes de l’orleanais (Loiret).” *Les Sites de Saint-Benoit-Sur-Loire Et de La Vallee Des Mauves a Meung-Sur-Loire. Revue Archeologique Du Loiret*.
Visset, L, C. Delalande, and C. Joly. 2006. “Tourbiere Et Marecage a Hoedic.” *Histoire Du Paysage Depuis 6000 Ans. Melvan*.
Visset, L, G Hauray, L. Charrieau, and N. Rouzeau. 2001. “Paleoenvironnement Urbain : Histoire Du Comblement Des Vallees de La Metropole Nantaise, Du Tardiglaciaire a La Fin de l’holocene.” *Annales de Bretagne Et Des Pays de l’Ouest*.
Visset, Lionel. 1985. “Dernieres Donnees Pollenanalytiques Et Radiometriques Du Golfe Brieron (Massif Armoricain, France).” *Ecologia Mediterranea* 11 (1): 107–16. https://doi.org/10.3406/ecmed.1985.1080.
———. 1987. “Etude Pollenanalytique de Quelques Sites Du Marais Poitevin.” *Bulletin de lAssociation Française Pour letude Du Quaternaire* 24 (2): 81–91. https://doi.org/10.3406/quate.1987.1836.
VISSET, LIONEL. 1988. “The Briere Marshlands: A Palynological Survey.” *New Phytologist* 110 (3): 409–24. https://doi.org/10.1111/j.1469-8137.1988.tb00279.x.
Visset, Lionel, Delphine Barbier, and Abdelouahed Ouguerram. 2005. “Le Paysage Vegetal Dans Le Bas-Maine (Mayenne, France), Le Long de La Vallee de lerve, de La Fin Du Mesolithique Alepoque Recente / Botanic Landscape in Bas-Maine (Mayenne, France), Along the Erve Valley, from the End of Mesolithic to Recent Times.” *Revue Archeologique de louest* 22 (1): 85–92. https://doi.org/10.3406/rao.2005.1117.
Visset, Lionel, and Jacques Bernard. 2006. “Évolution Du Littoral Et Du Paysage, de La Presqu’ile de Rhuys à La Rivière d’étel (Massif Armoricain – France), Du Néolithique Au Moyen Âge.” *Archeosciences*, no. 30 (December): 143–56. https://doi.org/10.4000/archeosciences.315.
Visset, Lionel, and Jean Land Jacques Bernard. 1996. “La Tourbiere Submergee de La Pointe de Kerpenhir aLocmariaquer (Morbihan). Etude Environnementale Et Mise En Evidence de Deforestations Et de Pratiques Agricoles Neolithiques.” *Revue Archeologique de louest* 13 (1): 79–87. https://doi.org/10.3406/rao.1996.1041.
Visset, Lionel, and A. Gauthier. 1998. “Naissance Et Evolution d’une Tourbiere : La Tourbiere de La Fontaine de La Lutiniere aAuzay Dans Le Marais Poitevin ; Implications Sur Les Relations Homme-Milieu.” *Le Journal de Botanique* 6 (1): 83–88. https://doi.org/10.3406/jobot.1998.1738.
Visset, Lionel, Dominique Sellier, and Jean L. 1995. “Le Paleoenvironnement de La Region de Carnac. Sondage Dans Le Marais de Kerdual, La Trinite-Sur-Mer (Morbihan).” *Revue Archeologique de louest* 12 (1): 57–71. https://doi.org/10.3406/rao.1995.1025.
Voarintsoa, Ny Riavo G, George A Brook, Fuyuan Liang, Eugene Marais, Ben Hardt, Hai Cheng, R Lawrence Edwards, and L Bruce Railsback. 2016. “Stalagmite Multi-Proxy Evidence of Wet and Dry Intervals in Northeastern Namibia: Linkage to Latitudinal Shifts of the Inter-Tropical Convergence Zone and Changing Solar Activity from AD 1400 to 1950.” *The Holocene* 27 (3): 384–96. https://doi.org/10.1177/0959683616660170.
Voeltzel, D. 1987. “Recherches Pollenanalytiques Sur La Vegetation Holocene de La Plaine Alluviale de l’estuaire de La Loire Et Des Coteaux Environnants.” *Doctoral Dissertation. Universite d’Aix-Marseille*.
Voigt, R. 1996. “Palaolimnologische Und Vegetationsgeschichtliche-Untersuchungen an Sedimenten Aus Fuschlsee Und Chiemsee-(salzburg Und Bayern).” *Dissertationes Botanicae*.
von Gunten, Lucien, William J. Dand Raymond S. Bradley, and Yongsong Huan. 2012. “Proxy-to-Proxy Calibration: Increasing the Temporal Resolution of Quantitative Climate Reconstructions.” *Scientific Reports* 2 (1). https://doi.org/10.1038/srep00609.
von Rad, Ulrich, Michael Schaaf, Klaus H. Michels, Hartmut Schulz, Wolfgang H. Berger, and Frank Sirocko. 1999. “A 5000-Yr Record of Climate Change in Varved Sediments from the Oxygen Minimum Zone Off Pakistan, Northeastern Arabian Sea.” *Quaternary Research* 51 (1): 39–53. https://doi.org/10.1006/qres.1998.2016.

Vorren, K. D., M. Blaauw, S. Wastegaard, J. Van Der Plicht, and C. Jenson. 2007. "High-Resolution Stratigraphy of the Northernmost Concentric Raised Bog in Europe: Sellevollmyra, andøya, Northern Norway." *Boreas* 36 (3): 253–77. https://doi.org/10.1111/j.1502-3885.2007.tb01249.x.

Vorren, K. D., C. E. Jensen, and E. Nilssen. 2011. "Climate Changes During the Last c. 7500 Years as Recorded by the Degree of Peat Humification in the Lofoten Region, Norway." *Boreas* 41 (1): 13–30. https://doi.org/10.1111/j.1502-3885.2011.00220.x.

VORREN, KARLNANADAG, MAARTEN BLAAUW, STEFAN WASTEGÅRD, JOHANNES VAN DER PLICHT, and CHRISTIN JENSE. 2007. "High-resolution Stratigraphy of the Northernmost Concentric Raised Bog in Europe: Sellevollmyra, Andøya, Northern Norway." *Boreas* 36 (3): 253–77. https://doi.org/10.1111/j.1502-3885.2007.tb01249.x.

Vuorela, I. 1981. "The Vegetational and Settlement History in Sysmä, Central South Finland, Interpreted on the Basis of Two Pollen Diagrams." *Bulletin of the Geological Society of Finland* 53 (1): 47–61. https://doi.org/10.17741/bgsf/53.1.005.

———. 1991. "Lounais-Suomen Varhaismetallikautinen Asutus Ja Viljely Siitepolyanalyysin Valossa." *Karhunhammas*.

Wahl, David, Roger Byrne, and Lysanna Anderson. 2014. "An 8700 Year Paleoclimate Reconstruction from the Southern Maya Lowlands." *Quaternary Science Reviews* 103 (November): 19–25. https://doi.org/10.1016/j.quascirev.2014.08.004.

Wahl, David, Roger Byrne, Thomas Schreiner, and Richard Hansen. 2006. "Holocene Vegetation Change in the Northern Peten and Its Implications for Maya Prehistory." *Quaternary Research* 65 (3): 380–89. https://doi.org/10.1016/j.yqres.2005.10.004.

Wahlmuller, N. 1985. "Beitrage Zur Vegetationsgeschichte Tirols v: Nordtiroler Kalkalpen." *Berichte Des Naturwissenschaftlich-Medizinischen Vereins in Innsbruck*.

Waller, M. P. 1987. "The Flandrian Vegetational History and Environmental Development of the Brede and Panel Valleys East Sussex." *Doctoral Dissertation. Polytechnic of North London*.

———. 1993. "Flandrian Vegetational History of Southeastern England. Pollen Data from Pannel Bridge, East Sussex." *New Phytologist* 124 (2): 345–69. https://doi.org/10.1111/j.1469-8137.1993.tb03825.x.

———. 1994. "The Fenland Project, Number 9: Flandrian Environmental Change in Fenland (No." *70). Cambridgeshire Archaeological Committee*.

Waller, M. P., and S. Hamilton. 2000. "Vegetation History of the English Chalklands: A Mid-Holocene Pollen Sequence from the Caburn, East Sussex." *Journal of Quaternary Science* 15 (3): 253–72. https://doi.org/10.1002/(sici)1099-1417(200003)15:3<253::aid-jqs481>3.0.co;2-8.

Waller, M. P., A. J. Long, D. Long, and J. B. Innes. 1999. "Patterns and Processes in the Development of Coastal Mire Vegetation: Multi-Site Investigations from Walland Marsh, Southeast England." *Quaternary Science Reviews* 18 (12): 1419–44. https://doi.org/10.1016/s0277-3791(98)00072-9.

Waller, M. P., and A. D. Marlow. 1994. "Flandrian Vegetational History of Sout-Heastern England. Stratigraphy of the Brede Valley and Pollen Data from Brede Bridge." *New Phytologist* 126 (2): 369–92. https://doi.org/10.1111/j.1469-8137.1994.tb03956.x.

Wang, Canfa, James A. Bendle, Hongbin Zhang, Yi Yang, Deng Liu, Junhua Huang, Jingwei Cui, and Shucheng Xie. 2018. "Holocene Temperature and Hydrological Changes Reconstructed by Bacterial 3-Hydroxy Fatty Acids in a Stalagmite from Central China." *Quaternary Science Reviews* 192 (July): 97–105. https://doi.org/10.1016/j.quascirev.2018.05.030.

Wang, Dandan, Manyue Li, Shengrui Zhang, Qinghai Xu, and Liwei Wu. 2022. "Spatial and Temporal Characteristics of the Precipitation Response to the 4.2 Ka Event in the Asian Summer Monsoon Region." *Global and Planetary Change* 214 (July): 103854. https://doi.org/10.1016/j.gloplacha.2022.103854.

Wang, L. G. 2003. "Study on Climatic and Environmental Changes During the Past 4.0 Ka in the Southern Margin of Tarim Basin, Southern Xinjiang." *Master Thesis. Xinjiang University*.

Wang, J., L. Sun, L., Chen, L., Xu, Y. Wang, and X. Wang. 2016. "The abrupt climate change near 4,400 yr BP on the cultural transition in Yuchisi, China and its global linkage". *Scientific Reports*, *6*(1), 27723.

Wang, Luejiang, Michael Sarnthein, Pieter M. Grootes, and Helmut Erlenkeuser. 1999. "Millennial Reoccurrence of Century-Scale Abrupt Events of East Asian Monsoon: A Possible Heat Conveyor for the Global Deglaciation." *Paleoceanography* 14 (6): 725–31. https://doi.org/10.1029/1999pa900028.

WANG, Mingda, Yaping YANG, Jiawu ZHANG, and Juzhi HOU. 2020. "Paleohydrological Changes in the Western Tibetan Plateau over the Past 16,000 Years Based on Sedimentary Records Ofn-alkanes and Grain Size." *Acta Geologica Sinica - English Edition* 94 (3): 707–16. https://doi.org/10.1111/1755-6724.14538.

Wang, M., Y. Yang, J. Zhang, and J. Hou. 2020. "Paleohydrological Changes in the Western Tibetan Plateau over the Past 16,000 Years Based on Sedimentary Records of n-Alkanes and Grain Size." *Acta Geologica Sinica - English Edition* 94 (3): 707–16. https://doi.org/10.1111/1755-6724.14538.

Wang, Tianli, Dong Li, Xing Cheng, Jianghu Lan, R. Lawrence Edwards, Hai Cheng, Xingxing Liu, et al. 2022. "Hydroclimatic Changes in South-Central China During the 4.2 Ka Event and Their Potential Impacts on the Development of Neolithic Culture." *Quaternary Research* 109 (May): 39–52. https://doi.org/10.1017/qua.2022.11.

Wang, Wei, Zhaodong Feng, Min Ran, and Chengjun Zhang. 2013. "Holocene Climate and Vegetation Changes Inferred from Pollen Records of Lake Aibi, Northern Xinjiang, China: A Potential Contribution to Understanding of Holocene Climate Pattern in East-Central Asia." *Quaternary International* 311 (October): 54–62. https://doi.org/10.1016/j.quaint.2013.07.034.

Wang, Xianfeng, R. Lawrence Edwards, Augusto S. Auler, Hai Cheng, Xinggong Kong, Yongjin Wang, Francisco W. Cruz, Jeffrey A. Dorale, and Hong-Wei Chiang. 2017. "Hydroclimate Changes Across the Amazon Lowlands over the Past 45,000 Years." *Nature* 541 (7636): 204–7. https://doi.org/10.1038/nature20787.

Wang, Y., U. Herzschuh, L. S. Shumilovskikh, S. Mischke, H. J. B. Birks, J. Wischnewski, J. Böhner, et al. 2014. "Quantitative Reconstruction of Precipitation Changes on the NE Tibetan Plateau Since the Last Glacial Maximum – Extending the Concept of Pollen Source Area to Pollen-Based Climate Reconstructions from Large Lakes." *Climate of the Past* 10 (1): 21–39. https://doi.org/10.5194/cp-10-21-2014.

Wang, Yongjin, Hai Cheng, R. Lawrence Edwards, Yaoqi He, Xinggong Kong, Zhisheng An, Jiangying Wu, Megan J. Kelly, Carolyn A. Dykoski, and Xiangdong Li. 2005. "The Holocene Asian Monsoon: Links to Solar Changes and North Atlantic Climate." *Science* 308 (5723): 854–57. https://doi.org/10.1126/science.1106296.

Wang, Yue, Jacquelyn L. Gill, Jeremiah Marsicek, Anna Dierking, Bryan Shuman, and John W Williams. 2015. "Pronounced Variations in Fagus Grandifolia Abundances in the Great Lakes Region During the Holocene." *The Holocene* 26 (4): 578–91. https://doi.org/10.1177/0959683615612586.

Wang, Zhenjun, Shitao Chen, Yongjin Wang, Kan Zhao, Yijia Liang, Hai Cheng, Qingfeng Shao, et al. 2022. "A High-Resolution Stalagmite Record from Luoshui Cave, Central China over the Past 23.5 Kyr." *Quaternary Science Reviews* 282 (April): 107443. https://doi.org/10.1016/j.quascirev.2022.107443.

Ward, Brittany Marie, Corinne I. Wong, Valdir F. Novello, David McGee, Roberto V. Santos, Lucas C. R. Silva, Francisco W. Cruz, Xianfeng Wang, R. Lawrence Edwards, and Hai Cheng. 2019a. "Reconstruction of Holocene Coupling Between the South American Monsoon System and Local Moisture Variability from Speleothem d18O and 87Sr/86Sr Records." *Quaternary Science Reviews* 210 (April): 51–63. https://doi.org/10.1016/j.quascirev.2019.02.019.

———. 2019b. "Reconstruction of Holocene Coupling Between the South American Monsoon System and Local Moisture Variability from Speleothem δ18O and 87Sr/86Sr Records." *Quaternary Science Reviews* 210 (April): 51–63. https://doi.org/10.1016/j.quascirev.2019.02.019.

Wassenburg, Jasper A., Stephan Dietrich, Jan Fietzke, Jens Fohlmeister, Klaus Peter Jochum, Denis Scholz, Detlev K. Richter, et al. 2016. "Reorganization of the North Atlantic Oscillation During Early Holocene Deglaciation." *Nature Geoscience* 9 (8): 602–5. https://doi.org/10.1038/ngeo2767.

Watanabe, O., J. Jouzel, S. Johnsen, F. Parrenin, H. Shoji, and N. Yoshida. 2003. "Homogeneous Climate Variability Across East Antarctica over the Past Three Glacial Cycles." *Nature* 422 (6931): 509–12. https://doi.org/10.1038/nature01525.

WATKINS, RUTH, JAMES D. SCOURSE, and JUDY R. M. ALLEN. 2007. "The Holocene Vegetation History of the Arfon Platform, North Wales, UK." *Boreas* 36 (2): 170–81. https://doi.org/10.1111/j.1502-3885.2007.tb01190.x.

Watson, Benjamin I., John W. Williams, James M. Russell, Stephen T. Jackson, Linda Shane, and Thomas V. Lowell. 2018. "Temperature Variations in the Southern Great Lakes During the Last Deglaciation: Comparison Between Pollen and GDGT Proxies." *Quaternary Science Reviews* 182 (February): 78–92. https://doi.org/10.1016/j.quascirev.2017.12.011.

Watson, Clare S. 1996. "The Vegetational History of the Northern Apennines, Italy: Information from Three New Sequences and a Review of Regional Vegetational Change." *Journal of Biogeography* 23 (6): 805–41. https://doi.org/10.1111/j.1365-2699.1996.tb00041.x.

Watts, W. A. 1979. "Late Quaternary Vegetation of Central Appalachia and the New Jersey Coastal Plain." *Ecological Monographs* 49 (4): 427–69. https://doi.org/10.2307/1942471.

WATTS, W. A., and R. C. BRIGHT. 1968. "Pollen, Seed, and Mollusk Analysis of a Sediment Core from Pickerel Lake, Northeastern South Dakota." *Geological Society of America Bulletin* 79 (7): 855. https://doi.org/10.1130/0016-7606(1968)79[855:psamao]2.0.co;2.

Weber, Michael, Denis Scholz, Andrea Schröder-Ritzrau, Michael Deininger, Christoph Spötl, Federico Lugli, Regina Mertz-Kraus, et al. 2018. "Evidence of Warm and Humid Interstadials in Central Europe During Early MIS 3 Revealed by a Multi-Proxy Speleothem Record." *Quaternary Science Reviews* 200 (November): 276–86. https://doi.org/10.1016/j.quascirev.2018.09.045.

Wegmuller, S. 1977. "Pollenanalytische Untersuchungen Zur Spat-Und Postglazialen Vegetationsgeschichte Der Franzosischen Alpen (Dauphine)." *Verlag Paul Haupt Bern Edition. Bern*.

Weijers, Johan W. H., Stefan Schouten, Enno Schefuß, Ralph R. Schneider, and Jaap S. Sinninghe Damst'e. 2009. "Disentangling Marine, Soil and Plant Organic Carbon Contributions to Continental Margin Sediments: A Multi-Proxy Approach in a 20,000 Year Sediment Record from the Congo Deep-Sea Fan." *Geochimica Et Cosmochimica Acta* 73 (1): 119–32. https://doi.org/10.1016/j.gca.2008.10.016.

Weirich, J., and S. Bortenschlager. 1980. "Beitrage Zur Vegetationsgeschichte Tirols III: Stubaier Alpen - Zillertaler Alpen." *Berichte Des Naturwissenschaftlich-Medizinischen Vereins in Innsbruck*.

Weldeab, Syee, David W. Lea, Hedi Oberhänsli, and Ralph R. Schneider. 2014. "Links Between Southwestern Tropical Indian Ocean SST and Precipitation over Southeastern Africa over the Last 17kyr." *Palaeogeography, Palaeoclimatology, Palaeoecology* 410 (September): 200–212. https://doi.org/10.1016/j.palaeo.2014.06.001.

Weldeab, Syee, David W. Lea, Ralph R. Schneider, and Nils Andersen. 2007. "155,000 Years of West African Monsoon and Ocean Thermal Evolution." *Science* 316 (5829): 1303–7. https://doi.org/10.1126/science.1140461.

Wells, Stephen G., William J. Brown, Yehouda Enzel, Roger Y. Anderson, and Leslie D. McFadden. 2003. "Late Quaternary Geology and Paleohydrology of Pluvial Lake Mojave, Southern California." In *Paleoenvironments and Paleohydrology of the Mojave and Southern Great Basin Deserts*. Geological Society of America. https://doi.org/10.1130/0-8137-2368-x.79.

Welten, M. 1977. "Resultats Palynologiques Sur Le Developpement de La Vegetation Et Sa Degradation Par l'homme Al'etage Inferieur Du Valais Central (Suisse)." *Approche Ecologique de l'homme Fossile*.

Welten, Max. 1982. *Vegetationsgeschichtliche Untersuchungen in Den Westlichen Schweizer Alpen: Bern-Wallis*. Birkhäuser Basel. https://doi.org/10.1007/978-3-0348-5359-0.

Wen, R., J. Xiao, Z. Chang, D. Zhai, X. Xu, Y. LI, and S. Itoh. 2010. "Holocene Precipitation and Temperature Variations in the East Asian Monsoonal Margin from Pollen Data from Hulun Lake in Northeastern Inner Mongolia, China." *Boreas* 39 (2): 262–72. https://doi.org/10.1111/j.1502-3885.2009.00125.x.

Weninger, J. M., and J. H. McAndrews. 1989. "Late Holocene Aggradation in the Lower Humber River Valley, Toronto, Ontario." *Canadian Journal of Earth Sciences* 26 (9): 1842–49. https://doi.org/10.1139/e89-157.

Werner, Kirstin, Robert F. Spielhagen, Dorothea Bauch, H. Christian Hass, and Evgeniya Kandiano. 2013. "Atlantic Water Advection Versus Sea-ice Advances in the Eastern Fram Strait During the Last 9 Ka: Multiproxy Evidence for a Two-phase Holocene." *Paleoceanography* 28 (2): 283–95. https://doi.org/10.1002/palo.20028.

Werner, K., J. Müller, K. Husum, R. F. Spielhagen, E. S. Kandiano, and L. Polyak. 2016. "Holocene Sea Subsurface and Surface Water Masses in the Fram Strait - Comparisons of Temperature and Sea-Ice Reconstructions." *Quaternary Science Reviews* 147 (September): 194–209. https://doi.org/10.1016/j.quascirev.2015.09.007.

Werner, K., P. E. Tarasov, A. A. Andreev, S. Maller, F. Kienast, M Zech, W. Zech, and B. Diekmann. 2010. "A 12.5-Kyr History of Vegetation Dynamics and Mire Development with Evidence of Younger Dryas Larch Presence in the Verkhoyansk Mountains, East Siberia, Russia." *Boreas* 39 (1): 56–68. https://doi.org/10.1111/j.1502-3885.2009.00116.x.

White, James M., and Rolf W. Mathewes. 1986. "Postglacial Vegetation and Climatic Change in the Upper Peace River District, Alberta." *Canadian Journal of Botany* 64 (10): 2305–18. https://doi.org/10.1139/b86-302.

Whitehead, Donald R. 1979. "Late-Glacial and Postglacial Vegetational History of the Berkshires, Western Massachusetts." *Quaternary Research* 12 (3): 333–57. https://doi.org/10.1016/0033-5894(79)90033-4.

Whitlock, Cathy, Walter E. Dean, Sherilyn C. Fritz, Lora R. Stevens, Jeffery R. Stone, Mitchell J. Power, Joseph R. Rosenbaum, Kenneth L. Pierce, and Brandi B. Bracht-Flyr. 2012. "Holocene Seasonal Variability Inferred from Multiple Proxy Records from Crevice Lake, Yellowstone National Park, USA." *Palaeogeography, Palaeoclimatology, Palaeoecology* 331-332 (May): 90–103. https://doi.org/10.1016/j.palaeo.2012.03.001.

Whittaker, Thomas E., Chris H. Hendy, and John C. Hellstrom. 2011a. “Abrupt Millennial-Scale Changes in Intensity of Southern Hemisphere Westerly Winds During Marine Isotope Stages 2-4.” *Geology* 39 (5): 455–58. https://doi.org/10.1130/g31827.1.
———. 2011b. “Abrupt Millennial-Scale Changes in Intensity of Southern Hemisphere Westerly Winds During Marine Isotope Stages 2–4.” *Geology* 39 (5): 455–58. https://doi.org/10.1130/g31827.1.
Wilkins, Daniel, Chris Gouramanis, Patrick De Deckker, L Keith Fifield, and Jon Olley. 2013. “Holocene Lake-Level Fluctuations in Lakes Keilambete and Gnotuk, Southwestern Victoria, Australia.” *The Holocene* 23 (6): 784–95. https://doi.org/10.1177/0959683612471983.
Willard, D. A., C. Bernhardt, D. Korejwo, and S. Meyers. 2005. “Impact of Millennial-Scale Holocene Climate Variability on Eastern North American Terrestrial Ecosystems: Pollen-Based Climatic Reconstruction.” *Global and Planetary Change* 47 (1): 17–35. https://doi.org/10.1016/j.gloplacha.2004.11.017.
Willard, D. A., T. M. Cronin, and S. Verardo. 2003. “Late-Holocene Climate and Ecosystem History from Chesapeake Bay Sediment Cores, USA.” *The Holocene* 13 (2): 201–14. https://doi.org/10.1191/0959683603hl607rp.
Willard, D. A., and L. M. Weimer. 1997. “Palynological Census Data from Surface Samples in South Florida.” *US Geological Survey Open-File Report*.
Willemse, Nico W., and Torbjörn E. Törnqvist. 1999. “Holocene Century-Scale Temperature Variability from West Greenland Lake Records.” *Geology* 27 (7): 580. https://doi.org/10.1130/0091-7613(1999)027<0580:hcstvf>2.3.co;2.
Williams, P. W., D. N. T. King, J.-X. Zhao, and K. D. Collerson. 2005. “Late Pleistocene to Holocene Composite Speleothem d18O and d13C Chronologies from South Island, New Zealand-Did a Global Younger Dryas Really Exist?” *Earth and Planetary Science Letters* 230 (3-4): 301–17. https://doi.org/10.1016/j.epsl.2004.10.024.
WILLIS, K. J. 1992. “The Late Quaternary Vegetational History of Northwest Greece. III. A Comparative Study of Two Contrasting Sites.” *New Phytologist* 121 (1): 139–55. https://doi.org/10.1111/j.1469-8137.1992.tb01099.x.
Willutzki, H, and 1962. Zur Waldgeschichte und Vermoorung. NA. “Missing Title.” *Unknown*, NA.
Winkler, Marjorie Green. 1985. “A 12,000-Year History of Vegetation and Climate for Cape Cod, Massachusetts.” *Quaternary Research* 23 (3): 301–12. https://doi.org/10.1016/0033-5894(85)90037-7.
Winter, Amos, Davide Zanchettin, Matthew Lachniet, Rolf Vieten, Francesco S. R. Pausata, Fredrik Charpentier Ljungqvist, Hai Cheng, et al. 2020. “Initiation of a Stable Convective Hydroclimatic Regime in Central America Circa 9000 Years BP.” *Nature Communications* 11 (1). https://doi.org/10.1038/s41467-020-14490-y.
Wirth, Stefanie B., Lukas Glur, Adrian Gilli, and Flavio S. Anselmetti. 2013. “Holocene Flood Frequency Across the Central Alps – Solar Forcing and Evidence for Variations in North Atlantic Atmospheric Circulation.” *Quaternary Science Reviews* 80 (November): 112–28. https://doi.org/10.1016/j.quascirev.2013.09.002.
Wogau, Kurt H., Helge W Arz, Harald N. Böhnel, Norbert R. Nowaczyk, and Jungjae Park. 2019. “High Resolution Paleoclimate and Paleoenvironmental Reconstruction in the Northern Mesoamerican Frontier for Prehistory to Historical Times.” *Quaternary Science Reviews* 226 (December): 106001. https://doi.org/10.1016/j.quascirev.2019.106001.
Wolff, Christian, Birgit Plessen, Alexey S Dudashvilli, Sebastian FM Breitenbach, Hai Cheng, Lawrence R Edwards, and Manfred R Strecker. 2016. “Precipitation Evolution of Central Asia During the Last 5000 Years.” *The Holocene* 27 (1): 142–54. https://doi.org/10.1177/0959683616652711.
Wolters, S. 2002. “Vegetationsgeschichtliche Untersuchungen Zur Spatglazialen Und Holozanen Landschaftsenwicklung in Der Doberitzer Heide (Brandenburg).” *Dissertationes Botanicae*.
Wolters, Steffen, Manfred Zeiler, and Friederike Bungenstock. 2009. “Early Holocene Environmental History of Sunken Landscapes: Pollen, Plant Macrofossil and Geochemical Analyses from the Borkum Riffgrund, Southern North Sea.” *International Journal of Earth Sciences* 99 (8): 1707–19. https://doi.org/10.1007/s00531-009-0477-6.
Wong, Corinne I., Jay L. Banner, and MaryLynn Musgrove. 2015. “Holocene Climate Variability in Texas, USA: An Integration of Existing Paleoclimate Data and Modeling with a New, High-Resolution Speleothem Record.” *Quaternary Science Reviews* 127 (November): 155–73. https://doi.org/10.1016/j.quascirev.2015.06.023.
Wong, Minn Lin, Xianfeng Wang, Edgardo M. Latrubesse, Shaoneng He, and Maximiliano Bayer. 2021a. “Variations in the South Atlantic Convergence Zone over the Mid-to-Late Holocene Inferred from

Speleothem d18O in Central Brazil." *Quaternary Science Reviews* 270 (October): 107178. https://doi.org/10.1016/j.quascirev.2021.107178.
———. 2021b. "Variations in the South Atlantic Convergence Zone over the Mid-to-Late Holocene Inferred from Speleothem δ18O in Central Brazil." *Quaternary Science Reviews* 270 (October): 107178. https://doi.org/10.1016/j.quascirev.2021.107178.
Wortham, Barbara E., Corinne I. Wong, Lucas C. R. Silva, David McGee, Isabel P. Montañez, E. Troy Rasbury, Kari M. Cooper, Warren D. Sharp, Justin J. G. Glessner, and Roberto V. Santos. 2017. "Assessing Response of Local Moisture Conditions in Central Brazil to Variability in Regional Monsoon Intensity Using Speleothem 87Sr/86Sr Values." *Earth and Planetary Science Letters* 463 (April): 310–22. https://doi.org/10.1016/j.epsl.2017.01.034.
Wright, Aaron M. 2012. "Low-Frequency Climate in the Mesa Verde Region." In *Emergence and Collapse of Early Villages: Models of Central Mesa Verde Archaeology*, edited by Timothy A. Kohler and Mark D. Varien, 41–58. University of California Press.
Wright Jr, H. E., and W. A. Watts. 1969. "Glacial and Vegetational History of Northeastern Minnesota." *Special Publications Series 11. Minnesota Geological Survey*.
Wu, L, X. Y Wang, G. S. Zhang, and X. Y. Xiao. 2008. "Vegetation Evolution and Climate Change Since the Holocene Recorded by Pollen-Charcoal Assemblages from Lacustrine Sediments of Chaohu Lake in Anhui Province." *Journal of Palaeogeography*.
Wündsch, Michael, Torsten Haberzettl, Hayley C. Cawthra, Kelly L. Kirsten, Lynne J. Quick, Matthias Zabel, Peter Frenzel, et al. 2018a. "Holocene Environmental Change Along the Southern Cape Coast of South Africa - Insights from the Eilandvlei Sediment Record Spanning the Last 8.9 Kyr." *Global and Planetary Change* 163 (April): 51–66. https://doi.org/10.1016/j.gloplacha.2018.02.002.
———, et al. 2018b. "Holocene Environmental Change Along the Southern Cape Coast of South Africa – Insights from the Eilandvlei Sediment Record Spanning the Last 8.9 Kyr." *Global and Planetary Change* 163 (April): 51–66. https://doi.org/10.1016/j.gloplacha.2018.02.002.
Wurtzel, Jennifer B., Nerilie J. Abram, Sophie C. Lewis, Petra Bajo, John C. Hellstrom, Ulrike Troitzsch, and David Heslop. 2018. "Tropical Indo-Pacific Hydroclimate Response to North Atlantic Forcing During the Last Deglaciation as Recorded by a Speleothem from Sumatra, Indonesia." *Earth and Planetary Science Letters* 492 (June): 264–78. https://doi.org/10.1016/j.epsl.2018.04.001.
Xiangdong, Yang, and Zhu Yuxin, Jiang Xuezhong, Wu Yanhong, and Wang Sumin. 1998. "Environmental Changes from Spore-Pollen Record of Mianyang Region over the Past 10000 Years." *Journal of Lake Sciences* 10 (2): 23–29. https://doi.org/10.18307/1998.0205.
Xiao, Jule, Shengrui Zhang, Jiawei Fan, Ruilin Wen, Qinghai Xu, Yoshio Inouchi, and Toshio Nakamura. 2019. "The 4.2 Ka Event and Its Resulting Cultural Interruption in the Daihai Lake Basin at the East Asian Summer Monsoon Margin." *Quaternary International* 527 (August): 87–93. https://doi.org/10.1016/j.quaint.2018.06.025.
Xie, Y. Y, C. A Li, Q. L. Wang, and H. F. Yin. 2008. "Palynological Records of Early Human Activities in Holocene at Jiangling Area, Hubei Province." *Scientia Geographica Sinica*.
Xu, Hai, Yonaton Goldsmith, Jianghu Lan, Liangcheng Tan, Xulong Wang, Xinying Zhou, Jun Cheng, Yunchao Lang, and Congqiang Liu. 2020. "Juxtaposition of Western Pacific Subtropical High on Asian Summer Monsoon Shapes Subtropical East Asian Precipitation." *Geophysical Research Letters* 47 (3). https://doi.org/10.1029/2019gl084705.
Xu, Jian, Ann Holbourn, Wolfgang Kuhnt, Zhimin Jian, and Hiroshi Kawamura. 2008. "Changes in the Thermocline Structure of the Indonesian Outflow During Terminations i and II." *Earth and Planetary Science Letters* 273 (1-2): 152–62. https://doi.org/10.1016/j.epsl.2008.06.029.
Xu, Q. H, Z. C Kong, X. D Chen, X. L Yang, W. D. Liang, and L. M. Sun. 2002. "Discussion on the Environment Changes and the Effects of Human Impacts in the East Ordos Plateau Since 4000 a BP." *Quaternary Sciences*.
Pokorný Petr. 2002. "A High-Resolution Record of Late-Glacial and Early-Holocene Climatic and Environmental Change in the Czech Republic." *Quaternary International* 91 (1): 101–22. https://doi.org/10.1016/s1040-6182(01)00105-7.
Pokorný, Petr, Nicole Boenke, Miloslav Chytr'aček, Kateřina Nov'akov'a, Jiř'ıS'adlo, Josef Vese ý, Petr Kuneš, and Vlasta Jankovsk'a. 2006. "Insight into the Environment of a Pre-Roman Iron Age Hillfort at Vladař, Czech Republic, Using a Multi-Proxy Approach." *Vegetation History and Archaeobotany* 15 (4): 419–33. https://doi.org/10.1007/s00334-006-0064-8.

Pokorný, Petr, and Willem O. van der Knaap. 2010. “10. Na Bahně (Czech Republic): Vegetation Development over the Last 2.5 Millennia in the Eastern Bohemian Lowland.” *Grana* 49 (1): 79–81. https://doi.org/10.1080/00173130903435846.

Yan, Dada, and Bernd Wünnemann. 2014. “Late Quaternary Water Depth Changes in Hala Lake, Northeastern Tibetan Plateau, Derived from Ostracod Assemblages and Sediment Properties in Multiple Sediment Records.” *Quaternary Science Reviews* 95 (July): 95–114. https://doi.org/10.1016/j.quascirev.2014.04.030.

Yan, S, S. F Li, Z. C Kong, Z. J. Yang, and J. Ni. 2004. “The Pollen Analyses and Environment Changes of the Dongdaohaizi Area in Urumqi, Xinjiang.” *Quaternary Sciences*.

Yancheva, Gergana, Norbert R. Nowaczyk, Jens Mingram, Peter Dulski, Georg Schettler, Jörg F. W. Negendank, Jiaqi Liu, Daniel M. Sigman, Larry C. Peterson, and Gerald H. Haug. 2007. “Influence of the Intertropical Convergence Zone on the East Asian Monsoon.” *Nature* 445 (7123): 74–77. https://doi.org/10.1038/nature05431.

Yang, Bao, Chun Qin, Achim Bräuning, Timothy J. Osborn, Valerie Trouet, Fredrik Charpentier Ljungqvist, Jan Esper, et al. 2021. “Long-Term Decrease in Asian Monsoon Rainfall and Abrupt Climate Change Events over the Past 6,700 Years.” *Proceedings of the National Academy of Sciences* 118 (30). https://doi.org/10.1073/pnas.2102007118.

Yang, X. D, Y. X Zhu, X. Z Jiang, Y. H. Wu, and S. M. Wang. 1998. “Environmental Changes from Spore-Pollen Record of Mianyang Region over the Past 10000 Years.” *Journal of Lake Sciences*.

Yang, Xunlin, Hong Yang, Baoyan Wang, Li-Jung Huang, Chuan-Chou Shen, R Lawrence Edwards, and Hai Cheng. 2019. “Early-Holocene Monsoon Instability and Climatic Optimum Recorded by Chinese Stalagmites.” *The Holocene* 29 (6): 1059–67. https://doi.org/10.1177/0959683619831433.

Yang, Yan, DaoXian Yuan, Hai Cheng, MeiLiang Zhang, JiaMing Qin, YuShi Lin, XiaoYan Zhu, and R. Lawrence Edwards. 2010. “Precise Dating of Abrupt Shifts in the Asian Monsoon During the Last Deglaciation Based on Stalagmite Data from Yamen Cave, Guizhou Province, China.” *Science China Earth Sciences* 53 (5): 633–41. https://doi.org/10.1007/s11430-010-0025-z.

Yao, Qiang, Kam-biu Liu, William J. Platt, and Victor H. Rivera-Monroy. 2015. “Palynological Reconstruction of Environmental Changes in Coastal Wetlands of the Florida Everglades Since the Mid-Holocene.” *Quaternary Research* 83 (3): 449–58. https://doi.org/10.1016/j.yqres.2015.03.005.

Yll, E. I, R. P Perez-Obiol, J. Pantaleon-Cano, and J. M. Roure. 1995a. “Dinamica Del Paisaje Vegetal En La Vertiente Mediterranea de La Peninsula Iberica e Islas Baleares Desde El Tardig Glaciar Hasta El Presente.” *In: Reconstitution de La Ambient Es y Cambios Climaticos Durante El Cuaternarion*.

Yll, E. I, R Perez-Obiol, J. Pantaleon-Cano, and J. M. Roure. 1995b. “Dinamica Del Paisaje Vegetal En La Vertiente Mediterranea de La Peninsula Iberica e Islas Baleares Desde El Tardiglaciar Hasta El Presente.” *In: Reconstitution de La Ambient Es y Cambios Climaticos Durante El Cuaternarion*.

Yll, Errikarta-Imanol, Ramon Perez-Obiol, Jose Pantaleon-Cano, and Joan Maria Roure. 1997. “Palynological Evidence for Climatic Change and Human Activity During the Holocene on Minorca (Balearic Islands).” *Quaternary Research* 48 (3): 339–47. https://doi.org/10.1006/qres.1997.1925.

Yoo, Dong G., Seong P. Kim, Tae S. Chang, Gee S. Kong, Nyeon K. Kang, Yi K. Kwon, Seung L. Nam, and Soo C. Park. 2014a. “Late Quaternary Inner Shelf Deposits in Response to Late Pleistocene - Holocene Sea Level Changes: Nakdong River, SE Korea.” *Quaternary International* 344 (September): 156–69. https://doi.org/10.1016/j.quaint.2014.02.004.

———. 2014b. “Late Quaternary Inner Shelf Deposits in Response to Late Pleistocene–Holocene Sea Level Changes: Nakdong River, SE Korea.” *Quaternary International* 344 (September): 156–69. https://doi.org/10.1016/j.quaint.2014.02.004.

Yu, Shi-Yong. 2013. “Quantitative Reconstruction of Mid- to Late-Holocene Climate in NE China from Peat Cellulose Stable Oxygen and Carbon Isotope Records and Mechanistic Models.” *The Holocene* 23 (11): 1507–16. https://doi.org/10.1177/0959683613496292.

Yu, Xuefeng, Weijian Zhou, Lars G. Franzen, Feng Xian, Peng Cheng, and A. J. Tim Jull. 2006. “High-Resolution Peat Records for Holocene Monsoon History in the Eastern Tibetan Plateau.” *Science in China Series D* 49 (6): 615–21. https://doi.org/10.1007/s11430-006-0615-y.

Yu, Zicheng. 2007. “Rapid Response of Forested Vegetation to Multiple Climatic Oscillations During the Last Deglaciation in the Northeastern United States.” *Quaternary Research* 67 (2): 297–303. https://doi.org/10.1016/j.yqres.2006.08.006.

Yu, Zicheng, Ian D. Campbell, Celina Campbell, Dale H. Vitt, Gerard C. Bond, and Michael J. Apps. 2003. “Carbon Sequestration in Western Canadian Peat Highly Sensitive to Holocene Wet-Dry Climate Cycles at Millennial Timescales.” *The Holocene* 13 (6): 801–8. https://doi.org/10.1191/0959683603hl667ft.

Yu, Zicheng, and John H. McAndrews. 1994. “Holocene Water Levels at Rice Lake, Ontario, Canada: Sediment, Pollen and Plant-Macrofossil Evidence.” *The Holocene* 4 (2): 141–52. https://doi.org/10.1177/095968369400400204.

Yuan, Daoxian, Hai Cheng, R. Lawrence Edwards, Carolyn A. Dykoski, Megan J. Kelly, Meiliang Zhang, Jiaming Qing, et al. 2004. “Timing, Duration, and Transitions of the Last Interglacial Asian Monsoon.” *Science* 304 (5670): 575–78. https://doi.org/10.1126/science.1091220.

Yuan, Fasong, Max R. Koran, and Andrew Valdez. 2013. “Late Glacial and Holocene Record of Climatic Change in the Southern Rocky Mountains from Sediments in San Luis Lake, Colorado, USA.” *Palaeogeography, Palaeoclimatology, Palaeoecology* 392 (December): 146–60. https://doi.org/10.1016/j.palaeo.2013.09.016.

Yue, Yuanfu, Zhuo Zheng, Kangyou Huang, Manuel Chevalier, Brian M. Chase, Matthieu Carr’e, Marie-Pierre Ledru, and Rachid Cheddadi. 2012. “A Continuous Record of Vegetation and Climate Change over the Past 50,000years in the Fujian Province of Eastern Subtropical China.” *Palaeogeography, Palaeoclimatology, Palaeoecology* 365-366 (December): 115–23. https://doi.org/10.1016/j.palaeo.2012.09.018.

Zanchetta, G., R. N. Drysdale, J. C. Hellstrom, A. E. Fallick, I. Isola, M. K. Gagan, and M. T. Pareschi. 2007. “Enhanced Rainfall in the Western Mediterranean During Deposition of Sapropel S1: Stalagmite Evidence from Corchia Cave (Central Italy).” *Quaternary Science Reviews* 26 (3-4): 279–86. https://doi.org/10.1016/j.quascirev.2006.12.003.

Zanchetta, Giovanni, Aurelien Van Welden, Ilaria Baneschi, Russell Drysdale, Laura Sadori, Neil Roberts, Marco Giardini, Christian Beck, Vincenzo Pascucci, and Roberto Sulpizio. 2012. “Multiproxy Record for the Last 4500 Years from Lake Shkodra (Albania/Montenegro).” *Journal of Quaternary Science* 27 (8): 780–89. https://doi.org/10.1002/jqs.2563.

Zanchetta, G., E. Regattieri, I. Isola, R. N. Drysdale, M. Bini, I. Baneschi, and J. C. Hellstrom. 2016. “The so-Called "4.2 Event" in the Central Mediterranean and Its Climatic Teleconnections.” *Alpine and Mediterranean Quaternary* 29: 5–17.

Zanon, Marco, Ingmar Unkel, Nils Andersen, and Wiebke Kirleis. 2019. “Palaeoenvironmental Dynamics at the Southern Alpine Foothills Between the Neolithic and the Bronze Age Onset. A Multi-Proxy Study from Bande Di Cavriana (Mantua, Italy).” *Quaternary Science Reviews* 221 (October): 105891. https://doi.org/10.1016/j.quascirev.2019.105891.

Zaragosi, S., F. Eynaud, C. Pujol, G. A. Auffret, J.-L. Turon, and T. Garlan. 2001. “Initiation of the European Deglaciation as Recorded in the Northwestern Bay of Biscay Slope Environments (Meriadzek Terrace and Trevelyan Escarpment): A Multi-Proxy Approach.” *Earth and Planetary Science Letters* 188 (3-4): 493–507. https://doi.org/10.1016/s0012-821x(01)00332-6.

Zatyko, C. S., I. Juhasz, and P. Suemegi. 2007. “Environmental Archaeology in Transdanubia.” *Unknown*.

Zerathe, Swann, Thomas Lebourg, R’egis Braucher, and Didier Bourl‘es. 2014. “Mid-Holocene Cluster of Large-Scale Landslides Revealed in the Southwestern Alps by 36Cl Dating. Insight on an Alpine-Scale Landslide Activity.” *Quaternary Science Reviews* 90 (April): 106–27. https://doi.org/10.1016/j.quascirev.2014.02.015.

Zernitskaya, V. P, E. A. Krutous, and V. A. Klimanov. 1988. “Studies of Mire for the Purpose of Reconstruction of the Climatic Peculiarities of the Byelorussian Poles’e.” *In: Problem of the Practical Geomorphology (Pp.68-73). Nauka i Tekhnika*.

Zhang, Enlou, Jie Chang, Yanmin Cao, Weiwei Sun, James Shulmeister, Hongqu Tang, Peter G. Langdon, Xiangdong Yang, and Ji Shen. 2017. “Holocene High-Resolution Quantitative Summer Temperature Reconstruction Based on Subfossil Chironomids from the Southeast Margin of the Qinghai-Tibetan Plateau.” *Quaternary Science Reviews* 165 (June): 1–12. https://doi.org/10.1016/j.quascirev.2017.04.008.

Zhang, Guangsheng, Cheng Zhu, Jihuai Wang, Guangyao Zhu, Chunmei Ma, Chaogui Zheng, Lanhui Zhao, Zhongxuan Li, Lan Li, and Aichun Jin. 2010. “Environmental Archaeology on Longshan Culture (4500–4000 aBP) at Yuhuicun Site in Bengbu, Anhui Province.” *Journal of Geographical Sciences* 20 (3): 455–68. https://doi.org/10.1007/s11442-010-0455-8.

Zhang, Haiwei, Hai Cheng, Yanjun Cai, Christoph Spötl, Gayatri Kathayat, Ashish Sinha, R. Lawrence Edwards, and Liangcheng Tan. 2018. “Hydroclimatic Variations in Southeastern China During the 4.2 Ka Event Reflected by Stalagmite Records.” *Climate of the Past* 14 (11): 1805–17. https://doi.org/10.5194/cp-14-1805-2018.

Zhang, Hongbin, Michael L. Griffiths, John C. H. Chiang, Wenwen Kong, Shitou Wu, Alyssa Atwood, Junhua Huang, Hai Cheng, Youfeng Ning, and Shucheng Xie. 2018. “East Asian Hydroclimate Modulated by the

Position of the Westerlies During Termination i." *Science* 362 (6414): 580–83. https://doi.org/10.1126/science.aat9393.
Zhang, Hui-Ling, Ke-Fu Yu, Jian-Xin Zhao, Yue-Xing Feng, Yu-Shi Lin, Wei Zhou, and Guo-Hui Liu. 2013a. "East Asian Summer Monsoon Variations in the Past 12.5ka: High-Resolution d18O Record from a Precisely Dated Aragonite Stalagmite in Central China." *Journal of Asian Earth Sciences* 73 (September): 162–75. https://doi.org/10.1016/j.jseaes.2013.04.015.
———. 2013b. "East Asian Summer Monsoon Variations in the Past 12.5ka: High-Resolution δ18O Record from a Precisely Dated Aragonite Stalagmite in Central China." *Journal of Asian Earth Sciences* 73 (September): 162–75. https://doi.org/10.1016/j.jseaes.2013.04.015.
Zhang, J. H, Z. C. Kong, and N. Q. Du. 1997. "An Analysis of Different Sedimentary Environment Influence on Pollen Deposit in Beijing." *Acta Sedimentologica Sinica*.
Zhang, Shengrui, Jule Xiao, Qinghai Xu, Ruilin Wen, Jiawei Fan, Yun Huang, Manyue Li, and Jian Liang. 2020. "Contrasting Impacts of the 8.2- and 4.2-ka Abrupt Climatic Events on the Regional Vegetation of the Hulun Lake Region in North-eastern China." *Journal of Quaternary Science* 35 (6): 831–40. https://doi.org/10.1002/jqs.3231.
Zhang, Y., Z. C. Kong, Z. J. Yang, S. Yan, and J. Ni. 2004. "Vegetation Changes and Environmental Evolution in the Urumqi River Head, Central Tianshan Mountains Since 3.6 Ka B.P.: A Case Study of Daxigou Profile." *Journal of Integrative Plant Biology* 46 (6): 655–67.
Zhang, Yun, Zhaochen Kong, Shun Yan, Zhenjing Yang, and Jian Ni. 2006. "Fluctuation of Picea Timber-Line and Paleo-Environment on the Northern Slope of Tianshan Mountains During the Late Holocene." *Chinese Science Bulletin* 51 (14): 1747–56. https://doi.org/10.1007/s11434-006-2029-9.
Zhang, Zhaohui, Guillaume Leduc, and Julian P. Sachs. 2014a. "El Nino Evolution During the Holocene Revealed by a Biomarker Rain Gauge in the Galapagos Islands." *Earth and Planetary Science Letters* 404 (October): 420–34. https://doi.org/10.1016/j.epsl.2014.07.013.
———. 2014b. "El Niño Evolution During the Holocene Revealed by a Biomarker Rain Gauge in the Galápagos Islands." *Earth and Planetary Science Letters* 404 (October): 420–34. https://doi.org/10.1016/j.epsl.2014.07.013.
Zhao, Cheng, Jun Cheng, Jingjing Wang, Hong Yan, Chengcheng Leng, Can Zhang, Xiaoping Feng, Weiguo Liu, Xiangdong Yang, and Ji Shen. 2021. "Paleoclimate Significance of Reconstructed Rainfall Isotope Changes in Asian Monsoon Region." *Geophysical Research Letters* 48 (12). https://doi.org/10.1029/2021gl092460.
Zhao, Cheng, Zicheng Yu, Yan Zhao, Emi Ito, Kenneth P. Kodama, and Fahu Chen. 2010. "Holocene Millennial-Scale Climate Variations Documented by Multiple Lake-Level Proxies in Sediment Cores from Hurleg Lake, Northwest China." *Journal of Paleolimnology* 44 (4): 995–1008. https://doi.org/10.1007/s10933-010-9469-6.
Zhao, M., N. A. S. Beveridge, N. J. Shackleton, M. Sarnthein, and G. Eglinton. 1995. "Molecular Stratigraphy of Cores Off Northwest Africa: Sea Surface Temperature History over the Last 80 Ka." *Paleoceanography* 10 (3): 661–75. https://doi.org/10.1029/94pa03354.
Zhao, Yan, Zicheng Yu, and Wenwei Zhao. 2011. "Holocene Vegetation and Climate Histories in the Eastern Tibetan Plateau: Controls by Insolation-Driven Temperature or Monsoon-Derived Precipitation Changes?" *Quaternary Science Reviews* 30 (9-10): 1173–84. https://doi.org/10.1016/j.quascirev.2011.02.006.
Zhu, Xiao Hong, Bing Li, Chun Mei Ma, Cheng Zhu, Li Wu, and Hui Liu. 2017. "Late Neolithic Phytolith and Charcoal Records of Human Activities and Vegetation Change in Shijiahe Culture, Tanjialing Site, China." Edited by ChengNANASen L. *PLOS ONE* 12 (5): e0177287. https://doi.org/10.1371/journal.pone.0177287.
Zhu, Zongmin, Joshua M. Feinberg, Shucheng Xie, Mark D. Bourne, Chunju Huang, Chaoyong Hu, and Hai Cheng. 2017. "Holocene ENSO-Related Cyclic Storms Recorded by Magnetic Minerals in Speleothems of Central China." *Proceedings of the National Academy of Sciences* 114 (5): 852–57. https://doi.org/10.1073/pnas.1610930114.
Zielhofer, Christoph, Anne Köhler, Steffen Mischke, Abdelfattah Benkaddour, Abdeslam Mikdad, and William J. Fletcher. 2019. "Western Mediterranean Hydro-Climatic Consequences of Holocene Ice-Rafted Debris (Bond) Events." *Climate of the Past* 15 (2): 463–75. https://doi.org/10.5194/cp-15-463-2019.
Zielhofer, Christoph, Hans von Suchodoletz, William J. Fletcher, Birgit Schneider, Elisabeth Dietze, Michael Schlegel, Kerstin Schepanski, Bernhard Weninger, Steffen Mischke, and Abdeslam Mikdad. 2017. "Millennial-Scale Fluctuations in Saharan Dust Supply Across the Decline of the African Humid Period." *Quaternary Science Reviews* 171 (September): 119–35. https://doi.org/10.1016/j.quascirev.2017.07.010.

Zolitschka, Bernd, An-Sheng Lee, Daniela Piraquive Berm’udez, and Thomas Giesecke. 2021. “Environmental Variability at the Margin of the South American Monsoon System Recorded by a High-Resolution Sediment Record from Lagoa Dourada (South Brazil).” *Quaternary Science Reviews* 272 (November): 107204. https://doi.org/10.1016/j.quascirev.2021.107204.

Zumaque, Jena, Fr’ed’erique Eynaud, and Anne de Vernal. 2017. “Holocene Paleoceanography of the Bay of Biscay: Evidence for West-East Linkages in the North Atlantic Based on Dinocyst Data.” *Palaeogeography, Palaeoclimatology, Palaeoecology* 468 (February): 403–13. https://doi.org/10.1016/j.palaeo.2016.12.031.
