# Supplementary material for: The 4.2 ka event is not remarkable in the context of Holocene climate variability
Source: Nat Commun. 2024 Aug 2;15:6555. doi: 10.1038/s41467-024-50886-w (PMC11297131; doi:10.1038/s41467-024-50886-w)
Supplement: Supplementary file 3 — Description of Additional Supplementary Files [file 41467_2024_50886_MOESM3_ESM.pdf]

Supplemental Data 1. Datasets used in excursion detection analysis. Metadata describing the datasets and timeseries used, including archive type, coordinates, and citation are included (bibliography included in supplementary information). The timing of significant excursions detected by the algorithm are also presented in yr BP.
